# Identification and Functional Characterization of N-Terminally Acetylated Proteins in *Drosophila melanogaster*

**DOI:** 10.1371/journal.pbio.1000236

**Published:** 2009-11-03

**Authors:** Sandra Goetze, Ermir Qeli, Christian Mosimann, An Staes, Bertran Gerrits, Bernd Roschitzki, Sonali Mohanty, Eva M. Niederer, Endre Laczko, Evy Timmerman, Vinzenz Lange, Ernst Hafen, Ruedi Aebersold, Joël Vandekerckhove, Konrad Basler, Christian H. Ahrens, Kris Gevaert, Erich Brunner

**Affiliations:** 1 Center for Model Organism Proteomes, University of Zurich, Switzerland; 2 Institute of Molecular Systems Biology, ETH Zurich, Switzerland; 3 Institute for Molecular Biology, University of Zurich, Switzerland; 4 Department of Medical Protein Research, Flanders Institute for Biotechnology, Ghent, Belgium; 5 Department of Biochemistry, Ghent University, Ghent, Belgium; 6 Functional Genomics Center, ETH and University of Zurich, Switzerland; 7 Faculty of Science, University of Zurich, Switzerland; 8 Institute for Systems Biology, Seattle, Washington, United States of America; University of Washington, United States of America

## Abstract

Protein modifications play a major role for most biological processes in living organisms. Amino-terminal acetylation of proteins is a common modification found throughout the tree of life: the N-terminus of a nascent polypeptide chain becomes co-translationally acetylated, often after the removal of the initiating methionine residue. While the enzymes and protein complexes involved in these processes have been extensively studied, only little is known about the biological function of such N-terminal modification events. To identify common principles of N-terminal acetylation, we analyzed the amino-terminal peptides from proteins extracted from *Drosophila* Kc167 cells. We detected more than 1,200 mature protein N-termini and could show that N-terminal acetylation occurs in insects with a similar frequency as in humans. As the sole true determinant for N-terminal acetylation we could extract the (X)PX rule that indicates the prevention of acetylation under all circumstances. We could show that this rule can be used to genetically engineer a protein to study the biological relevance of the presence or absence of an acetyl group, thereby generating a generic assay to probe the functional importance of N-terminal acetylation. We applied the assay by expressing mutated proteins as transgenes in cell lines and in flies. Here, we present a straightforward strategy to systematically study the functional relevance of N-terminal acetylations in cells and whole organisms. Since the (X)PX rule seems to be of general validity in lower as well as higher eukaryotes, we propose that it can be used to study the function of N-terminal acetylation in all species.

## Abstract 2

A new study reveals a functional rule for N-terminal acetylation in higher eukaryotes called the (X)PX rule and describes a generic method that prevents this modification to allow the study of N-terminal acetylation in any given protein.

## Author Summary

Widely hailed as the workhorses of the cell, proteins participate in virtually every process within a living organism. How well they perform these diverse tasks depends on successful passage through the intricate course of protein production, from transcription of the protein-encoded DNA template to processing and folding of the nascent amino acid chain. Some of the processing steps—including enzymatic cleavage or the attachment of chemical modifications—take place during protein synthesis, while others occur afterward. One modification that takes place during protein synthesis is the attachment of an acetyl group at the tip (N-terminus) of proteins. Although N-terminal acetylation is found throughout the tree of life and the machinery and mechanisms responsible for this modification are quite well characterized, little is known about how it affects protein function. We analyzed the acetylation state of proteins in the fruit fly *Drosophila melanogaster* and show that this modification occurs at a lower frequency in flies than in man but at a much higher frequency than in yeast. Based on our dataset we developed a generic method that can analyze the biological relevance of N-terminal protein acetylation in any organism.

## Introduction

To attain full functionality and/or to reach their final cellular localization, many proteins undergo obligatory modification or processing. During this maturation process, proteins are concurrently properly folded, proteolytically processed, and enzymatically modified. Some of these processes occur co-translationally, i.e. during protein synthesis, while others take place after protein synthesis has been completed. Acetylation of protein N-terminal α-amino groups takes place during protein synthesis [1]. This very common and irreversible modification of proteins often combines two consecutive events [2],[3]. In the first step, the N-terminal methionine (also referred to as initiator methionine [iMet]) is removed from the nascent polypeptide chain by methionine aminopeptidases. This event is not obligatory in protein biosynthesis and has been shown to take place only if the second amino acid is small and uncharged [4],[5]. Larger amino acids at this position prevent removal of iMet by steric hindrance [6]. In the second step, the acetylation of the amino-terminus is catalyzed by N-terminal acetyl transferases (NATs), a class of enzymes conserved in pro- and eukaryotes [7]–[11]. In eukaryotes both processes usually take place co-translationally on the nascent polypeptide chain and appear to be completed when 25–50 residues extrude from the ribosome, as revealed by in vitro studies [12],[13]. This indicates that the N-terminal region of a protein defines its acetylation status. Although previous work could show sequence specificities of the different NAT complexes, for some proteins acetylation does not take place even if the appropriate amino acid sequences are present, suggesting that additional yet unknown amino acid sequence patterns or other determinants like the secondary structure of the protein's N-terminus may play a role [10].

An estimated 60%–90% of the cytosolic proteins are acetylated at their N-terminus [3],[14], however the biological relevance of N-terminal acetylation has been determined only for a few proteins. This was in most cases achieved either through the analysis of mutants of NAT complex components [7], in vitro modification [5], or through mutants for single proteins [15]. Small GTPases such as Arl3p or Arl8 for instance require amino-terminal acetylation for their recruitment to Golgi membranes and lysosomes [15],[16]. In other cases, the acetylated N-terminus promotes protein-protein interactions as has been shown to be important for the binding of F-actin and tropomyosin and the maintenance of the resulting higher order structure [17],[18]. These examples clearly demonstrate that N-terminal acetylation promotes a variety of biological functions that cannot be predicted from the primary amino acid sequence. Therefore, there is a need for a method to generate and express—in cells and organisms—proteins that differ in N-terminal acetylation to investigate functional consequences of the presence or absence of an N-terminal acetyl group.

N-terminal acetylation has been identified in various organisms [10],[19]. A detailed analysis of NAT substrate specificity, sequence requirements, and conservation of substrate specificity for acetylation were only recently documented for yeast and human [11]. Datasets for invertebrates are not available and it has been suggested that acetylations in invertebrates appear to be rare [10]. Here we present an extensive compilation of mature protein N-termini of *Drosophila melanogaster* that was obtained by shotgun proteomics as well as the enrichment of N-terminal peptides by COFRADIC [20]. We show that amino-terminal acetylation is a common event in *Drosophila* and that the sequence requirements (amino acids) that promote iMet cleavage and N-terminal acetylation are similar to those in other eukaryotes. Moreover, our dataset enabled us to detect the use of 124 previously unknown alternative translation initiation sites and/or splice variants. A Pfam analysis [21] revealed that a protein's acetylation state in some cases strongly correlates with the presence of certain functional protein domains.

Finally, in contrast to earlier studies that were limited to the identification of amino acid determinants that promoted or inhibited N- terminal acetylation, in this study we could identify a definite determinant, i.e. a proline at position one or two of a nascent protein that prevents N-terminal acetylation under all circumstances. We refer to this finding as (X)PX rule. We have applied this rule to genetically modify a protein such that the biological relevance of N-terminal acetylation could be studied in cell lines and in flies. Since the (X)PX motif seems to be conserved among organisms we propose that by applying the (X)PX rule in similar ways in other species, the function of N-terminal acetylation can now be generically studied.

## Results

### Characterization of N-terminal Most Peptides in *Drosophila melanogaster*

**Figure 1:**
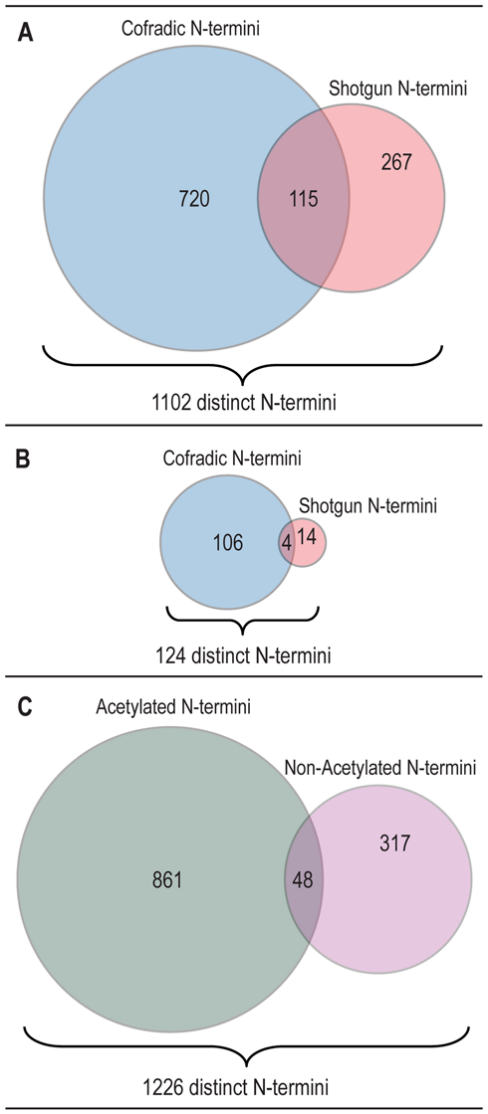
Graphical representation of the datasets using Venn diagrams. (A) Comparison of dataset generated by COFRADIC and shotgun analysis. A total of 1,102 distinct N-terminal peptides were identified with an overlap of 115 sequences. The COFRADIC approach yielded 835 N-terminal peptides among a total 4,203 distinct peptides identified from 8,402 spectra. In contrast, a classical shotgun approach on Kc cells not using COFRADIC enrichment yielded 382 N-terminal peptides among 19,915 distinct peptides (34,175 spectra) retrieved from Loevenich et al. [24]. (B) Identification of 124 distinct putative, alternative translation initiation sites identified in the COFRADIC and shotgun dataset, respectively. (C) Comparison of N-terminal peptides according to their acetylation status. From 1,226 distinct N-termini 861 were found to be acetylated, 317 non-acetylated with an overlap of 48 identical N-termini showing partial acetylation.

To enrich for N-terminal peptides, proteins from a membrane, cytoplasmic, and nuclear fraction of *Drosophila* Kc167 cells, respectively, were subjected to combined fractional diagonal chromatography (COFRADIC) [11],[20],[22]. In COFRADIC, free primary amino groups of proteins (i.e. α-N-termini and ε-amines from lysine residues) need to be chemically acetylated on the protein level. To further distinguish naturally acetylated and non-acetylated protein N-termini, protein amines were blocked by trideutero-acetylation, which leaves a mass tag of 3 Dalton on each free primary amino group [11],[23]. The fractions enriched for N-terminal peptides were then analyzed by mass spectrometry. We identified 835 N-terminal peptides (peptides starting at the residue 1 or 2 of the predicted sequence; Figure 1A, Table S1) among a total 4,203 distinct peptides (19.5%) identified from 8,402 fragment ion spectra. This corresponds to roughly 8.7% of the protein N-termini detectable by mass spectrometry (see Figure S1 for calculations). The actual coverage reached has to be considered much higher since only a subset of all annotated proteins will be expressed in exponentially growing Kc cells. Furthermore, a dataset consisting of 382 N-terminal peptides (Figure 1A) identified by a classical shotgun proteomics approach on Kc cells, that is not using COFRADIC, was additionally considered in subsequent analyses (retrieved from [24]). A comparison of the two datasets revealed that COFRADIC enriched for N-terminal peptides by a factor of roughly 10. In total the two datasets identified 1,102 protein N-termini.

**Figure 2:**
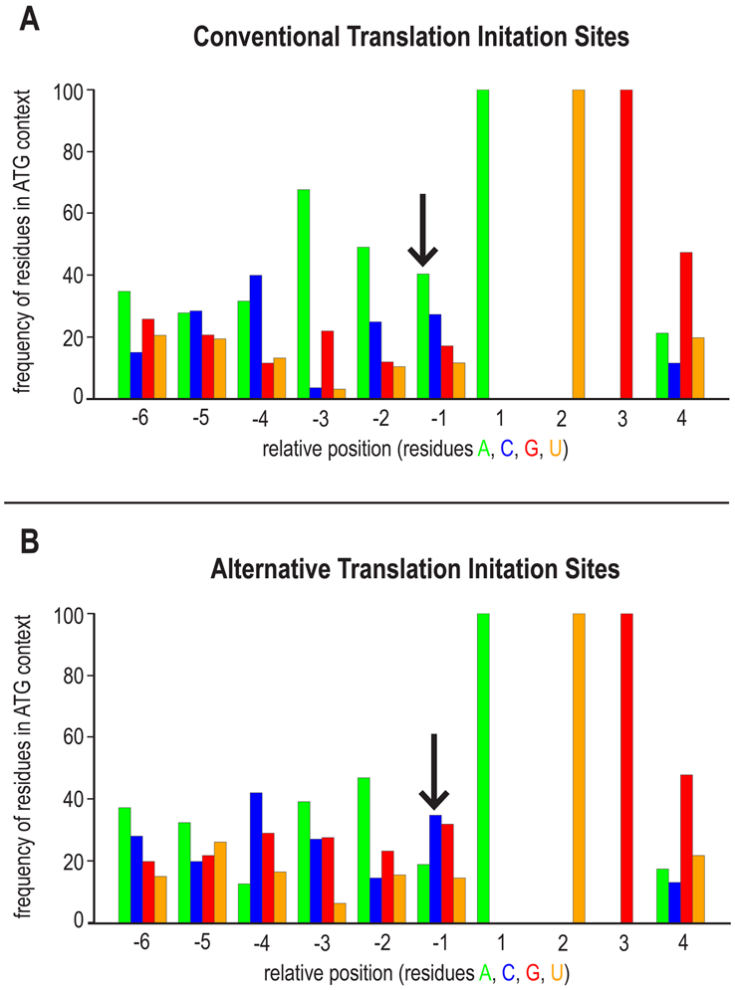
Analysis of translational start sites. (A) A Frequency analysis [54] of all predicted N-termini present in the *Drosophila* database BDGP_Release_3.2 revealed the presence of the Cavener consensus sequence C_A/G_A_A/C_ATG [25],[26]. The histogram shows the relative frequencies of nucleotides 5′ to the predicted (conventional) initiator codon generating acetylated as well as non-acetylated N-termini starting at position 1 or 2 of the predicted protein sequence. The nucleotide 3′ of the initiator sequence has been proposed to preferentially be a G at +4 [55] for strong initiation but has been shown not to be relevant for *Drosophila* [25],[26]. Interestingly the G at +4 is nevertheless predominant in all cases. (B) Relative frequencies of nucleotides 5′ to the alternative translation initiation sites. The Cavener sequence is conserved showing a shift in sequence preference at position −1 (from A to C), which fully complies with the Cavener consensus sequence (C_A_A_C_ATG) [25],[26].

Besides the confirmation of these 1,102 distinct annotated protein N-termini, we expected to find alternative start sites in these two datasets, i.e. peptides with an amino-terminus that starts at position 3 or later of the predicted polypeptide chain and by convention are considered to be internal peptides. However, some of these supposedly internal peptides start with a Met and are semi-tryptic. Others start with a small and uncharged residue, are preceded by a Met in the predicted protein sequence that is missing in the identified peptide, hence indicating an iMet removal as found for a classical protein N-terminus. Our dataset contains 124 distinct peptides that fulfill above criteria (Figure 1B, Table S2) and that we consider to represent alternative translation initiation sites or un-annotated splice variants. To further verify this, we analyzed the sequence context of the AUG that served as putative alternative start codon with respect to its Cavener sequence (C_A/G_A_A/C_AUG; Kozak sequence for insects, the initial Kozak sequence being CC_A/G_C_C AUG_G) [25],[26]. In addition, we analyzed whether the AUG used is the first AUG of that particular exon. A frequency analysis of the residues in the AUG context revealed that the presence of the Cavener sequence could be confirmed for the entire N-terminal dataset (C_A_A_A_AUG; Figure 2A) as well as the putative alternative start sites (C_A_A_C_AUG; Figure 2B). Notably, we detected a change in sequence preference at position −1 (from A to C), which fully complies with the Cavener consensus sequence [25],[26]. It is important to note that these consensus patterns are derived from aggregate frequencies of nucleotides 5′ to the AUG used for translation initiation. If however the Cavener sequence is analyzed for the proposed alternative initiation sites of a single gene model, the Cavener sequences of the used AUG may deviate from the consensus sequence. Cavener and Ray have already recognized this phenomenon and described the consensus as a “strictly statistical term” whereas the optimal context for each individual AUG is defined as a “functional term” [26]. Nevertheless, in about 61% in of the cases the AUG is flanked by an adequate or a strong Cavener sequence, indicating that they represent true alternative start sites (Text S1).

Although essential for genome annotation, computer-based prediction of protein N-termini and alternative translation initiation sites remains a difficult task [27]. In that respect, our dataset not only allowed us to confirm many of the predicted translation sites in the fly but also to identify novel alternative translation initiation sites. In combination, we identified 1,226 amino termini for *Drosophila* Kc (Figure 1C, Table S1), which have been used for all subsequent analyses.

We next analyzed these 1,226 N-termini with respect to N-terminal acetylation (Figure 1C, Table S1). We observed that in the majority of cases (63%) the iMet is removed and that aminopeptidase cleavage follows the same rules as determined for other organisms [28]. About 71% of the N-terminal peptides are acetylated. Of these 61% have the iMet removed, whereas for free N-termini almost 68% showed iMet removal. A comparison of the present data with the respective data from yeast and man [11] shows that N-terminal acetylation occurs in insects with a similar frequency as in humans. Moreover, the acetylation frequency with respect to certain residues seems to have shifted during evolution (Table 1). For instance, whereas most N-acetylated protein-termini in yeast begin with Ser and rarely with Ala, *Drosophila* has a high percentage of acetylated proteins that start with Ser or Ala, whereas Ala is the most commonly acetylated N-terminus in man. Finally, the acetylation state of a protein's N-terminus appears in most cases fixed in *Drosophila* cells as in human HeLa cells but is rare or often incomplete in yeast. Only 57 proteins were identified with both, either a free or an acetylated N-terminus (5% of total in *Drosophila*, 8% in human, and 45% in yeast [11]; Table S3). Thereof, 48 N-termini exhibited the same iMet cleavage and thus had identical amino acid sequences (Figure 1C), whereas nine showed alternative iMet cleavage.

**Table 1:** Frequencies of acetylated and non-acetylated *Drosophila* Kc 167, yeast, and HeLa cell proteins with various N-terminal sequences (position 1 or 2).

	*Fly*								*Yeast*								*Human*							
	Ace	Free	Partial	Total	% Ace	% Free	% Partial	% AceTotal	Ace	Free	Partial	Total	% Ace	% Free	% Partial	% AceTotal	Ace	Free	Partial	Total	% Ace	% Free	% Partial	% AceTotal
*NatA substrates*																								
Ala-	201	21	9	231	87.0	9.1	3.9	90.9	0	23	17	40	0.0	57.5	42.5	42.5	252	11	26	289	87.2	3.8	9.0	96.2
Cys-	6	4	0	10	60.0	40.0	0.0	60.0	0	1	0	1	0.0	100.0	0.0	0.0	1	0	0	1	100.0	0.0	0.0	100.0
Gly-	15	35	1	51	29.4	68.6	2.0	31.4	0	13	0	13	0.0	100.0	0.0	0.0	6	16	5	27	22.2	59.3	19.0	40.7
Ser-	248	19	11	278	89.2	6.8	4.0	93.2	0	3	90	93	0.0	3.2	96.8	96.8	109	1	1	111	98.2	0.9	1.0	99.1
Thr-	44	20	5	69	63.8	29.0	7.2	71.0	0	14	8	22	0.0	63.6	36.4	36.4	21	2	3	26	80.8	7.7	12.0	92.3
Val-	9	47	5	61	14.8	77.0	8.2	23.0	0	22	0	22	0.0	100.0	0.0	0.0	1	25	5	31	3.2	80.6	16.0	19.4
*NatB substrates*																								
Met-Asn-	38	4	1	43	88.4	9.3	2.3	90.7	7	0	0	7	100.0	0.0	0.0	100.0	12	0	2	14	85.7	0.0	14.0	100.0
Met-Asp- & Met-Glu-	206	9	4	219	94.1	4.1	1.8	95.9	18	0	0	18	100.0	0.0	0.0	100.0	124	0	3	127	97.6	0.0	2.0	100.0
*NatC substrates*																								
Met-Ile-	9	6	1	16	56.3	37.5	6.3	62.5	0	2	0	2	0.0	100.0	0.0	0.0	2	1	1	4	50.0	25.0	25.0	75.0
Met-Leu-	17	13	2	32	53.1	40.6	6.3	59.4	1	6	1	8	12.5	75.0	12.5	25.0	4	3	3	10	40.0	30.0	30.0	70.0
Met-Phe	13	6	1	20	65.0	30.0	5.0	70.0	1	1	0	2	50.0	50.0	0.0	50.0	7	2	1	10	70.0	20.0	10.0	80.0
*Other*																								
Asp- & Glu-	0	0	0	0	N.D.	N.D.	N.D.	N.D.	0	0	0	0	N.D.	N.D.	N.D.	N.D.	2	0	0	2	100.0	0.0	0.0	100.0
Met-Gln-	23	3	1	27	85.2	11.1	3.7	88.9	2	1	1	4	50.0	25.0	25.0	75.0	7	1	2	10	70.0	10.0	20.0	90.0
Met-Lys-	3	25	3	31	9.7	80.6	9.7	19.4	0	10	0	10	0.0	100.0	0.0	0.0	2	11	6	19	10.5	57.9	32.0	42.1
Met-Met-	5	1	1	7	71.4	14.3	14.3	85.7	0	0	0	0	N.D.	N.D.	N.D.	N.D.	7	0	0	7	100.0	0.0	0.0	100.0
Met-X*-	24	33	3	60	40.0	55.0	5.0	45.0	2	5	1	8	25.0	62.5	12.5	37.5	8	2	3	13	61.5	15.4	23.1	84.6
Pro-	0	71	0	71	0.0	100.0	0.0	0.0	0	11	0	11	0.0	100.0	0.0	0.0	0	40	0	40	0.0	100.0	0.0	0.0
Ile-	0	0	0	0	N.D.	N.D.	N.D.	N.D.	0	1	0	1	0.0	100.0	0.0	0.0	0	1	0	1	0.0	100.0	0.0	0.0
Total	861	317	48	1226	70.2	25.9	3.9	74.1	31	113	118	262	11.8	43.1	45.0	56.9	565	116	61	742	76.1	15.6	8.2	84.4

Table 1 visualizes the *Drosophila* (fly) dataset according to known NAT substrates in humans and yeast (table adapted from [11]). Ace, acetylated N-termini; free, non acetylated N-termini; X* = A,G,H,P,R,S,T,V,Y.

### Correlation of the N-terminal Acetylation Status with GO Categories and Pfam Domains

To assess whether particular protein functions or functional domains are preferentially associated with the N-terminal acetylation state, a Gene Ontology analysis on a reduced set of GO categories (referred to as GO Slim) on all three levels, namely *Cellular Component*, *Molecular Function*, and *Biological Process*, was performed [29]. The results of this analysis are shown in Table S4 as well as in Figure S2A–S2C. Despite the fact that some categories show a statistically significant (*p*<0.05) over- or underrepresentation of either acetylated or free N-termini, the overall spread of the distributions of acetylated versus non-acetylated gene models does not allow one to make a clear correlation of protein function with a certain GO category or a group of GO categories. Specifically, none of the detected associations with GO categories was strong enough to predict the acetylation state of a protein.

To determine whether proteins that share a specific functional domain also share a common N-terminus (i.e., an acetylated or free amino terminus), a Pfam analysis was performed (see Materials and Methods for details). Pfam is a specialized database that stores protein family classifications and protein domain data and allows one to find relationships between functional domains and any other protein property of interest or classify a so far unknown protein into a protein family [21]. Because N-terminal acetylation is a co-translational process completed after the first part of a protein has been synthesized [12],[13], Pfam domains that start within the first 60 amino acids of a protein were considered. In contrast to the GO analysis presented above, some Pfam domains show a strong association with the acetylation status of certain protein N-termini (Tables 2 and S5A). For example, for the Importin-beta_N-terminal domain (IBN_N, PF03810.11), the N-termini of six out of 15 proteins predicted to contain such a domain have been identified (*p*<0.048). In all six cases the N-terminus was found to be acetylated. The lack of an N-terminal acetylation appears to correlate with a few selected domains comprising for instance the Tubulin/FtsZ_family,_GTPase_domain (PF00091.17, six out of 14 found: *p*<0.00025) or the Ubiquitin_family (PF00240.15, seven out of 20 found).

**Table 2:** Pfam analysis to correlate functional domains with the N-terminal acetylation status of a protein.

Domain Name	Domain Accession	Domain Description	Domains Total	Hits Ace	Hits Free
Acetyltransf_1	PF00583.16	Acetyltransferase_(GNAT)_family	**6**	0	**2**
Actin	PF00022.11	Actin	15	2	7
Aldedh	PF00171.14	Aldehyde_dehydrogenase_family	11	5	1
Aldo_ket_red	PF00248.13	Aldo/keto_reductase_family	*14*	*3*	0
AT_hook	PF02178.11	AT_hook_motif	*7*	*3*	0
OSCP	PF00213.10	ATP_synthase_delta_(OSCP)_subunit	**2**	0	**2**
BolA	PF01722.10	BolA-like_protein	2	2	1
BTB	PF00651.23	BTB/POZ_domain	*99*	*22*	0
Metallophos	PF00149.20	Calcineurin-like_phosphoesterase	22	5	1
CH	PF00307.23	Calponin_homology_(CH)_domain	27	7	2
CoA_binding	PF02629.11	CoA_binding_domain	*2*	*2*	0
Cofilin_ADF	PF00241.12	Cofilin/tropomyosin-type_actin-binding_protein	7	4	2
COG6	PF06419.3	Conserved_oligomeric_complex_COG6	*2*	*2*	0
cwf21	PF08312.4	cwf21	*2*	*2*	0
COX6B	PF02297.9	Cytochrome_oxidase_c_subunit_VIb	*2*	*2*	0
dUTPase	PF00692.11	dUTPase	**2**	0	**2**
Dynamin_N	PF00350.15	Dynamin_family	*4*	*4*	0
efhand	PF00036.24	EF_hand	27	4	3
ENTH	PF01417.12	ENTH_domain	*7*	*4*	0
FERM_N	PF09379.2	FERM_N-terminal_domain	**15**	0	**6**
Glycolytic	PF00274.11	Fructose-bisphosphate_aldolase_class-I	*6*	*5*	0
GSHPx	PF00255.11	Glutathione_peroxidase	3	2	2
GST_N	PF02798.12	Glutathione_S-transferase,_N-terminal_domain	42	11	5
HATPase_c	PF02518.18	Histidine_kinase-,_DNA_gyrase_B-,_and_HSP90-like_ATPase	4	1	2
IBN_N	PF03810.11	Importin-beta_N-terminal_domain	*15*	*6*	0
BIR	PF00653.13	Inhibitor_of_Apoptosis_domain	*3*	*2*	0
Iso_dh	PF00180.12	Isocitrate/isopropylmalate_dehydrogenase	*8*	*4*	0
Kinesin	PF00225.15	Kinesin_motor_domain	*19*	*4*	0
L27	PF02828.8	L27_domain	*2*	*2*	0
LSM	PF01423.14	LSM_domain	16	7	1
Lactamase_B	PF00753.19	Metallo-beta-lactamase_superfamily	7	1	3
MBD	PF01429.11	Methyl-CpG_binding_domain	*4*	*2*	0
Miro	PF08477.5	Miro-like_protein	72	8	2
MIT	PF04212.10	MIT_(microtubule_interacting_and_transport)_domain	*2*	*2*	0
IATP	PF04568.4	Mitochondrial_ATPase_inhibitor,_IATP	3	2	2
Mito_carr	PF00153.19	Mitochondrial_carrier_protein	47	3	3
Myosin_N	PF02736.11	Myosin_N-terminal_SH3-like_domain	**16**	0	**2**
NTF2	PF02136.12	Nuclear_transport_factor_2_(NTF2)_domain	4	3	1
HEAT_PBS	PF03130.8	PBS_lyase_HEAT-like_repeat	**2**	0	**2**
PRA1	PF03208.11	PRA1_family_protein	*2*	*2*	0
Proteasome	PF00227.18	Proteasome_A-type_and_B-type	*23*	*4*	0
Pkinase	PF00069.17	Protein_kinase_domain	*99*	*9*	0
Pkinase_Tyr	PF07714.9	Protein_tyrosine_kinase	*106*	*9*	0
Y_phosphatase	PF00102.19	Protein-tyrosine_phosphatase	5	2	2
Esterase	PF00756.12	Putative_esterase	**2**	0	**2**
PK	PF00224.13	Pyruvate_kinase,_barrel_domain	4	2	2
Ras	PF00071.14	Ras_family	76	8	2
Ribosomal_S6e	PF01092.11	Ribosomal_protein_S6e	3	3	3
Rieske	PF00355.18	Rieske_[2Fe-2S]_domain	*3*	*2*	0
RNA_pol_L	PF01193.16	RNA_polymerase_Rpb3/Rpb11_dimerisation_domain	5	1	3
RRM_1	PF00076.14	RNA_recognition_motif._(a.k.a._RRM,_RBD,_or_RNP_domain)	70	10	3
SelR	PF01641.10	SelR_domain	*5*	*5*	0
Septin	PF00735.10	Septin	*5*	*3*	0
Serpin	PF00079.12	Serpin_(serine_protease_inhibitor)	*30*	*9*	0
adh_short	PF00106.17	short_chain_dehydrogenase	*58*	*5*	0
Cpn60_TCP1	PF00118.16	TCP-1/cpn60_chaperonin_family	14	6	4
TPR_1	PF00515.20	Tetratricopeptide_repeat	11	3	2
Thymosin	PF01290.12	Thymosin_beta-4_family	*4*	*4*	0
Tmemb_18A	PF09771.1	Transmembrane_protein_188	*3*	*2*	0
Tubulin	PF00091.17	Tubulin/FtsZ_family,_GTPase_domain	**14**	0	**6**
TPD52	PF04201.7	Tumour_protein_D52_family	*4*	*3*	0
ubiquitin	PF00240.15	Ubiquitin_family	**20**	0	**7**
Cg6151-P	PF10233.1	Uncharacterized_conserved_protein_CG6151-P	*3*	*3*	0
Vps35	PF03635.9	Vacuolar_protein_sorting-associated_protein_35	2	2	2
V-ATPase_H	PF03224.6	V-ATPase_subunit_H	*3*	*3*	0
VHS	PF00790.11	VHS_domain	4	1	2
WD40	PF00400.24	WD_domain,_G-beta_repeat	42	11	5

Table 2 shows the correlation of Pfam domains starting within the first 60 amino acids of a protein with its N-terminal acetylation status. Pfam domains that were solely associated with an acetylated N-terminus are indicated in italics. Pfam domains that were found to be exclusively associated with a free N-terminus are shown in bold. The *p* values for these correlations are summarized in Table S5A. Ace, acetylated N-termini; free, non-acetylated N-termini.

The functional relevance of any of the correlations we identified in this Pfam analysis is currently unknown. Assuming that the function of a particular domain requires an exclusively acetylated or free N-terminus, two possibilities arise: (i) the acetylation state of the N-terminus is determined by the first amino acid residues of the primary amino acid sequence and/or (ii) the domain contains a so far unknown motif that prevents or promotes acetylation. An example for the first is the association of the IBN_N domain with an acetylated N-terminus: all proteins containing such a domain have amino acids at their N-terminus that promote acetylation. The IBN_N domain itself however starts at position 24 or later, which implies that the conservation of the acetylation state is most likely linked with function. We cannot rule out that unknown motifs within the IBN_N domain exist that support N-terminal acetylation.

For the Tubulin/FtsZ_family,_GTPase_domain and the Ubiquitin_family domain that are linked with a non-acetylated N-terminus, the latter assumption is true: Proteins carrying either domain do not necessarily have amino acid residues that prevent N-terminal acetylation. Other, unknown determinants, likely to reside within the domains themselves, may exist that eventually prevent an acetylation of these protein N-termini.

Our initial Pfam analysis was restricted to domains starting within the first 60 residues of the N-terminal protein sequence. To see whether the correlation of any Pfam domain with the N-terminal acetylation remains, we extended the Pfam analysis to all domains irrespective of their location in the protein (Table S5B). For most of these domains, this association with a free or an exclusively acetylated N-terminus remains.

### Proline Prevents N-terminal Acetylation under all Circumstances: The (X)PX Rule

**Figure 3:**
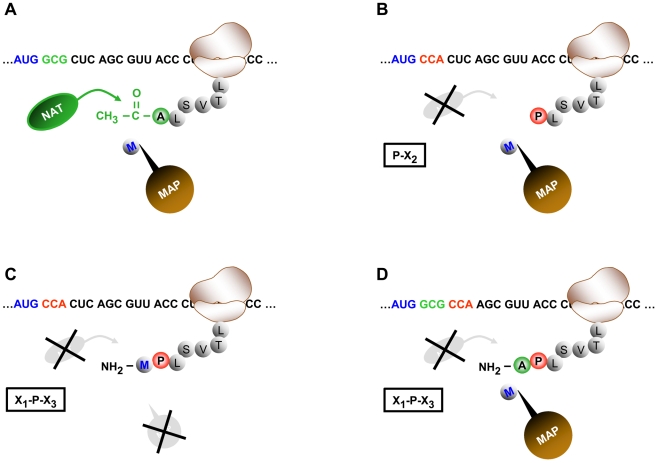
Schematic drawing of the (X)PX rule. (A) During translation of a protein with the sequence Met-*Sur* at its N-terminus (*Sur* being a small and uncharged amino acid residue, in this case *Sur* is equal to an Ala residue in green), the iMet will be removed by a methionine aminopeptidase (MAP, brown bubble) and the processed amino-terminus will be acetylated at the alpha amine of the Ala residue by a NAT (green oval). (B and C) A protein with the sequence Met-Pro at its N-terminus (referred to as Pro-X_2_, panel B) will undergo iMet cleavage by the methionine aminopeptidase (MAP, brown bubble) and the processed amino-terminus will remain unacetylated. If iMet cleavage is not taking place, proteins with the sequence Met-Pro-X_3_ at their mature amino-terminus (panel C) will also remain unacetylated. The Pro residue (red) thus prevents acetylation even if iMet cleavage occurs only partially. (D) Similarly, from a protein having the sequence X_1_-Pro-X_3_ (X_1_ being Ala or Ser, in this case *Sur* is equal to an Ala residue in green, Pro in red), the iMet will be removed and the processed amino-terminus will remain unacetylated (panel D). Although partial removal of the iMet is rarely observed under these circumstances, the N-terminus with the amino acid sequence M-A-P usually remains unacetylated as also observed by SRM measurements.

In the previous section we described the correlation between the N-terminal acetylation state and certain Pfam domains present in a protein. We next asked whether a similar correlation could be detected on the level of the primary amino acid sequence, i.e. within the first two amino acids of a protein's N-terminus. As already shown in Table 1, Ala, Ser, and Thr residues at the mature N-terminus as well as Met-Asp and Met-Glu are acetylated with a high frequency in flies and man (Table 1). On the other hand, proteins with a Val, Gly, or Pro residue at the mature N-terminus or a lysine or arginine as the penultimate residue tend to have a free N-terminus (Tables 1 and S1). This has already been shown for yeast and man [10],[11]. However, it has never been claimed that the presence of one of these amino acid residues (or any other) at the amino terminus is sufficient to unequivocally define a protein's acetylation state. The comparison of our dataset with the ones available for yeast and man (Table 1) clearly shows that a proline residue at the first or second position of the mature protein N-terminus always and in all species analyzed so far prevents amino-terminal modification by the acetylation machinery and thus seems to represent a generic inhibitory signal. Hence, we have formulated a simple rule: a protein with the sequence X_1_-Pro-X_3_ or Pro-X_2_ at its very amino-terminus remains unacetylated under all circumstances (X_1_ being Met or any small amino acid that allows iMet removal by Met aminopeptidases, X_2_ and X_3_ being any amino acid; Figure 3A–3D). This means that proteins, which undergo the partial removal of the initiator Met (iMet) in proteins with the sequence Met-Pro-X_3_, as has been reported by Boissel and others [5], remain not acetylated (Figure 3B, 3C). Similarly, from a protein having the sequence Met-*Sur*-Pro (*Sur* being a small and uncharged amino acid residue), the iMet will be removed and the processed amino-terminus will remain unacetylated (Figure 2D). We would like to emphasize that in this context a Pro at position 2 of the mature amino-terminus overrules N-terminal acetylation even in the presence of promoting amino acids such as Ser or Ala at position 1 (Tables S1). To the above described inhibitory potential of a Pro, we refer to as (X)PX rule.

To unequivocally confirm the (X)PX rule we quantified Pro residue containing protein N-termini by Selective-Reaction-Monitoring (SRM) in total lysates from Kc-cells (for details see Materials and Methods, Figure S3) [30]. SRM enables one to specifically target and quantify peptides of interest in complex mixtures and has shown to be more sensitive and selective than classical tandem-mass spectrometry experiments [31]–[33]. In contrast to conventional mass spectrometry approaches, SRM not only allows for the detection of peptides and peptide modifications but also for their absence. SRM measurements on a set of 17 N-termini following the (X)PX rule were carried out. In our measurements we included the acetylated and non-acetylated form of each peptide and also generated transitions for the iMet either to be cleaved or not (Tables S6 and S7). These targeted SRM measurements revealed that all N-termini are non-acetylated. The acetylated isoforms remained undetectable. Taken together, these findings confirm that a Pro at position 1 or 2 efficiently prevents the acetylation of a protein N-terminus.

### Design of Cell Culture Assays to Challenge the (X)PX Rule

In order to test the (X)PX rule in an in vitro situation we decided to either introduce or replace an inhibitory Pro into selected proteins with a conserved amino terminus and to measure the consequences of these alterations on N-terminal acetylation, similarly to the experiments reported by Boissel and colleagues [5]. First, in our dataset we identified the amino terminal most peptide (Ace-ADPLSLLR) of Hyrax/Parafibromin (Hyx, CG11990) as being acetylated after iMet cleavage. The tumor suppressor Hyx is a component of the Polymerase-Associated Factor 1 (PAF1) complex and has recently been found to be required for nuclear transduction of the Wnt/Wg signal in *Drosophila* [34]. As a second test protein, we chose to investigate the Cyclin-dependent kinase subunit 85A (Cks85A, CG9790) of *Drosophila* [35]. Cks85A has an important role in mitotic progression. The protein follows the (X)PX rule with a proline at position 2 of the primary sequence and the iMet cleaved upon translation (PADQIQYSEK, Table S1). In our datasets, we always found the protein to be non-acetylated.

**Figure 4:**
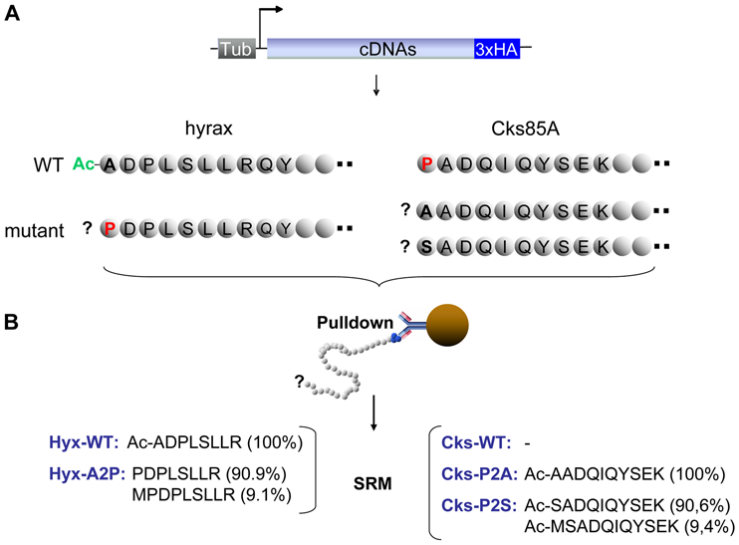
Workflow to test the (X)PX rule. (A) The cDNAs of *hyrax* (*hyx*) and c*ks85A* were modified as follows: in the *Drosophila hyx* cDNA the codon for the secondary Ala was replaced by a Pro to prevent acetylation of the amino-terminus. Similarly, we replaced the codon for the secondary Pro in the *Drosophila cks85A* cDNA by either a Ser or an Ala to promote acetylation. In addition, all constructs were C-terminally HA-tagged and subjected to the control of the tubulin-1α promoter. The different constructs subsequently express Hyx-A2P-HA, Hyx-Wt-HA, Cks-P2A-HA, Cks-P2S-HA, and Cks-Wt-HA, respectively. (B) To test the (X)PX rule in vitro transient transfections of S2 cells were performed. The tagged proteins were isolated via immunoprecipitation and subjected to mass spectrometry analysis via SRM. As expected, the N-terminus of Hyx-Wt-HA was found to be acetylated in combination with a complete iMet removal. In contrast, the amino terminus of Hyx-A2P-HA was always unmodified but showed partial iMet cleavage (9.1%). For both kinase mutants Cks-P2A-HA as well as Cks-P2S-HA the N-Terminus is always acetylated. For Cks-P2S-HA we also detected the acetylated peptide MSADQIQYSEK caused by an incomplete iMet removal (9.4%). Cks-Wt-HA could not be detected due to toxicity effects of the expressed transgene but has initially been isolated via COFRADIC with a free N-terminus.

In order to challenge the (X)PX rule, i.e. to either create or abolish an N-terminal acetylation, the cDNAs of hyrax and Cks85A were modified as follows: (i) in the *Drosophila hyx* cDNA the codon for the secondary Ala was replaced by a Pro (Figure 4A). Both the wild-type (wt) as well as the mutated cDNA were C-terminally HA-tagged, which allowed for the isolation of the respective proteins via immunoprecipitation; (ii) similarly, we replaced the codon for the secondary Pro in the *Drosophila cks85A* cDNA by either a Ser or an Ala. For both amino acids we found a strong promoting effect on N-terminal acetylation (Table 1). All constructs are driven by a ubiquitous tubulin-1α promoter. In the following we will refer to the different constructs as Hyx-A2P-HA, Hyx-Wt-HA, Cks-P2A-HA, Cks-P2S-HA, and Cks-Wt-HA, respectively. To test the (X)PX rule in vitro, *Drosophila* Schneider S2 cells were transiently transfected with one of the above cDNAs. The tagged proteins were isolated via immunoprecipitation trypsinized and the N-terminal peptides were subjected to mass spectrometry analysis via SRM (Figure 4B).

As expected, the N-terminus of Hyx-Wt-HA was found to be acetylated whereas the amino terminus of Hyx-A2P-HA was unmodified. Neither acetylated Hyx-A2P-HA nor unacetylated Hyx-Wt-HA was detectable. In addition, we observed a complete iMet removal from the Hyx-Wt-HA N-terminus and we could detect the unacetylated peptide MPDPLSLLR via SRM, indicating an incomplete iMet removal from the Hyx-A2P-HA isoform (Table S7). Quantitative analysis revealed that the iMet cleavage was omitted for approximately 9% of the proteins (Figure 4B), confirming the results of Boissel and others that have observed iMet retention in 20% of the cases [5]. This observation also demonstrates the inhibitory potential of a Pro residue at the penultimate position of the mature protein N-terminus ((X)PX-rule).

For both kinase mutants, Cks-P2A-HA and Cks-P2S-HA, we found the N-terminus to be acetylated in immunoprecipitation experiments (Table S7). For Cks-P2S-HA (but not Cks-P2A-HA) we also detected the acetylated peptide MSADQIQYSEK indicating an incomplete iMet removal (9.4%). When over-expressing the wt form of the kinase (Cks-Wt-HA) via a tubulin promoter (four attempts in two different cell lines), the cells stopped growing, and the protein could not be successfully measured after immunoprecipitation, neither by LC-MS/MS nor by SRM. This might be due to interference of this form of the protein with a proper cell cycle progression. Wt Cks85A is known to be essential for progression in mitosis [35]. Since the mutated Cks proteins could be well detected, this suggests that an unacetylated N-terminus of Cks seems to be relevant for its proper function.

In conclusion, we could show that the (X)PX rule robustly predicts the acetylation state of proteins in vitro. Furthermore, our experiments show that a single amino acid change is sufficient to explore the function of an N-terminal acetylation of any protein of interest.

### Functional In Vivo Assay for Hyx N-Terminal Acetylation

To test the functional relevance of amino-terminal protein acetylation of Hyx in vivo, transgenic flies were generated using either *tub>a2p-HA* or *tub>wt-HA*, respectively. The hyx transgenes were integrated at the identical, pre-defined chromosomal locus (51D) on the second chromosome, making use of the site-specific phiC31-mediated integration system [36]. Transgene integration at the identical genomic locus guarantees the same protein expression levels. In our experimental context it is important to note that the Ala to Pro exchange in Hyx-A2P-HA alters the translation initiation context at position +4 and thus expression levels might differ between the two transgenes. In *Drosophila*, however, the +4 position has been shown not to be relevant for translation initiation efficiency [25],[26].

**Figure 5:**
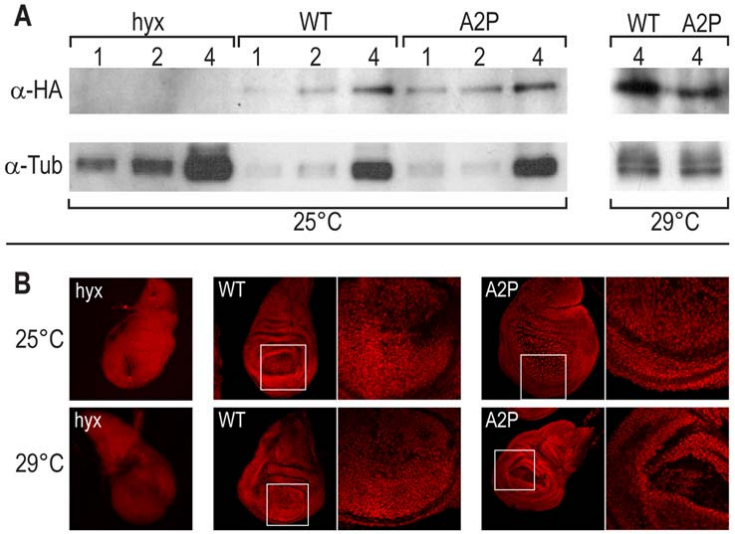
In vivo analysis of genetically modified hyrax transgenes. For an in vivo analysis, the hyrax Hyx-A2P-HA, Hyx-Wt-HA transgenes were integrated into the fly genome using the site-specific phiC31-mediated integration system [36]. Fly genotypes: *tub>hyx-wt-HA/CyO*; *hyx^2^/TM6b* (lanes labeled WT), *tub>hyx-A2P-HA/CyO*; *hyx^2^/TM6b* (lanes labeled A2P), *Sp/CyO*; *hyx^2^/TM6b* (negative controls, lanes labeled *hyx*). Numbers in lanes represent µg of protein loaded (A, B). (A) Total fly lysates of flies reared at 25°C were subjected to Western blot analysis and revealed identical expression levels for the two transgenes (97% Hyx-Wt-HA in comparison to Hyx-A2P-HA). Western blot analysis of total fly lysates of flies reared at 29°C revealed a 32% reduced amount of Hyx-A2P-HA protein as compared to Hyx-Wt-HA controls. (B) Confocal images of stained wing discs from 3^rd^ instar larvae of Sp/CyO; hyx^2^/TM6b (negative controls, opened pinhole), Hyx-Wt-HA/CyO; hyx^2^/TM6b and Hyx-A2P-HA/CyO; hyx^2^/TM6b reared at 25°C and 29°C, respectively. At both temperatures, a similar strong nuclear staining was observed for Hyx-Wt-HA and Hyx-A2P-HA and no difference in localization could be detected.

Flies harboring one copy of either the tub>*wt-HA* or the tub>*a2p-HA* construct rescued the lethality of *hyx* transheterozygous mutants (as had been shown for untagged *hyx* before [34]). To determine the expression levels of the transgenes, tub>*wt-HA/CyO*; *hyx2/TM6B* or the tub>*a2p-HA/CyO*; *hyx2/TM6B* flies (carrying one copy of the transgene and only one endogenous *hyx* wt gene) were collected. Total fly lysates were subjected to Western blot analysis without any prefractionation of the samples and revealed identical expression levels for the transgenes (Figure 5A). This confirms the predictions of Cavener and Ray [26] and due to equal expression levels allowed us to directly compare the biological activity of the two proteins in vivo.

To determine the acetylation state of the respective N-termini, Hyx-Wt-HA and Hyx-A2P-HA were immunoprecipitated from total fly lysates of either of the transgenic lines and analyzed by SRM, whereby transitions specific for (M)ADPLSLLR or (M)PDPLSLLR were measured. These data showed that the N-terminus of Hyx-Wt-HA was exclusively acetylated whereas the amino terminus of Hyx-A2P-HA always remained unmodified as was shown in the in vitro experiments.

To test the relevance of the N-terminal acetylation for the biological activity of Hyx, the transgenes were crossed into a *hyx* homozygous mutant background [34]. Both Hyx-WT-HA as well as Hyx-A2P-HA fully rescued mutant *hyx* allele combinations (hyx^1^/hyx^2^) to adulthood at 18°C as well as 25°C, without obvious phenotypic defects and at the expected Mendelian ratios (Table S8).

However, at 29°C animals carrying a single copy of the Hyx-A2P-HA transgene showed a ∼50% reduction in rescue capability (*p*>6.3521E-06) compared to the Hyx-Wt-HA flies that rescued at the expected rates. To assess whether the observed reduction in rescue capability might be correlated with changes in protein localization, an α-HA antibody staining of 3^rd^ instar wing imaginal discs was performed. Figure 5B shows confocal images of Hyx-Wt-HA and Hyx-A2P-HA of stained wing discs from 3^rd^ instar larvae reared at 25°C and 29°C, respectively. At both temperatures, a similarly strong nuclear staining was observed for Hyx-Wt-HA and Hyx-A2P-HA and no difference in localization could be detected.

To test whether the protein levels have changed at 29°C and might contribute to the difference in rescue capability a Western blot analysis of flies reared at 29°C was performed (same setup as described above). Indeed the Western blot revealed a 32% reduction of Hyx-A2P-HA protein as compared to the Hyx-Wt-HA control (Figure 5A). Therefore, it might be possible that at least in part the reduced protein levels of Hyx-A2P-HA at 29°C are the cause for the lower numbers of rescued flies. Mechanistically, the reduced protein levels may be explained in two ways: (i) one possibility is that protein expression is reduced due to the exchange of the codon for Ala to the one for Pro. This mutation leads to an exchange from G to A at position +4. Although Cavener and colleagues state that the +4 position is not relevant for translation initiation in *Drosophila* [25], we cannot rule out effects on the translation machinery under these conditions (i.e., 29°C). (ii) As a second possibility, protein half life might be affected. It has previously been reported that N-terminal acetylation may contribute to protein stability [3]. If this was the case for Hyx-A2P-HA, we assume that the reduced protein amount in A2P flies reared at 29°C is not due to a change of the N-terminal residue from an Ala to a Pro but rather directly related to the changed modification state of the protein's N-terminus. This hypothesis is supported by the so-called N-end rule, which relates the in vivo half life of a protein to the identity of its N-terminal residue. According to this rule both a Pro and an Ala (as well as Ser, Thr, Gly, Val, and Met) as N-terminal residues confer protein stability in all species [37].

In summary, our experiments show that the (X)PX rule allows one to generate transgenes in order to study the biological relevance of the presence or the absence of an N-terminal acetyl group in vivo.

## Discussion

Although the cellular machinery that is required to acetylate a nascent protein N-terminus is also conserved in invertebrates, it had been suggested that acetylation was a rare event in this animal subphylum since only a few modified N-termini had been reported [10]. In this study we present a comprehensive compilation of mature protein N-termini from *Drosophila melanogaster* Kc 167 cells and show that (i) N-terminal acetylation in fact is also a common protein modification in insects and (ii) follows the same rules (in terms of sequence requirements and iMet cleavage) and (iii) occurs with a lower frequency than in man but at with a much higher frequency than in yeast. Moreover, we observed that the acetylation at the alpha amines of certain residues seem to have shifted in frequency during evolution such that the acetylation of an N-terminus in insects is in some cases in strong agreement with the patterns found in man, while for others it resembles more the distributions and frequencies found in yeast. These differences in acetylation frequency, the partial acetylation of proteins, as well as the lack of comprehensive datasets from different animal kingdoms may explain why prediction of the acetylation state from a primary protein sequence is still not yet clear-cut [11].

In fact, for some N-termini we still lack the understanding of what determines their acetylation state (especially for Gly, Val, or Thr as the first or second residue in the mature protein). Also some proteins carrying an acetylation promoting Ser and Ala at position 2 remain unacetylated. In *Drosophila*, 30 of a total of 289 identified N-termini that expose a Ser residue at their mature N-terminus are not acetylated (11 of which are only partially acetylated, i.e. appear acetylated and non-acetylated after iMet cleavage) (Table S9). In two cases, an inhibitory Pro residue fulfilling the (X)PX rule prevents the acetylation in spite of a Ser in the first position. One protein CG2679-PB, for which we have defined a putative alternative start site (putative alternative iMet at position 36 in the predicted sequence; Text S1, Table S2), has a signal sequence (SignalP 3.0, http://www.cbs.dtu.dk/services/SignalP/; [38]) predicted to be cleaved between residue 25 and 26. For the remainder (as well as for the Val, Gly, and Thr) the only plausible explanation is that there must be other features within the first 50 amino acids that prevent N-terminal acetylation during protein synthesis. Polevoda and Sherman suggested that the secondary structure of a protein's N-terminus may play a role [10]. We have analyzed the predicted secondary structure of all identified proteins (residues 1–50, unpublished data) but could not identify any correlation of structure and acetylation state. Consequently the expected determinants (such as complex sequence patterns within the first amino acids) remain to be identified through sophisticated computational analysis for which this dataset may act as an ideal starting point.

If no additional determinants (such as patterns or structural motifs) exist steric hindrance could be responsible for the preservation of a free N-terminus despite the presence of promoting amino acid sequences. During protein synthesis, cofactors like chaperones could bind to the nascent polypeptide chain and prevent proper function of the acetylation machinery: for instance it is known that tubulins undergo a sequence of folding steps catalyzed by chaperones, which are commonly assumed to take place after translation is completed [39],[40]. Our data show that tubulins always appear to have a free N-terminus despite the occasional presence of amino acids that promote its acetylation. In this context it may therefore well be that chaperones could already associate with the transcription machinery, thereby preventing the acetylation of tubulin by steric hindrance.

We have observed that *in Drosophila* the acetylation is fixed in most cases. This is similar to the case in human and in contrast to yeast where partial acetylation is common [11]. Possible explanations for this discrepancy between the organisms could be that yeast cells (but not fly or human cells) grow too fast, making acetyl-CoA poorly available as substrate, or the degradation of the unacetylated proteins cannot cope with division rates and thus the proteins with a free N-terminus accumulate in the cells. Nevertheless, in certain cases a partial acetylation of proteins may be of relevance for yeast as well as for higher eukaryotes. To asses this relevance such proteins could be genetically engineered according to the (X)PX rule, shifting the equilibrium of the mutant proteins towards a fixed acetylated or free N-terminus.

N-terminal acetylation has been shown to be absolutely necessary for the proper localization, activity, or stability of various proteins. The current view is that the presence rather than the absence of an acetylation is important for protein function. Our Pfam analysis challenges this view as it also shows a clear correlation for a non-acetylated N-terminus and certain protein domains. Additional evidence for the perspective that the absence of an acetylation is also important for proper protein function has been demonstrated in the case of human hemoglobin. The exchange of Val to Ala at the second position in the human hemoglobin beta chain reverts the usually free N-terminus into an acetylated one. Patients carrying this so-called “Raleigh” mutation suffer from thalassaemia due to a reduced affinity of the mutant hemoglobin to oxygen [41], underlining the importance of the free amino terminus for proper function. Finally, our own transgene analysis on Cks85A strongly indicates that the preservation of the protein's non-acetylated N-terminus is relevant for its proper function. In this context it is important to note that no pattern exists that guarantees an N-terminal acetylation, although Ser and Ala as second residues usually ascertain a modification. For the prevention of acetylation the opposite is true: based on the data we formulated the (X)PX rule that states that a Pro residue at the primary or secondary position of the mature protein N-terminus prevents acetylation and assures a free amino terminus under all circumstances. Since the majority of protein N-termini are acetylated in eukaryotic organisms investigated so far, and due to the presence of an absolute inhibitory rather than a promoting signal, we conclude that the acetylation of protein N-termini is the default state and, where necessary, is prevented by specific motifs. Along this line it has been suggested that some N-termini are acetylated just because of the presence of a promoting pattern but that acetylation has no functional relevance [3]. One can easily imagine such a case: a Ser at position 2 in the primary sequence has been evolutionary conserved because its phosphorylation is relevant for its biological activity. Upon protein synthesis this Ser by default also promotes iMet cleavage and the subsequent acetylation of the N-terminus. The conservation of the Ser, however, occurred due to the need for Ser phosphorylation and not N-terminal acetylation. In such a scenario one could apply the (X)PX rule to discriminate whether acetylation and phosphorylation or phosphorylation alone would contribute to protein function: the introduction of an inhibitory Pro after the Ser residue would allow iMet cleavage as well as the phosphorylation of the Ser but would prevent the acetylation of the N-terminus. Likewise, the Ser to Ala (or comparable) amino-acid exchange would preserve the acetylation (at least in insects and human) but not the phosphorylation of the protein, allowing one to assess the contribution of the N-terminal acetylation to protein function. A relevant example would be the rat RNA polymerase subunit 6 (RPB6), which is phosphorylated by Casein KinaseII (CkII) at a Ser at residue 2. It has, however, not been reported whether the acetylation state of this protein is relevant for its function [42].

Unlike a phosphorylation, which is “dynamic” and may be rapidly added or removed from a protein upon stimulation of a cell, N-terminal acetylation has been shown to be a co-translational, irreversible, and thus a “static” modification [3]. However, we and others have evidence that acetylation of N-termini does not only occur co-translationally [14]: we have identified 511 internal, acetylated peptides that reside within the annotated protein sequence (Table S10). These N-termini do not comply with the suggested N-terminal acetylation rules and thus are suggested to occur post-translationally. They require a to-date unknown acetylase activity as well as internal proteolytic cleavage at a specific site of the protein. It is tempting to speculate that these post-translational acetylation events may also occur in a dynamic fashion, allowing fast responses to various stimuli.

It remains to be noted that acetylation of N-terminal Pro residues has been reported in yeast [43]–[45]. The experimental evidence for an N-terminally acetylated Pro residue is solely based on shifts of protein spots observed in comparative 2D gel experiments of NAT-mutant versus wt strains. A direct measurement of acetylation has not been performed in this context [45]. In contrast, in two large-scale studies N-terminal Pro acetylation has only been observed if an internal Pro residue is exposed at the N-terminus after post-translational processing of the protein (see Tables S1 and S10). From this we conclude that the (X)-P-X rule, which is formulated for the first three residues of the primary amino acid sequence, is robust and that possible exceptions to this rule will be extremely rare and most likely attached post-translationally by a yet undescribed protein activity.

Although amino-terminal acetylation has been studied for more than 30 years, some aspects, foremost the relevance of its presence or absence for individual protein function, remain unclear for most of the cases. With this work we have defined tools that will help to better understand the elusive mechanisms as well as to explore the function of protein N-terminal acetylation in all organisms in a protein-specific manner. Complementary studies using for instance mutations in the relevant NAT enzymes will help to eventually draw firm conclusions about the function of the N-terminal acetylation.

## Materials and Methods

### Construction of Vectors

To generate expression vectors for the hyrax and Cks85A transgenes, the respective DNAs were amplified by PCR. The forward primers contained a 5′ Kozak consensus (CGCCACC) and the appropriate base exchanges to generate the desired wt or mutant forms.

1. hyx_WT_fw: AAAGGTACCGCCACCATGGCAGATCCGCTCAGCCTGC;
2. hyx_A2P_fw: AAAGGTACCGCCACCATGCCAGATCCGCTCAGCCTGC;
3. hyx_rev: AAAGGCGCGCCCCATATCGTAAATCGGGCTTGTACTTGG;
4. Cks85A_WT_fw: AAAGGTACCGCCACCATGCCGGCCGATCAAATTCAATAC;
5. Cks85A_P2A_fw: AAAGGTACCGCCACCATGGCGGCCGATCAAATTCAATAC;
6. Cks85A_P2S_fw: AAAGGTACCGCCACCATGTCGGCCGATCAAATTCAATAC;
7. Cks85A_rev: AAAAAGCGCGCACCGCAGAGATTCGCGATGGC.

PCR fragments were inserted into the *pOP-118* vector to create cDNAs driven by a ubiquitous tubulin-1*α* promoter and tagged with a 3xHA. For in vivo experiments the expression cassettes were cloned into an attB integration backbone [36].

### Cell Culture and Immunoprecipitation Experiments

*Drosophila melanogaster* Kc 167 and S2 cells were cultivated in Schneider's *Drosophila* medium (Gibco/Invitrogen) supplemented with 10% heat-inactivated fetal-calf serum, penicillin (100 U/ml), and streptomycin (100 µg/ml) at 25°C. Cells were split 1∶4 (v/v) every 3–4 d when they reached confluency.

For immunoprecipitations 75 cm^2^ flasks of S2 cells were transfected with 2 µg of the respective tub-[cDNA]-HA plasmid via Effectene Transfection Reagent (Qiagen). Cells were collected 48 h after transfection and lysed with lysis buffer (20 mM sodium phosphate-buffer, 200 mM NaCl, 0.5% NP40, supplemented just before use with 1 mM DTT, and Complete™ protease inhibitor cocktail (Roche)). Cell lysates were incubated for 3 h with monoclonal anti-HA agarose beads (clone HA-7, Sigma). Finally, beads were washed three times with lysis buffer, twice with lysis buffer without detergent, and bound proteins were eluted with 0.2 M glycine (pH 2.5).

### Fly Stocks

For in vivo assays, the following tester lines were used: transgenic vectors were injected into *Drosophila* (yw) that contained the second-chromosomal attP landing site at map position 51D [36]. Potential founders were individually outcrossed against the balancer stock *yw*, *hs-flp*; *Sp/CyO*; *TM6b/MRKS*. Their *white+* progeny was then individually backcrossed to the balancer background to establish clonal stocks. The resulting founder stocks were verified for the correct transgene by PCR. Successfully established transgenic insertions were then crossed into *hyx* mutant backgrounds, as described previously [34].

### Immunohistochemistry

Wing discs were isolated, fixed, and incubated as previously described [46]. Antibodies used for the staining were mouse HA11 (1∶1000, BAbCO) and a goat secondary antibody coupled to Alexa^594^ (1∶500, Molecular Probes).

### Western Blot Analysis

For total protein isolation adult flies were grinded in liquid nitrogen and the homogenate was resuspended in a 4 times volume of lysis buffer (20 mM TrisHCl pH 7.5, 150 mM NaCl, 0.2% NP40, 10% glycerol supplemented just before use with 1 mM DTT, and Complete™ protease inhibitor cocktail (Roche)). Protein samples were run on a 10% SDS-PAGE at 140 V for 90 min and subsequently transferred to a Hybond-P PVDF Membrane (Amersham). After the transfer, the membrane was rinsed in PBST, blocked with 5% nonfat dried milk in PBST and then incubated with either monoclonal mouse anti-alpha tubulin DM1A (1∶2000, Sigma) or mouse anti-HA.11 (1∶1000, BAbCO). As secondary antibody, peroxidase-conjugated goat anti-mouse IgG (1∶10000, Jackson) was applied. Signals were detected via ECL Plus (Amersham). Intensities of bands were quantified using the histogram option (average gray value) of ImageJ (NIH).

### Sample Preparation for Mass Spectrometry

Total protein lysates or proteins isolated via immunoprecipitation were reduced with 5 mM Tris(2-carboxyethyl)phosphine hydrochloride (TCEP) and treated with 10 mM iodoacetamide to modify cysteine residues. Tryptic digestion was carried out overnight using 5 µg trypsin per sample. Samples were purified by reverse phase C-18 chromatography (Sep-PacK, Waters). For mass spectrometry analysis samples were resuspended in buffer A (5% acetonitrile, 0.2% formic acid).

### SRM

SRM was performed on a triple quadrupole mass spectrometer (TSQ Quantum Ultra EMR, Thermo Fisher Scientific) operated with Xcalibur 2.0.7 (Thermo Fisher Scientific). The instrument was coupled to an Eksigent nano-LC system. Samples were automatically injected into a 10-µl sample loop and loaded onto an analytical column (9 cm length×75 µm (internal diameter) packed in-house with Magic C18 AQ beads 5 µm, 100 Å (Microm)). Peptide mixtures were delivered to the analytical column at a flow rate of 500 nl/min of buffer A (5% acetonitrile, 0.2% formic acid) for 18 min and then eluted using a gradient of acetonitrile (10%–35%; 0.36%/min) in 0.2% formic acid at a flow rate of 250 nl/min.

SRM measurements were carried out with a Q1 resolution of 0.4 and a Q3 resolution of 0.7 *m*/*z* half-maximum peak width. Scan speed was set to 0.020 ms per scan event. SRM transitions specific for proteotypic peptides were generated using the SRM Workflow (software from Thermo Fisher Scientific). For each precursor transitions were calculated with precursor charges 2+ or 2+ and 3+. At least four y-ions with *m*/*z*> precursor were monitored. Collision energies (CE) were calculated according to the following formulas: CE = 0.034×*m*/*z*+3.314 (2+) and CE = 0.044×*m*/*z*+3.314 (3+). SRM traces were evaluated via the SRM Workflow. For each peptide the co-elution of all transitions was confirmed. Peptides with non-coeluting elution profiles or bad resolution or signal to noise ratios were not considered for further analysis. iMet cleavage was quantified by comparing the ion current profile of identical y-product ions.

SRM-triggered MS/MS experiments were performed to validate the identity of the peptides for the relevant SRM traces. MS/MS spectra were acquired with a Q3 resolution at 0.7 *m*/*z* half-maximum peak width. The scan range was automatically determined by the instrument using the assigned charge state of the precursor ion (2+). MS/MS spectra were assigned to peptide sequences using the Mascot software version 2.2 (Matrix Science).

### Processing of Shotgun Data

The data for Kc167 cells from the previously released proteome catalog for *Drosophila melanogaster* (retrieved from [24]) were searched using the Mascot search algorithm for finding N-terminal peptides. The search criteria were set as follows: Variable modifications were set to Acetyl (N-term), Deamidation (NQ), Carbamidomethyl (C, or where required ICAT-C, ICAT-C:13C(9)), Oxidation (M), Mass values monoisotopic, Peptide mass tolerance 3 Da, Fragment mass tolerance ±0.8 Da, Maximum missed cleavages 2, Instrument type Esi Trap. All peptide assignments where the Mascot ion score is greater than the homology score and where the Mascot expect score is smaller than 0.05 were considered as good hits for further analysis.

### Post-Processing of Searched Data

All peptides called as present by Mascot in the SAX, SCX, and the published datasets [47] were remapped against the Berkeley *Drosophila melanogaster* protein database (BDGP) release 3.2 for the purpose of extracting all the possible splice variants and alternative proteins belonging to a specific peptide. Furthermore, the proteins obtained in this way were then classified according to their information content into five different classes [48]. This information content considers the relationship between the protein sequences and gene models and minimizes protein inference errors due to ambiguous protein sequences.

The N-termini were extracted from the overall list of peptides. Two types of N-termini are distinguished: (i) N-termini that start in position 1 or 2 (after methionine cleavage) in at least one protein of all the possible alternatives for the respective peptide and (ii) potential alternative methionine starting sites, i.e. semi-tryptic peptides that start with methionine, or where the precedent amino acid is a methionine. This list was manually cured, excluding fully tryptic peptides that likely represent internal peptides (the peptides identified in the COFRADIC approach may be preceded by a Lys residue since they are modified and thus no longer cleaved by trypsin). Furthermore, possible redundancies with respect to N-terminus were reduced, i.e. cases where one N-terminus is a prefix of another N-terminus with the exception of partially acetylated N-termini. The entire list of N-termini is presented in Table S1.

### Extraction of Kozak/Cavener Context

Peptide sequences were mapped onto the genomic sequence in order to extract the DNA context information of the iMet (or of the starting ATG triplet). For this purpose, for each gene model of BDGP3.2 the protein coding sequence, the respective transcript, as well as the exon containing the potential alternative iMet were extracted and the Cavener and Kozak context was analyzed. A sequence (−4 to −1) for which up to 1 nucleotide was identical to the Cavener or Kozak consensus sequence was labeled as weak, 2–3 perfect nucleotide matches were labeled as adequate, and identical sequences were labeled as strong (see Text S1).

Weblogos were created using the library weblogo (http://code.google.com/p/weblogo/) in Python in order to represent the relative frequency and information content of different nucleotides at varying positions around the Kozak/Cavener sequence and bar plots were created using R (www.r-project.org).

### Pfam Analysis

Pfam (www.sanger.ac.uk/Software/Pfam/) was used to identify functional domains in the experimentally identified protein sequences. For this purpose, the set of 16,743 distinct protein sequences of BDGP3.2 were searched against the Pfam database of Hidden Markov Models (release July 23, 2008, 10,340 protein family models) using the HMMER software package (hmmer2.3.2, http://hmmer.janelia.org/).

The Pfam results were compared then with the results of acetylated seen protein sequences for any over- or underrepresentation and Fisher's exact test with multiple testing correction was performed for selected Pfam categories.

### Gene Ontology Analysis

To assess any possible association between the acetylation status of a group of proteins and different GO terms, all possible gene models were extracted from Table 1 and were submitted to a GO Slim analysis (http://go.princeton.edu/cgi-bin/GOTermMapper). This tool uses the map2slim.pl script (Chris Mungall, BDGP) to bin the submitted gene lists to a static set of broader, high-level parent generic GO-slim terms.

The GO analysis was performed for “Biological Process,” “Cellular Component,” and “Molecular Function,” and Fisher's exact test, with multiple testing correction, was performed for each GO term to provide a *p* value for the respective over- or underrepresentation.

### SAX Peptide Fractionation Prior to N-Terminal COFRADIC Analysis

8 mg of the Kc167 cell cytosolic fraction was first dissolved in 1 ml of 6 M guanidinium hydrochloride in 50 mM phosphate buffer (pH 7.5), after which the guanidinium hydrochloride concentration was lowered to 4 M by adding 0.5 ml of 50 mM phosphate buffer (pH 7.5). Proteins were reduced and alkylated by adding TCEP and iodoacetamide to final concentrations of 50 mM and 100 mM, respectively (reaction was allowed for 60 min at 30°C). Then, excess reagents were removed by desalting the protein mixture over a 10 ml Zeba Spin Desalting Column (Pierce, Thermo Fisher Scientific, Erembodegem, Belgium). Proteins were collected in 1 ml of 2 M guanidinium hydrochloride in 50 mM phosphate buffer (pH 8). Free primary amines (protein α-N-termini and ε-amines of lysines) were acetylated using an N-hydroxysuccinimide ester of trideutero-acetate (prepared according to [49]). Here, 5.8 mg (about 36 µmol) of this NHS-ester was dissolved in 20 µl DMSO and then added to the protein sample. Acetylation was allowed for 90 min at 30°C, after which O-acetylation of Ser, Thr, and Tyr side-chains was reversed by adding 10 µl of 50% of hydroxylamine to the protein solution and incubation for 20 min at room temperature.

This modified protein mixture was desalted as indicated above, but now eluted in 50 mM of NH_4_HCO_3_ (pH 8, freshly prepared). The protein mixture was boiled for 5 min, placed on ice for another 5 min, and then overnight digested by adding 40 µg of sequencing-grade modified trypsin (Promega Corporation, Madison, WI, USA). Small particulate matter was removed by centrifugation for 10 min at 10,000 g. Half of the digest (500 µl) was loaded on a strong anion exchange (SAX) column (SAX-Zirchrom 2.1 mm I.D.×150 mm length; 3 µm particles, ZirChrom Separations, Anoka, MN, USA) after which a binary solvent gradient was applied to fractionate peptides. Solvent A consisted of 10 mM Tris-HCl (pH 8) in 25% acetonitrile and solvent B was 1 M NaCl in 10 mM Tris-HCl (pH 8) and 25% acetonitrile. Under a constant flow of 70 µl/min, the following gradient was applied: 0% solvent B (0–50 min), 10% solvent B (55 min), 50% solvent B (100 min), washing with 50% solvent B until 105 min, 100% solvent B (107 min), followed by a 5 min wash with solvent B, 100% solvent A (114 min), and re-equilibration with solvent A (170 min). SAX-separated peptides were collected in 2 min wide fractions between 10 and 130 min and were finally pooled into six fractions in such a way that each fraction contained similar amounts of total peptide material (judged from the UV absorbance trace).

### SCX-Based Enrichment of N-Terminal Peptides

It was previously demonstrated that strong cation exchange (SCX) chromatography at acidic pH can be used to enrich N-terminal peptides from whole proteome tryptic digests [14],[22]. These peptides are in fact less well retained by the SCX column as other peptides and are thereby segregated from the bulk of non-N-terminal peptides. One disadvantage, however, is that N-terminal peptides containing histidine will still be retained by the SCX column and are thus largely absent from the final lists of reported identifications [22].

Here, SCX was used to enrich N-terminal peptides from digests of the cytoplasmic, nuclear, and membrane fraction of Kc 167 cells prior to COFRADIC. Full experimental details can be found in [22], and in brief, protein preparations were first reduced and S-alkylated with iodoacetamide, then all free primary amines were blocked by trideutero-acetylation, and finally proteins were digested into peptides using trypsin. This peptide mixture was then applied on a SCX column at pH 3 and blocked N-terminal peptides were found enriched in the non-binding fraction. These peptides were then further enriched by N-terminal COFRADIC (see below).

### Isolation of N-Terminal Peptides by COFRADIC, LC-MS/MS Analysis, and MASCOT Analysis

All six SAX fractions and the SCX-enriched proteome digests of the Kc167 cell cytosolic, microsomal, and nuclear fractions were then further analyzed by the N-terminal COFRADIC technology [20] as described in [22]. Such isolated peptides were then further analyzed by LC-MS/MS analysis on either a Bruker Esquire HCT ion trap (SAX peptides, ion trap operated as described in [50]) or an LTQ linear iontrap operated as described in [47] or an Agilent XCT-Ultra IT mass spectrometer equipped with Agilent's Chip Cube (SCX peptides, ion trap operated as described in [51]). Following analysis, raw MS/MS-spectra were converted to mgf files, which were used to identify the corresponding peptides using the MASCOT database search tool [52], in-house installed. The following parameters were set. Carbamidomethylation of Cys and trideutero-acetylation of Lys were set as fixed modifications. Acetylation or trideutero-acetylation of peptide N-termini, carbamylation of Lys (only for SAX-separated peptides), deamidation of Asn and Gln, oxidation of Met (sulfoxide), N-terminal pyrocarbamidomethyl Cys, and N-terminal pyroglutamic acid were all set as variable modifications. The mass tolerances for both the precursor ion and fragment ions were set to ±0.5 Da. The enzyme was set at endoproteinase Arg-C and cleavage between Arg-Pro and one missed cleavage were allowed. MASCOT's instrument setting was ESI-TRAP. The BDGP 3.2 database was searched as well truncated versions of this database that anticipate for protein processing events and were previously found to increase the number of identifications. These truncated versions were made using the DBToolkit algorithm [53]. The raw DAT result files of Mascot were then queried using in-house developed software tools. Only MS/MS-spectra receiving a score exceeding Mascot's identity threshold score at the 95% confidence level were kept. Such identified peptides were then automatically stored in a MySQL relational database (see http://genesis.ugent.be/ms_lims/) in which links were made to their MS/MS-spectra and precursor proteins.

## Supporting Information

Figure S1 **Calculation of theoretically visible N-termini.** (A–B) In theory, 9,319 (56%) of the very N-terminal tryptic peptides of the 16,743 distinct *Drosophila* proteins are within the detectable dynamic range of current mass spectrometers in a regular trypsin digest (A). For calculation the following constraints have been applied: the peptides have to be fully tryptic without missed cleavages, are at least six amino acids long (green lines), and their precursor m/z value is within the range 450–1,500 (considering the charge states 1+ to 3+). Interestingly, the N-terminal tryptic peptides of close to 7,000 distinct *Drosophila* proteins are six or less amino acids in length (blue lines and below 450 m/z and thus not detectable). Using COFRADIC (B), where lysines are blocked and no longer available for trypsin cleavage, the number of detectable N-terminal tryptic peptides rises to 10,373 (62%). However, since some of these N-terminal peptides might be shared between different proteins, the number of proteins distinguishable based solely on their N-terminal peptide drops to 8,347 for a regular digest (Figure S1A, 50% of all annotated proteins), and to 9,389 for the COFRADIC treatment (Figure S1B, 56.1%). Finally, if we also consider proteins with a predicted signal peptide (SignalP 3.0 [38], the number of detectable proteins is further reduced, and in theory, we could identify 7430 (A) or 8212 proteins (B), respectively).
(0.23 MB TIF)

Figure S2 **Analysis to correlate GO categories with the N-terminal acetylation status.** (A–C) To assess whether particular protein functions or functional domains are associated with the N-terminal acetylation state, a Gene Ontology analysis on a reduced set of GO categories (referred to as GO Slim) on all three levels, namely *Cellular Component* (A), *Molecular Function* (B), and *Biological Process* (C) was performed. The results of this analysis (representative examples with at least two members seen in a category) are also shown in Table S4. For more details please see Text S1. * statistically significant different frequency observed (*p*<0.05).
(1.09 MB TIF)

Figure S3 **The principle of SRM in a triple quadrupole mass spectrometer**. SRM is performed in a specific type of mass spectrometer, which consists of a linear series of three mass analyzers, so-called quadrupoles. The first quadrupole (Q1) functions as a mass filter, capable of selectively transmitting only the parent ion (peptide) of interest from a complex peptide mixture. The second quadrupole (Q2) serves as a fragmentation unit, in which the selected peptide is fragmented into smaller product ions by collisionally activated dissociation. Finally, a single fragment (product ion) that is characteristic for a peptide is passed through to the third mass analyzer Q3 and can be quantified.
(0.08 MB TIF)

Table S1 **Compilation of all N-terminal peptides identified in this study.**
(0.28 MB XLS)

Table S2 **Alternative start sites.**
(0.04 MB XLS)

Table S3 **Ambiguously acetylated N-termini.**
(0.03 MB XLS)

Table S4 **(A–C) Statistics of the GO Slim analysis.**
(0.03 MB XLS)

Table S5 **(A–B) PFAM analysis for associating functional domains with the N-terminal acetylation status of a protein.**
(0.05 MB XLS)

Table S6 **Confirmation of the XPX and PX rule by targeted SRM.**
(0.03 MB XLS)

Table S7 **Targeted SRM measurements of hyrax and Cks85A (pulldown experiments).**
(0.02 MB XLS)

Table S8 **Rescue experiments with Hyx-Wt-HA and Hyx-A2P-HA.**
(0.02 MB XLS)

Table S9 **Non-acetylated N-termini with a serine residue at the mature N-terminus.**
(0.02 MB XLS)

Table S10 **Acetylated internal peptides. Their N-termini do not comply with the suggested N-terminal acetylation rules.**
(0.11 MB XLS)

Text S1 **Kozak/Cavener analysis of putative alternative start sites, and GO Slim analysis.**
(0.50 MB DOC)

## Abbreviations

BDGP: Berkeley *Drosophila* Genome Project
CE: collision energy
Cks85A: Cyclin-dependent kinase subunit 85A (CG9790)
COFRADIC: Combined Fractional Diagonal Chromatography
GO: Gene Onthology
HA: hemagglutinin
Hyx: Hyrax protein (CG11990)
i-Met: initiator methionine
MAP: Methionine aminopeptidase
NAT: N-terminal Acetyl Transferase
Pfam: Protein Families Database of Alignments and Hidden Markov Models
SAX: strong anion exchange
SCX: strong cation exchange
SRM: Selective-Reaction-Monitoring
TCEP: Tris(2-carboxyethyl)phosphine hydrochloride
wt: wild-type
