# Supplementary figures and images for: Identification and Functional Characterization of N-Terminally Acetylated Proteins in Drosophila melanogaster
Source: PLoS Biol. 2009 Nov 3;7(11):e1000236. doi: 10.1371/journal.pbio.1000236 (PMC2762599; doi:10.1371/journal.pbio.1000236)

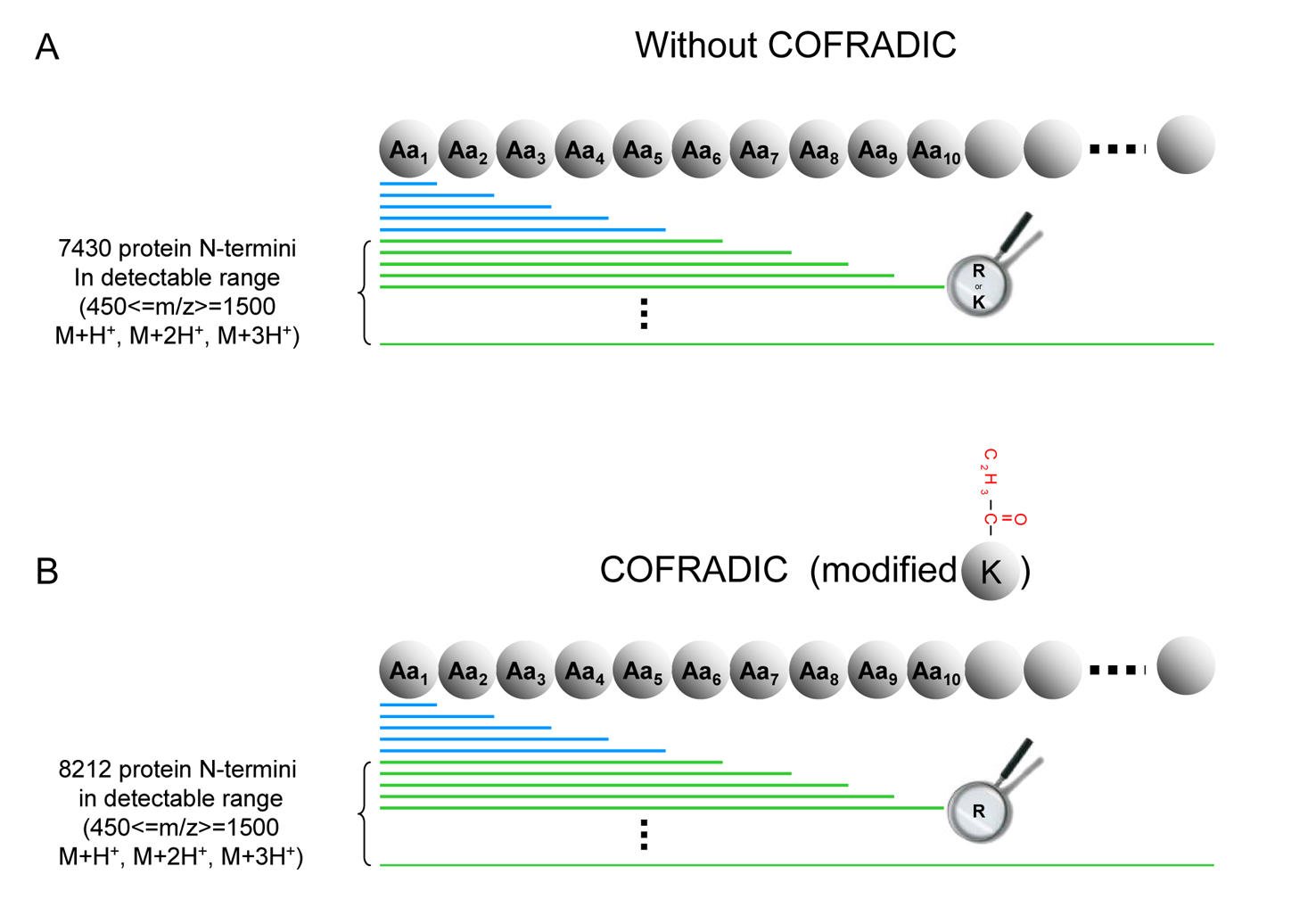

Supplement: Figure S1 — Calculation of theoretically visible N-termini. (A–B) In theory, 9,319 (56%) of the very N-terminal tryptic peptides of the 16,743 distinct Drosophila proteins are within the detectable dynamic range of current mass spectrometers in a regular trypsin digest (A). For calculation the following constraints have been applied: the peptides have to be fully tryptic without missed cleavages, are at least six amino acids long (green lines), and their precursor m/z value is within the range 450–1,500 (considering the charge states 1+ to 3+). Interestingly, the N-terminal tryptic peptides of close to 7,000 distinct Drosophila proteins are six or less amino acids in length (blue lines and below 450 m/z and thus not detectable). Using COFRADIC (B), where lysines are blocked and no longer available for trypsin cleavage, the number of detectable N-terminal tryptic peptides rises to 10,373 (62%). However, since some of these N-terminal peptides might be shared between different proteins, the number of proteins distinguishable based solely on their N-terminal peptide drops to 8,347 for a regular digest (Figure S1A, 50% of all annotated proteins), and to 9,389 for the COFRADIC treatment (Figure S1B, 56.1%). Finally, if we also consider proteins with a predicted signal peptide (SignalP 3.0 [38], the number of detectable proteins is further reduced, and in theory, we could identify 7430 (A) or 8212 proteins (B), respectively). (0.23 MB TIF) [file pbio.1000236.s001.tif]

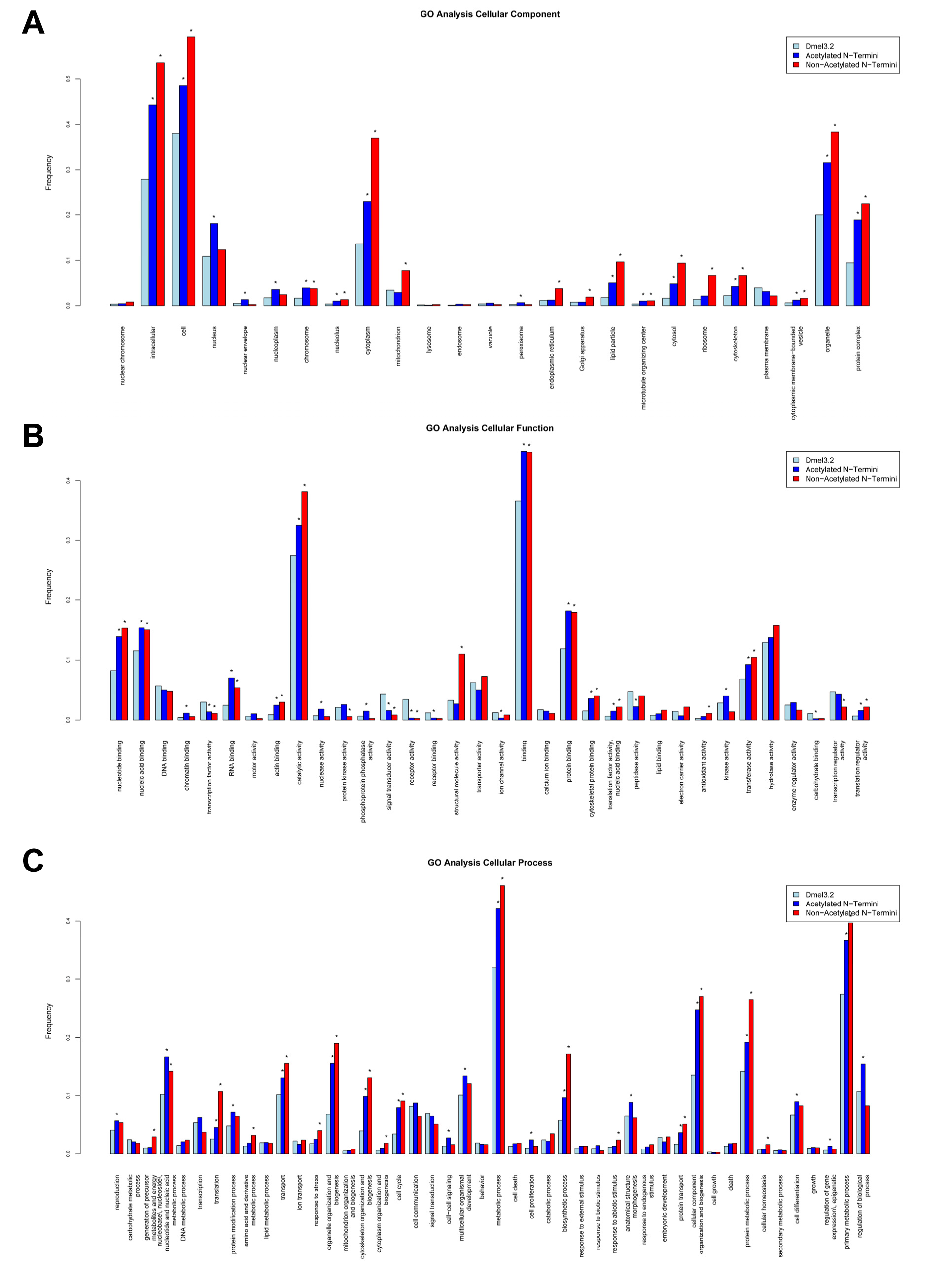

Supplement: Figure S2 — Analysis to correlate GO categories with the N-terminal acetylation status. (A–C) To assess whether particular protein functions or functional domains are associated with the N-terminal acetylation state, a Gene Ontology analysis on a reduced set of GO categories (referred to as GO Slim) on all three levels, namely Cellular Component (A), Molecular Function (B), and Biological Process (C) was performed. The results of this analysis (representative examples with at least two members seen in a category) are also shown in Table S4. For more details please see Text S1. * statistically significant different frequency observed (p<0.05). (1.09 MB TIF) [file pbio.1000236.s002.tif]

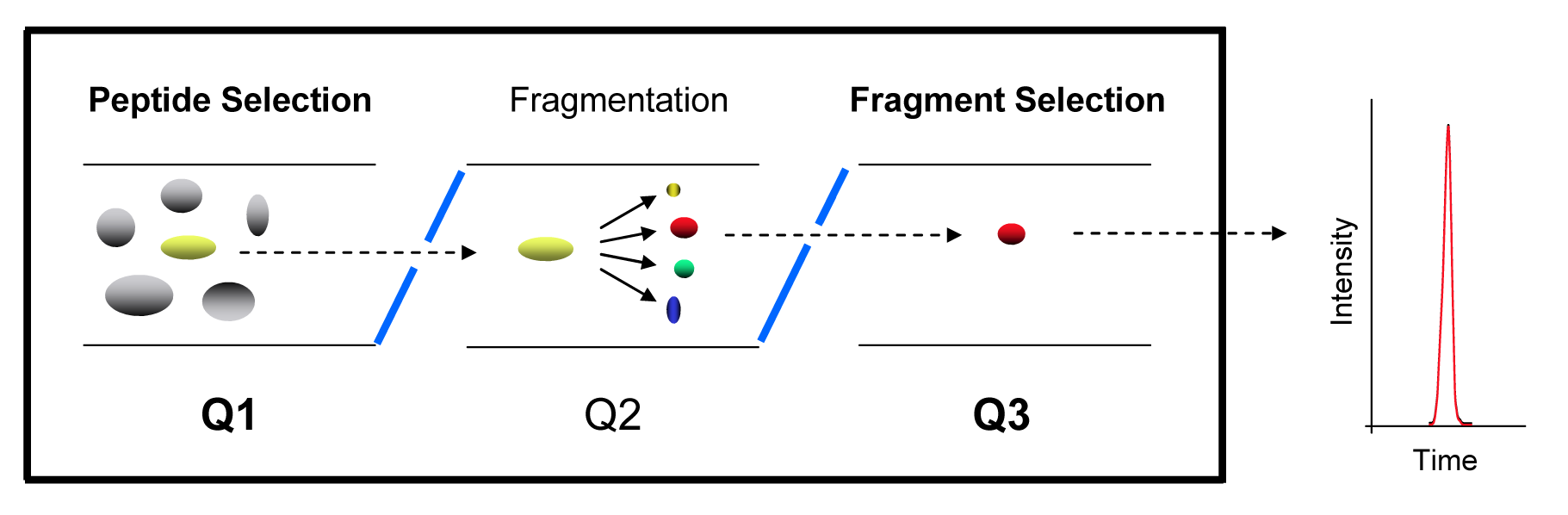

Supplement: Figure S3 — The principle of SRM in a triple quadrupole mass spectrometer. SRM is performed in a specific type of mass spectrometer, which consists of a linear series of three mass analyzers, so-called quadrupoles. The first quadrupole (Q1) functions as a mass filter, capable of selectively transmitting only the parent ion (peptide) of interest from a complex peptide mixture. The second quadrupole (Q2) serves as a fragmentation unit, in which the selected peptide is fragmented into smaller product ions by collisionally activated dissociation. Finally, a single fragment (product ion) that is characteristic for a peptide is passed through to the third mass analyzer Q3 and can be quantified. (0.08 MB TIF) [file pbio.1000236.s003.tif]
