# Supplementary material for: Identification and Functional Characterization of N-Terminally Acetylated Proteins in Drosophila melanogaster
Source: PLoS Biol. 2009 Nov 3;7(11):e1000236. doi: 10.1371/journal.pbio.1000236 (PMC2762599; doi:10.1371/journal.pbio.1000236)
Supplement: Text S1 — Kozak/Cavener analysis of putative alternative start sites, and GO Slim analysis. (0.50 MB DOC) [file pbio.1000236.s014.doc]

Kozak/Cavener analysis of putative alternative start sites

These are the Kozak/Cavener contexts for all ATGs that are in frame with the predicted ORF. Some peptides that indicate the use of alternative start sites can be mapped to different gene models or splice variants (e.g. CG100069 and CG4027 or CG10069-PB and CG10069-PC, respectively). The Cavener and Kozak context for all possible gene models/splice variants are listed. An ***** marks the use of this alternative start sites and the acetylation state of the identified peptide is indicated. The confirmed use of the first ATG of the predicted ORF is marked with ***** (i.e. this N-terminal peptide was identified in this study).

CG10069-PA

CGG TCG ATG AGA :Koz:weak:Cav:weak:Exon 2:ExonStart false|RSMR

CGA AAG ATG AGC :Koz:adequate:Cav:adequate:Exon 2:ExonStart false|RKMS***AcetSite**

TAC AAG ATG TCA :Koz:adequate:Cav:strong:Exon 3:ExonStart false|TKMS

TAT CAC ATG TGC :Koz:weak:Cav:weak:Exon 3:ExonStart false|THMC

TTT GGA ATG CTG :Koz:adequate:Cav:weak:Exon 4:ExonStart false|FGML

ATC GCT ATG TTC :Koz:adequate:Cav:adequate:Exon 4:ExonStart false|IAMF

TTG TCC ATG GGC :Koz:adequate:Cav:weak:Exon 4:ExonStart false|LSMG

ATG GGC ATG ATC :Koz:adequate:Cav:weak:Exon 4:ExonStart false|MGMI

ACT TAC ATG TTC :Koz:weak:Cav:weak:Exon 4:ExonStart false|TTMF

ACC TTC ATG TAC :Koz:weak:Cav:adequate:Exon 5:ExonStart false|TFMT

AGC GGA ATG TCG :Koz:adequate:Cav:adequate:Exon 6:ExonStart false|SGMS

ACA GGC ATG TTG :Koz:adequate:Cav:weak:Exon 6:ExonStart false|TGML

CTG CTG ATG TAT :Koz:weak:Cav:weak:Exon 7:ExonStart false|LLMT

TTG TCG ATG ACA :Koz:weak:Cav:weak:Exon 7:ExonStart false|LSMT

TTC CAC ATG CTC :Koz:weak:Cav:adequate:Exon 7:ExonStart false|FHML

ATT GCC ATG GTG :Koz:strong:Cav:weak:Exon 7:ExonStart false|IAMV

CG10069-PB

AAA TTG ATG AGG :Koz:weak:Cav:weak:Exon 1:ExonStart false|KLMR

CGG TCG ATG AGA :Koz:weak:Cav:weak:Exon 2:ExonStart false|RSMR

CGA AAG ATG AGC :Koz:adequate:Cav:adequate:Exon 2:ExonStart false|RKMS***AcetSite**

TAC AAG ATG TCA :Koz:adequate:Cav:strong:Exon 3:ExonStart false|TKMS

TAT CAC ATG TGC :Koz:weak:Cav:weak:Exon 3:ExonStart false|THMC

TTT GGA ATG CTG :Koz:adequate:Cav:weak:Exon 4:ExonStart false|FGML

ATC GCT ATG TTC :Koz:adequate:Cav:adequate:Exon 4:ExonStart false|IAMF

TTG TCC ATG GGC :Koz:adequate:Cav:weak:Exon 4:ExonStart false|LSMG

ATG GGC ATG ATC :Koz:adequate:Cav:weak:Exon 4:ExonStart false|MGMI

ACT TAC ATG TTC :Koz:weak:Cav:weak:Exon 4:ExonStart false|TTMF

ACC TTC ATG TAC :Koz:weak:Cav:adequate:Exon 5:ExonStart false|TFMT

AGC GGA ATG TCG :Koz:adequate:Cav:adequate:Exon 6:ExonStart false|SGMS

ACA GGC ATG TTG :Koz:adequate:Cav:weak:Exon 6:ExonStart false|TGML

CTG CTG ATG TAT :Koz:weak:Cav:weak:Exon 7:ExonStart false|LLMT

TTG TCG ATG ACA :Koz:weak:Cav:weak:Exon 7:ExonStart false|LSMT

TTC CAC ATG CTC :Koz:weak:Cav:adequate:Exon 7:ExonStart false|FHML

ATT GCC ATG GTG :Koz:strong:Cav:weak:Exon 7:ExonStart false|IAMV

CG10069-PC

CGG TCG ATG AGA :Koz:weak:Cav:weak:Exon 2:ExonStart false|RSMR

CGA AAG ATG AGC :Koz:adequate:Cav:adequate:Exon 2:ExonStart false|RKMS***AcetSite**

TAC AAG ATG TCA :Koz:adequate:Cav:strong:Exon 3:ExonStart false|TKMS

TAT CAC ATG TGC :Koz:weak:Cav:weak:Exon 3:ExonStart false|THMC

TTT GGA ATG CTG :Koz:adequate:Cav:weak:Exon 4:ExonStart false|FGML

ATC GCT ATG TTC :Koz:adequate:Cav:adequate:Exon 4:ExonStart false|IAMF

TTG TCC ATG GGC :Koz:adequate:Cav:weak:Exon 4:ExonStart false|LSMG

ATG GGC ATG ATC :Koz:adequate:Cav:weak:Exon 4:ExonStart false|MGMI

ACT TAC ATG TTC :Koz:weak:Cav:weak:Exon 4:ExonStart false|TTMF

ACC TTC ATG TAC :Koz:weak:Cav:adequate:Exon 5:ExonStart false|TFMT

AGC GGA ATG TCG :Koz:adequate:Cav:adequate:Exon 6:ExonStart false|SGMS

ACA GGC ATG TTG :Koz:adequate:Cav:weak:Exon 6:ExonStart false|TGML

CTG CTG ATG TAT :Koz:weak:Cav:weak:Exon 7:ExonStart false|LLMT

TTG TCG ATG ACA :Koz:weak:Cav:weak:Exon 7:ExonStart false|LSMT

TTC CAC ATG CTC :Koz:weak:Cav:adequate:Exon 7:ExonStart false|FHML

ATT GCC ATG GTG :Koz:strong:Cav:weak:Exon 7:ExonStart false|IAMV

CG1009-PA

CGC AAG ATG GCC :Koz:strong:Cav:strong:Exon 2:ExonStart false|RKMA*****

CTG CAT ATG TCC :Koz:weak:Cav:weak:Exon 3:ExonStart false|LHMS***NonAcetSite**

GAC AAG ATG AAG :Koz:adequate:Cav:strong:Exon 3:ExonStart false|DKMK

TCC AAC ATG CCG :Koz:adequate:Cav:strong:Exon 3:ExonStart false|SNMP

CCC ATC ATG TCC :Koz:adequate:Cav:strong:Exon 3:ExonStart false|PIMS

CCC AAG ATG GAC :Koz:strong:Cav:strong:Exon 3:ExonStart false|PKMD

GGC GCC ATG GAG :Koz:strong:Cav:adequate:Exon 3:ExonStart false|GAME

TCG CTG ATG CGC :Koz:weak:Cav:weak:Exon 3:ExonStart false|SLMR

GTA ACT ATG GAG :Koz:strong:Cav:adequate:Exon 4:ExonStart false|VTME

ACA GAC ATG TAC :Koz:adequate:Cav:weak:Exon 4:ExonStart false|TDMT

ATT CGC ATG CTG :Koz:weak:Cav:weak:Exon 4:ExonStart false|IRML

AAG GGC ATG AAT :Koz:adequate:Cav:weak:Exon 4:ExonStart false|KGMN

GAC GTG ATG ACC :Koz:adequate:Cav:adequate:Exon 4:ExonStart false|DVMT

TCC TCA ATG GAG :Koz:adequate:Cav:adequate:Exon 4:ExonStart false|SSME

CAA GAA ATG CTA :Koz:adequate:Cav:weak:Exon 4:ExonStart false|QEML

CAG CTG ATG CCC :Koz:weak:Cav:weak:Exon 4:ExonStart false|QLMP

GAG AAG ATG GAG :Koz:strong:Cav:adequate:Exon 4:ExonStart false|EKME

GAT GAC ATG TTT :Koz:adequate:Cav:weak:Exon 4:ExonStart false|DDMF

TTT GCT ATG GTG :Koz:strong:Cav:weak:Exon 4:ExonStart false|FAMV

GAT CTT ATG GAG :Koz:adequate:Cav:weak:Exon 4:ExonStart false|DLME

AAT GAG ATG CTT :Koz:adequate:Cav:weak:Exon 4:ExonStart false|NEML

TTT GCC ATG TCG :Koz:adequate:Cav:weak:Exon 4:ExonStart false|FAMS

CGT GAC ATG GCT :Koz:strong:Cav:weak:Exon 5:ExonStart false|RDMA

CG1009-PB

CGC AAG ATG GCC :Koz:strong:Cav:strong:Exon 2:ExonStart false|RKMA*****

CTG CAT ATG TCC :Koz:weak:Cav:weak:Exon 3:ExonStart false|LHMS***NonAcetSite**

GAC AAG ATG AAG :Koz:adequate:Cav:strong:Exon 3:ExonStart false|DKMK

TCC AAC ATG CCG :Koz:adequate:Cav:strong:Exon 3:ExonStart false|SNMP

CCC ATC ATG TCC :Koz:adequate:Cav:strong:Exon 3:ExonStart false|PIMS

CCC AAG ATG GAC :Koz:strong:Cav:strong:Exon 3:ExonStart false|PKMD

GGC GCC ATG GAG :Koz:strong:Cav:adequate:Exon 3:ExonStart false|GAME

TCG CTG ATG CGC :Koz:weak:Cav:weak:Exon 3:ExonStart false|SLMR

GTA ACT ATG GAG :Koz:strong:Cav:adequate:Exon 4:ExonStart false|VTME

ACA GAC ATG TAC :Koz:adequate:Cav:weak:Exon 4:ExonStart false|TDMT

ATT CGC ATG CTG :Koz:weak:Cav:weak:Exon 4:ExonStart false|IRML

AAG GGC ATG AAT :Koz:adequate:Cav:weak:Exon 4:ExonStart false|KGMN

GAC GTG ATG ACC :Koz:adequate:Cav:adequate:Exon 4:ExonStart false|DVMT

TCC TCA ATG GAG :Koz:adequate:Cav:adequate:Exon 4:ExonStart false|SSME

CAA GAA ATG CTA :Koz:adequate:Cav:weak:Exon 4:ExonStart false|QEML

CAG CTG ATG CCC :Koz:weak:Cav:weak:Exon 4:ExonStart false|QLMP

GAG AAG ATG GAG :Koz:strong:Cav:adequate:Exon 4:ExonStart false|EKME

GAT GAC ATG TTT :Koz:adequate:Cav:weak:Exon 4:ExonStart false|DDMF

TTT GCT ATG GTG :Koz:strong:Cav:weak:Exon 4:ExonStart false|FAMV

GAT CTT ATG GAG :Koz:adequate:Cav:weak:Exon 4:ExonStart false|DLME

AAT GAG ATG CTT :Koz:adequate:Cav:weak:Exon 4:ExonStart false|NEML

TTT GCC ATG TCG :Koz:adequate:Cav:weak:Exon 4:ExonStart false|FAMS

CGT GAC ATG GCT :Koz:strong:Cav:weak:Exon 5:ExonStart false|RDMA

CG1009-PC

AAA GTC ATG TAC :Koz:adequate:Cav:weak:Exon 1:ExonStart false|KVMT

CGC AAG ATG GCC :Koz:strong:Cav:strong:Exon 3:ExonStart false|RKMA

CTG CAT ATG TCC :Koz:weak:Cav:weak:Exon 4:ExonStart false|LHMS***NonAcetSite**

GAC AAG ATG AAG :Koz:adequate:Cav:strong:Exon 4:ExonStart false|DKMK

TCC AAC ATG CCG :Koz:adequate:Cav:strong:Exon 4:ExonStart false|SNMP

CCC ATC ATG TCC :Koz:adequate:Cav:strong:Exon 4:ExonStart false|PIMS

CCC AAG ATG GAC :Koz:strong:Cav:strong:Exon 4:ExonStart false|PKMD

GGC GCC ATG GAG :Koz:strong:Cav:adequate:Exon 4:ExonStart false|GAME

TCG CTG ATG CGC :Koz:weak:Cav:weak:Exon 4:ExonStart false|SLMR

GTA ACT ATG GAG :Koz:strong:Cav:adequate:Exon 5:ExonStart false|VTME

ACA GAC ATG TAC :Koz:adequate:Cav:weak:Exon 5:ExonStart false|TDMT

ATT CGC ATG CTG :Koz:weak:Cav:weak:Exon 5:ExonStart false|IRML

AAG GGC ATG AAT :Koz:adequate:Cav:weak:Exon 5:ExonStart false|KGMN

GAC GTG ATG ACC :Koz:adequate:Cav:adequate:Exon 5:ExonStart false|DVMT

TCC TCA ATG GAG :Koz:adequate:Cav:adequate:Exon 5:ExonStart false|SSME

CAA GAA ATG CTA :Koz:adequate:Cav:weak:Exon 5:ExonStart false|QEML

CAG CTG ATG CCC :Koz:weak:Cav:weak:Exon 5:ExonStart false|QLMP

GAG AAG ATG GAG :Koz:strong:Cav:adequate:Exon 5:ExonStart false|EKME

GAT GAC ATG TTT :Koz:adequate:Cav:weak:Exon 5:ExonStart false|DDMF

TTT GCT ATG GTG :Koz:strong:Cav:weak:Exon 5:ExonStart false|FAMV

GAT CTT ATG GAG :Koz:adequate:Cav:weak:Exon 5:ExonStart false|DLME

AAT GAG ATG CTT :Koz:adequate:Cav:weak:Exon 5:ExonStart false|NEML

TTT GCC ATG TCG :Koz:adequate:Cav:weak:Exon 5:ExonStart false|FAMS

CGT GAC ATG GCT :Koz:strong:Cav:weak:Exon 6:ExonStart false|RDMA

CG1009-PD

CGC AAG ATG GCC :Koz:strong:Cav:strong:Exon 2:ExonStart false|RKMA*****

CTG CAT ATG TCC :Koz:weak:Cav:weak:Exon 3:ExonStart false|LHMS***NonAcetSite**

GAC AAG ATG AAG :Koz:adequate:Cav:strong:Exon 3:ExonStart false|DKMK

TCC AAC ATG CCG :Koz:adequate:Cav:strong:Exon 3:ExonStart false|SNMP

CCC ATC ATG TCC :Koz:adequate:Cav:strong:Exon 3:ExonStart false|PIMS

CCC AAG ATG GAC :Koz:strong:Cav:strong:Exon 3:ExonStart false|PKMD

GGC GCC ATG GAG :Koz:strong:Cav:adequate:Exon 3:ExonStart false|GAME

TCG CTG ATG CGC :Koz:weak:Cav:weak:Exon 3:ExonStart false|SLMR

GTA ACT ATG GAG :Koz:strong:Cav:adequate:Exon 4:ExonStart false|VTME

ACA GAC ATG TAC :Koz:adequate:Cav:weak:Exon 4:ExonStart false|TDMT

ATT CGC ATG CTG :Koz:weak:Cav:weak:Exon 4:ExonStart false|IRML

AAG GGC ATG AAT :Koz:adequate:Cav:weak:Exon 4:ExonStart false|KGMN

GAC GTG ATG ACC :Koz:adequate:Cav:adequate:Exon 4:ExonStart false|DVMT

TCC TCA ATG GAG :Koz:adequate:Cav:adequate:Exon 4:ExonStart false|SSME

CAA GAA ATG CTA :Koz:adequate:Cav:weak:Exon 4:ExonStart false|QEML

CAG CTG ATG CCC :Koz:weak:Cav:weak:Exon 4:ExonStart false|QLMP

GAG AAG ATG GAG :Koz:strong:Cav:adequate:Exon 4:ExonStart false|EKME

GAT GAC ATG TTT :Koz:adequate:Cav:weak:Exon 4:ExonStart false|DDMF

TTT GCT ATG GTG :Koz:strong:Cav:weak:Exon 4:ExonStart false|FAMV

GAT CTT ATG GAG :Koz:adequate:Cav:weak:Exon 4:ExonStart false|DLME

AAT GAG ATG CTT :Koz:adequate:Cav:weak:Exon 4:ExonStart false|NEML

TTT GCC ATG TCG :Koz:adequate:Cav:weak:Exon 4:ExonStart false|FAMS

CGT GAC ATG GCT :Koz:strong:Cav:weak:Exon 5:ExonStart false|RDMA

CG1009-PE

AAA GTC ATG TAC :Koz:adequate:Cav:weak:Exon 1:ExonStart false|KVMT

CGC AAG ATG GCC :Koz:strong:Cav:strong:Exon 2:ExonStart false|RKMA

CTG CAT ATG TCC :Koz:weak:Cav:weak:Exon 3:ExonStart false|LHMS***NonAcetSite**

GAC AAG ATG AAG :Koz:adequate:Cav:strong:Exon 3:ExonStart false|DKMK

TCC AAC ATG CCG :Koz:adequate:Cav:strong:Exon 3:ExonStart false|SNMP

CCC ATC ATG TCC :Koz:adequate:Cav:strong:Exon 3:ExonStart false|PIMS

CCC AAG ATG GAC :Koz:strong:Cav:strong:Exon 3:ExonStart false|PKMD

GGC GCC ATG GAG :Koz:strong:Cav:adequate:Exon 3:ExonStart false|GAME

TCG CTG ATG CGC :Koz:weak:Cav:weak:Exon 3:ExonStart false|SLMR

GTA ACT ATG GAG :Koz:strong:Cav:adequate:Exon 4:ExonStart false|VTME

ACA GAC ATG TAC :Koz:adequate:Cav:weak:Exon 4:ExonStart false|TDMT

ATT CGC ATG CTG :Koz:weak:Cav:weak:Exon 4:ExonStart false|IRML

AAG GGC ATG AAT :Koz:adequate:Cav:weak:Exon 4:ExonStart false|KGMN

GAC GTG ATG ACC :Koz:adequate:Cav:adequate:Exon 4:ExonStart false|DVMT

TCC TCA ATG GAG :Koz:adequate:Cav:adequate:Exon 4:ExonStart false|SSME

CAA GAA ATG CTA :Koz:adequate:Cav:weak:Exon 4:ExonStart false|QEML

CAG CTG ATG CCC :Koz:weak:Cav:weak:Exon 4:ExonStart false|QLMP

GAG AAG ATG GAG :Koz:strong:Cav:adequate:Exon 4:ExonStart false|EKME

GAT GAC ATG TTT :Koz:adequate:Cav:weak:Exon 4:ExonStart false|DDMF

TTT GCT ATG GTG :Koz:strong:Cav:weak:Exon 4:ExonStart false|FAMV

GAT CTT ATG GAG :Koz:adequate:Cav:weak:Exon 4:ExonStart false|DLME

AAT GAG ATG CTT :Koz:adequate:Cav:weak:Exon 4:ExonStart false|NEML

TTT GCC ATG TCG :Koz:adequate:Cav:weak:Exon 4:ExonStart false|FAMS

CGT GAC ATG GCT :Koz:strong:Cav:weak:Exon 5:ExonStart false|RDMA

CG1009-PF

CGC AAG ATG GCC :Koz:strong:Cav:strong:Exon 2:ExonStart false|RKMA*****

CTG CAT ATG TCC :Koz:weak:Cav:weak:Exon 3:ExonStart false|LHMS***NonAcetSite**

GAC AAG ATG AAG :Koz:adequate:Cav:strong:Exon 3:ExonStart false|DKMK

TCC AAC ATG CCG :Koz:adequate:Cav:strong:Exon 3:ExonStart false|SNMP

CCC ATC ATG TCC :Koz:adequate:Cav:strong:Exon 3:ExonStart false|PIMS

CCC AAG ATG GAC :Koz:strong:Cav:strong:Exon 3:ExonStart false|PKMD

GGC GCC ATG GAG :Koz:strong:Cav:adequate:Exon 3:ExonStart false|GAME

TCG CTG ATG CGC :Koz:weak:Cav:weak:Exon 3:ExonStart false|SLMR

GTA ACT ATG GAG :Koz:strong:Cav:adequate:Exon 4:ExonStart false|VTME

ACA GAC ATG TAC :Koz:adequate:Cav:weak:Exon 4:ExonStart false|TDMT

ATT CGC ATG CTG :Koz:weak:Cav:weak:Exon 4:ExonStart false|IRML

AAG GGC ATG AAT :Koz:adequate:Cav:weak:Exon 4:ExonStart false|KGMN

GAC GTG ATG ACC :Koz:adequate:Cav:adequate:Exon 4:ExonStart false|DVMT

TCC TCA ATG GAG :Koz:adequate:Cav:adequate:Exon 4:ExonStart false|SSME

CAA GAA ATG CTA :Koz:adequate:Cav:weak:Exon 4:ExonStart false|QEML

CAG CTG ATG CCC :Koz:weak:Cav:weak:Exon 4:ExonStart false|QLMP

GAG AAG ATG GAG :Koz:strong:Cav:adequate:Exon 4:ExonStart false|EKME

GAT GAC ATG TTT :Koz:adequate:Cav:weak:Exon 4:ExonStart false|DDMF

TTT GCT ATG GTG :Koz:strong:Cav:weak:Exon 4:ExonStart false|FAMV

GAT CTT ATG GAG :Koz:adequate:Cav:weak:Exon 4:ExonStart false|DLME

AAT GAG ATG CTT :Koz:adequate:Cav:weak:Exon 4:ExonStart false|NEML

TTT GCC ATG TCG :Koz:adequate:Cav:weak:Exon 4:ExonStart false|FAMS

CGT GAC ATG GCT :Koz:strong:Cav:weak:Exon 5:ExonStart false|RDMA

CG10120-PA

ACC GCC ATG TAC :Koz:adequate:Cav:adequate:Exon 1:ExonStart false|TAMT

GAC AAG ATG AGC :Koz:adequate:Cav:strong:Exon 2:ExonStart false|DKMS***AcetSite**

CAT GGC ATG CTG :Koz:adequate:Cav:weak:Exon 3:ExonStart false|HGML

AAG TAC ATG TAC :Koz:weak:Cav:weak:Exon 3:ExonStart false|KTMT

GCC TAC ATG ATG :Koz:weak:Cav:adequate:Exon 3:ExonStart false|ATMM

TAC ATG ATG CCA :Koz:adequate:Cav:strong:Exon 3:ExonStart false|TMMP

AAG GGC ATG TTC :Koz:adequate:Cav:weak:Exon 3:ExonStart false|KGMF

AAC GGA ATG GGT :Koz:strong:Cav:adequate:Exon 3:ExonStart false|NGMG

GAG TTC ATG CAT :Koz:weak:Cav:weak:Exon 3:ExonStart false|EFMH

TGC CTG ATG GCC :Koz:adequate:Cav:adequate:Exon 3:ExonStart false|CLMA

ATG GCC ATG AAG :Koz:adequate:Cav:weak:Exon 3:ExonStart false|MAMK

ATC TGG ATG GTG :Koz:adequate:Cav:adequate:Exon 3:ExonStart false|IWMV

GAG CTG ATG GCC :Koz:adequate:Cav:weak:Exon 4:ExonStart false|ELMA

GCC GGC ATG CTG :Koz:adequate:Cav:adequate:Exon 4:ExonStart false|AGML

TGT TCG ATG GCC :Koz:adequate:Cav:weak:Exon 4:ExonStart false|CSMA

GCC CAG ATG TAC :Koz:weak:Cav:adequate:Exon 4:ExonStart false|AQMT

TAT AAG ATG TAG :Koz:adequate:Cav:adequate:Exon 4:ExonStart false|TKMstop

CG10120-PB

CCC AAA ATG GGT :Koz:strong:Cav:strong:Exon 1:ExonStart false|PKMG

GAC AAG ATG AGC :Koz:adequate:Cav:strong:Exon 2:ExonStart false|DKMS***AcetSite**

CAT GGC ATG CTG :Koz:adequate:Cav:weak:Exon 3:ExonStart false|HGML

AAG TAC ATG TAC :Koz:weak:Cav:weak:Exon 3:ExonStart false|KTMT

GCC TAC ATG ATG :Koz:weak:Cav:adequate:Exon 3:ExonStart false|ATMM

TAC ATG ATG CCA :Koz:adequate:Cav:strong:Exon 3:ExonStart false|TMMP

AAG GGC ATG TTC :Koz:adequate:Cav:weak:Exon 3:ExonStart false|KGMF

AAC GGA ATG GGT :Koz:strong:Cav:adequate:Exon 3:ExonStart false|NGMG

GAG TTC ATG CAT :Koz:weak:Cav:weak:Exon 3:ExonStart false|EFMH

TGC CTG ATG GCC :Koz:adequate:Cav:adequate:Exon 3:ExonStart false|CLMA

ATG GCC ATG AAG :Koz:adequate:Cav:weak:Exon 3:ExonStart false|MAMK

ATC TGG ATG GTG :Koz:adequate:Cav:adequate:Exon 3:ExonStart false|IWMV

GAG CTG ATG GCC :Koz:adequate:Cav:weak:Exon 4:ExonStart false|ELMA

GCC GGC ATG CTG :Koz:adequate:Cav:adequate:Exon 4:ExonStart false|AGML

TGT TCG ATG GCC :Koz:adequate:Cav:weak:Exon 4:ExonStart false|CSMA

GCC CAG ATG TAC :Koz:weak:Cav:adequate:Exon 4:ExonStart false|AQMT

TAT AAG ATG TAG :Koz:adequate:Cav:adequate:Exon 4:ExonStart false|TKMstop

CG10206-PA

AAC GCG ATG TTT :Koz:adequate:Cav:adequate:Exon 1:ExonStart false|NAMF*****

GAG CTG ATG CGC :Koz:weak:Cav:weak:Exon 1:ExonStart false|ELMR

CGT GAG ATG ACC :Koz:adequate:Cav:weak:Exon 1:ExonStart false|REMT

ACC GCC ATG GCC :Koz:strong:Cav:adequate:Exon 1:ExonStart false|TAMA

GAC ACA ATG ATT :Koz:adequate:Cav:strong:Exon 1:ExonStart false|DTMI

AAC TAC ATG ATG :Koz:weak:Cav:adequate:Exon 1:ExonStart false|NTMM

TAC ATG ATG CGT :Koz:adequate:Cav:strong:Exon 1:ExonStart false|TMMR

GAT AAT ATG GCC :Koz:strong:Cav:adequate:Exon 1:ExonStart false|DNMA

ATC TCT ATG GGT :Koz:adequate:Cav:adequate:Exon 1:ExonStart false|ISMG

GCC AGA ATG ATG :Koz:adequate:Cav:strong:Exon 1:ExonStart false|ARMM

AGA ATG ATG GCC :Koz:strong:Cav:adequate:Exon 1:ExonStart false|RMMA***NonAcetSite**

ATG GCC ATG GCT :Koz:strong:Cav:weak:Exon 1:ExonStart false|MAMA

GGC AAA ATG TCG :Koz:adequate:Cav:strong:Exon 1:ExonStart false|GKMS

CG10223-PA

AGC ATC ATG GAG :Koz:strong:Cav:strong:Exon 2:ExonStart false|SIME

GAA CAG ATG TAC :Koz:weak:Cav:weak:Exon 2:ExonStart false|EQMT

GAG CTG ATG TGG :Koz:weak:Cav:weak:Exon 2:ExonStart false|ELMW

AAC CGC ATG GTG :Koz:adequate:Cav:adequate:Exon 2:ExonStart false|NRMV

AAG AGC ATG AAC :Koz:adequate:Cav:adequate:Exon 2:ExonStart false|KSMN

CGC AAT ATG GTG :Koz:strong:Cav:strong:Exon 2:ExonStart false|RNMV

GTG ACC ATG CAC :Koz:adequate:Cav:adequate:Exon 2:ExonStart false|VTMH

CAG AAG ATG TAC :Koz:adequate:Cav:adequate:Exon 2:ExonStart false|QKMT

CCA ACG ATG ATC :Koz:adequate:Cav:adequate:Exon 2:ExonStart false|PTMI

AAC AAC ATG GGA :Koz:strong:Cav:strong:Exon 2:ExonStart false|NNMG

TTC AAG ATG GAC :Koz:strong:Cav:strong:Exon 3:ExonStart false|FKMD

GCT CTA ATG TCG :Koz:weak:Cav:weak:Exon 3:ExonStart false|ALMS

GAG AAC ATG ACT :Koz:adequate:Cav:adequate:Exon 4:ExonStart false|ENMT

AAC AAC ATG TCC :Koz:adequate:Cav:strong:Exon 4:ExonStart false|NNMS

AAA GTG ATG ATC :Koz:adequate:Cav:weak:Exon 4:ExonStart false|KVMI

ATG ATC ATG ACA :Koz:adequate:Cav:adequate:Exon 4:ExonStart false|MIMT

CAA GAC ATG GAT :Koz:strong:Cav:weak:Exon 4:ExonStart false|QDMD

ATT GTT ATG GCC :Koz:strong:Cav:weak:Exon 4:ExonStart false|IVMA

AAC CAC ATG GAC :Koz:adequate:Cav:adequate:Exon 4:ExonStart false|NHMD

AAG GTG ATG TTC :Koz:adequate:Cav:weak:Exon 4:ExonStart false|KVMF

GCG GAG ATG TCA :Koz:adequate:Cav:weak:Exon 4:ExonStart false|AEMS

CTA CAG ATG ACA :Koz:weak:Cav:weak:Exon 4:ExonStart false|LQMT

ACT ATA ATG TCT :Koz:adequate:Cav:adequate:Exon 4:ExonStart false|TIMS

ATA CCG ATG GTA :Koz:adequate:Cav:weak:Exon 4:ExonStart false|IPMV

GAG ATT ATG AAA :Koz:adequate:Cav:adequate:Exon 4:ExonStart false|EIMK

AGG AAG ATG ATA :Koz:adequate:Cav:adequate:Exon 4:ExonStart false|RKMI

AGT GTG ATG CAT :Koz:adequate:Cav:weak:Exon 4:ExonStart false|SVMH

GGA CGC ATG GAG :Koz:adequate:Cav:weak:Exon 4:ExonStart false|GRME

AAC CAG ATG CAT :Koz:weak:Cav:adequate:Exon 4:ExonStart false|NQMH

AAG GCC ATG TGT :Koz:adequate:Cav:weak:Exon 4:ExonStart false|KAMC

ATT AAA ATG GAG :Koz:strong:Cav:adequate:Exon 4:ExonStart false|IKME***NonAcetSite**

CTG GGT ATG TCC :Koz:adequate:Cav:weak:Exon 4:ExonStart false|LGMS

ATG TCC ATG TGG :Koz:weak:Cav:weak:Exon 4:ExonStart false|MSMW

ATG TGG ATG TTG :Koz:weak:Cav:weak:Exon 4:ExonStart false|MWML

CCG GAG ATG CTT :Koz:adequate:Cav:weak:Exon 4:ExonStart false|PEML

AAG TCA ATG GGA :Koz:adequate:Cav:weak:Exon 4:ExonStart false|KSMG

AAG AAA ATG GCA :Koz:strong:Cav:adequate:Exon 4:ExonStart false|KKMA

GAT GCA ATG GTC :Koz:strong:Cav:weak:Exon 4:ExonStart false|DAMV

CG10275-PA

... ... ATG TGC :Koz:weak:Cav:weak:Exon 1:ExonStart false|nullnullMC

GTA TCG ATG CCG :Koz:weak:Cav:weak:Exon 2:ExonStart false|VSMP

GCC AAG ATG TCA :Koz:adequate:Cav:strong:Exon 2:ExonStart false|AKMS

TAC ATA ATG CGC :Koz:adequate:Cav:strong:Exon 3:ExonStart false|TIMR

AGC TTC ATG AGG :Koz:weak:Cav:adequate:Exon 4:ExonStart false|SFMR

TCC TTG ATG ATG :Koz:weak:Cav:adequate:Exon 4:ExonStart false|SLMM

TTG ATG ATG AAA :Koz:adequate:Cav:adequate:Exon 4:ExonStart false|LMMK

TAT GCT ATG GGA :Koz:strong:Cav:weak:Exon 4:ExonStart false|TAMG

CGC ACA ATG GCC :Koz:strong:Cav:strong:Exon 5:ExonStart false|RTMA

CAG TTC ATG ACA :Koz:weak:Cav:weak:Exon 5:ExonStart false|QFMT

GAG GAG ATG CGC :Koz:adequate:Cav:weak:Exon 5:ExonStart false|EEMR

GGC TGC ATG CGC :Koz:weak:Cav:adequate:Exon 5:ExonStart false|GCMR

GCC GAG ATG ACT :Koz:adequate:Cav:adequate:Exon 5:ExonStart false|AEMT

TCA CCC ATG CAG :Koz:weak:Cav:weak:Exon 6:ExonStart false|SPMQ

CTG GAT ATG CAG :Koz:adequate:Cav:weak:Exon 6:ExonStart false|LDMQ

CTC ATA ATG ATC :Koz:adequate:Cav:strong:Exon 6:ExonStart false|LIMI

GTG GAA ATG TTT :Koz:adequate:Cav:weak:Exon 7:ExonStart false|VEMF***NonAcetSite**

TCC AAT ATG GAA :Koz:strong:Cav:strong:Exon 7:ExonStart false|SNME

AGG ATT ATG TTC :Koz:adequate:Cav:adequate:Exon 7:ExonStart false|RIMF

GTC TCC ATG ATG :Koz:weak:Cav:adequate:Exon 7:ExonStart false|VSMM

TCC ATG ATG GCA :Koz:strong:Cav:strong:Exon 7:ExonStart false|SMMA

TTG GAT ATG AAA :Koz:adequate:Cav:weak:Exon 7:ExonStart false|LDMK

ATT CTC ATG TAC :Koz:weak:Cav:weak:Exon 7:ExonStart false|ILMT

GCC TCA ATG GAT :Koz:adequate:Cav:adequate:Exon 7:ExonStart false|ASMD

GAG GTC ATG TTC :Koz:adequate:Cav:weak:Exon 8:ExonStart false|EVMF

TCT CCC ATG CAC :Koz:weak:Cav:weak:Exon 9:ExonStart false|SPMH

TTG GAA ATG CAG :Koz:adequate:Cav:weak:Exon 9:ExonStart false|LEMQ

CAG TCC ATG TCC :Koz:weak:Cav:weak:Exon 9:ExonStart false|QSMS

GAA AAA ATG TTT :Koz:adequate:Cav:adequate:Exon 9:ExonStart false|EKMF

CAA CTG ATG ATA :Koz:weak:Cav:weak:Exon 9:ExonStart false|QLMI

CTA TAT ATG CAC :Koz:weak:Cav:weak:Exon 9:ExonStart false|LTMH

GTG GCC ATG GCC :Koz:strong:Cav:weak:Exon 9:ExonStart false|VAMA

GAG CCC ATG ATG :Koz:weak:Cav:weak:Exon 9:ExonStart false|EPMM

CCC ATG ATG GTC :Koz:strong:Cav:strong:Exon 9:ExonStart false|PMMV

TAT TTC ATG CAC :Koz:weak:Cav:weak:Exon 10:ExonStart false|TFMH

ACC CAA ATG TTG :Koz:weak:Cav:adequate:Exon 10:ExonStart false|TQML

GTT ATC ATG GAG :Koz:strong:Cav:adequate:Exon 10:ExonStart false|VIME

AAG CAT ATG CAG :Koz:weak:Cav:weak:Exon 10:ExonStart false|KHMQ

ATA CTG ATG TCG :Koz:weak:Cav:weak:Exon 10:ExonStart false|ILMS

CCC CAA ATG ACA :Koz:weak:Cav:adequate:Exon 11:ExonStart false|PQMT

GAT GTG ATG AGT :Koz:adequate:Cav:weak:Exon 11:ExonStart false|DVMS

TTT GGA ATG CCA :Koz:adequate:Cav:weak:Exon 11:ExonStart false|FGMP

TCA GCA ATG CAA :Koz:adequate:Cav:weak:Exon 11:ExonStart false|SAMQ

GCT CCA ATG AGA :Koz:weak:Cav:weak:Exon 11:ExonStart false|APMR

GTG CAT ATG GAA :Koz:adequate:Cav:weak:Exon 11:ExonStart false|VHME

AGC TAT ATG CAA :Koz:weak:Cav:adequate:Exon 11:ExonStart false|STMQ

GTG GTA ATG GAG :Koz:strong:Cav:weak:Exon 11:ExonStart false|VVME

ATA GTA ATG AAG :Koz:adequate:Cav:weak:Exon 11:ExonStart false|IVMK

ACT AGA ATG CCA :Koz:adequate:Cav:adequate:Exon 11:ExonStart false|TRMP

CAG CAA ATG ATG :Koz:weak:Cav:weak:Exon 11:ExonStart false|QQMM

CAA ATG ATG CTG :Koz:adequate:Cav:adequate:Exon 11:ExonStart false|QMML

TAT CAC ATG GAG :Koz:adequate:Cav:weak:Exon 11:ExonStart false|THME

TGC GAT ATG GCG :Koz:strong:Cav:adequate:Exon 11:ExonStart false|CDMA

CG10460-PA

GTC AAG ATG TCC :Koz:adequate:Cav:strong:Exon 1:ExonStart false|VKMS

GAT CTG ATG CGT :Koz:weak:Cav:weak:Exon 2:ExonStart false|DLMR

TGG AAA ATG GGA :Koz:strong:Cav:adequate:Exon 2:ExonStart false|WKMG***AcetSite**

CG10639-PA

AGA GAA ATG GCT :Koz:strong:Cav:weak:Exon 1:ExonStart false|REMA

GAG GGC ATG CAT :Koz:adequate:Cav:weak:Exon 2:ExonStart false|EGMH

CTG CGA ATG ATC :Koz:weak:Cav:weak:Exon 2:ExonStart false|LRMI

GGA GTG ATG GCG :Koz:strong:Cav:weak:Exon 2:ExonStart false|GVMA

CAG CAT ATG GTC :Koz:adequate:Cav:weak:Exon 2:ExonStart false|QHMV

CCG CGA ATG GAC :Koz:adequate:Cav:weak:Exon 2:ExonStart false|PRMD

GTA AAG ATG GCG :Koz:strong:Cav:adequate:Exon 3:ExonStart false|VKMA

AGC GAG ATG TCC :Koz:adequate:Cav:adequate:Exon 3:ExonStart false|SEMS

CAA GCA ATG GAT :Koz:strong:Cav:weak:Exon 3:ExonStart false|QAMD

GCC AAG ATG ATC :Koz:adequate:Cav:strong:Exon 3:ExonStart false|AKMI***NonAcetSite**

CG1065-PA

GAT AAT ATG GCT :Koz:strong:Cav:adequate:Exon 1:ExonStart false|DNMA

GCT TCA ATG AGG :Koz:weak:Cav:weak:Exon 1:ExonStart false|ASMR

CAC GAC ATG GTG :Koz:strong:Cav:adequate:Exon 2:ExonStart false|HDMV

GGC ATC ATG CCG :Koz:adequate:Cav:strong:Exon 3:ExonStart false|GIMP

CGT CGC ATG GGT :Koz:adequate:Cav:weak:Exon 4:ExonStart false|RRMG***AcetSite**

GCC AAA ATG GGC :Koz:strong:Cav:strong:Exon 4:ExonStart false|AKMG

AAG GAG ATG AAG :Koz:adequate:Cav:weak:Exon 4:ExonStart false|KEMK

CG10850-PA

CGG CTG ATG ATT :Koz:weak:Cav:weak:Exon 1:ExonStart false|RLMI

TTA ACC ATG AAT :Koz:adequate:Cav:adequate:Exon 1:ExonStart false|LTMN***AcetSite**

ATA TCC ATG CGC :Koz:weak:Cav:weak:Exon 2:ExonStart false|ISMR

CGG CGC ATG TTC :Koz:weak:Cav:weak:Exon 2:ExonStart false|RRMF

TTC TAT ATG CTG :Koz:weak:Cav:adequate:Exon 2:ExonStart false|FTML

ACT CTG ATG CTG :Koz:weak:Cav:weak:Exon 2:ExonStart false|TLML

AAA GCC ATG TCC :Koz:adequate:Cav:weak:Exon 2:ExonStart false|KAMS

TTC AGC ATG GTG :Koz:strong:Cav:strong:Exon 2:ExonStart false|FSMV

CCT GAG ATG ATG :Koz:adequate:Cav:weak:Exon 2:ExonStart false|PEMM

GAG ATG ATG GAT :Koz:strong:Cav:adequate:Exon 2:ExonStart false|EMMD

ATG GAT ATG TAC :Koz:adequate:Cav:weak:Exon 2:ExonStart false|MDMT

TCA AGA ATG GCC :Koz:strong:Cav:adequate:Exon 2:ExonStart false|SRMA

GGC TAT ATG AAT :Koz:weak:Cav:adequate:Exon 2:ExonStart false|GTMN

CGC CTG ATG AGC :Koz:weak:Cav:adequate:Exon 2:ExonStart false|RLMS

AGC ATT ATG CTG :Koz:adequate:Cav:strong:Exon 2:ExonStart false|SIML

GGT TGG ATG ATA :Koz:weak:Cav:weak:Exon 2:ExonStart false|GWMI

CTT AGG ATG AGG :Koz:adequate:Cav:adequate:Exon 2:ExonStart false|LRMR

TGT GGC ATG GCG :Koz:strong:Cav:weak:Exon 2:ExonStart false|CGMA

ACA ACA ATG CTT :Koz:adequate:Cav:adequate:Exon 2:ExonStart false|TTML

GAG GAA ATG TCC :Koz:adequate:Cav:weak:Exon 2:ExonStart false|EEMS

GCC CAG ATG GAT :Koz:adequate:Cav:adequate:Exon 2:ExonStart false|AQMD

CTC GGT ATG CCG :Koz:adequate:Cav:adequate:Exon 2:ExonStart false|LGMP

CGC TGC ATG CAC :Koz:weak:Cav:adequate:Exon 2:ExonStart false|RCMH

CG10997-PA

ATC GCA ATG TCG :Koz:adequate:Cav:adequate:Exon 1:ExonStart false|IAMS

TAC TTC ATG GAC :Koz:adequate:Cav:adequate:Exon 2:ExonStart false|TFMD

GTG GAT ATG CAG :Koz:adequate:Cav:weak:Exon 2:ExonStart false|VDMQ

CAC ATC ATG AAG :Koz:adequate:Cav:strong:Exon 2:ExonStart false|HIMK

AAG CTG ATG CTG :Koz:weak:Cav:weak:Exon 2:ExonStart false|KLML

GAC ACC ATG TGC :Koz:adequate:Cav:strong:Exon 2:ExonStart false|DTMC

GAG CTG ATG CCG :Koz:weak:Cav:weak:Exon 2:ExonStart false|ELMP

CGC TAC ATG TAT :Koz:weak:Cav:adequate:Exon 3:ExonStart false|RTMT

TAT CAC ATG TAC :Koz:weak:Cav:weak:Exon 3:ExonStart false|THMT

CTC AAA ATG AAG :Koz:adequate:Cav:strong:Exon 4:ExonStart false|LKMK***NonAcetSite**

CG11154-PA

AGA AAA ATG TTC :Koz:adequate:Cav:adequate:Exon 1:ExonStart false|RKMF

ATC GCC ATG GAT :Koz:strong:Cav:adequate:Exon 2:ExonStart false|IAMD

GTT CAG ATG TCT :Koz:weak:Cav:weak:Exon 3:ExonStart false|VQMS

TTG ATC ATG GAG :Koz:strong:Cav:adequate:Exon 3:ExonStart false|LIME

AAT GAG ATG ATC :Koz:adequate:Cav:weak:Exon 3:ExonStart false|NEMI

GGG CAA ATG AAC :Koz:weak:Cav:weak:Exon 3:ExonStart false|GQMN

ACC GAC ATG GGT :Koz:strong:Cav:adequate:Exon 3:ExonStart false|TDMG

GGT TCT ATG CAG :Koz:weak:Cav:weak:Exon 3:ExonStart false|GSMQ

CGA ATC ATG GAT :Koz:strong:Cav:adequate:Exon 3:ExonStart false|RIMD

CTC GGA ATG GAT :Koz:strong:Cav:adequate:Exon 3:ExonStart false|LGMD

TTC TAC ATG GTC :Koz:adequate:Cav:adequate:Exon 3:ExonStart false|FTMV***NonAcetSite**

CG11517-PA

... ... ATG CTG :Koz:weak:Cav:weak:Exon 1:ExonStart false|nullnullML

ATG CTG ATG GGC :Koz:adequate:Cav:weak:Exon 1:ExonStart false|MLMG

AAA TGG ATG AGA :Koz:weak:Cav:weak:Exon 1:ExonStart false|KWMR***NonAcetSite**

CTG GTG ATG ACC :Koz:adequate:Cav:weak:Exon 2:ExonStart false|LVMT

CG11590-PA

ATC TGA ATG CAA :Koz:weak:Cav:adequate:Exon 1:ExonStart false|IstopMQ

TCC AAA ATG GGC :Koz:strong:Cav:strong:Exon 1:ExonStart false|SKMG***AcetSite**

CTG TTG ATG ATC :Koz:weak:Cav:weak:Exon 1:ExonStart false|LLMI

CG12013-PA

ATC GAC ATG TCT :Koz:adequate:Cav:adequate:Exon 2:ExonStart false|IDMS*****

TCC CAG ATG CCG :Koz:weak:Cav:adequate:Exon 2:ExonStart false|SQMP***NonAcetSite**

GAG GCC ATG GTG :Koz:strong:Cav:weak:Exon 2:ExonStart false|EAMV

GAT CCC ATG GAC :Koz:adequate:Cav:weak:Exon 3:ExonStart false|DPMD

CG12013-PB

ATC GAC ATG TCT :Koz:adequate:Cav:adequate:Exon 1:ExonStart false|IDMS*****

TCC CAG ATG CCG :Koz:weak:Cav:adequate:Exon 1:ExonStart false|SQMP***NonAcetSite**

GAG GCC ATG GTG :Koz:strong:Cav:weak:Exon 1:ExonStart false|EAMV

GAT CCC ATG GAC :Koz:adequate:Cav:weak:Exon 2:ExonStart false|DPMD

CG12013-PC

TCA GTA ATG GCT :Koz:strong:Cav:weak:Exon 1:ExonStart false|SVMA

TTC ACC ATG CAA :Koz:adequate:Cav:strong:Exon 1:ExonStart false|FTMQ

ATC GAC ATG TCT :Koz:adequate:Cav:adequate:Exon 2:ExonStart false|IDMS

TCC CAG ATG CCG :Koz:weak:Cav:adequate:Exon 2:ExonStart false|SQMP***NonAcetSite**

GAG GCC ATG GTG :Koz:strong:Cav:weak:Exon 2:ExonStart false|EAMV

GAT CCC ATG GAC :Koz:adequate:Cav:weak:Exon 3:ExonStart false|DPMD

CG12013-PD

TAA AGA ATG AGT :Koz:adequate:Cav:adequate:Exon 2:ExonStart false|stopRMS

TAC TCG ATG AGG :Koz:weak:Cav:adequate:Exon 2:ExonStart false|TSMR

ACC ATC ATG CTT :Koz:adequate:Cav:strong:Exon 2:ExonStart false|TIML

ACG CCC ATG AAC :Koz:weak:Cav:weak:Exon 2:ExonStart false|TPMN

ATC GAC ATG TCT :Koz:adequate:Cav:adequate:Exon 3:ExonStart false|IDMS

TCC CAG ATG CCG :Koz:weak:Cav:adequate:Exon 3:ExonStart false|SQMP***NonAcetSite**

GAG GCC ATG GTG :Koz:strong:Cav:weak:Exon 3:ExonStart false|EAMV

GAT CCC ATG GAC :Koz:adequate:Cav:weak:Exon 4:ExonStart false|DPMD

CG12051-PA

TAC AAA ATG TGT :Koz:adequate:Cav:strong:Exon 2:ExonStart false|TKMC

TCC GGC ATG TGC :Koz:adequate:Cav:adequate:Exon 2:ExonStart false|SGMC***NonAcetSite**

GGC GTA ATG GTA :Koz:strong:Cav:adequate:Exon 2:ExonStart false|GVMV

GTA GGA ATG GGA :Koz:strong:Cav:weak:Exon 2:ExonStart false|VGMG*NonAcetSite

GAC GAC ATG GAG :Koz:strong:Cav:adequate:Exon 2:ExonStart false|DDME

GAG AAG ATG ACT :Koz:adequate:Cav:adequate:Exon 2:ExonStart false|EKMT

CAG ATT ATG TTT :Koz:adequate:Cav:adequate:Exon 2:ExonStart false|QIMF

CCG GCC ATG TAT :Koz:adequate:Cav:weak:Exon 2:ExonStart false|PAMT

TAC CTG ATG AAG :Koz:weak:Cav:adequate:Exon 2:ExonStart false|TLMK

CAG GAG ATG GCC :Koz:strong:Cav:weak:Exon 2:ExonStart false|QEMA

CTC GGC ATG GAG :Koz:strong:Cav:adequate:Exon 2:ExonStart false|LGME

TCA ATC ATG AAG :Koz:adequate:Cav:adequate:Exon 2:ExonStart false|SIMK

ACC ACC ATG TAC :Koz:adequate:Cav:strong:Exon 2:ExonStart false|TTMT

GAC CGC ATG CAA :Koz:weak:Cav:adequate:Exon 2:ExonStart false|DRMQ

TCC ACC ATG AAG :Koz:adequate:Cav:strong:Exon 2:ExonStart false|STMK

CAG CAG ATG TGG :Koz:weak:Cav:weak:Exon 2:ExonStart false|QQMW

CG12079-PA

TGT AGA ATG GCG :Koz:strong:Cav:adequate:Exon 1:ExonStart false|CRMA

CTT CGG ATG GCC :Koz:adequate:Cav:weak:Exon 2:ExonStart false|LRMA***NonAcetSite**

TGG GAC ATG TAC :Koz:adequate:Cav:weak:Exon 3:ExonStart false|WDMT

CG12238-PA

ATA GCA ATG AAT :Koz:adequate:Cav:weak:Exon 2:ExonStart false|IAMN

CAG CAG ATG GTA :Koz:adequate:Cav:weak:Exon 2:ExonStart false|QQMV

GGA CCG ATG GCC :Koz:adequate:Cav:weak:Exon 2:ExonStart false|GPMA***AcetSite**

TCA GCT ATG CCC :Koz:adequate:Cav:weak:Exon 2:ExonStart false|SAMP

GCG GAG ATG CAG :Koz:adequate:Cav:weak:Exon 2:ExonStart false|AEMQ

AAG CTG ATG ATC :Koz:weak:Cav:weak:Exon 2:ExonStart false|KLMI

GTC ATA ATG ACG :Koz:adequate:Cav:strong:Exon 2:ExonStart false|VIMT

ATA GAG ATG ATC :Koz:adequate:Cav:weak:Exon 2:ExonStart false|IEMI

GCT CCC ATG ACG :Koz:weak:Cav:weak:Exon 2:ExonStart false|APMT

GAC GAC ATG GTG :Koz:strong:Cav:adequate:Exon 2:ExonStart false|DDMV

CCG CCC ATG CCC :Koz:weak:Cav:weak:Exon 2:ExonStart false|PPMP

GTG GAC ATG GTG :Koz:strong:Cav:weak:Exon 2:ExonStart false|VDMV

CTG CGG ATG CAA :Koz:weak:Cav:weak:Exon 2:ExonStart false|LRMQ

CGC CTG ATG GCG :Koz:adequate:Cav:adequate:Exon 2:ExonStart false|RLMA

GCC TAC ATG GAC :Koz:adequate:Cav:adequate:Exon 2:ExonStart false|ATMD

AAC CCG ATG CTC :Koz:weak:Cav:adequate:Exon 2:ExonStart false|NPML

CTC GCA ATG GAT :Koz:strong:Cav:adequate:Exon 2:ExonStart false|LAMD

CAG CGT ATG GAT :Koz:adequate:Cav:weak:Exon 2:ExonStart false|QRMD

ATC GAC ATG GTG :Koz:strong:Cav:adequate:Exon 3:ExonStart false|IDMV

GCG GAC ATG AAA :Koz:adequate:Cav:weak:Exon 3:ExonStart false|ADMK

CCG ACG ATG GCT :Koz:strong:Cav:adequate:Exon 3:ExonStart false|PTMA

CTC ACA ATG TGC :Koz:adequate:Cav:strong:Exon 3:ExonStart false|LTMC

AGC TCG ATG CTA :Koz:weak:Cav:adequate:Exon 3:ExonStart false|SSML

TCT ATC ATG GCC :Koz:strong:Cav:adequate:Exon 3:ExonStart false|SIMA

TTG CCT ATG CCC :Koz:weak:Cav:weak:Exon 4:ExonStart false|LPMP

CCG CGA ATG AGC :Koz:weak:Cav:weak:Exon 4:ExonStart false|PRMS

ATC GCC ATG AGT :Koz:adequate:Cav:adequate:Exon 4:ExonStart false|IAMS

ACG CGT ATG AGC :Koz:weak:Cav:weak:Exon 5:ExonStart false|TRMS

CAG TCG ATG GCC :Koz:adequate:Cav:weak:Exon 5:ExonStart false|QSMA

AGC CTG ATG GAA :Koz:adequate:Cav:adequate:Exon 5:ExonStart false|SLME

TCG TCC ATG GTG :Koz:adequate:Cav:weak:Exon 6:ExonStart false|SSMV

GTG AGC ATG ATG :Koz:adequate:Cav:adequate:Exon 6:ExonStart false|VSMM

AGC ATG ATG GAC :Koz:strong:Cav:strong:Exon 6:ExonStart false|SMMD

AAG AAG ATG GAA :Koz:strong:Cav:adequate:Exon 6:ExonStart false|KKME

ACC TTC ATG ATT :Koz:weak:Cav:adequate:Exon 7:ExonStart false|TFMI

GTC GAC ATG GAG :Koz:strong:Cav:adequate:Exon 7:ExonStart false|VDME

GAC ATC ATG TAC :Koz:adequate:Cav:strong:Exon 7:ExonStart false|DIMT

CGT GCC ATG CTT :Koz:adequate:Cav:weak:Exon 7:ExonStart false|RAML

TCC TGC ATG GAC :Koz:adequate:Cav:adequate:Exon 8:ExonStart false|SCMD

TTC TCA ATG GCC :Koz:adequate:Cav:adequate:Exon 8:ExonStart false|FSMA

GAT CTG ATG GCT :Koz:adequate:Cav:weak:Exon 9:ExonStart false|DLMA

AGG GAC ATG CCG :Koz:adequate:Cav:weak:Exon 10:ExonStart false|RDMP

GTT GAT ATG CCA :Koz:adequate:Cav:weak:Exon 11:ExonStart false|VDMP

CCG CGC ATG GTG :Koz:adequate:Cav:weak:Exon 11:ExonStart false|PRMV

GGA AAG ATG CTC :Koz:adequate:Cav:adequate:Exon 11:ExonStart false|GKML

TTC TGC ATG CGA :Koz:weak:Cav:adequate:Exon 12:ExonStart false|FCMR

GCC GCC ATG TTC :Koz:adequate:Cav:adequate:Exon 12:ExonStart false|AAMF

AGT CCG ATG CCC :Koz:weak:Cav:weak:Exon 12:ExonStart false|SPMP

ACA ACG ATG CAG :Koz:adequate:Cav:adequate:Exon 12:ExonStart false|TTMQ

CG12276-PA

TAA ATA ATG GTC :Koz:strong:Cav:adequate:Exon 1:ExonStart false|stopIMV*****

GTG GAT ATG GAC :Koz:strong:Cav:weak:Exon 1:ExonStart false|VDMD***AcetSite**

AAT CCC ATG GTG :Koz:adequate:Cav:weak:Exon 2:ExonStart false|NPMV

CG1236-PA

AAC TCA ATG CCA :Koz:weak:Cav:adequate:Exon 1:ExonStart false|NSMP

CGA AGG ATG AGC :Koz:adequate:Cav:adequate:Exon 1:ExonStart false|RRMS***AcetSite**

GCC CCC ATG TGG :Koz:weak:Cav:adequate:Exon 2:ExonStart false|APMW

ATG TGG ATG TGC :Koz:weak:Cav:weak:Exon 2:ExonStart false|MWMC

GAC GAG ATG CTT :Koz:adequate:Cav:adequate:Exon 2:ExonStart false|DEML

CAG AAG ATG AAG :Koz:adequate:Cav:adequate:Exon 2:ExonStart false|QKMK

AAG GAA ATG TCC :Koz:adequate:Cav:weak:Exon 2:ExonStart false|KEMS

GAT AAA ATG GTA :Koz:strong:Cav:adequate:Exon 2:ExonStart false|DKMV

CG1242-PA

TAC AAG ATG CCA :Koz:adequate:Cav:strong:Exon 1:ExonStart false|TKMP

CAG CTG ATG TCC :Koz:weak:Cav:weak:Exon 2:ExonStart false|QLMS

ATC GGT ATG ACC :Koz:adequate:Cav:adequate:Exon 2:ExonStart false|IGMT

GCC TTC ATG GAG :Koz:adequate:Cav:adequate:Exon 2:ExonStart false|AFME

ATT TCC ATG ATC :Koz:weak:Cav:weak:Exon 2:ExonStart false|ISMI

AAG GAG ATG GAG :Koz:strong:Cav:weak:Exon 2:ExonStart false|KEME

TTC ATC ATG GAC :Koz:strong:Cav:strong:Exon 2:ExonStart false|FIMD

AAC TTC ATG AAG :Koz:weak:Cav:adequate:Exon 2:ExonStart false|NFMK

CGT GAG ATG CTG :Koz:adequate:Cav:weak:Exon 2:ExonStart false|REML***NonAcetSite**

AAG ACC ATG GAG :Koz:strong:Cav:adequate:Exon 2:ExonStart false|KTME

TCG CGC ATG AAG :Koz:weak:Cav:weak:Exon 2:ExonStart false|SRMK

GTC TAC ATG ACC :Koz:weak:Cav:adequate:Exon 2:ExonStart false|VTMT

AAG CTG ATG AAG :Koz:weak:Cav:weak:Exon 2:ExonStart false|KLMK

GCT AAC ATG GAG :Koz:strong:Cav:adequate:Exon 2:ExonStart false|ANME

CGC ATC ATG AAG :Koz:adequate:Cav:strong:Exon 2:ExonStart false|RIMK

GCC ACA ATG GGC :Koz:strong:Cav:strong:Exon 2:ExonStart false|ATMG

GGC TAC ATG GCC :Koz:adequate:Cav:adequate:Exon 2:ExonStart false|GTMA

TAC CGC ATG ATC :Koz:weak:Cav:adequate:Exon 2:ExonStart false|TRMI

GAG CCT ATG ACT :Koz:weak:Cav:weak:Exon 2:ExonStart false|EPMT

TCC CAC ATG GAG :Koz:adequate:Cav:adequate:Exon 2:ExonStart false|SHME

CG13425-PA

CTG AGA ATG AAG :Koz:adequate:Cav:adequate:Exon 2:ExonStart false|LRMK

CGT GAA ATG AAC :Koz:adequate:Cav:weak:Exon 2:ExonStart false|REMN***AcetSite**

CAG AAG ATG AGG :Koz:adequate:Cav:adequate:Exon 3:ExonStart false|QKMR

ACC GAA ATG CTG :Koz:adequate:Cav:adequate:Exon 5:ExonStart false|TEML

GAT CCT ATG AAC :Koz:weak:Cav:weak:Exon 7:ExonStart false|DPMN

GCT GGA ATG GGG :Koz:strong:Cav:weak:Exon 7:ExonStart false|AGMG

ATA CAA ATG GCC :Koz:adequate:Cav:weak:Exon 8:ExonStart false|IQMA

CG13425-PC

AAG AAC ATG ACA :Koz:adequate:Cav:adequate:Exon 2:ExonStart false|KNMT

CTG AGA ATG AAG :Koz:adequate:Cav:adequate:Exon 3:ExonStart false|LRMK

CGT GAA ATG AAC :Koz:adequate:Cav:weak:Exon 3:ExonStart false|REMN***AcetSite**

CAG AAG ATG AGG :Koz:adequate:Cav:adequate:Exon 4:ExonStart false|QKMR

ACC GAA ATG CTG :Koz:adequate:Cav:adequate:Exon 6:ExonStart false|TEML

GAT CCT ATG AAC :Koz:weak:Cav:weak:Exon 8:ExonStart false|DPMN

GCT GGA ATG GGG :Koz:strong:Cav:weak:Exon 8:ExonStart false|AGMG

ATA CAA ATG GCC :Koz:adequate:Cav:weak:Exon 9:ExonStart false|IQMA

CG13645-PA

GAA CAG ATG ATT :Koz:weak:Cav:weak:Exon 1:ExonStart false|EQMI

AAG AAA ATG TCA :Koz:adequate:Cav:adequate:Exon 1:ExonStart false|KKMS***AcetSite**

ACA CCC ATG CAC :Koz:weak:Cav:weak:Exon 1:ExonStart false|TPMH

CTT CGG ATG TTC :Koz:weak:Cav:weak:Exon 1:ExonStart false|LRMF

TTC GAA ATG CAG :Koz:adequate:Cav:adequate:Exon 2:ExonStart false|FEMQ

TGT GCC ATG GTC :Koz:strong:Cav:weak:Exon 2:ExonStart false|CAMV

CAG TGG ATG CGC :Koz:weak:Cav:weak:Exon 2:ExonStart false|QWMR

CAG GAC ATG GAC :Koz:strong:Cav:weak:Exon 5:ExonStart false|QDMD

ATC ACC ATG CAG :Koz:adequate:Cav:strong:Exon 5:ExonStart false|ITMQ

CG13645-PB

GAA CAG ATG ATT :Koz:weak:Cav:weak:Exon 1:ExonStart false|EQMI

AAG AAA ATG TCA :Koz:adequate:Cav:adequate:Exon 1:ExonStart false|KKMS***AcetSite**

ACA CCC ATG CAC :Koz:weak:Cav:weak:Exon 1:ExonStart false|TPMH

CTT CGG ATG TTC :Koz:weak:Cav:weak:Exon 1:ExonStart false|LRMF

TTC GAA ATG CAG :Koz:adequate:Cav:adequate:Exon 2:ExonStart false|FEMQ

TGT GCC ATG GTC :Koz:strong:Cav:weak:Exon 2:ExonStart false|CAMV

CAG TGG ATG CGC :Koz:weak:Cav:weak:Exon 2:ExonStart false|QWMR

CG13751-PA

GGA AAA ATG CCT :Koz:adequate:Cav:adequate:Exon 1:ExonStart false|GKMP

AAA GCC ATG GCA :Koz:strong:Cav:weak:Exon 1:ExonStart false|KAMA*AcetSite

GAA AAC ATG CAG :Koz:adequate:Cav:adequate:Exon 1:ExonStart false|ENMQ

CG1404-PA

AGA AGG ATG GCT :Koz:strong:Cav:adequate:Exon 1:ExonStart false|RRMA*****

CGG CAC ATG ACC :Koz:weak:Cav:weak:Exon 1:ExonStart false|RHMT

GTC ATC ATG TTC :Koz:adequate:Cav:strong:Exon 1:ExonStart false|VIMF

GTC GCC ATG CCA :Koz:adequate:Cav:adequate:Exon 2:ExonStart false|VAMP

GTT AAG ATG GAT :Koz:strong:Cav:adequate:Exon 2:ExonStart false|VKMD***NonAcetSite**

CG1404-PB

AGA AGG ATG GCT :Koz:strong:Cav:adequate:Exon 2:ExonStart false|RRMA*****

CGG CAC ATG ACC :Koz:weak:Cav:weak:Exon 2:ExonStart false|RHMT

GTC ATC ATG TTC :Koz:adequate:Cav:strong:Exon 2:ExonStart false|VIMF

GTC GCC ATG CCA :Koz:adequate:Cav:adequate:Exon 3:ExonStart false|VAMP

GTT AAG ATG GAT :Koz:strong:Cav:adequate:Exon 3:ExonStart false|VKMD***NonAcetSite**

CG14438-PA

TGC AAA ATG GAG :Koz:strong:Cav:strong:Exon 2:ExonStart false|CKME

ACC TCG ATG AAG :Koz:weak:Cav:adequate:Exon 2:ExonStart false|TSMK

GTA TCG ATG AGC :Koz:weak:Cav:weak:Exon 3:ExonStart false|VSMS

ACT TCA ATG GAT :Koz:adequate:Cav:weak:Exon 4:ExonStart false|TSMD

GCT GTC ATG CAG :Koz:adequate:Cav:weak:Exon 4:ExonStart false|AVMQ

CCA AGC ATG CCT :Koz:adequate:Cav:adequate:Exon 4:ExonStart false|PSMP

CCG CCT ATG CGG :Koz:weak:Cav:weak:Exon 4:ExonStart false|PPMR

CCA AGG ATG AAG :Koz:adequate:Cav:adequate:Exon 4:ExonStart false|PRMK

CGG GAG ATG GAA :Koz:strong:Cav:weak:Exon 4:ExonStart false|REME

CGT AGA ATG TCC :Koz:adequate:Cav:adequate:Exon 4:ExonStart false|RRMS

AAA TCC ATG AAA :Koz:weak:Cav:weak:Exon 4:ExonStart false|KSMK

GTC GAG ATG AAG :Koz:adequate:Cav:adequate:Exon 4:ExonStart false|VEMK

GAC AAC ATG GCA :Koz:strong:Cav:strong:Exon 4:ExonStart false|DNMA

CGG ATT ATG CAG :Koz:adequate:Cav:adequate:Exon 4:ExonStart false|RIMQ

ACG TAT ATG AAG :Koz:weak:Cav:weak:Exon 4:ExonStart false|TTMK

AAG CCG ATG GTG :Koz:adequate:Cav:weak:Exon 4:ExonStart false|KPMV

TTT GGC ATG GGC :Koz:strong:Cav:weak:Exon 4:ExonStart false|FGMG

GTG GAG ATG AAG :Koz:adequate:Cav:weak:Exon 4:ExonStart false|VEMK

GCG CAA ATG TAT :Koz:weak:Cav:weak:Exon 4:ExonStart false|AQMT

GAA GTG ATG CGT :Koz:adequate:Cav:weak:Exon 4:ExonStart false|EVMR

GCA TTG ATG GAG :Koz:adequate:Cav:weak:Exon 4:ExonStart false|ALME

ATG GAG ATG ATA :Koz:adequate:Cav:weak:Exon 4:ExonStart false|MEMI

CAT TAT ATG ATG :Koz:weak:Cav:weak:Exon 4:ExonStart false|HTMM

TAT ATG ATG TGC :Koz:adequate:Cav:adequate:Exon 4:ExonStart false|TMMC

AAA GGC ATG ATC :Koz:adequate:Cav:weak:Exon 4:ExonStart false|KGMI

GGC CAT ATG GGC :Koz:adequate:Cav:adequate:Exon 4:ExonStart false|GHMG

TGT AAT ATG CCA :Koz:adequate:Cav:adequate:Exon 4:ExonStart false|CNMP

CTA CCG ATG AGT :Koz:weak:Cav:weak:Exon 4:ExonStart false|LPMS

CAG TTT ATG CAG :Koz:weak:Cav:weak:Exon 4:ExonStart false|QFMQ

GAA AAT ATG GAC :Koz:strong:Cav:adequate:Exon 4:ExonStart false|ENMD

GAG TTG ATG CTC :Koz:weak:Cav:weak:Exon 4:ExonStart false|ELML

CGC TTC ATG AAA :Koz:weak:Cav:adequate:Exon 4:ExonStart false|RFMK

ATG AAA ATG GTC :Koz:strong:Cav:adequate:Exon 4:ExonStart false|MKMV

CAG AAC ATG ACC :Koz:adequate:Cav:adequate:Exon 4:ExonStart false|QNMT

TGT CTA ATG ACC :Koz:weak:Cav:weak:Exon 4:ExonStart false|CLMT

TCT GAA ATG GAC :Koz:strong:Cav:weak:Exon 4:ExonStart false|SEMD

ACG GAC ATG GAA :Koz:strong:Cav:weak:Exon 4:ExonStart false|TDME

CAA CAT ATG AGC :Koz:weak:Cav:weak:Exon 4:ExonStart false|QHMS

AGC TTA ATG GTA :Koz:adequate:Cav:adequate:Exon 4:ExonStart false|SLMV

ACT TCT ATG TTA :Koz:weak:Cav:weak:Exon 4:ExonStart false|TSML

TAT GAG ATG GAT :Koz:strong:Cav:weak:Exon 4:ExonStart false|TEMD

GAG CTA ATG CCA :Koz:weak:Cav:weak:Exon 4:ExonStart false|ELMP

CCA ACA ATG CAA :Koz:adequate:Cav:adequate:Exon 4:ExonStart false|PTMQ

CCA GAG ATG GGG :Koz:strong:Cav:weak:Exon 4:ExonStart false|PEMG

TGT GGC ATG TGC :Koz:adequate:Cav:weak:Exon 4:ExonStart false|CGMC

CGC CAC ATG AGG :Koz:weak:Cav:adequate:Exon 4:ExonStart false|RHMR

GTG CAC ATG AAG :Koz:weak:Cav:weak:Exon 4:ExonStart false|VHMK

AAT GGG ATG CTA :Koz:adequate:Cav:weak:Exon 4:ExonStart false|NGML

AGG TGC ATG GCC :Koz:adequate:Cav:weak:Exon 4:ExonStart false|RCMA

AAG CTC ATG TTC :Koz:weak:Cav:weak:Exon 4:ExonStart false|KLMF

ATG TTC ATG CGG :Koz:weak:Cav:weak:Exon 4:ExonStart false|MFMR

GAG CAC ATG AAG :Koz:weak:Cav:weak:Exon 4:ExonStart false|EHMK

CGG GAT ATG CTC :Koz:adequate:Cav:weak:Exon 4:ExonStart false|RDML

AAG GCG ATG CAT :Koz:adequate:Cav:weak:Exon 5:ExonStart false|KAMH

CGC ATA ATG ATG :Koz:adequate:Cav:strong:Exon 5:ExonStart false|RIMM

ATA ATG ATG TAT :Koz:adequate:Cav:adequate:Exon 5:ExonStart false|IMMT

TAT CAC ATG AGC :Koz:weak:Cav:weak:Exon 5:ExonStart false|THMS

CTC CAG ATG CAG :Koz:weak:Cav:adequate:Exon 5:ExonStart false|LQMQ

GAT GAA ATG GAC :Koz:strong:Cav:weak:Exon 5:ExonStart false|DEMD

GTT AAC ATG CAG :Koz:adequate:Cav:adequate:Exon 5:ExonStart false|VNMQ

ATG CAG ATG CAC :Koz:weak:Cav:weak:Exon 5:ExonStart false|MQMH

AAA CAC ATG CAG :Koz:weak:Cav:weak:Exon 5:ExonStart false|KHMQ

CTT GCA ATG CAT :Koz:adequate:Cav:weak:Exon 5:ExonStart false|LAMH

GTG GAC ATG ATC :Koz:adequate:Cav:weak:Exon 5:ExonStart false|VDMI

TCG CTA ATG GAG :Koz:adequate:Cav:weak:Exon 6:ExonStart false|SLME

CAG ATC ATG GCC :Koz:strong:Cav:adequate:Exon 6:ExonStart false|QIMA

ATC GCT ATG CAC :Koz:adequate:Cav:adequate:Exon 6:ExonStart false|IAMH

CTG GCA ATG GTG :Koz:strong:Cav:weak:Exon 6:ExonStart false|LAMV

CAG CCG ATG CTG :Koz:weak:Cav:weak:Exon 7:ExonStart false|QPML

CGA CCG ATG GAG :Koz:adequate:Cav:weak:Exon 7:ExonStart false|RPME

GCC CTG ATG CTG :Koz:weak:Cav:adequate:Exon 7:ExonStart false|ALML

GTG GTT ATG CCA :Koz:adequate:Cav:weak:Exon 7:ExonStart false|VVMP

TTC TGC ATG TTC :Koz:weak:Cav:adequate:Exon 7:ExonStart false|FCMF

CGC CAT ATG GTG :Koz:adequate:Cav:adequate:Exon 7:ExonStart false|RHMV

AAA TGT ATG CTT :Koz:weak:Cav:weak:Exon 7:ExonStart false|KCML***NonAcetSite**

GAG CTA ATG GCC :Koz:adequate:Cav:weak:Exon 7:ExonStart false|ELMA

GTT CAG ATG CAG :Koz:weak:Cav:weak:Exon 7:ExonStart false|VQMQ

CG14438-PB

TGC AAA ATG GAG :Koz:strong:Cav:strong:Exon 2:ExonStart false|CKME

ACC TCG ATG AAG :Koz:weak:Cav:adequate:Exon 2:ExonStart false|TSMK

GTA TCG ATG AGC :Koz:weak:Cav:weak:Exon 3:ExonStart false|VSMS

ACT TCA ATG GAT :Koz:adequate:Cav:weak:Exon 4:ExonStart false|TSMD

GCT GTC ATG CAG :Koz:adequate:Cav:weak:Exon 4:ExonStart false|AVMQ

CCA AGC ATG CCT :Koz:adequate:Cav:adequate:Exon 4:ExonStart false|PSMP

CCG CCT ATG CGG :Koz:weak:Cav:weak:Exon 4:ExonStart false|PPMR

CCA AGG ATG AAG :Koz:adequate:Cav:adequate:Exon 4:ExonStart false|PRMK

CGG GAG ATG GAA :Koz:strong:Cav:weak:Exon 4:ExonStart false|REME

CGT AGA ATG TCC :Koz:adequate:Cav:adequate:Exon 4:ExonStart false|RRMS

AAA TCC ATG AAA :Koz:weak:Cav:weak:Exon 4:ExonStart false|KSMK

GTC GAG ATG AAG :Koz:adequate:Cav:adequate:Exon 4:ExonStart false|VEMK

GAC AAC ATG GCA :Koz:strong:Cav:strong:Exon 4:ExonStart false|DNMA

CGG ATT ATG CAG :Koz:adequate:Cav:adequate:Exon 4:ExonStart false|RIMQ

ACG TAT ATG AAG :Koz:weak:Cav:weak:Exon 4:ExonStart false|TTMK

AAG CCG ATG GTG :Koz:adequate:Cav:weak:Exon 4:ExonStart false|KPMV

TTT GGC ATG GGC :Koz:strong:Cav:weak:Exon 4:ExonStart false|FGMG

GTG GAG ATG AAG :Koz:adequate:Cav:weak:Exon 4:ExonStart false|VEMK

GCG CAA ATG TAT :Koz:weak:Cav:weak:Exon 4:ExonStart false|AQMT

GAA GTG ATG CGT :Koz:adequate:Cav:weak:Exon 4:ExonStart false|EVMR

GCA TTG ATG GAG :Koz:adequate:Cav:weak:Exon 4:ExonStart false|ALME

ATG GAG ATG ATA :Koz:adequate:Cav:weak:Exon 4:ExonStart false|MEMI

CAT TAT ATG ATG :Koz:weak:Cav:weak:Exon 4:ExonStart false|HTMM

TAT ATG ATG TGC :Koz:adequate:Cav:adequate:Exon 4:ExonStart false|TMMC

AAA GGC ATG ATC :Koz:adequate:Cav:weak:Exon 4:ExonStart false|KGMI

GGC CAT ATG GGC :Koz:adequate:Cav:adequate:Exon 4:ExonStart false|GHMG

TGT AAT ATG CCA :Koz:adequate:Cav:adequate:Exon 4:ExonStart false|CNMP

CTA CCG ATG AGT :Koz:weak:Cav:weak:Exon 4:ExonStart false|LPMS

CAG TTT ATG CAG :Koz:weak:Cav:weak:Exon 4:ExonStart false|QFMQ

GAA AAT ATG GAC :Koz:strong:Cav:adequate:Exon 4:ExonStart false|ENMD

GAG TTG ATG CTC :Koz:weak:Cav:weak:Exon 4:ExonStart false|ELML

CGC TTC ATG AAA :Koz:weak:Cav:adequate:Exon 4:ExonStart false|RFMK

ATG AAA ATG GTC :Koz:strong:Cav:adequate:Exon 4:ExonStart false|MKMV

CAG AAC ATG ACC :Koz:adequate:Cav:adequate:Exon 4:ExonStart false|QNMT

TGT CTA ATG ACC :Koz:weak:Cav:weak:Exon 4:ExonStart false|CLMT

TCT GAA ATG GAC :Koz:strong:Cav:weak:Exon 4:ExonStart false|SEMD

ACG GAC ATG GAA :Koz:strong:Cav:weak:Exon 4:ExonStart false|TDME

CAA CAT ATG AGC :Koz:weak:Cav:weak:Exon 4:ExonStart false|QHMS

AGC TTA ATG GTA :Koz:adequate:Cav:adequate:Exon 4:ExonStart false|SLMV

ACT TCT ATG TTA :Koz:weak:Cav:weak:Exon 4:ExonStart false|TSML

TAT GAG ATG GAT :Koz:strong:Cav:weak:Exon 4:ExonStart false|TEMD

GAG CTA ATG CCA :Koz:weak:Cav:weak:Exon 4:ExonStart false|ELMP

CCA ACA ATG CAA :Koz:adequate:Cav:adequate:Exon 4:ExonStart false|PTMQ

CCA GAG ATG GGG :Koz:strong:Cav:weak:Exon 4:ExonStart false|PEMG

TGT GGC ATG TGC :Koz:adequate:Cav:weak:Exon 4:ExonStart false|CGMC

CGC CAC ATG AGG :Koz:weak:Cav:adequate:Exon 4:ExonStart false|RHMR

GTG CAC ATG AAG :Koz:weak:Cav:weak:Exon 4:ExonStart false|VHMK

AAT GGG ATG CTA :Koz:adequate:Cav:weak:Exon 4:ExonStart false|NGML

AGG TGC ATG GCC :Koz:adequate:Cav:weak:Exon 4:ExonStart false|RCMA

AAG CTC ATG TTC :Koz:weak:Cav:weak:Exon 4:ExonStart false|KLMF

ATG TTC ATG CGG :Koz:weak:Cav:weak:Exon 4:ExonStart false|MFMR

GAG CAC ATG AAG :Koz:weak:Cav:weak:Exon 4:ExonStart false|EHMK

CGG GAT ATG CTC :Koz:adequate:Cav:weak:Exon 4:ExonStart false|RDML

AAG GCG ATG CAT :Koz:adequate:Cav:weak:Exon 5:ExonStart false|KAMH

CGC ATA ATG ATG :Koz:adequate:Cav:strong:Exon 5:ExonStart false|RIMM

ATA ATG ATG TAT :Koz:adequate:Cav:adequate:Exon 5:ExonStart false|IMMT

TAT CAC ATG AGC :Koz:weak:Cav:weak:Exon 5:ExonStart false|THMS

CTC CAG ATG CAG :Koz:weak:Cav:adequate:Exon 5:ExonStart false|LQMQ

GAT GAA ATG GAC :Koz:strong:Cav:weak:Exon 5:ExonStart false|DEMD

GTT AAC ATG CAG :Koz:adequate:Cav:adequate:Exon 5:ExonStart false|VNMQ

ATG CAG ATG CAC :Koz:weak:Cav:weak:Exon 5:ExonStart false|MQMH

AAA CAC ATG CAG :Koz:weak:Cav:weak:Exon 5:ExonStart false|KHMQ

CTT GCA ATG CAT :Koz:adequate:Cav:weak:Exon 5:ExonStart false|LAMH

GTG GAC ATG ATC :Koz:adequate:Cav:weak:Exon 5:ExonStart false|VDMI

TCG CTA ATG GAG :Koz:adequate:Cav:weak:Exon 6:ExonStart false|SLME

CAG ATC ATG GCC :Koz:strong:Cav:adequate:Exon 6:ExonStart false|QIMA

ATC GCT ATG CAC :Koz:adequate:Cav:adequate:Exon 6:ExonStart false|IAMH

CTG GCA ATG GTG :Koz:strong:Cav:weak:Exon 6:ExonStart false|LAMV

CAG CCG ATG CTG :Koz:weak:Cav:weak:Exon 7:ExonStart false|QPML

CGA CCG ATG GAG :Koz:adequate:Cav:weak:Exon 7:ExonStart false|RPME

GCC CTG ATG CTG :Koz:weak:Cav:adequate:Exon 7:ExonStart false|ALML

GTG GTT ATG CCA :Koz:adequate:Cav:weak:Exon 7:ExonStart false|VVMP

TTC TGC ATG TTC :Koz:weak:Cav:adequate:Exon 7:ExonStart false|FCMF

CGC CAT ATG GTG :Koz:adequate:Cav:adequate:Exon 7:ExonStart false|RHMV

AAA TGT ATG CTT :Koz:weak:Cav:weak:Exon 7:ExonStart false|KCML***NonAcetSite**

GAG CTA ATG GCC :Koz:adequate:Cav:weak:Exon 7:ExonStart false|ELMA

GTT CAG ATG CAG :Koz:weak:Cav:weak:Exon 7:ExonStart false|VQMQ

CG14998-PA

TGA AAT ATG GCG :Koz:strong:Cav:adequate:Exon 1:ExonStart false|stopNMA*****

AGC GCC ATG TCC :Koz:adequate:Cav:adequate:Exon 7:ExonStart false|SAMS

ACA AAC ATG TCC :Koz:adequate:Cav:adequate:Exon 7:ExonStart false|TNMS

ACG GGC ATG TCT :Koz:adequate:Cav:weak:Exon 7:ExonStart false|TGMS

AAC CTT ATG TCC :Koz:weak:Cav:adequate:Exon 8:ExonStart false|NLMS***NonAcetSite**

TCT GCA ATG ACT :Koz:adequate:Cav:weak:Exon 9:ExonStart false|SAMT

AGG TCG ATG ATT :Koz:weak:Cav:weak:Exon 9:ExonStart false|RSMI

GAT CTG ATG ACC :Koz:weak:Cav:weak:Exon 9:ExonStart false|DLMT

GCT TCG ATG ATG :Koz:weak:Cav:weak:Exon 9:ExonStart false|ASMM

TCG ATG ATG GCC :Koz:strong:Cav:adequate:Exon 9:ExonStart false|SMMA

GCC ATC ATG CTG :Koz:adequate:Cav:strong:Exon 10:ExonStart false|AIML

CAG GCG ATG TAC :Koz:adequate:Cav:weak:Exon 11:ExonStart false|QAMT

TCC ACG ATG ATC :Koz:adequate:Cav:strong:Exon 11:ExonStart false|STMI

CG14998-PB

TGA AAT ATG GCG :Koz:strong:Cav:adequate:Exon 1:ExonStart false|stopNMA*****

AGC GCC ATG TCC :Koz:adequate:Cav:adequate:Exon 6:ExonStart false|SAMS

ACA AAC ATG TCC :Koz:adequate:Cav:adequate:Exon 6:ExonStart false|TNMS

ACG GGC ATG TCT :Koz:adequate:Cav:weak:Exon 6:ExonStart false|TGMS

AAC CTT ATG TCC :Koz:weak:Cav:adequate:Exon 7:ExonStart false|NLMS***NonAcetSite**

TCT GCA ATG ACT :Koz:adequate:Cav:weak:Exon 8:ExonStart false|SAMT

AGG TCG ATG ATT :Koz:weak:Cav:weak:Exon 8:ExonStart false|RSMI

GAT CTG ATG ACC :Koz:weak:Cav:weak:Exon 8:ExonStart false|DLMT

GCT TCG ATG ATG :Koz:weak:Cav:weak:Exon 8:ExonStart false|ASMM

TCG ATG ATG GCC :Koz:strong:Cav:adequate:Exon 8:ExonStart false|SMMA

GCC ATC ATG CTG :Koz:adequate:Cav:strong:Exon 9:ExonStart false|AIML

CAG GCG ATG TAC :Koz:adequate:Cav:weak:Exon 10:ExonStart false|QAMT

TCC ACG ATG ATC :Koz:adequate:Cav:strong:Exon 10:ExonStart false|STMI

CG14998-PC

TGA AAT ATG GCG :Koz:strong:Cav:adequate:Exon 1:ExonStart false|stopNMA*****

AGC GCC ATG TCC :Koz:adequate:Cav:adequate:Exon 5:ExonStart false|SAMS

ACA AAC ATG TCC :Koz:adequate:Cav:adequate:Exon 5:ExonStart false|TNMS

ACG GGC ATG TCT :Koz:adequate:Cav:weak:Exon 5:ExonStart false|TGMS

AAC CTT ATG TCC :Koz:weak:Cav:adequate:Exon 6:ExonStart false|NLMS***NonAcetSite**

TCT GCA ATG ACT :Koz:adequate:Cav:weak:Exon 7:ExonStart false|SAMT

AGG TCG ATG ATT :Koz:weak:Cav:weak:Exon 7:ExonStart false|RSMI

GAT CTG ATG ACC :Koz:weak:Cav:weak:Exon 7:ExonStart false|DLMT

GCT TCG ATG ATG :Koz:weak:Cav:weak:Exon 7:ExonStart false|ASMM

TCG ATG ATG GCC :Koz:strong:Cav:adequate:Exon 7:ExonStart false|SMMA

GCC ATC ATG CTG :Koz:adequate:Cav:strong:Exon 8:ExonStart false|AIML

CAG GCG ATG TAC :Koz:adequate:Cav:weak:Exon 9:ExonStart false|QAMT

TCC ACG ATG ATC :Koz:adequate:Cav:strong:Exon 9:ExonStart false|STMI

CG14998-PD

TGA AAT ATG GCG :Koz:strong:Cav:adequate:Exon 1:ExonStart false|stopNMA*****

TCC TCC ATG TAC :Koz:weak:Cav:adequate:Exon 6:ExonStart false|SSMT

TCA CCC ATG GTG :Koz:adequate:Cav:weak:Exon 7:ExonStart false|SPMV

GAC CTT ATG CCG :Koz:weak:Cav:adequate:Exon 7:ExonStart false|DLMP

AGC GCC ATG TCC :Koz:adequate:Cav:adequate:Exon 8:ExonStart false|SAMS

ACA AAC ATG TCC :Koz:adequate:Cav:adequate:Exon 8:ExonStart false|TNMS

ACG GGC ATG TCT :Koz:adequate:Cav:weak:Exon 8:ExonStart false|TGMS

AAC CTT ATG TCC :Koz:weak:Cav:adequate:Exon 9:ExonStart false|NLMS***NonAcetSite**

TCT GCA ATG ACT :Koz:adequate:Cav:weak:Exon 10:ExonStart false|SAMT

AGG TCG ATG ATT :Koz:weak:Cav:weak:Exon 10:ExonStart false|RSMI

GAT CTG ATG ACC :Koz:weak:Cav:weak:Exon 10:ExonStart false|DLMT

GCT TCG ATG ATG :Koz:weak:Cav:weak:Exon 10:ExonStart false|ASMM

TCG ATG ATG GCC :Koz:strong:Cav:adequate:Exon 10:ExonStart false|SMMA

GCC ATC ATG CTG :Koz:adequate:Cav:strong:Exon 11:ExonStart false|AIML

CAG GCG ATG TAC :Koz:adequate:Cav:weak:Exon 12:ExonStart false|QAMT

TCC ACG ATG ATC :Koz:adequate:Cav:strong:Exon 12:ExonStart false|STMI

CG14998-PE

TGA AAT ATG GCG :Koz:strong:Cav:adequate:Exon 1:ExonStart false|stopNMA*****

TCA CCC ATG GTG :Koz:adequate:Cav:weak:Exon 6:ExonStart false|SPMV

GAC CTT ATG CCG :Koz:weak:Cav:adequate:Exon 6:ExonStart false|DLMP

AGC GCC ATG TCC :Koz:adequate:Cav:adequate:Exon 7:ExonStart false|SAMS

ACA AAC ATG TCC :Koz:adequate:Cav:adequate:Exon 7:ExonStart false|TNMS

ACG GGC ATG TCT :Koz:adequate:Cav:weak:Exon 7:ExonStart false|TGMS

AAC CTT ATG TCC :Koz:weak:Cav:adequate:Exon 8:ExonStart false|NLMS***NonAcetSite**

TCT GCA ATG ACT :Koz:adequate:Cav:weak:Exon 9:ExonStart false|SAMT

AGG TCG ATG ATT :Koz:weak:Cav:weak:Exon 9:ExonStart false|RSMI

GAT CTG ATG ACC :Koz:weak:Cav:weak:Exon 9:ExonStart false|DLMT

GCT TCG ATG ATG :Koz:weak:Cav:weak:Exon 9:ExonStart false|ASMM

TCG ATG ATG GCC :Koz:strong:Cav:adequate:Exon 9:ExonStart false|SMMA

GCC ATC ATG CTG :Koz:adequate:Cav:strong:Exon 10:ExonStart false|AIML

CAG GCG ATG TAC :Koz:adequate:Cav:weak:Exon 11:ExonStart false|QAMT

TCC ACG ATG ATC :Koz:adequate:Cav:strong:Exon 11:ExonStart false|STMI

CG15443-PA

GAT TCC ATG AAA :Koz:weak:Cav:weak:Exon 1:ExonStart false|DSMK

AAA CAA ATG GAC :Koz:adequate:Cav:weak:Exon 1:ExonStart false|KQMD***AcetSite**

CTT GAG ATG GGC :Koz:strong:Cav:weak:Exon 1:ExonStart false|LEMG

ATT GGA ATG GCC :Koz:strong:Cav:weak:Exon 1:ExonStart false|IGMA

AGC TAC ATG GTC :Koz:adequate:Cav:adequate:Exon 2:ExonStart false|STMV

CTG CAA ATG CAA :Koz:weak:Cav:weak:Exon 2:ExonStart false|LQMQ

TAC TTC ATG TAC :Koz:weak:Cav:adequate:Exon 2:ExonStart false|TFMT

CGT GTA ATG ACC :Koz:adequate:Cav:weak:Exon 2:ExonStart false|RVMT

GAC GCG ATG CGC :Koz:adequate:Cav:adequate:Exon 2:ExonStart false|DAMR

CG1633-PA

TCA AGG ATG CCC :Koz:adequate:Cav:adequate:Exon 1:ExonStart false|SRMP*****

GGC AGC ATG GAC :Koz:strong:Cav:strong:Exon 2:ExonStart false|GSMD

AAG TCG ATG AAG :Koz:weak:Cav:weak:Exon 2:ExonStart false|KSMK

AAG ACC ATG GTG :Koz:strong:Cav:adequate:Exon 2:ExonStart false|KTMV***NonAcetSite**

CG1633-PB

TTT AAA ATG CCC :Koz:adequate:Cav:adequate:Exon 1:ExonStart false|FKMP*****

GGC AGC ATG GAC :Koz:strong:Cav:strong:Exon 2:ExonStart false|GSMD

AAG TCG ATG AAG :Koz:weak:Cav:weak:Exon 2:ExonStart false|KSMK

AAG ACC ATG GTG :Koz:strong:Cav:adequate:Exon 2:ExonStart false|KTMV***NonAcetSite**

CG16912-PA

CGC ATA ATG CTG :Koz:adequate:Cav:strong:Exon 1:ExonStart false|RIML

CGC CAG ATG TCG :Koz:weak:Cav:adequate:Exon 1:ExonStart false|RQMS***NonAcetSite**

CCC AGG ATG AAA :Koz:adequate:Cav:strong:Exon 2:ExonStart false|PRMK

GTA ATA ATG GGC :Koz:strong:Cav:adequate:Exon 2:ExonStart false|VIMG

GCC AAC ATG GGA :Koz:strong:Cav:strong:Exon 3:ExonStart false|ANMG

TTT CGC ATG GGG :Koz:adequate:Cav:weak:Exon 3:ExonStart false|FRMG

GGG TCA ATG CTC :Koz:weak:Cav:weak:Exon 3:ExonStart false|GSML

GAC GGA ATG AGC :Koz:adequate:Cav:adequate:Exon 3:ExonStart false|DGMS

TTT CAA ATG GGA :Koz:adequate:Cav:weak:Exon 3:ExonStart false|FQMG

AAT TTA ATG ACT :Koz:weak:Cav:weak:Exon 3:ExonStart false|NLMT

CTG CGC ATG CCA :Koz:weak:Cav:weak:Exon 3:ExonStart false|LRMP

CAG TTG ATG CGG :Koz:weak:Cav:weak:Exon 3:ExonStart false|QLMR

GCT ACT ATG GTG :Koz:strong:Cav:adequate:Exon 4:ExonStart false|ATMV

CCA GGA ATG TCC :Koz:adequate:Cav:weak:Exon 4:ExonStart false|PGMS

CTC GCC ATG AAG :Koz:adequate:Cav:adequate:Exon 4:ExonStart false|LAMK

CG17019-PA

ACC ACG ATG CCC :Koz:adequate:Cav:strong:Exon 1:ExonStart false|TTMP

GAG ATT ATG GCC :Koz:strong:Cav:adequate:Exon 3:ExonStart false|EIMA

AGT GAG ATG GGC :Koz:strong:Cav:weak:Exon 3:ExonStart false|SEMG***AcetSite**

GAG AAA ATG CGA :Koz:adequate:Cav:adequate:Exon 3:ExonStart false|EKMR

TCG CAA ATG TCC :Koz:weak:Cav:weak:Exon 3:ExonStart false|SQMS

CCG GAC ATG CCC :Koz:adequate:Cav:weak:Exon 3:ExonStart false|PDMP

GTG CTG ATG CTG :Koz:weak:Cav:weak:Exon 4:ExonStart false|VLML

AAA ACC ATG CGC :Koz:adequate:Cav:adequate:Exon 4:ExonStart false|KTMR

ATC TGC ATG GAT :Koz:adequate:Cav:adequate:Exon 5:ExonStart false|ICMD

GGA CAC ATG GCC :Koz:adequate:Cav:weak:Exon 5:ExonStart false|GHMA

CG1721-PA

GCT AAA ATG GGC :Koz:strong:Cav:adequate:Exon 1:ExonStart false|AKMG*****

ATC GTG ATG GTG :Koz:strong:Cav:adequate:Exon 1:ExonStart false|IVMV

CCA CCG ATG GAG :Koz:adequate:Cav:weak:Exon 2:ExonStart false|PPME

CCC CAG ATG AAG :Koz:weak:Cav:adequate:Exon 2:ExonStart false|PQMK

GCC ATC ATG GCC :Koz:strong:Cav:strong:Exon 3:ExonStart false|AIMA

GTG TCC ATG CAG :Koz:weak:Cav:weak:Exon 3:ExonStart false|VSMQ***NonAcetSite**

CG17257-PA

AAA TCG ATG TTC :Koz:weak:Cav:weak:Exon 1:ExonStart false|KSMF

TTC TTG ATG ACA :Koz:weak:Cav:adequate:Exon 1:ExonStart false|FLMT

GAC GGC ATG CTC :Koz:adequate:Cav:adequate:Exon 2:ExonStart false|DGML***AcetSite**

CCC CTC ATG CTG :Koz:weak:Cav:adequate:Exon 2:ExonStart false|PLML

GTG CTG ATG CTC :Koz:weak:Cav:weak:Exon 2:ExonStart false|VLML

TGG GGC ATG GAG :Koz:strong:Cav:weak:Exon 2:ExonStart false|WGME

TTC TAC ATG ATC :Koz:weak:Cav:adequate:Exon 2:ExonStart false|FTMI

GCG TTA ATG TTC :Koz:weak:Cav:weak:Exon 2:ExonStart false|ALMF

CTC CAG ATG GAG :Koz:adequate:Cav:adequate:Exon 2:ExonStart false|LQME

GGG GAT ATG GAC :Koz:strong:Cav:weak:Exon 2:ExonStart false|GDMD

GGC TTG ATG CAC :Koz:weak:Cav:adequate:Exon 2:ExonStart false|GLMH

CCG TTT ATG GTG :Koz:adequate:Cav:weak:Exon 2:ExonStart false|PFMV

CG17257-PB

AAA TCG ATG TTC :Koz:weak:Cav:weak:Exon 2:ExonStart false|KSMF

TTC TTG ATG ACA :Koz:weak:Cav:adequate:Exon 2:ExonStart false|FLMT

GAC GGC ATG CTC :Koz:adequate:Cav:adequate:Exon 3:ExonStart false|DGML***AcetSite**

CCC CTC ATG CTG :Koz:weak:Cav:adequate:Exon 3:ExonStart false|PLML

GTG CTG ATG CTC :Koz:weak:Cav:weak:Exon 3:ExonStart false|VLML

TGG GGC ATG GAG :Koz:strong:Cav:weak:Exon 3:ExonStart false|WGME

TTC TAC ATG ATC :Koz:weak:Cav:adequate:Exon 3:ExonStart false|FTMI

GCG TTA ATG TTC :Koz:weak:Cav:weak:Exon 3:ExonStart false|ALMF

CTC CAG ATG GAG :Koz:adequate:Cav:adequate:Exon 3:ExonStart false|LQME

GGG GAT ATG GAC :Koz:strong:Cav:weak:Exon 3:ExonStart false|GDMD

GGC TTG ATG CAC :Koz:weak:Cav:adequate:Exon 3:ExonStart false|GLMH

CCG TTT ATG GTG :Koz:adequate:Cav:weak:Exon 3:ExonStart false|PFMV

CG1782-PA

GAA GCT ATG TCG :Koz:adequate:Cav:weak:Exon 1:ExonStart false|EAMS*****

CAA TTG ATG GCC :Koz:adequate:Cav:weak:Exon 1:ExonStart false|QLMA***AcetSite**

GGG GCA ATG GGA :Koz:strong:Cav:weak:Exon 1:ExonStart false|GAMG

ATG GGA ATG GAA :Koz:strong:Cav:weak:Exon 1:ExonStart false|MGME

AGC AAC ATG GCT :Koz:strong:Cav:strong:Exon 1:ExonStart false|SNMA***AcetSite**

GAT GCG ATG CGT :Koz:adequate:Cav:weak:Exon 1:ExonStart false|DAMR

CGT CGG ATG GCC :Koz:adequate:Cav:weak:Exon 1:ExonStart false|RRMA

TCC ACC ATG ATA :Koz:adequate:Cav:strong:Exon 2:ExonStart false|STMI

CAG GGC ATG CAG :Koz:adequate:Cav:weak:Exon 2:ExonStart false|QGMQ

GTG AAG ATG CCC :Koz:adequate:Cav:adequate:Exon 2:ExonStart false|VKMP

TTT GGA ATG CTG :Koz:adequate:Cav:weak:Exon 2:ExonStart false|FGML

ACA GAC ATG GAT :Koz:strong:Cav:weak:Exon 2:ExonStart false|TDMD

AAA TCA ATG ACG :Koz:weak:Cav:weak:Exon 2:ExonStart false|KSMT

AAA CGG ATG AAT :Koz:weak:Cav:weak:Exon 2:ExonStart false|KRMN

ATA TAT ATG GAT :Koz:adequate:Cav:weak:Exon 2:ExonStart false|ITMD

GAT CCC ATG CAC :Koz:weak:Cav:weak:Exon 3:ExonStart false|DPMH

TTG CAC ATG GAC :Koz:adequate:Cav:weak:Exon 4:ExonStart false|LHMD

CCG TTC ATG GCC :Koz:adequate:Cav:weak:Exon 4:ExonStart false|PFMA

ATC ACC ATG CTC :Koz:adequate:Cav:strong:Exon 4:ExonStart false|ITML

GTG TCC ATG CTG :Koz:weak:Cav:weak:Exon 4:ExonStart false|VSML

TTC TTC ATG CCC :Koz:weak:Cav:adequate:Exon 4:ExonStart false|FFMP

TTG CCG ATG TCG :Koz:weak:Cav:weak:Exon 4:ExonStart false|LPMS

CG17949-PA

TGA ACA ATG CCT :Koz:adequate:Cav:adequate:Exon 1:ExonStart false|stopTMP

AAG GCG ATG AGC :Koz:adequate:Cav:weak:Exon 1:ExonStart false|KAMS***NonAcetSite**

AGC ATA ATG AAC :Koz:adequate:Cav:strong:Exon 1:ExonStart false|SIMN

CG18290-PA

GCC AAG ATG TGT :Koz:adequate:Cav:strong:Exon 2:ExonStart false|AKMC

TCC GGA ATG TGC :Koz:adequate:Cav:adequate:Exon 2:ExonStart false|SGMC***NonAcetSite**

GGC GTA ATG GTG :Koz:strong:Cav:adequate:Exon 2:ExonStart false|GVMV

GTG GGC ATG GGA :Koz:strong:Cav:weak:Exon 2:ExonStart false|VGMG***NonAcetSite**

GAC GAT ATG GAG :Koz:strong:Cav:adequate:Exon 2:ExonStart false|DDME

GAG AAG ATG ACC :Koz:adequate:Cav:adequate:Exon 2:ExonStart false|EKMT***AcetSite**

CAG ATC ATG TTC :Koz:adequate:Cav:adequate:Exon 2:ExonStart false|QIMF

CCC GCC ATG TAT :Koz:adequate:Cav:adequate:Exon 2:ExonStart false|PAMT

TAC CTG ATG AAG :Koz:weak:Cav:adequate:Exon 2:ExonStart false|TLMK

CAG GAG ATG GCC :Koz:strong:Cav:weak:Exon 2:ExonStart false|QEMA

CTG GGA ATG GAA :Koz:strong:Cav:weak:Exon 2:ExonStart false|LGME

TCG ATC ATG AAG :Koz:adequate:Cav:adequate:Exon 2:ExonStart false|SIMK

ATC GTC ATG TCG :Koz:adequate:Cav:adequate:Exon 2:ExonStart false|IVMS***NonAcetSite**

ACC ACC ATG TAC :Koz:adequate:Cav:strong:Exon 2:ExonStart false|TTMT

GAT CGT ATG CAG :Koz:weak:Cav:weak:Exon 2:ExonStart false|DRMQ

CAG CAG ATG TGG :Koz:weak:Cav:weak:Exon 2:ExonStart false|QQMW

CG18290-PB

GCC AAG ATG TGT :Koz:adequate:Cav:strong:Exon 1:ExonStart false|AKMC

TCC GGA ATG TGC :Koz:adequate:Cav:adequate:Exon 1:ExonStart false|SGMC***NonAcetSite**

GGC GTA ATG GTG :Koz:strong:Cav:adequate:Exon 1:ExonStart false|GVMV

GTG GGC ATG GGA :Koz:strong:Cav:weak:Exon 1:ExonStart false|VGMG***NonAcetSite**

GAC GAT ATG GAG :Koz:strong:Cav:adequate:Exon 1:ExonStart false|DDME

GAG AAG ATG ACC :Koz:adequate:Cav:adequate:Exon 1:ExonStart false|EKMT***AcetSite**

CAG ATC ATG TTC :Koz:adequate:Cav:adequate:Exon 1:ExonStart false|QIMF

CCC GCC ATG TAT :Koz:adequate:Cav:adequate:Exon 1:ExonStart false|PAMT

TAC CTG ATG AAG :Koz:weak:Cav:adequate:Exon 1:ExonStart false|TLMK

CAG GAG ATG GCC :Koz:strong:Cav:weak:Exon 1:ExonStart false|QEMA

CTG GGA ATG GAA :Koz:strong:Cav:weak:Exon 1:ExonStart false|LGME

TCG ATC ATG AAG :Koz:adequate:Cav:adequate:Exon 1:ExonStart false|SIMK

ATC GTC ATG TCG :Koz:adequate:Cav:adequate:Exon 1:ExonStart false|IVMS***NonAcetSite**

ACC ACC ATG TAC :Koz:adequate:Cav:strong:Exon 1:ExonStart false|TTMT

GAT CGT ATG CAG :Koz:weak:Cav:weak:Exon 1:ExonStart false|DRMQ

CAG CAG ATG TGG :Koz:weak:Cav:weak:Exon 1:ExonStart false|QQMW

CG18497-PA

CTG CGA ATG TTC :Koz:weak:Cav:weak:Exon 2:ExonStart false|LRMF

CGA AAC ATG GTA :Koz:strong:Cav:adequate:Exon 2:ExonStart false|RNMV

TTC ACA ATG AGC :Koz:adequate:Cav:strong:Exon 3:ExonStart false|FTMS

CGA AAT ATG CTT :Koz:adequate:Cav:adequate:Exon 3:ExonStart false|RNML

CCA ACA ATG CCC :Koz:adequate:Cav:adequate:Exon 3:ExonStart false|PTMP

TGT GCG ATG GCA :Koz:strong:Cav:weak:Exon 3:ExonStart false|CAMA

AAG GCA ATG CGC :Koz:adequate:Cav:weak:Exon 4:ExonStart false|KAMR

CGC AAA ATG GAC :Koz:strong:Cav:strong:Exon 4:ExonStart false|RKMD

AAA TCC ATG CCC :Koz:weak:Cav:weak:Exon 4:ExonStart false|KSMP

AAG GAT ATG CGT :Koz:adequate:Cav:weak:Exon 4:ExonStart false|KDMR

AGT GCG ATG CCA :Koz:adequate:Cav:weak:Exon 5:ExonStart false|SAMP

ACA TCC ATG CCC :Koz:weak:Cav:weak:Exon 5:ExonStart false|TSMP

TTG CCT ATG TCT :Koz:weak:Cav:weak:Exon 5:ExonStart false|LPMS

CAT CGA ATG GTT :Koz:adequate:Cav:weak:Exon 5:ExonStart false|HRMV

ATC AAA ATG GAT :Koz:strong:Cav:strong:Exon 5:ExonStart false|IKMD

TCC CTG ATG CTC :Koz:weak:Cav:adequate:Exon 6:ExonStart false|SLML

AGC AGC ATG GAT :Koz:strong:Cav:strong:Exon 6:ExonStart false|SSMD

CAG CAC ATG ATG :Koz:weak:Cav:weak:Exon 6:ExonStart false|QHMM

CAC ATG ATG AAT :Koz:adequate:Cav:strong:Exon 6:ExonStart false|HMMN***AcetSite**

ACT ACT ATG CAA :Koz:adequate:Cav:adequate:Exon 6:ExonStart false|TTMQ

GAT GTG ATG TGG :Koz:adequate:Cav:weak:Exon 6:ExonStart false|DVMW

CAG GGA ATG GGC :Koz:strong:Cav:weak:Exon 6:ExonStart false|QGMG

TGC TTG ATG GCC :Koz:adequate:Cav:adequate:Exon 6:ExonStart false|CLMA

CTC TCT ATG CAC :Koz:weak:Cav:adequate:Exon 6:ExonStart false|LSMH

ATG CAC ATG AAT :Koz:weak:Cav:weak:Exon 6:ExonStart false|MHMN

TCA CCC ATG AAT :Koz:weak:Cav:weak:Exon 6:ExonStart false|SPMN

GCT TCG ATG TCG :Koz:weak:Cav:weak:Exon 6:ExonStart false|ASMS

CCA GAA ATG GGA :Koz:strong:Cav:weak:Exon 6:ExonStart false|PEMG

AAG GAA ATG CGT :Koz:adequate:Cav:weak:Exon 6:ExonStart false|KEMR

CGA GCT ATG GAT :Koz:strong:Cav:weak:Exon 6:ExonStart false|RAMD

GGA AGG ATG CGT :Koz:adequate:Cav:adequate:Exon 6:ExonStart false|GRMR

AGC AAA ATG GAT :Koz:strong:Cav:strong:Exon 6:ExonStart false|SKMD

AAT GCC ATG GAC :Koz:strong:Cav:weak:Exon 6:ExonStart false|NAMD

ATA AGA ATG GAG :Koz:strong:Cav:adequate:Exon 6:ExonStart false|IRME

AAA TTG ATG ATG :Koz:weak:Cav:weak:Exon 6:ExonStart false|KLMM

TTG ATG ATG TTG :Koz:adequate:Cav:adequate:Exon 6:ExonStart false|LMML

GTA TCT ATG TAC :Koz:weak:Cav:weak:Exon 6:ExonStart false|VSMT

AAG AAT ATG CAA :Koz:adequate:Cav:adequate:Exon 6:ExonStart false|KNMQ

CGT CAG ATG CAA :Koz:weak:Cav:weak:Exon 6:ExonStart false|RQMQ

AAT GTG ATG AAG :Koz:adequate:Cav:weak:Exon 6:ExonStart false|NVMK

TTG GTG ATG ACC :Koz:adequate:Cav:weak:Exon 6:ExonStart false|LVMT

AGA AAT ATG ACG :Koz:adequate:Cav:adequate:Exon 6:ExonStart false|RNMT

AAG AGC ATG GAT :Koz:strong:Cav:adequate:Exon 6:ExonStart false|KSMD

GAT AAA ATG GAG :Koz:strong:Cav:adequate:Exon 6:ExonStart false|DKME

GTG GAT ATG GAT :Koz:strong:Cav:weak:Exon 6:ExonStart false|VDMD

CAA CTA ATG AGT :Koz:weak:Cav:weak:Exon 6:ExonStart false|QLMS

TCG GAT ATG ACA :Koz:adequate:Cav:weak:Exon 6:ExonStart false|SDMT

GTG GAT ATG ACT :Koz:adequate:Cav:weak:Exon 6:ExonStart false|VDMT

GAT GGC ATG GAT :Koz:strong:Cav:weak:Exon 6:ExonStart false|DGMD

GAT GAA ATG AGC :Koz:adequate:Cav:weak:Exon 6:ExonStart false|DEMS

CCA GAA ATG GAA :Koz:strong:Cav:weak:Exon 6:ExonStart false|PEME

GAG GAT ATG ATG :Koz:adequate:Cav:weak:Exon 6:ExonStart false|EDMM

GAT ATG ATG GAC :Koz:strong:Cav:adequate:Exon 6:ExonStart false|DMMD

AAT GCC ATG CCC :Koz:adequate:Cav:weak:Exon 6:ExonStart false|NAMP

TTG ATA ATG CAA :Koz:adequate:Cav:adequate:Exon 6:ExonStart false|LIMQ

CCC CAA ATG GTG :Koz:adequate:Cav:adequate:Exon 6:ExonStart false|PQMV

ACT TAT ATG GTT :Koz:adequate:Cav:weak:Exon 6:ExonStart false|TTMV

CGT CCA ATG GTA :Koz:adequate:Cav:weak:Exon 6:ExonStart false|RPMV

CAG TTA ATG ACT :Koz:weak:Cav:weak:Exon 6:ExonStart false|QLMT

CAA AAA ATG ACA :Koz:adequate:Cav:adequate:Exon 6:ExonStart false|QKMT

GCC AAG ATG ACT :Koz:adequate:Cav:strong:Exon 6:ExonStart false|AKMT

CAG CAC ATG CAG :Koz:weak:Cav:weak:Exon 6:ExonStart false|QHMQ

CAG TTC ATG CAT :Koz:weak:Cav:weak:Exon 6:ExonStart false|QFMH

CAG CAA ATG ATA :Koz:weak:Cav:weak:Exon 6:ExonStart false|QQMI

CAA CAC ATG CAG :Koz:weak:Cav:weak:Exon 6:ExonStart false|QHMQ

CAC CAA ATG CAT :Koz:weak:Cav:adequate:Exon 6:ExonStart false|HQMH

CAG CAA ATG CAT :Koz:weak:Cav:weak:Exon 6:ExonStart false|QQMH

CCA GCT ATG TCC :Koz:adequate:Cav:weak:Exon 6:ExonStart false|PAMS

GTG GAA ATG CAG :Koz:adequate:Cav:weak:Exon 6:ExonStart false|VEMQ

CAG TCT ATG GCC :Koz:adequate:Cav:weak:Exon 6:ExonStart false|QSMA

ACT TCA ATG AGT :Koz:weak:Cav:weak:Exon 6:ExonStart false|TSMS

AAG GGA ATG GAA :Koz:strong:Cav:weak:Exon 6:ExonStart false|KGME

ATG GAA ATG GTG :Koz:strong:Cav:weak:Exon 6:ExonStart false|MEMV

AGT GAA ATG GAG :Koz:strong:Cav:weak:Exon 6:ExonStart false|SEME

CCA GCC ATG GTC :Koz:strong:Cav:weak:Exon 6:ExonStart false|PAMV

CCT ACA ATG CCT :Koz:adequate:Cav:adequate:Exon 6:ExonStart false|PTMP

CTT CCG ATG CAT :Koz:weak:Cav:weak:Exon 6:ExonStart false|LPMH

GCT GGA ATG CCC :Koz:adequate:Cav:weak:Exon 6:ExonStart false|AGMP

GGT CAC ATG TTG :Koz:weak:Cav:weak:Exon 6:ExonStart false|GHML

ATA CAA ATG CCT :Koz:weak:Cav:weak:Exon 6:ExonStart false|IQMP

ATA ATT ATG CCT :Koz:adequate:Cav:adequate:Exon 6:ExonStart false|IIMP

CCT GGC ATG CTA :Koz:adequate:Cav:weak:Exon 6:ExonStart false|PGML

CAG ATT ATG CCA :Koz:adequate:Cav:adequate:Exon 6:ExonStart false|QIMP

GAT TTT ATG CTC :Koz:weak:Cav:weak:Exon 9:ExonStart false|DFML

CAT TTA ATG CGA :Koz:weak:Cav:weak:Exon 9:ExonStart false|HLMR

GAC AAG ATG GGC :Koz:strong:Cav:strong:Exon 9:ExonStart false|DKMG

CCA CAA ATG GCT :Koz:adequate:Cav:weak:Exon 10:ExonStart false|PQMA

CAA CAA ATG CCT :Koz:weak:Cav:weak:Exon 10:ExonStart false|QQMP

AAG GCA ATG GAT :Koz:strong:Cav:weak:Exon 10:ExonStart false|KAMD

ATG GAT ATG CAG :Koz:adequate:Cav:weak:Exon 10:ExonStart false|MDMQ

GAT GAG ATG GAC :Koz:strong:Cav:weak:Exon 10:ExonStart false|DEMD

GAC CGC ATG TCA :Koz:weak:Cav:adequate:Exon 10:ExonStart false|DRMS

ATG TCA ATG ATT :Koz:weak:Cav:weak:Exon 10:ExonStart false|MSMI

GCA GGC ATG GAA :Koz:strong:Cav:weak:Exon 11:ExonStart false|AGME

CCA GCG ATG GCA :Koz:strong:Cav:weak:Exon 11:ExonStart false|PAMA

CCG GTA ATG TGG :Koz:adequate:Cav:weak:Exon 11:ExonStart false|PVMW

GTC CAA ATG CAC :Koz:weak:Cav:adequate:Exon 11:ExonStart false|VQMH

CAA AGA ATG CGA :Koz:adequate:Cav:adequate:Exon 11:ExonStart false|QRMR

AAG AAG ATG CAG :Koz:adequate:Cav:adequate:Exon 11:ExonStart false|KKMQ

CAT TGC ATG CTT :Koz:weak:Cav:weak:Exon 12:ExonStart false|HCML

CAG AAA ATG GCA :Koz:strong:Cav:adequate:Exon 12:ExonStart false|QKMA

CG18497-PB

CTG CGA ATG TTC :Koz:weak:Cav:weak:Exon 2:ExonStart false|LRMF

CGA AAC ATG GTA :Koz:strong:Cav:adequate:Exon 2:ExonStart false|RNMV

TTC ACA ATG AGC :Koz:adequate:Cav:strong:Exon 3:ExonStart false|FTMS

CGA AAT ATG CTT :Koz:adequate:Cav:adequate:Exon 3:ExonStart false|RNML

CCA ACA ATG CCC :Koz:adequate:Cav:adequate:Exon 3:ExonStart false|PTMP

TGT GCG ATG GCA :Koz:strong:Cav:weak:Exon 3:ExonStart false|CAMA

AAG GCA ATG CGC :Koz:adequate:Cav:weak:Exon 4:ExonStart false|KAMR

CGC AAA ATG GAC :Koz:strong:Cav:strong:Exon 4:ExonStart false|RKMD

AAA TCC ATG CCC :Koz:weak:Cav:weak:Exon 4:ExonStart false|KSMP

AAG GAT ATG CGT :Koz:adequate:Cav:weak:Exon 4:ExonStart false|KDMR

AGT GCG ATG CCA :Koz:adequate:Cav:weak:Exon 5:ExonStart false|SAMP

ACA TCC ATG CCC :Koz:weak:Cav:weak:Exon 5:ExonStart false|TSMP

TTG CCT ATG TCT :Koz:weak:Cav:weak:Exon 5:ExonStart false|LPMS

CAT CGA ATG GTT :Koz:adequate:Cav:weak:Exon 5:ExonStart false|HRMV

ATC AAA ATG GAT :Koz:strong:Cav:strong:Exon 5:ExonStart false|IKMD

TCC CTG ATG CTC :Koz:weak:Cav:adequate:Exon 6:ExonStart false|SLML

AGC AGC ATG GAT :Koz:strong:Cav:strong:Exon 6:ExonStart false|SSMD

CAG CAC ATG ATG :Koz:weak:Cav:weak:Exon 6:ExonStart false|QHMM

CAC ATG ATG AAT :Koz:adequate:Cav:strong:Exon 6:ExonStart false|HMMN***AcetSite**

ACT ACT ATG CAA :Koz:adequate:Cav:adequate:Exon 6:ExonStart false|TTMQ

GAT GTG ATG TGG :Koz:adequate:Cav:weak:Exon 6:ExonStart false|DVMW

CAG GGA ATG GGC :Koz:strong:Cav:weak:Exon 6:ExonStart false|QGMG

TGC TTG ATG GCC :Koz:adequate:Cav:adequate:Exon 6:ExonStart false|CLMA

CTC TCT ATG CAC :Koz:weak:Cav:adequate:Exon 6:ExonStart false|LSMH

ATG CAC ATG AAT :Koz:weak:Cav:weak:Exon 6:ExonStart false|MHMN

TCA CCC ATG AAT :Koz:weak:Cav:weak:Exon 6:ExonStart false|SPMN

GCT TCG ATG TCG :Koz:weak:Cav:weak:Exon 6:ExonStart false|ASMS

CCA GAA ATG GGA :Koz:strong:Cav:weak:Exon 6:ExonStart false|PEMG

AAG GAA ATG CGT :Koz:adequate:Cav:weak:Exon 6:ExonStart false|KEMR

CGA GCT ATG GAT :Koz:strong:Cav:weak:Exon 6:ExonStart false|RAMD

GGA AGG ATG CGT :Koz:adequate:Cav:adequate:Exon 6:ExonStart false|GRMR

AGC AAA ATG GAT :Koz:strong:Cav:strong:Exon 6:ExonStart false|SKMD

AAT GCC ATG GAC :Koz:strong:Cav:weak:Exon 6:ExonStart false|NAMD

ATA AGA ATG GAG :Koz:strong:Cav:adequate:Exon 6:ExonStart false|IRME

AAA TTG ATG ATG :Koz:weak:Cav:weak:Exon 6:ExonStart false|KLMM

TTG ATG ATG TTG :Koz:adequate:Cav:adequate:Exon 6:ExonStart false|LMML

GTA TCT ATG TAC :Koz:weak:Cav:weak:Exon 6:ExonStart false|VSMT

AAG AAT ATG CAA :Koz:adequate:Cav:adequate:Exon 6:ExonStart false|KNMQ

CGT CAG ATG CAA :Koz:weak:Cav:weak:Exon 6:ExonStart false|RQMQ

AAT GTG ATG AAG :Koz:adequate:Cav:weak:Exon 6:ExonStart false|NVMK

TTG GTG ATG ACC :Koz:adequate:Cav:weak:Exon 6:ExonStart false|LVMT

AGA AAT ATG ACG :Koz:adequate:Cav:adequate:Exon 6:ExonStart false|RNMT

AAG AGC ATG GAT :Koz:strong:Cav:adequate:Exon 6:ExonStart false|KSMD

GAT AAA ATG GAG :Koz:strong:Cav:adequate:Exon 6:ExonStart false|DKME

GTG GAT ATG GAT :Koz:strong:Cav:weak:Exon 6:ExonStart false|VDMD

CAA CTA ATG AGT :Koz:weak:Cav:weak:Exon 6:ExonStart false|QLMS

TCG GAT ATG ACA :Koz:adequate:Cav:weak:Exon 6:ExonStart false|SDMT

GTG GAT ATG ACT :Koz:adequate:Cav:weak:Exon 6:ExonStart false|VDMT

GAT GGC ATG GAT :Koz:strong:Cav:weak:Exon 6:ExonStart false|DGMD

GAT GAA ATG AGC :Koz:adequate:Cav:weak:Exon 6:ExonStart false|DEMS

CCA GAA ATG GAA :Koz:strong:Cav:weak:Exon 6:ExonStart false|PEME

GAG GAT ATG ATG :Koz:adequate:Cav:weak:Exon 6:ExonStart false|EDMM

GAT ATG ATG GAC :Koz:strong:Cav:adequate:Exon 6:ExonStart false|DMMD

AAT GCC ATG CCC :Koz:adequate:Cav:weak:Exon 6:ExonStart false|NAMP

TTG ATA ATG CAA :Koz:adequate:Cav:adequate:Exon 6:ExonStart false|LIMQ

CCC CAA ATG GTG :Koz:adequate:Cav:adequate:Exon 6:ExonStart false|PQMV

ACT TAT ATG GTT :Koz:adequate:Cav:weak:Exon 6:ExonStart false|TTMV

CGT CCA ATG GTA :Koz:adequate:Cav:weak:Exon 6:ExonStart false|RPMV

CAG TTA ATG ACT :Koz:weak:Cav:weak:Exon 6:ExonStart false|QLMT

CAA AAA ATG ACA :Koz:adequate:Cav:adequate:Exon 6:ExonStart false|QKMT

GCC AAG ATG ACT :Koz:adequate:Cav:strong:Exon 6:ExonStart false|AKMT

CAG CAC ATG CAG :Koz:weak:Cav:weak:Exon 6:ExonStart false|QHMQ

CAG TTC ATG CAT :Koz:weak:Cav:weak:Exon 6:ExonStart false|QFMH

CAG CAA ATG ATA :Koz:weak:Cav:weak:Exon 6:ExonStart false|QQMI

CAA CAC ATG CAG :Koz:weak:Cav:weak:Exon 6:ExonStart false|QHMQ

CAC CAA ATG CAT :Koz:weak:Cav:adequate:Exon 6:ExonStart false|HQMH

CAG CAA ATG CAT :Koz:weak:Cav:weak:Exon 6:ExonStart false|QQMH

CCA GCT ATG TCC :Koz:adequate:Cav:weak:Exon 6:ExonStart false|PAMS

GTG GAA ATG CAG :Koz:adequate:Cav:weak:Exon 6:ExonStart false|VEMQ

CAG TCT ATG GCC :Koz:adequate:Cav:weak:Exon 6:ExonStart false|QSMA

ACT TCA ATG AGT :Koz:weak:Cav:weak:Exon 6:ExonStart false|TSMS

AAG GGA ATG GAA :Koz:strong:Cav:weak:Exon 6:ExonStart false|KGME

ATG GAA ATG GTG :Koz:strong:Cav:weak:Exon 6:ExonStart false|MEMV

AGT GAA ATG GAG :Koz:strong:Cav:weak:Exon 6:ExonStart false|SEME

CCA GCC ATG GTC :Koz:strong:Cav:weak:Exon 6:ExonStart false|PAMV

CCT ACA ATG CCT :Koz:adequate:Cav:adequate:Exon 6:ExonStart false|PTMP

CTT CCG ATG CAT :Koz:weak:Cav:weak:Exon 6:ExonStart false|LPMH

GCT GGA ATG CCC :Koz:adequate:Cav:weak:Exon 6:ExonStart false|AGMP

GGT CAC ATG TTG :Koz:weak:Cav:weak:Exon 6:ExonStart false|GHML

ATA CAA ATG CCT :Koz:weak:Cav:weak:Exon 6:ExonStart false|IQMP

ATA ATT ATG CCT :Koz:adequate:Cav:adequate:Exon 6:ExonStart false|IIMP

CCT GGC ATG CTA :Koz:adequate:Cav:weak:Exon 6:ExonStart false|PGML

CAG ATT ATG CCA :Koz:adequate:Cav:adequate:Exon 6:ExonStart false|QIMP

GAT TTT ATG CTC :Koz:weak:Cav:weak:Exon 8:ExonStart false|DFML

CAT TTA ATG CGA :Koz:weak:Cav:weak:Exon 8:ExonStart false|HLMR

GAC AAG ATG GGC :Koz:strong:Cav:strong:Exon 8:ExonStart false|DKMG

CCA CAA ATG GCT :Koz:adequate:Cav:weak:Exon 9:ExonStart false|PQMA

CAA CAA ATG CCT :Koz:weak:Cav:weak:Exon 9:ExonStart false|QQMP

AAG GCA ATG GAT :Koz:strong:Cav:weak:Exon 9:ExonStart false|KAMD

ATG GAT ATG CAG :Koz:adequate:Cav:weak:Exon 9:ExonStart false|MDMQ

GAT GAG ATG GAC :Koz:strong:Cav:weak:Exon 9:ExonStart false|DEMD

GAC CGC ATG TCA :Koz:weak:Cav:adequate:Exon 9:ExonStart false|DRMS

ATG TCA ATG ATT :Koz:weak:Cav:weak:Exon 9:ExonStart false|MSMI

GCA GGC ATG GAA :Koz:strong:Cav:weak:Exon 10:ExonStart false|AGME

CCA GCG ATG GCA :Koz:strong:Cav:weak:Exon 10:ExonStart false|PAMA

CCG GTA ATG TGG :Koz:adequate:Cav:weak:Exon 10:ExonStart false|PVMW

GTC CAA ATG CAC :Koz:weak:Cav:adequate:Exon 10:ExonStart false|VQMH

CAA AGA ATG CGA :Koz:adequate:Cav:adequate:Exon 10:ExonStart false|QRMR

AAG AAG ATG CAG :Koz:adequate:Cav:adequate:Exon 10:ExonStart false|KKMQ

CAT TGC ATG CTT :Koz:weak:Cav:weak:Exon 11:ExonStart false|HCML

CAG AAA ATG GCA :Koz:strong:Cav:adequate:Exon 11:ExonStart false|QKMA

CG18497-PC

AAT AAT ATG CGA :Koz:adequate:Cav:adequate:Exon 1:ExonStart false|NNMR

TTC ACA ATG AGC :Koz:adequate:Cav:strong:Exon 2:ExonStart false|FTMS

CGA AAT ATG CTT :Koz:adequate:Cav:adequate:Exon 2:ExonStart false|RNML

CCA ACA ATG CCC :Koz:adequate:Cav:adequate:Exon 2:ExonStart false|PTMP

TGT GCG ATG GCA :Koz:strong:Cav:weak:Exon 2:ExonStart false|CAMA

AAG GCA ATG CGC :Koz:adequate:Cav:weak:Exon 3:ExonStart false|KAMR

CGC AAA ATG GAC :Koz:strong:Cav:strong:Exon 3:ExonStart false|RKMD

AAA TCC ATG CCC :Koz:weak:Cav:weak:Exon 3:ExonStart false|KSMP

AAG GAT ATG CGT :Koz:adequate:Cav:weak:Exon 3:ExonStart false|KDMR

AGT GCG ATG CCA :Koz:adequate:Cav:weak:Exon 4:ExonStart false|SAMP

ACA TCC ATG CCC :Koz:weak:Cav:weak:Exon 4:ExonStart false|TSMP

TTG CCT ATG TCT :Koz:weak:Cav:weak:Exon 4:ExonStart false|LPMS

CAT CGA ATG GTT :Koz:adequate:Cav:weak:Exon 4:ExonStart false|HRMV

ATC AAA ATG GAT :Koz:strong:Cav:strong:Exon 4:ExonStart false|IKMD

TCC CTG ATG CTC :Koz:weak:Cav:adequate:Exon 5:ExonStart false|SLML

AGC AGC ATG GAT :Koz:strong:Cav:strong:Exon 5:ExonStart false|SSMD

CAG CAC ATG ATG :Koz:weak:Cav:weak:Exon 5:ExonStart false|QHMM

CAC ATG ATG AAT :Koz:adequate:Cav:strong:Exon 5:ExonStart false|HMMN***AcetSite**

ACT ACT ATG CAA :Koz:adequate:Cav:adequate:Exon 5:ExonStart false|TTMQ

GAT GTG ATG TGG :Koz:adequate:Cav:weak:Exon 5:ExonStart false|DVMW

CAG GGA ATG GGC :Koz:strong:Cav:weak:Exon 5:ExonStart false|QGMG

TGC TTG ATG GCC :Koz:adequate:Cav:adequate:Exon 5:ExonStart false|CLMA

CTC TCT ATG CAC :Koz:weak:Cav:adequate:Exon 5:ExonStart false|LSMH

ATG CAC ATG AAT :Koz:weak:Cav:weak:Exon 5:ExonStart false|MHMN

TCA CCC ATG AAT :Koz:weak:Cav:weak:Exon 5:ExonStart false|SPMN

GCT TCG ATG TCG :Koz:weak:Cav:weak:Exon 5:ExonStart false|ASMS

CCA GAA ATG GGA :Koz:strong:Cav:weak:Exon 5:ExonStart false|PEMG

AAG GAA ATG CGT :Koz:adequate:Cav:weak:Exon 5:ExonStart false|KEMR

CGA GCT ATG GAT :Koz:strong:Cav:weak:Exon 5:ExonStart false|RAMD

GGA AGG ATG CGT :Koz:adequate:Cav:adequate:Exon 5:ExonStart false|GRMR

AGC AAA ATG GAT :Koz:strong:Cav:strong:Exon 5:ExonStart false|SKMD

AAT GCC ATG GAC :Koz:strong:Cav:weak:Exon 5:ExonStart false|NAMD

ATA AGA ATG GAG :Koz:strong:Cav:adequate:Exon 5:ExonStart false|IRME

AAA TTG ATG ATG :Koz:weak:Cav:weak:Exon 5:ExonStart false|KLMM

TTG ATG ATG TTG :Koz:adequate:Cav:adequate:Exon 5:ExonStart false|LMML

GTA TCT ATG TAC :Koz:weak:Cav:weak:Exon 5:ExonStart false|VSMT

AAG AAT ATG CAA :Koz:adequate:Cav:adequate:Exon 5:ExonStart false|KNMQ

CGT CAG ATG CAA :Koz:weak:Cav:weak:Exon 5:ExonStart false|RQMQ

AAT GTG ATG AAG :Koz:adequate:Cav:weak:Exon 5:ExonStart false|NVMK

TTG GTG ATG ACC :Koz:adequate:Cav:weak:Exon 5:ExonStart false|LVMT

AGA AAT ATG ACG :Koz:adequate:Cav:adequate:Exon 5:ExonStart false|RNMT

AAG AGC ATG GAT :Koz:strong:Cav:adequate:Exon 5:ExonStart false|KSMD

GAT AAA ATG GAG :Koz:strong:Cav:adequate:Exon 5:ExonStart false|DKME

GTG GAT ATG GAT :Koz:strong:Cav:weak:Exon 5:ExonStart false|VDMD

CAA CTA ATG AGT :Koz:weak:Cav:weak:Exon 5:ExonStart false|QLMS

TCG GAT ATG ACA :Koz:adequate:Cav:weak:Exon 5:ExonStart false|SDMT

GTG GAT ATG ACT :Koz:adequate:Cav:weak:Exon 5:ExonStart false|VDMT

GAT GGC ATG GAT :Koz:strong:Cav:weak:Exon 5:ExonStart false|DGMD

GAT GAA ATG AGC :Koz:adequate:Cav:weak:Exon 5:ExonStart false|DEMS

CCA GAA ATG GAA :Koz:strong:Cav:weak:Exon 5:ExonStart false|PEME

GAG GAT ATG ATG :Koz:adequate:Cav:weak:Exon 5:ExonStart false|EDMM

GAT ATG ATG GAC :Koz:strong:Cav:adequate:Exon 5:ExonStart false|DMMD

AAT GCC ATG CCC :Koz:adequate:Cav:weak:Exon 5:ExonStart false|NAMP

TTG ATA ATG CAA :Koz:adequate:Cav:adequate:Exon 5:ExonStart false|LIMQ

CCC CAA ATG GTG :Koz:adequate:Cav:adequate:Exon 5:ExonStart false|PQMV

ACT TAT ATG GTT :Koz:adequate:Cav:weak:Exon 5:ExonStart false|TTMV

CGT CCA ATG GTA :Koz:adequate:Cav:weak:Exon 5:ExonStart false|RPMV

CAG TTA ATG ACT :Koz:weak:Cav:weak:Exon 5:ExonStart false|QLMT

CAA AAA ATG ACA :Koz:adequate:Cav:adequate:Exon 5:ExonStart false|QKMT

GCC AAG ATG ACT :Koz:adequate:Cav:strong:Exon 5:ExonStart false|AKMT

CAG CAC ATG CAG :Koz:weak:Cav:weak:Exon 5:ExonStart false|QHMQ

CAG TTC ATG CAT :Koz:weak:Cav:weak:Exon 5:ExonStart false|QFMH

CAG CAA ATG ATA :Koz:weak:Cav:weak:Exon 5:ExonStart false|QQMI

CAA CAC ATG CAG :Koz:weak:Cav:weak:Exon 5:ExonStart false|QHMQ

CAC CAA ATG CAT :Koz:weak:Cav:adequate:Exon 5:ExonStart false|HQMH

CAG CAA ATG CAT :Koz:weak:Cav:weak:Exon 5:ExonStart false|QQMH

CCA GCT ATG TCC :Koz:adequate:Cav:weak:Exon 5:ExonStart false|PAMS

GTG GAA ATG CAG :Koz:adequate:Cav:weak:Exon 5:ExonStart false|VEMQ

CAG TCT ATG GCC :Koz:adequate:Cav:weak:Exon 5:ExonStart false|QSMA

ACT TCA ATG AGT :Koz:weak:Cav:weak:Exon 5:ExonStart false|TSMS

AAG GGA ATG GAA :Koz:strong:Cav:weak:Exon 5:ExonStart false|KGME

ATG GAA ATG GTG :Koz:strong:Cav:weak:Exon 5:ExonStart false|MEMV

AGT GAA ATG GAG :Koz:strong:Cav:weak:Exon 5:ExonStart false|SEME

CCA GCC ATG GTC :Koz:strong:Cav:weak:Exon 5:ExonStart false|PAMV

CCT ACA ATG CCT :Koz:adequate:Cav:adequate:Exon 5:ExonStart false|PTMP

CTT CCG ATG CAT :Koz:weak:Cav:weak:Exon 5:ExonStart false|LPMH

GCT GGA ATG CCC :Koz:adequate:Cav:weak:Exon 5:ExonStart false|AGMP

GGT CAC ATG TTG :Koz:weak:Cav:weak:Exon 5:ExonStart false|GHML

ATA CAA ATG CCT :Koz:weak:Cav:weak:Exon 5:ExonStart false|IQMP

ATA ATT ATG CCT :Koz:adequate:Cav:adequate:Exon 5:ExonStart false|IIMP

CCT GGC ATG CTA :Koz:adequate:Cav:weak:Exon 5:ExonStart false|PGML

CAG ATT ATG CCA :Koz:adequate:Cav:adequate:Exon 5:ExonStart false|QIMP

GAT TTT ATG CTC :Koz:weak:Cav:weak:Exon 7:ExonStart false|DFML

CAT TTA ATG CGA :Koz:weak:Cav:weak:Exon 7:ExonStart false|HLMR

GAC AAG ATG GGC :Koz:strong:Cav:strong:Exon 7:ExonStart false|DKMG

CCA CAA ATG GCT :Koz:adequate:Cav:weak:Exon 8:ExonStart false|PQMA

CAA CAA ATG CCT :Koz:weak:Cav:weak:Exon 8:ExonStart false|QQMP

AAG GCA ATG GAT :Koz:strong:Cav:weak:Exon 8:ExonStart false|KAMD

ATG GAT ATG CAG :Koz:adequate:Cav:weak:Exon 8:ExonStart false|MDMQ

GAT GAG ATG GAC :Koz:strong:Cav:weak:Exon 8:ExonStart false|DEMD

GAC CGC ATG TCA :Koz:weak:Cav:adequate:Exon 8:ExonStart false|DRMS

ATG TCA ATG ATT :Koz:weak:Cav:weak:Exon 8:ExonStart false|MSMI

GCA GGC ATG GAA :Koz:strong:Cav:weak:Exon 9:ExonStart false|AGME

CCA GCG ATG GCA :Koz:strong:Cav:weak:Exon 9:ExonStart false|PAMA

CCG GTA ATG TGG :Koz:adequate:Cav:weak:Exon 9:ExonStart false|PVMW

GTC CAA ATG CAC :Koz:weak:Cav:adequate:Exon 9:ExonStart false|VQMH

CAA AGA ATG CGA :Koz:adequate:Cav:adequate:Exon 9:ExonStart false|QRMR

AAG AAG ATG CAG :Koz:adequate:Cav:adequate:Exon 9:ExonStart false|KKMQ

CAT TGC ATG CTT :Koz:weak:Cav:weak:Exon 10:ExonStart false|HCML

CAG AAA ATG GCA :Koz:strong:Cav:adequate:Exon 10:ExonStart false|QKMA

CG1915-PC

AGC AAA ATG CAA :Koz:adequate:Cav:strong:Exon 2:ExonStart false|SKMQ

TTC CGG ATG AGC :Koz:weak:Cav:adequate:Exon 3:ExonStart false|FRMS

GCA CCG ATG CCC :Koz:weak:Cav:weak:Exon 3:ExonStart false|APMP

GAC GTA ATG GCC :Koz:strong:Cav:adequate:Exon 3:ExonStart false|DVMA

GGT CGT ATG ACC :Koz:weak:Cav:weak:Exon 3:ExonStart false|GRMT

TCG CTG ATG ATC :Koz:weak:Cav:weak:Exon 3:ExonStart false|SLMI

AGC ACC ATG ACT :Koz:adequate:Cav:strong:Exon 3:ExonStart false|STMT

ATC ACC ATG AGT :Koz:adequate:Cav:strong:Exon 3:ExonStart false|ITMS

GTT CAC ATG GAG :Koz:adequate:Cav:weak:Exon 4:ExonStart false|VHME

CCG ACC ATG GTC :Koz:strong:Cav:adequate:Exon 4:ExonStart false|PTMV

AGC ATT ATG GGT :Koz:strong:Cav:strong:Exon 5:ExonStart false|SIMG

GAG TAT ATG TGC :Koz:weak:Cav:weak:Exon 5:ExonStart false|ETMC

CCC TCC ATG AGA :Koz:weak:Cav:adequate:Exon 5:ExonStart false|PSMR

TCA ACC ATG CGC :Koz:adequate:Cav:adequate:Exon 5:ExonStart false|STMR

AAT GCG ATG GGT :Koz:strong:Cav:weak:Exon 5:ExonStart false|NAMG

GCA ACG ATG ATA :Koz:adequate:Cav:adequate:Exon 5:ExonStart false|ATMI

TCT AGC ATG CAC :Koz:adequate:Cav:adequate:Exon 6:ExonStart false|SSMH

GAA CCG ATG GGC :Koz:adequate:Cav:weak:Exon 6:ExonStart false|EPMG

CCC ACC ATG CGC :Koz:adequate:Cav:strong:Exon 6:ExonStart false|PTMR

ACC ATC ATG CAA :Koz:adequate:Cav:strong:Exon 6:ExonStart false|TIMQ

CCT AGT ATG CGC :Koz:adequate:Cav:adequate:Exon 6:ExonStart false|PSMR

CCC TCA ATG CGT :Koz:weak:Cav:adequate:Exon 6:ExonStart false|PSMR

GAT ATT ATG TCC :Koz:adequate:Cav:adequate:Exon 6:ExonStart false|DIMS

TTC ACC ATG CCG :Koz:adequate:Cav:strong:Exon 6:ExonStart false|FTMP

ACC ACC ATG CAT :Koz:adequate:Cav:strong:Exon 6:ExonStart false|TTMH

CTC AAC ATG AAG :Koz:adequate:Cav:strong:Exon 6:ExonStart false|LNMK

GCC GCC ATG CAC :Koz:adequate:Cav:adequate:Exon 6:ExonStart false|AAMH

CCT ACC ATG AAG :Koz:adequate:Cav:adequate:Exon 6:ExonStart false|PTMK

ACC TAC ATG TGC :Koz:weak:Cav:adequate:Exon 6:ExonStart false|TTMC

CGC AGC ATG ATC :Koz:adequate:Cav:strong:Exon 7:ExonStart false|RSMI

GTG TAC ATG TGC :Koz:weak:Cav:weak:Exon 7:ExonStart false|VTMC

GCC TCT ATG CGC :Koz:weak:Cav:adequate:Exon 7:ExonStart false|ASMR

ATC CAA ATG GAT :Koz:adequate:Cav:adequate:Exon 7:ExonStart false|IQMD

CTT CAA ATG GGC :Koz:adequate:Cav:weak:Exon 7:ExonStart false|LQMG

GTC TAC ATG TGC :Koz:weak:Cav:adequate:Exon 7:ExonStart false|VTMC

ACT GCC ATG AAG :Koz:adequate:Cav:weak:Exon 7:ExonStart false|TAMK

GCT GCC ATG AAT :Koz:adequate:Cav:weak:Exon 7:ExonStart false|AAMN

CCA GAG ATG TTT :Koz:adequate:Cav:weak:Exon 7:ExonStart false|PEMF

CCT ACC ATG TCC :Koz:adequate:Cav:adequate:Exon 7:ExonStart false|PTMS

AAG ACC ATG TTC :Koz:adequate:Cav:adequate:Exon 7:ExonStart false|KTMF

GGC AAA ATG CTG :Koz:adequate:Cav:strong:Exon 7:ExonStart false|GKML

TTC AAG ATG ACC :Koz:adequate:Cav:strong:Exon 7:ExonStart false|FKMT

GTT ACC ATG GAT :Koz:strong:Cav:adequate:Exon 7:ExonStart false|VTMD

ATG GAT ATG ATC :Koz:adequate:Cav:weak:Exon 7:ExonStart false|MDMI

CAG AGC ATG GTC :Koz:strong:Cav:adequate:Exon 7:ExonStart false|QSMV

AAG CTG ATG CGT :Koz:weak:Cav:weak:Exon 7:ExonStart false|KLMR

CGA GGA ATG GAA :Koz:strong:Cav:weak:Exon 7:ExonStart false|RGME

TAC AGC ATG GTC :Koz:strong:Cav:strong:Exon 7:ExonStart false|TSMV

GCC ACC ATG AAG :Koz:adequate:Cav:strong:Exon 7:ExonStart false|ATMK

ATC ATC ATG GAA :Koz:strong:Cav:strong:Exon 7:ExonStart false|IIME

AAG GGC ATG GAG :Koz:strong:Cav:weak:Exon 7:ExonStart false|KGME

TTC GAT ATG GTT :Koz:strong:Cav:adequate:Exon 7:ExonStart false|FDMV

CCA TCG ATG CGT :Koz:weak:Cav:weak:Exon 7:ExonStart false|PSMR

ATC GTT ATG GAG :Koz:strong:Cav:adequate:Exon 7:ExonStart false|IVME

GAG ACC ATG CAT :Koz:adequate:Cav:adequate:Exon 7:ExonStart false|ETMH

CCA ACC ATG CGC :Koz:adequate:Cav:adequate:Exon 7:ExonStart false|PTMR

CAT GTT ATG GCT :Koz:strong:Cav:weak:Exon 7:ExonStart false|HVMA

GAG GGC ATG ACC :Koz:adequate:Cav:weak:Exon 7:ExonStart false|EGMT

TTC GAC ATG GGC :Koz:strong:Cav:adequate:Exon 7:ExonStart false|FDMG

CGC GGC ATG AAG :Koz:adequate:Cav:adequate:Exon 8:ExonStart false|RGMK

ACC CAA ATG GAA :Koz:adequate:Cav:adequate:Exon 8:ExonStart false|TQME

ACC GAG ATG GCC :Koz:strong:Cav:adequate:Exon 8:ExonStart false|TEMA

CCT AAC ATG AAG :Koz:adequate:Cav:adequate:Exon 8:ExonStart false|PNMK

GTG GCA ATG AAC :Koz:adequate:Cav:weak:Exon 9:ExonStart false|VAMN

GCT CAC ATG CAG :Koz:weak:Cav:weak:Exon 9:ExonStart false|AHMQ

CGC GAA ATG GAA :Koz:strong:Cav:adequate:Exon 9:ExonStart false|REME

AGG GTT ATG TGG :Koz:adequate:Cav:weak:Exon 10:ExonStart false|RVMW

GAT GGT ATG TTC :Koz:adequate:Cav:weak:Exon 11:ExonStart false|DGMF

AAG GGA ATG GCC :Koz:strong:Cav:weak:Exon 12:ExonStart false|KGMA

GTT CAC ATG AAC :Koz:weak:Cav:weak:Exon 13:ExonStart false|VHMN

AAC GGC ATG AAG :Koz:adequate:Cav:adequate:Exon 13:ExonStart false|NGMK

TTC AAG ATG CTG :Koz:adequate:Cav:strong:Exon 13:ExonStart false|FKML

CGC TGG ATG CGT :Koz:weak:Cav:adequate:Exon 13:ExonStart false|RWMR

TTC CAG ATG AGC :Koz:weak:Cav:adequate:Exon 13:ExonStart false|FQMS

GAG GGC ATG CCC :Koz:adequate:Cav:weak:Exon 13:ExonStart false|EGMP

TTC CAG ATG ATC :Koz:weak:Cav:adequate:Exon 13:ExonStart false|FQMI

GGC ATC ATG AGT :Koz:adequate:Cav:strong:Exon 15:ExonStart false|GIMS

ATC ACT ATG CGT :Koz:adequate:Cav:strong:Exon 15:ExonStart false|ITMR

AAG AGC ATG GAA :Koz:strong:Cav:adequate:Exon 15:ExonStart false|KSME

GAG ATT ATG GAG :Koz:strong:Cav:adequate:Exon 15:ExonStart false|EIME

ATG GAG ATG ACT :Koz:adequate:Cav:weak:Exon 15:ExonStart false|MEMT

AAG GAG ATG CGC :Koz:adequate:Cav:weak:Exon 15:ExonStart false|KEMR

GTC GTC ATG ATT :Koz:adequate:Cav:adequate:Exon 15:ExonStart false|VVMI

AAC ATT ATG CAT :Koz:adequate:Cav:strong:Exon 15:ExonStart false|NIMH

TCC CAG ATG ATG :Koz:weak:Cav:adequate:Exon 15:ExonStart false|SQMM

CAG ATG ATG AGC :Koz:adequate:Cav:adequate:Exon 15:ExonStart false|QMMS

CCT GTT ATG TGG :Koz:adequate:Cav:weak:Exon 15:ExonStart false|PVMW

GAG GAA ATG CAC :Koz:adequate:Cav:weak:Exon 15:ExonStart false|EEMH

CCA GTT ATG TGG :Koz:adequate:Cav:weak:Exon 15:ExonStart false|PVMW

CCT GTC ATG TGG :Koz:adequate:Cav:weak:Exon 15:ExonStart false|PVMW

CCT GTC ATG TGG :Koz:adequate:Cav:weak:Exon 15:ExonStart false|PVMW

GAT GAA ATG ATT :Koz:adequate:Cav:weak:Exon 15:ExonStart false|DEMI

GAA GAG ATG CCA :Koz:adequate:Cav:weak:Exon 15:ExonStart false|EEMP

CTA GAA ATG AAG :Koz:adequate:Cav:weak:Exon 15:ExonStart false|LEMK

ACT GTC ATG GAG :Koz:strong:Cav:weak:Exon 15:ExonStart false|TVME

GTT CAA ATG TTC :Koz:weak:Cav:weak:Exon 16:ExonStart false|VQMF

AAA TGG ATG CGC :Koz:weak:Cav:weak:Exon 16:ExonStart false|KWMR

GTC AAT ATG ACT :Koz:adequate:Cav:strong:Exon 16:ExonStart false|VNMT

GAG GTT ATG GAT :Koz:strong:Cav:weak:Exon 16:ExonStart false|EVMD

TCT CAA ATG GAG :Koz:adequate:Cav:weak:Exon 19:ExonStart false|SQME

GAG GAA ATG CAG :Koz:adequate:Cav:weak:Exon 19:ExonStart false|EEMQ

AAC GAA ATG CGT :Koz:adequate:Cav:adequate:Exon 20:ExonStart false|NEMR

TAT CGC ATG GTA :Koz:adequate:Cav:weak:Exon 21:ExonStart false|TRMV

GAA ATA ATG AAA :Koz:adequate:Cav:adequate:Exon 21:ExonStart false|EIMK

ATT GGC ATG ACA :Koz:adequate:Cav:weak:Exon 21:ExonStart false|IGMT

ATG ACA ATG ACT :Koz:adequate:Cav:adequate:Exon 21:ExonStart false|MTMT

AAA ACT ATG GTG :Koz:strong:Cav:adequate:Exon 21:ExonStart false|KTMV

ACC AGC ATG AAT :Koz:adequate:Cav:strong:Exon 21:ExonStart false|TSMN

ACA TAT ATG TAT :Koz:weak:Cav:weak:Exon 21:ExonStart false|TTMT

ATA CGC ATG ATT :Koz:weak:Cav:weak:Exon 21:ExonStart false|IRMI

GCG CAG ATG AAC :Koz:weak:Cav:weak:Exon 23:ExonStart false|AQMN

CGG AAG ATG AAA :Koz:adequate:Cav:adequate:Exon 23:ExonStart false|RKMK

CCC AAC ATG AGC :Koz:adequate:Cav:strong:Exon 23:ExonStart false|PNMS

GGC GAA ATG CAA :Koz:adequate:Cav:adequate:Exon 23:ExonStart false|GEMQ

GAA CGC ATG GAG :Koz:adequate:Cav:weak:Exon 23:ExonStart false|ERME

CCT TCG ATG TCG :Koz:weak:Cav:weak:Exon 23:ExonStart false|PSMS

GAC CAT ATG CCA :Koz:weak:Cav:adequate:Exon 23:ExonStart false|DHMP

ATA GAT ATG CAA :Koz:adequate:Cav:weak:Exon 23:ExonStart false|IDMQ

ATG CAA ATG CAG :Koz:weak:Cav:weak:Exon 23:ExonStart false|MQMQ

CCG AAA ATG GCC :Koz:strong:Cav:adequate:Exon 23:ExonStart false|PKMA

CAG CAG ATG AAT :Koz:weak:Cav:weak:Exon 23:ExonStart false|QQMN

TCA AAT ATG TTT :Koz:adequate:Cav:adequate:Exon 23:ExonStart false|SNMF

GAG ACG ATG TCG :Koz:adequate:Cav:adequate:Exon 23:ExonStart false|ETMS

CCA GAT ATG TTA :Koz:adequate:Cav:weak:Exon 23:ExonStart false|PDML

CTT CCG ATG GAG :Koz:adequate:Cav:weak:Exon 23:ExonStart false|LPME

ATT AGC ATG CAT :Koz:adequate:Cav:adequate:Exon 23:ExonStart false|ISMH

CAG CAT ATG GAA :Koz:adequate:Cav:weak:Exon 23:ExonStart false|QHME

AGT GAC ATG ACG :Koz:adequate:Cav:weak:Exon 23:ExonStart false|SDMT

TCT GAA ATG ATT :Koz:adequate:Cav:weak:Exon 23:ExonStart false|SEMI

CAT ACA ATG GTG :Koz:strong:Cav:adequate:Exon 23:ExonStart false|HTMV

GAA GCC ATG GGA :Koz:strong:Cav:weak:Exon 23:ExonStart false|EAMG

TTA GCG ATG ACT :Koz:adequate:Cav:weak:Exon 23:ExonStart false|LAMT

GTT CAA ATG GAT :Koz:adequate:Cav:weak:Exon 23:ExonStart false|VQMD

CTA GTT ATG CAT :Koz:adequate:Cav:weak:Exon 23:ExonStart false|LVMH

TTA GAA ATG GAA :Koz:strong:Cav:weak:Exon 23:ExonStart false|LEME

AAA GCA ATG CCA :Koz:adequate:Cav:weak:Exon 24:ExonStart false|KAMP

GGT GAC ATG CAA :Koz:adequate:Cav:weak:Exon 25:ExonStart false|GDMQ

CCG AAC ATG GAG :Koz:strong:Cav:adequate:Exon 25:ExonStart false|PNME

GTA GTT ATG TTA :Koz:adequate:Cav:weak:Exon 25:ExonStart false|VVML

TTA AAA ATG ATC :Koz:adequate:Cav:adequate:Exon 25:ExonStart false|LKMI

GAT GAA ATG GAT :Koz:strong:Cav:weak:Exon 26:ExonStart false|DEMD

ACC GAA ATG GAA :Koz:strong:Cav:adequate:Exon 26:ExonStart false|TEME***NonAcetSite**

ACT CCA ATG AAA :Koz:weak:Cav:weak:Exon 26:ExonStart false|TPMK

CCC AAA ATG GTT :Koz:strong:Cav:strong:Exon 26:ExonStart false|PKMV

CCA CAA ATG CTC :Koz:weak:Cav:weak:Exon 26:ExonStart false|PQML

GCT GAA ATG CCA :Koz:adequate:Cav:weak:Exon 26:ExonStart false|AEMP

GAA GAG ATG GAG :Koz:strong:Cav:weak:Exon 26:ExonStart false|EEME

AAG CGA ATG AAC :Koz:weak:Cav:weak:Exon 26:ExonStart false|KRMN

ACT AGA ATG TCA :Koz:adequate:Cav:adequate:Exon 26:ExonStart false|TRMS

TCA GAG ATG GAC :Koz:strong:Cav:weak:Exon 26:ExonStart false|SEMD

AAG GAT ATG CCA :Koz:adequate:Cav:weak:Exon 26:ExonStart false|KDMP

GAA GAA ATG GCT :Koz:strong:Cav:weak:Exon 26:ExonStart false|EEMA

ACT GAA ATG GAT :Koz:strong:Cav:weak:Exon 26:ExonStart false|TEMD

GAG GAT ATG GAA :Koz:strong:Cav:weak:Exon 26:ExonStart false|EDME

GTA AAA ATG AAA :Koz:adequate:Cav:adequate:Exon 26:ExonStart false|VKMK

AAA CGA ATG TTG :Koz:weak:Cav:weak:Exon 26:ExonStart false|KRML

ATT ACA ATG ATT :Koz:adequate:Cav:adequate:Exon 26:ExonStart false|ITMI

GTA GAA ATG CCA :Koz:adequate:Cav:weak:Exon 26:ExonStart false|VEMP

AAA AAG ATG TTG :Koz:adequate:Cav:adequate:Exon 27:ExonStart false|KKML

GCC CGT ATG GTC :Koz:adequate:Cav:adequate:Exon 27:ExonStart false|ARMV

TTT GTA ATG ACT :Koz:adequate:Cav:weak:Exon 27:ExonStart false|FVMT

ATT TCC ATG TCT :Koz:weak:Cav:weak:Exon 28:ExonStart false|ISMS

AAC ATT ATG TAC :Koz:adequate:Cav:strong:Exon 29:ExonStart false|NIMT

TAT CTG ATG GAG :Koz:adequate:Cav:weak:Exon 29:ExonStart false|TLME

TTT CAA ATG TTC :Koz:weak:Cav:weak:Exon 30:ExonStart false|FQMF

CGC AGC ATG TCT :Koz:adequate:Cav:strong:Exon 30:ExonStart false|RSMS

TCC GAC ATG CAC :Koz:adequate:Cav:adequate:Exon 30:ExonStart false|SDMH

ATG CAC ATG TAC :Koz:weak:Cav:weak:Exon 30:ExonStart false|MHMT

CGC AAC ATG GTT :Koz:strong:Cav:strong:Exon 30:ExonStart false|RNMV

AGC GGA ATG GAT :Koz:strong:Cav:adequate:Exon 31:ExonStart false|SGMD

GTC ATA ATG CGC :Koz:adequate:Cav:strong:Exon 31:ExonStart false|VIMR

GAG ATA ATG CAT :Koz:adequate:Cav:adequate:Exon 32:ExonStart false|EIMH

GTT TGG ATG AAG :Koz:weak:Cav:weak:Exon 33:ExonStart false|VWMK

CTG TCG ATG AAC :Koz:weak:Cav:weak:Exon 33:ExonStart false|LSMN

CAA TAC ATG TTC :Koz:weak:Cav:weak:Exon 33:ExonStart false|QTMF

TCG GGC ATG AAC :Koz:adequate:Cav:weak:Exon 34:ExonStart false|SGMN

CGG GAC ATG AAG :Koz:adequate:Cav:weak:Exon 36:ExonStart false|RDMK

AAG ACC ATG TGG :Koz:adequate:Cav:adequate:Exon 36:ExonStart false|KTMW

GAG TAC ATG ATT :Koz:weak:Cav:weak:Exon 36:ExonStart false|ETMI

AAG GCA ATG ACT :Koz:adequate:Cav:weak:Exon 36:ExonStart false|KAMT

ACA GAG ATG AGT :Koz:adequate:Cav:weak:Exon 37:ExonStart false|TEMS

CAT CAC ATG GAT :Koz:adequate:Cav:weak:Exon 37:ExonStart false|HHMD

CG1943-PA

GAC AAA ATG ACA :Koz:adequate:Cav:strong:Exon 2:ExonStart false|DKMT*****

GCC TGC ATG GGC :Koz:adequate:Cav:adequate:Exon 2:ExonStart false|ACMG***NonAcetSite**

CG1943-PB

GAC AAA ATG ACA :Koz:adequate:Cav:strong:Exon 2:ExonStart false|DKMT*****

GCC TGC ATG GGC :Koz:adequate:Cav:adequate:Exon 2:ExonStart false|ACMG***NonAcetSite**

CG1943-PC

GAC AAA ATG ACA :Koz:adequate:Cav:strong:Exon 1:ExonStart false|DKMT*****

GCC TGC ATG GGC :Koz:adequate:Cav:adequate:Exon 1:ExonStart false|ACMG***NonAcetSite**

CG1963-PA

TCG AAA ATG TTG :Koz:adequate:Cav:adequate:Exon 1:ExonStart false|SKML

CGC AAA ATG GTG :Koz:strong:Cav:strong:Exon 2:ExonStart false|RKMV

AGC TTC ATG ACG :Koz:weak:Cav:adequate:Exon 3:ExonStart false|SFMT

ATT CGC ATG GCC :Koz:adequate:Cav:weak:Exon 3:ExonStart false|IRMA***NonAcetSite**

CG2093-PA

... ... ATG GTC :Koz:adequate:Cav:weak:Exon 1:ExonStart false|nullnullMV*****

TAT GAG ATG GAC :Koz:strong:Cav:weak:Exon 3:ExonStart false|TEMD

TTG GAA ATG GAT :Koz:strong:Cav:weak:Exon 4:ExonStart false|LEMD

TGC TAC ATG GCC :Koz:adequate:Cav:adequate:Exon 4:ExonStart false|CTMA

CTA AAC ATG AAC :Koz:adequate:Cav:adequate:Exon 5:ExonStart false|LNMN

CTG GAA ATG GAA :Koz:strong:Cav:weak:Exon 5:ExonStart false|LEME

AAT CTC ATG AAG :Koz:weak:Cav:weak:Exon 5:ExonStart false|NLMK

GAC GCT ATG AAC :Koz:adequate:Cav:adequate:Exon 5:ExonStart false|DAMN

GCG GCA ATG ACC :Koz:adequate:Cav:weak:Exon 7:ExonStart false|AAMT

GAG AAG ATG TAC :Koz:adequate:Cav:adequate:Exon 7:ExonStart false|EKMT

ATC AGG ATG AAC :Koz:adequate:Cav:strong:Exon 7:ExonStart false|IRMN

TTT TCT ATG GCC :Koz:adequate:Cav:weak:Exon 7:ExonStart false|FSMA

GCT GGC ATG AGA :Koz:adequate:Cav:weak:Exon 7:ExonStart false|AGMR

ACT GGA ATG CAG :Koz:adequate:Cav:weak:Exon 7:ExonStart false|TGMQ

CAG TAC ATG ATC :Koz:weak:Cav:weak:Exon 7:ExonStart false|QTMI

CTG TTA ATG CCA :Koz:weak:Cav:weak:Exon 7:ExonStart false|LLMP

GTT AGC ATG GGC :Koz:strong:Cav:adequate:Exon 7:ExonStart false|VSMG

ACG GTA ATG GAA :Koz:strong:Cav:weak:Exon 7:ExonStart false|TVME

GTG CAG ATG CTG :Koz:weak:Cav:weak:Exon 7:ExonStart false|VQML

ACG GAG ATG CAC :Koz:adequate:Cav:weak:Exon 7:ExonStart false|TEMH

AGC TCA ATG TCC :Koz:weak:Cav:adequate:Exon 7:ExonStart false|SSMS

AGT GAC ATG GAT :Koz:strong:Cav:weak:Exon 8:ExonStart false|SDMD

AGG ATT ATG GAG :Koz:strong:Cav:adequate:Exon 8:ExonStart false|RIME

GAT CGG ATG GGC :Koz:adequate:Cav:weak:Exon 8:ExonStart false|DRMG

GAA ATA ATG ACC :Koz:adequate:Cav:adequate:Exon 8:ExonStart false|EIMT

GAC CAG ATG AAG :Koz:weak:Cav:adequate:Exon 8:ExonStart false|DQMK

GCA GAG ATG AAT :Koz:adequate:Cav:weak:Exon 8:ExonStart false|AEMN

GAT GAT ATG AAA :Koz:adequate:Cav:weak:Exon 9:ExonStart false|DDMK

GGC TGC ATG AAG :Koz:weak:Cav:adequate:Exon 9:ExonStart false|GCMK

GGT GTT ATG AAC :Koz:adequate:Cav:weak:Exon 10:ExonStart false|GVMN

AAG GCA ATG GAT :Koz:strong:Cav:weak:Exon 10:ExonStart false|KAMD

ACT CGT ATG AAA :Koz:weak:Cav:weak:Exon 10:ExonStart false|TRMK

TTC AAT ATG ATG :Koz:adequate:Cav:strong:Exon 11:ExonStart false|FNMM

AAT ATG ATG AAT :Koz:adequate:Cav:adequate:Exon 11:ExonStart false|NMMN

AAT CCC ATG AGT :Koz:weak:Cav:weak:Exon 11:ExonStart false|NPMS

TTG GTA ATG CTT :Koz:adequate:Cav:weak:Exon 12:ExonStart false|LVML

AAT TTA ATG GAG :Koz:adequate:Cav:weak:Exon 12:ExonStart false|NLME***NonAcetSite**

ATT CAA ATG GAT :Koz:adequate:Cav:weak:Exon 13:ExonStart false|IQMD

GAT GAC ATG CGA :Koz:adequate:Cav:weak:Exon 13:ExonStart false|DDMR

AAC TTC ATG GTC :Koz:adequate:Cav:adequate:Exon 13:ExonStart false|NFMV

CGA AAA ATG AAT :Koz:adequate:Cav:adequate:Exon 15:ExonStart false|RKMN

CTG AAG ATG TAC :Koz:adequate:Cav:adequate:Exon 16:ExonStart false|LKMT

ATG TAC ATG TGT :Koz:weak:Cav:weak:Exon 16:ExonStart false|MTMC

CGA GAG ATG ACG :Koz:adequate:Cav:weak:Exon 16:ExonStart false|REMT

GAG GGC ATG CAC :Koz:adequate:Cav:weak:Exon 16:ExonStart false|EGMH

AAG GCA ATG CTG :Koz:adequate:Cav:weak:Exon 16:ExonStart false|KAML

GGC ACC ATG ACC :Koz:adequate:Cav:strong:Exon 16:ExonStart false|GTMT

CTA AAT ATG CCT :Koz:adequate:Cav:adequate:Exon 17:ExonStart false|LNMP

TTC GAA ATG GGC :Koz:strong:Cav:adequate:Exon 17:ExonStart false|FEMG

ATG GGC ATG GAG :Koz:strong:Cav:weak:Exon 17:ExonStart false|MGME

CAG GCG ATG CAC :Koz:adequate:Cav:weak:Exon 17:ExonStart false|QAMH

ATG CAC ATG AAC :Koz:weak:Cav:weak:Exon 17:ExonStart false|MHMN

CTT CTT ATG GTG :Koz:adequate:Cav:weak:Exon 17:ExonStart false|LLMV

AAC CTG ATG CGC :Koz:weak:Cav:adequate:Exon 18:ExonStart false|NLMR

TTT TCC ATG CGC :Koz:weak:Cav:weak:Exon 19:ExonStart false|FSMR

CTT ATC ATG AAC :Koz:adequate:Cav:adequate:Exon 19:ExonStart false|LIMN

TCG GAA ATG AAG :Koz:adequate:Cav:weak:Exon 20:ExonStart false|SEMK

GTG GAC ATG GAT :Koz:strong:Cav:weak:Exon 20:ExonStart false|VDMD

AGC TTG ATG CTT :Koz:weak:Cav:adequate:Exon 21:ExonStart false|SLML

TTT TGG ATG ATC :Koz:weak:Cav:weak:Exon 21:ExonStart false|FWMI

ACC GGC ATG ATG :Koz:adequate:Cav:adequate:Exon 21:ExonStart false|TGMM

GGC ATG ATG CTC :Koz:adequate:Cav:strong:Exon 21:ExonStart false|GMML

AAT GAG ATG GAA :Koz:strong:Cav:weak:Exon 21:ExonStart false|NEME

ACT ATA ATG TAT :Koz:adequate:Cav:adequate:Exon 21:ExonStart false|TIMT

CCA AAA ATG CTT :Koz:adequate:Cav:adequate:Exon 21:ExonStart false|PKML

GTG ATC ATG CAA :Koz:adequate:Cav:adequate:Exon 22:ExonStart false|VIMQ

GAC CTT ATG ATC :Koz:weak:Cav:adequate:Exon 23:ExonStart false|DLMI

TGC AGC ATG GTG :Koz:strong:Cav:strong:Exon 23:ExonStart false|CSMV

CGC ATC ATG CCG :Koz:adequate:Cav:strong:Exon 23:ExonStart false|RIMP

GCC GAA ATG TTT :Koz:adequate:Cav:adequate:Exon 23:ExonStart false|AEMF

TTC TCG ATG GCC :Koz:adequate:Cav:adequate:Exon 23:ExonStart false|FSMA

GGT GCT ATG GGA :Koz:strong:Cav:weak:Exon 25:ExonStart false|GAMG

CTA GTG ATG GGC :Koz:strong:Cav:weak:Exon 26:ExonStart false|LVMG

AAG CGT ATG CGA :Koz:weak:Cav:weak:Exon 26:ExonStart false|KRMR

TGC TTG ATG GAG :Koz:adequate:Cav:adequate:Exon 26:ExonStart false|CLME

AAA ATA ATG AAG :Koz:adequate:Cav:adequate:Exon 27:ExonStart true|KIMK

CGG GTG ATG TAC :Koz:adequate:Cav:weak:Exon 27:ExonStart false|RVMT

AAC GAG ATG TTC :Koz:adequate:Cav:adequate:Exon 27:ExonStart false|NEMF

CG2163-PA

AAA GCA ATG GCC :Koz:strong:Cav:weak:Exon 2:ExonStart false|KAMA

GGC AGC ATG CAA :Koz:adequate:Cav:strong:Exon 3:ExonStart false|GSMQ***AcetSite**

AAG GAG ATG GAA :Koz:strong:Cav:weak:Exon 3:ExonStart false|KEME

AAG CAG ATG CAG :Koz:weak:Cav:weak:Exon 3:ExonStart false|KQMQ

AAA CAA ATG GCC :Koz:adequate:Cav:weak:Exon 3:ExonStart false|KQMA

TTG GCC ATG AAC :Koz:adequate:Cav:weak:Exon 3:ExonStart false|LAMN

AAG GTA ATG TCG :Koz:adequate:Cav:weak:Exon 4:ExonStart false|KVMS

AGA GCT ATG GGT :Koz:strong:Cav:weak:Exon 4:ExonStart false|RAMG

CG2163-PB

AAA GCA ATG GCC :Koz:strong:Cav:weak:Exon 2:ExonStart false|KAMA

GGC AGC ATG CAA :Koz:adequate:Cav:strong:Exon 3:ExonStart false|GSMQ***AcetSite**

AAG GAG ATG GAA :Koz:strong:Cav:weak:Exon 3:ExonStart false|KEME

AAG CAG ATG CAG :Koz:weak:Cav:weak:Exon 3:ExonStart false|KQMQ

AAA CAA ATG GCC :Koz:adequate:Cav:weak:Exon 3:ExonStart false|KQMA

TTG GCC ATG AAC :Koz:adequate:Cav:weak:Exon 3:ExonStart false|LAMN

AAG GTA ATG TCG :Koz:adequate:Cav:weak:Exon 4:ExonStart false|KVMS

AGA GCT ATG GGT :Koz:strong:Cav:weak:Exon 4:ExonStart false|RAMG

CG2210-PA

GAA CAC ATG AAG :Koz:weak:Cav:weak:Exon 1:ExonStart false|EHMK

CTC CTG ATG CTC :Koz:weak:Cav:adequate:Exon 1:ExonStart false|LLML

GCG ACA ATG GCG :Koz:strong:Cav:adequate:Exon 1:ExonStart false|ATMA***AcetSite**

TTC ATC ATG GTC :Koz:strong:Cav:strong:Exon 1:ExonStart false|FIMV

AAC TAC ATG AAC :Koz:weak:Cav:adequate:Exon 2:ExonStart false|NTMN

GTG CCC ATG GTG :Koz:adequate:Cav:weak:Exon 2:ExonStart false|VPMV

CGC CAG ATG CTC :Koz:weak:Cav:adequate:Exon 2:ExonStart false|RQML***NonAcetSite**

CG2216-PA

TCA AAG ATG GTG :Koz:strong:Cav:adequate:Exon 1:ExonStart false|SKMV

GTG GAC ATG AAG :Koz:adequate:Cav:weak:Exon 2:ExonStart false|VDMK

AAG GGC ATG CGC :Koz:adequate:Cav:weak:Exon 2:ExonStart false|KGMR

TTG GCC ATG GGC :Koz:strong:Cav:weak:Exon 2:ExonStart false|LAMG

CTG TCC ATG CGC :Koz:weak:Cav:weak:Exon 2:ExonStart false|LSMR

AAG AAG ATG ATG :Koz:adequate:Cav:adequate:Exon 3:ExonStart false|KKMM***NonAcetSite**

AAG ATG ATG GAC :Koz:strong:Cav:adequate:Exon 3:ExonStart false|KMMD

CG2216-PB

TCA AAG ATG GTG :Koz:strong:Cav:adequate:Exon 2:ExonStart false|SKMV

GTG GAC ATG AAG :Koz:adequate:Cav:weak:Exon 3:ExonStart false|VDMK

AAG GGC ATG CGC :Koz:adequate:Cav:weak:Exon 3:ExonStart false|KGMR

TTG GCC ATG GGC :Koz:strong:Cav:weak:Exon 3:ExonStart false|LAMG

CTG TCC ATG CGC :Koz:weak:Cav:weak:Exon 3:ExonStart false|LSMR

AAG AAG ATG ATG :Koz:adequate:Cav:adequate:Exon 4:ExonStart false|KKMM***NonAcetSite**

AAG ATG ATG GAC :Koz:strong:Cav:adequate:Exon 4:ExonStart false|KMMD

CG2216-PC

TCA AAG ATG GTG :Koz:strong:Cav:adequate:Exon 2:ExonStart false|SKMV

GTG GAC ATG AAG :Koz:adequate:Cav:weak:Exon 3:ExonStart false|VDMK

AAG GGC ATG CGC :Koz:adequate:Cav:weak:Exon 3:ExonStart false|KGMR

TTG GCC ATG GGC :Koz:strong:Cav:weak:Exon 3:ExonStart false|LAMG

CTG TCC ATG CGC :Koz:weak:Cav:weak:Exon 3:ExonStart false|LSMR

AAG AAG ATG ATG :Koz:adequate:Cav:adequate:Exon 4:ExonStart false|KKMM***NonAcetSite**

AAG ATG ATG GAC :Koz:strong:Cav:adequate:Exon 4:ExonStart false|KMMD

CG2216-PD

TCA AAG ATG GTG :Koz:strong:Cav:adequate:Exon 2:ExonStart false|SKMV

GTG GAC ATG AAG :Koz:adequate:Cav:weak:Exon 3:ExonStart false|VDMK

AAG GGC ATG CGC :Koz:adequate:Cav:weak:Exon 3:ExonStart false|KGMR

TTG GCC ATG GGC :Koz:strong:Cav:weak:Exon 3:ExonStart false|LAMG

CTG TCC ATG CGC :Koz:weak:Cav:weak:Exon 3:ExonStart false|LSMR

AAG AAG ATG ATG :Koz:adequate:Cav:adequate:Exon 4:ExonStart false|KKMM***NonAcetSite**

AAG ATG ATG GAC :Koz:strong:Cav:adequate:Exon 4:ExonStart false|KMMD

CG2331-PA

TTT ATC ATG GCA :Koz:strong:Cav:adequate:Exon 1:ExonStart false|FIMA*****
GCG AAG ATG GAT :Koz:strong:Cav:adequate:Exon 3:ExonStart false|AKMD
ATC CGC ATG AAC :Koz:weak:Cav:adequate:Exon 3:ExonStart false|IRMN
ATC CAC ATG GGC :Koz:adequate:Cav:adequate:Exon 3:ExonStart false|IHMG
GCC GCC ATG CGT :Koz:adequate:Cav:adequate:Exon 3:ExonStart false|AAMR
AAG GAG ATG GTT :Koz:strong:Cav:weak:Exon 3:ExonStart false|KEMV
ATT CTT ATG TAC :Koz:weak:Cav:weak:Exon 3:ExonStart false|ILMY*NonAcetSite
GAG ATC ATG TCC :Koz:adequate:Cav:adequate:Exon 3:ExonStart false|EIMS***NonAcetSite**
ACC CTG ATG GAT :Koz:adequate:Cav:adequate:Exon 3:ExonStart false|TLMD
GAT GGC ATG AAG :Koz:adequate:Cav:weak:Exon 3:ExonStart false|DGMK
ATC GTT ATG GCT :Koz:strong:Cav:adequate:Exon 3:ExonStart false|IVMA
AAG AAC ATG AAG :Koz:adequate:Cav:adequate:Exon 3:ExonStart false|KNMK
GAG AAG ATG GAC :Koz:strong:Cav:adequate:Exon 3:ExonStart false|EKMD
GTG ACA ATG GAG :Koz:strong:Cav:adequate:Exon 3:ExonStart false|VTME
TAC GCT ATG ACC :Koz:adequate:Cav:adequate:Exon 3:ExonStart false|YAMT
TTT GGC ATG CAG :Koz:adequate:Cav:weak:Exon 3:ExonStart false|FGMQ
CTG ACC ATG TGG :Koz:adequate:Cav:adequate:Exon 3:ExonStart false|LTMW
ACC GAA ATG GAT :Koz:strong:Cav:adequate:Exon 3:ExonStart false|TEMD
GAT GGC ATG GGA :Koz:strong:Cav:weak:Exon 3:ExonStart false|DGMG
TCC GCG ATG GAC :Koz:strong:Cav:adequate:Exon 3:ExonStart false|SAMD
ATG GAC ATG GAC :Koz:strong:Cav:weak:Exon 3:ExonStart false|MDMD
GAG GCC ATG AAG :Koz:adequate:Cav:weak:Exon 4:ExonStart false|EAMK
TAC GAG ATG TTT :Koz:adequate:Cav:adequate:Exon 4:ExonStart false|YEMF

CG2358-PA

GCA GCC ATG GGC :Koz:strong:Cav:weak:Exon 1:ExonStart false|AAMG*****

GCC AGC ATG TTG :Koz:adequate:Cav:strong:Exon 1:ExonStart false|ASML***AcetSite**

GAC GAG ATG CTG :Koz:adequate:Cav:adequate:Exon 1:ExonStart false|DEML

AAC AGA ATG AAC :Koz:adequate:Cav:strong:Exon 1:ExonStart false|NRMN

TTC GCC ATG ATC :Koz:adequate:Cav:adequate:Exon 2:ExonStart false|FAMI

GCG CTG ATG ATC :Koz:weak:Cav:weak:Exon 2:ExonStart false|ALMI

GGC CTG ATG GTG :Koz:adequate:Cav:adequate:Exon 2:ExonStart false|GLMV

GGC AGC ATG GAG :Koz:strong:Cav:strong:Exon 2:ExonStart false|GSME

ATC TTC ATG AAC :Koz:weak:Cav:adequate:Exon 3:ExonStart false|IFMN

CG2679-PB

TAC GTG ATG TAT :Koz:adequate:Cav:adequate:Exon 2:ExonStart false|TVMT

GCG GCC ATG TCC :Koz:adequate:Cav:weak:Exon 2:ExonStart false|AAMS***NonAcetSite**

TCC GGC ATG GAA :Koz:strong:Cav:adequate:Exon 2:ExonStart false|SGME

GGT AAC ATG CTG :Koz:adequate:Cav:adequate:Exon 2:ExonStart false|GNML

TGC AAC ATG GAA :Koz:strong:Cav:strong:Exon 2:ExonStart false|CNME

CAA GTA ATG CAG :Koz:adequate:Cav:weak:Exon 2:ExonStart false|QVMQ

GAG AAG ATG CAG :Koz:adequate:Cav:adequate:Exon 2:ExonStart false|EKMQ

GTT TTG ATG ATT :Koz:weak:Cav:weak:Exon 4:ExonStart false|VLMI

CGT TAC ATG CAG :Koz:weak:Cav:weak:Exon 4:ExonStart false|RTMQ

GCT ATC ATG AAG :Koz:adequate:Cav:adequate:Exon 5:ExonStart false|AIMK

TGT CCC ATG TGT :Koz:weak:Cav:weak:Exon 6:ExonStart false|CPMC

CGC GAA ATG CTG :Koz:adequate:Cav:adequate:Exon 7:ExonStart false|REML

AGC CTG ATG AGC :Koz:weak:Cav:adequate:Exon 7:ExonStart false|SLMS

CGC AGC ATG CGC :Koz:adequate:Cav:strong:Exon 7:ExonStart false|RSMR

GAT GAA ATG CAG :Koz:adequate:Cav:weak:Exon 7:ExonStart false|DEMQ

CG2863-PA

GCG AGA ATG TTG :Koz:adequate:Cav:adequate:Exon 1:ExonStart false|ARML

CAG AAA ATG CAG :Koz:adequate:Cav:adequate:Exon 1:ExonStart false|QKMQ***NonAcetSite**

AGT TCC ATG CCG :Koz:weak:Cav:weak:Exon 1:ExonStart false|SSMP

TGC CTT ATG AAC :Koz:weak:Cav:adequate:Exon 1:ExonStart false|CLMN

GTG AAG ATG TGG :Koz:adequate:Cav:adequate:Exon 1:ExonStart false|VKMW

GAG CGC ATG ACA :Koz:weak:Cav:weak:Exon 2:ExonStart false|ERMT

CAG TAC ATG GCC :Koz:adequate:Cav:weak:Exon 2:ExonStart false|QTMA

CG2890-PA

GCC TAA ATG GTC :Koz:adequate:Cav:adequate:Exon 2:ExonStart false|AstopMV*****

GTC ACC ATG GAA :Koz:strong:Cav:strong:Exon 2:ExonStart false|VTME***AcetSite**

GAG ATA ATG CAG :Koz:adequate:Cav:adequate:Exon 2:ExonStart false|EIMQ

GAG ACC ATG AAG :Koz:adequate:Cav:adequate:Exon 3:ExonStart false|ETMK

AAG TTT ATG CGT :Koz:weak:Cav:weak:Exon 3:ExonStart false|KFMR

CTC TCC ATG GAG :Koz:adequate:Cav:adequate:Exon 4:ExonStart false|LSME

ATT GAA ATG GAG :Koz:strong:Cav:weak:Exon 4:ExonStart false|IEME

GAC CCA ATG GTG :Koz:adequate:Cav:adequate:Exon 4:ExonStart false|DPMV

CAG CCC ATG GAG :Koz:adequate:Cav:weak:Exon 4:ExonStart false|QPME

GTG GTA ATG GCC :Koz:strong:Cav:weak:Exon 4:ExonStart false|VVMA

GGC GCA ATG GCC :Koz:strong:Cav:adequate:Exon 4:ExonStart false|GAMA

GCC GCC ATG GAG :Koz:strong:Cav:adequate:Exon 4:ExonStart false|AAME

GAG GTG ATG ATG :Koz:adequate:Cav:weak:Exon 4:ExonStart false|EVMM

GTG ATG ATG CAG :Koz:adequate:Cav:adequate:Exon 4:ExonStart false|VMMQ

CG2890-PB

GCC TAA ATG GTC :Koz:adequate:Cav:adequate:Exon 1:ExonStart false|AstopMV

GTC ACC ATG GAA :Koz:strong:Cav:strong:Exon 1:ExonStart false|VTME***AcetSite**

GAG ATA ATG CAG :Koz:adequate:Cav:adequate:Exon 1:ExonStart false|EIMQ

GAG ACC ATG AAG :Koz:adequate:Cav:adequate:Exon 2:ExonStart false|ETMK

AAG TTT ATG CGT :Koz:weak:Cav:weak:Exon 2:ExonStart false|KFMR

CTC TCC ATG GAG :Koz:adequate:Cav:adequate:Exon 3:ExonStart false|LSME

ATT GAA ATG GAG :Koz:strong:Cav:weak:Exon 3:ExonStart false|IEME

GAC CCA ATG GTG :Koz:adequate:Cav:adequate:Exon 3:ExonStart false|DPMV

CAG CCC ATG GAG :Koz:adequate:Cav:weak:Exon 3:ExonStart false|QPME

GTG GTA ATG GCC :Koz:strong:Cav:weak:Exon 3:ExonStart false|VVMA

GGC GCA ATG GCC :Koz:strong:Cav:adequate:Exon 3:ExonStart false|GAMA

GCC GCC ATG GAG :Koz:strong:Cav:adequate:Exon 3:ExonStart false|AAME

GAG GTG ATG ATG :Koz:adequate:Cav:weak:Exon 3:ExonStart false|EVMM

GTG ATG ATG CAG :Koz:adequate:Cav:adequate:Exon 3:ExonStart false|VMMQ

CG2890-PC

GCC TAA ATG GTC :Koz:adequate:Cav:adequate:Exon 2:ExonStart false|AstopMV

GTC ACC ATG GAA :Koz:strong:Cav:strong:Exon 2:ExonStart false|VTME***AcetSite**

GAG ATA ATG CAG :Koz:adequate:Cav:adequate:Exon 2:ExonStart false|EIMQ

GAG ACC ATG AAG :Koz:adequate:Cav:adequate:Exon 3:ExonStart false|ETMK

AAG TTT ATG CGT :Koz:weak:Cav:weak:Exon 3:ExonStart false|KFMR

CTC TCC ATG GAG :Koz:adequate:Cav:adequate:Exon 4:ExonStart false|LSME

ATT GAA ATG GAG :Koz:strong:Cav:weak:Exon 4:ExonStart false|IEME

GAC CCA ATG GTG :Koz:adequate:Cav:adequate:Exon 4:ExonStart false|DPMV

CAG CCC ATG GAG :Koz:adequate:Cav:weak:Exon 4:ExonStart false|QPME

GTG GTA ATG GCC :Koz:strong:Cav:weak:Exon 4:ExonStart false|VVMA

GGC GCA ATG GCC :Koz:strong:Cav:adequate:Exon 4:ExonStart false|GAMA

GCC GCC ATG GAG :Koz:strong:Cav:adequate:Exon 4:ExonStart false|AAME

GAG GTG ATG ATG :Koz:adequate:Cav:weak:Exon 4:ExonStart false|EVMM

GTG ATG ATG CAG :Koz:adequate:Cav:adequate:Exon 4:ExonStart false|VMMQ

CG2903-PA

ATA GAA ATG AGC :Koz:adequate:Cav:weak:Exon 1:ExonStart false|IEMS

GAC ACC ATG ACC :Koz:adequate:Cav:strong:Exon 2:ExonStart false|DTMT

GAT GCC ATG TTC :Koz:adequate:Cav:weak:Exon 2:ExonStart false|DAMF

TAT CGC ATG CAG :Koz:weak:Cav:weak:Exon 3:ExonStart false|TRMQ

TCA TCC ATG GCA :Koz:adequate:Cav:weak:Exon 3:ExonStart false|SSMA

AGC CCC ATG CCA :Koz:weak:Cav:adequate:Exon 3:ExonStart false|SPMP

CAG ATC ATG CCC :Koz:adequate:Cav:adequate:Exon 3:ExonStart false|QIMP

GCC AAT ATG CGC :Koz:adequate:Cav:strong:Exon 3:ExonStart false|ANMR

AAC CGC ATG AAG :Koz:weak:Cav:adequate:Exon 3:ExonStart false|NRMK

CTC TTC ATG ACC :Koz:weak:Cav:adequate:Exon 3:ExonStart false|LFMT

AAG GAG ATG GAC :Koz:strong:Cav:weak:Exon 3:ExonStart false|KEMD

AAG CGC ATG TGG :Koz:weak:Cav:weak:Exon 3:ExonStart false|KRMW

CGA CAG ATG CAG :Koz:weak:Cav:weak:Exon 3:ExonStart false|RQMQ***NonAcetSite**

ATG CAG ATG GCC :Koz:adequate:Cav:weak:Exon 3:ExonStart false|MQMA

GAC ATA ATG CGC :Koz:adequate:Cav:strong:Exon 3:ExonStart false|DIMR

CGC GAG ATG CAG :Koz:adequate:Cav:adequate:Exon 3:ExonStart false|REMQ

TAT TTG ATG GGC :Koz:adequate:Cav:weak:Exon 3:ExonStart false|TLMG

CCC TAC ATG CCA :Koz:weak:Cav:adequate:Exon 3:ExonStart false|PTMP

CCG GGC ATG TAC :Koz:adequate:Cav:weak:Exon 3:ExonStart false|PGMT

CAG CCG ATG CCG :Koz:weak:Cav:weak:Exon 3:ExonStart false|QPMP

GGA TTA ATG CAA :Koz:weak:Cav:weak:Exon 3:ExonStart false|GLMQ

CCC CAG ATG ATG :Koz:weak:Cav:adequate:Exon 3:ExonStart false|PQMM

CAG ATG ATG CCG :Koz:adequate:Cav:adequate:Exon 3:ExonStart false|QMMP

CCG CCC ATG CCC :Koz:weak:Cav:weak:Exon 3:ExonStart false|PPMP

CCC CAA ATG GGT :Koz:adequate:Cav:adequate:Exon 3:ExonStart false|PQMG

CAC GTA ATG CTG :Koz:adequate:Cav:adequate:Exon 3:ExonStart false|HVML

CG2903-PB

GAA AAC ATG TTT :Koz:adequate:Cav:adequate:Exon 1:ExonStart false|ENMF

AAG AAG ATG AAC :Koz:adequate:Cav:adequate:Exon 3:ExonStart false|KKMN

TGC GAG ATG TTC :Koz:adequate:Cav:adequate:Exon 3:ExonStart false|CEMF

CAG AAG ATG CTG :Koz:adequate:Cav:adequate:Exon 3:ExonStart false|QKML

GAC ACC ATG ACC :Koz:adequate:Cav:strong:Exon 4:ExonStart false|DTMT

GAT GCC ATG TTC :Koz:adequate:Cav:weak:Exon 4:ExonStart false|DAMF

TAT CGC ATG CAG :Koz:weak:Cav:weak:Exon 5:ExonStart false|TRMQ

TCA TCC ATG GCA :Koz:adequate:Cav:weak:Exon 5:ExonStart false|SSMA

AGC CCC ATG CCA :Koz:weak:Cav:adequate:Exon 5:ExonStart false|SPMP

CAG ATC ATG CCC :Koz:adequate:Cav:adequate:Exon 5:ExonStart false|QIMP

GCC AAT ATG CGC :Koz:adequate:Cav:strong:Exon 5:ExonStart false|ANMR

AAC CGC ATG AAG :Koz:weak:Cav:adequate:Exon 5:ExonStart false|NRMK

CTC TTC ATG ACC :Koz:weak:Cav:adequate:Exon 5:ExonStart false|LFMT

AAG GAG ATG GAC :Koz:strong:Cav:weak:Exon 5:ExonStart false|KEMD

AAG CGC ATG TGG :Koz:weak:Cav:weak:Exon 5:ExonStart false|KRMW

CGA CAG ATG CAG :Koz:weak:Cav:weak:Exon 5:ExonStart false|RQMQ***NonAcetSite**

ATG CAG ATG GCC :Koz:adequate:Cav:weak:Exon 5:ExonStart false|MQMA

GAC ATA ATG CGC :Koz:adequate:Cav:strong:Exon 5:ExonStart false|DIMR

CGC GAG ATG CAG :Koz:adequate:Cav:adequate:Exon 5:ExonStart false|REMQ

TAT TTG ATG GGC :Koz:adequate:Cav:weak:Exon 5:ExonStart false|TLMG

CCC TAC ATG CCA :Koz:weak:Cav:adequate:Exon 5:ExonStart false|PTMP

CCG GGC ATG TAC :Koz:adequate:Cav:weak:Exon 5:ExonStart false|PGMT

CAG CCG ATG CCG :Koz:weak:Cav:weak:Exon 5:ExonStart false|QPMP

GGA TTA ATG CAA :Koz:weak:Cav:weak:Exon 5:ExonStart false|GLMQ

CCC CAG ATG ATG :Koz:weak:Cav:adequate:Exon 5:ExonStart false|PQMM

CAG ATG ATG CCG :Koz:adequate:Cav:adequate:Exon 5:ExonStart false|QMMP

CCG CCC ATG CCC :Koz:weak:Cav:weak:Exon 5:ExonStart false|PPMP

CCC CAA ATG GGT :Koz:adequate:Cav:adequate:Exon 5:ExonStart false|PQMG

CAC GTA ATG CTG :Koz:adequate:Cav:adequate:Exon 5:ExonStart false|HVML

CG2903-PC

GAA AAC ATG TTT :Koz:adequate:Cav:adequate:Exon 2:ExonStart false|ENMF

AAG AAG ATG AAC :Koz:adequate:Cav:adequate:Exon 4:ExonStart false|KKMN

TGC GAG ATG TTC :Koz:adequate:Cav:adequate:Exon 4:ExonStart false|CEMF

CAG AAG ATG CTG :Koz:adequate:Cav:adequate:Exon 4:ExonStart false|QKML

GAC ACC ATG ACC :Koz:adequate:Cav:strong:Exon 5:ExonStart false|DTMT

GAT GCC ATG TTC :Koz:adequate:Cav:weak:Exon 5:ExonStart false|DAMF

TAT CGC ATG CAG :Koz:weak:Cav:weak:Exon 6:ExonStart false|TRMQ

TCA TCC ATG GCA :Koz:adequate:Cav:weak:Exon 6:ExonStart false|SSMA

AGC CCC ATG CCA :Koz:weak:Cav:adequate:Exon 6:ExonStart false|SPMP

CAG ATC ATG CCC :Koz:adequate:Cav:adequate:Exon 6:ExonStart false|QIMP

GCC AAT ATG CGC :Koz:adequate:Cav:strong:Exon 6:ExonStart false|ANMR

AAC CGC ATG AAG :Koz:weak:Cav:adequate:Exon 6:ExonStart false|NRMK

CTC TTC ATG ACC :Koz:weak:Cav:adequate:Exon 6:ExonStart false|LFMT

AAG GAG ATG GAC :Koz:strong:Cav:weak:Exon 6:ExonStart false|KEMD

AAG CGC ATG TGG :Koz:weak:Cav:weak:Exon 6:ExonStart false|KRMW

CGA CAG ATG CAG :Koz:weak:Cav:weak:Exon 6:ExonStart false|RQMQ***NonAcetSite**

ATG CAG ATG GCC :Koz:adequate:Cav:weak:Exon 6:ExonStart false|MQMA

GAC ATA ATG CGC :Koz:adequate:Cav:strong:Exon 6:ExonStart false|DIMR

CGC GAG ATG CAG :Koz:adequate:Cav:adequate:Exon 6:ExonStart false|REMQ

TAT TTG ATG GGC :Koz:adequate:Cav:weak:Exon 6:ExonStart false|TLMG

CCC TAC ATG CCA :Koz:weak:Cav:adequate:Exon 6:ExonStart false|PTMP

CCG GGC ATG TAC :Koz:adequate:Cav:weak:Exon 6:ExonStart false|PGMT

CAG CCG ATG CCG :Koz:weak:Cav:weak:Exon 6:ExonStart false|QPMP

GGA TTA ATG CAA :Koz:weak:Cav:weak:Exon 6:ExonStart false|GLMQ

CCC CAG ATG ATG :Koz:weak:Cav:adequate:Exon 6:ExonStart false|PQMM

CAG ATG ATG CCG :Koz:adequate:Cav:adequate:Exon 6:ExonStart false|QMMP

CCG CCC ATG CCC :Koz:weak:Cav:weak:Exon 6:ExonStart false|PPMP

CCC CAA ATG GGT :Koz:adequate:Cav:adequate:Exon 6:ExonStart false|PQMG

CAC GTA ATG CTG :Koz:adequate:Cav:adequate:Exon 6:ExonStart false|HVML

CG2915-PA

GGA AAA ATG GGA :Koz:strong:Cav:adequate:Exon 2:ExonStart false|GKMG

ATA AAT ATG CCA :Koz:adequate:Cav:adequate:Exon 2:ExonStart false|INMP

GTA GCG ATG GCA :Koz:strong:Cav:weak:Exon 2:ExonStart false|VAMA***AcetSite**

GAC AAA ATG GTC :Koz:strong:Cav:strong:Exon 3:ExonStart false|DKMV

CTG GAT ATG GGC :Koz:strong:Cav:weak:Exon 3:ExonStart false|LDMG

GAC CGA ATG TGG :Koz:weak:Cav:adequate:Exon 3:ExonStart false|DRMW

TCT CAA ATG CTA :Koz:weak:Cav:weak:Exon 3:ExonStart false|SQML

ATG CTA ATG TTC :Koz:weak:Cav:weak:Exon 3:ExonStart false|MLMF

GGC AGC ATG GAT :Koz:strong:Cav:strong:Exon 3:ExonStart false|GSMD

CG2915-PB

GGA AAA ATG GGA :Koz:strong:Cav:adequate:Exon 1:ExonStart false|GKMG

ATA AAT ATG CCA :Koz:adequate:Cav:adequate:Exon 1:ExonStart false|INMP

GTA GCG ATG GCA :Koz:strong:Cav:weak:Exon 1:ExonStart false|VAMA***AcetSite**

GAC AAA ATG GTC :Koz:strong:Cav:strong:Exon 2:ExonStart false|DKMV

CTG GAT ATG GGC :Koz:strong:Cav:weak:Exon 2:ExonStart false|LDMG

GAC CGA ATG TGG :Koz:weak:Cav:adequate:Exon 2:ExonStart false|DRMW

TCT CAA ATG CTA :Koz:weak:Cav:weak:Exon 2:ExonStart false|SQML

ATG CTA ATG TTC :Koz:weak:Cav:weak:Exon 2:ExonStart false|MLMF

GGC AGC ATG GAT :Koz:strong:Cav:strong:Exon 2:ExonStart false|GSMD

CG3011-PA

AAA GAC ATG CAG :Koz:adequate:Cav:weak:Exon 2:ExonStart false|KDMQ

AAG AAT ATG GCC :Koz:strong:Cav:adequate:Exon 3:ExonStart false|KNMA***AcetSite**

CTC GAG ATG ATC :Koz:adequate:Cav:adequate:Exon 3:ExonStart false|LEMI

CGC ATC ATG GGC :Koz:strong:Cav:strong:Exon 4:ExonStart false|RIMG

GAG AGC ATG CCG :Koz:adequate:Cav:adequate:Exon 4:ExonStart false|ESMP

TAC CTG ATG GCC :Koz:adequate:Cav:adequate:Exon 4:ExonStart false|TLMA

GCC GAC ATG GCC :Koz:strong:Cav:adequate:Exon 4:ExonStart false|ADMA

TCC GCC ATG AAT :Koz:adequate:Cav:adequate:Exon 4:ExonStart false|SAMN

CG30323-PA

TTT GCC ATG ATG :Koz:adequate:Cav:weak:Exon 1:ExonStart false|FAMM

GCC ATG ATG CTG :Koz:adequate:Cav:strong:Exon 1:ExonStart false|AMML

TCT GCG ATG TGC :Koz:adequate:Cav:weak:Exon 1:ExonStart false|SAMC

TTC AGC ATG GTG :Koz:strong:Cav:strong:Exon 1:ExonStart false|FSMV***NonAcetSite**

CTG TGC ATG ACT :Koz:weak:Cav:weak:Exon 1:ExonStart false|LCMT

GGC AAT ATG TGC :Koz:adequate:Cav:strong:Exon 1:ExonStart false|GNMC

GGC TTC ATG TTT :Koz:weak:Cav:adequate:Exon 1:ExonStart false|GFMF

CG30410-PA

AAC CAC ATG TTA :Koz:weak:Cav:adequate:Exon 1:ExonStart false|NHML

ATA CGC ATG ATG :Koz:weak:Cav:weak:Exon 1:ExonStart false|IRMM***AcetSite**

CGC ATG ATG GAT :Koz:strong:Cav:strong:Exon 1:ExonStart false|RMMD***AcetSite**

CGC CAT ATG GTG :Koz:adequate:Cav:adequate:Exon 1:ExonStart false|RHMV

GCG CCC ATG GCG :Koz:adequate:Cav:weak:Exon 1:ExonStart false|APMA

TTG CGT ATG GCC :Koz:adequate:Cav:weak:Exon 1:ExonStart false|LRMA

GTG AAC ATG GCC :Koz:strong:Cav:adequate:Exon 1:ExonStart false|VNMA

TAC GGC ATG GCC :Koz:strong:Cav:adequate:Exon 1:ExonStart false|TGMA

CG31363-PA

GGC AAA ATG ATC :Koz:adequate:Cav:strong:Exon 1:ExonStart false|GKMI

TCG GAG ATG CCG :Koz:adequate:Cav:weak:Exon 2:ExonStart false|SEMP

AAT CGC ATG GCG :Koz:adequate:Cav:weak:Exon 2:ExonStart false|NRMA***NonAcetSite**

AAC CAC ATG AAG :Koz:weak:Cav:adequate:Exon 3:ExonStart false|NHMK

CTC AAG ATG AAC :Koz:adequate:Cav:strong:Exon 3:ExonStart false|LKMN

CGC AAT ATG AGC :Koz:adequate:Cav:strong:Exon 4:ExonStart false|RNMS

CG31363-PC

ACA GAA ATG GCC :Koz:strong:Cav:weak:Exon 2:ExonStart false|TEMA

TCG GAG ATG CCG :Koz:adequate:Cav:weak:Exon 3:ExonStart false|SEMP

AAT CGC ATG GCG :Koz:adequate:Cav:weak:Exon 3:ExonStart false|NRMA***NonAcetSite**

AAC CAC ATG AAG :Koz:weak:Cav:adequate:Exon 4:ExonStart false|NHMK

CTC AAG ATG AAC :Koz:adequate:Cav:strong:Exon 4:ExonStart false|LKMN

CG31363-PD

GGC AAA ATG ATC :Koz:adequate:Cav:strong:Exon 1:ExonStart false|GKMI

TCG GAG ATG CCG :Koz:adequate:Cav:weak:Exon 2:ExonStart false|SEMP

AAT CGC ATG GCG :Koz:adequate:Cav:weak:Exon 2:ExonStart false|NRMA***NonAcetSite**

AAC CAC ATG AAG :Koz:weak:Cav:adequate:Exon 3:ExonStart false|NHMK

CTC AAG ATG AAC :Koz:adequate:Cav:strong:Exon 3:ExonStart false|LKMN

CG3163-PA

CAA AAA ATG GAC :Koz:strong:Cav:adequate:Exon 1:ExonStart false|QKMD

GGG GAG ATG TAC :Koz:adequate:Cav:weak:Exon 1:ExonStart false|GEMT

TCC CTG ATG TTC :Koz:weak:Cav:adequate:Exon 1:ExonStart false|SLMF

AAC TCC ATG CAG :Koz:weak:Cav:adequate:Exon 1:ExonStart false|NSMQ

ATC AAG ATG TCA :Koz:adequate:Cav:strong:Exon 1:ExonStart false|IKMS

CGC TCC ATG GAC :Koz:adequate:Cav:adequate:Exon 1:ExonStart false|RSMD

GGC CAG ATG AAA :Koz:weak:Cav:adequate:Exon 1:ExonStart false|GQMK

ATG AAA ATG CTG :Koz:adequate:Cav:adequate:Exon 1:ExonStart false|MKML***NonAcetSite**

CAG TTT ATG GGC :Koz:adequate:Cav:weak:Exon 1:ExonStart false|QFMG

CG3210-PA

CTC AGA ATG GAG :Koz:strong:Cav:strong:Exon 1:ExonStart false|LRME

ACG GAC ATG GCC :Koz:strong:Cav:weak:Exon 3:ExonStart false|TDMA

GAT CTC ATG GAT :Koz:adequate:Cav:weak:Exon 3:ExonStart false|DLMD

GGC GTT ATG AAT :Koz:adequate:Cav:adequate:Exon 3:ExonStart false|GVMN

GAC ATC ATG GAC :Koz:strong:Cav:strong:Exon 3:ExonStart false|DIMD

GAT CAG ATG AAG :Koz:weak:Cav:weak:Exon 3:ExonStart false|DQMK

CTC CTG ATG CAC :Koz:weak:Cav:adequate:Exon 3:ExonStart false|LLMH

AAC ATC ATG GCC :Koz:strong:Cav:strong:Exon 4:ExonStart false|NIMA

GCC CGG ATG GGC :Koz:adequate:Cav:adequate:Exon 4:ExonStart false|ARMG***AcetSite**

AGC AAG ATG GAC :Koz:strong:Cav:strong:Exon 4:ExonStart false|SKMD

GAG GAG ATG CAG :Koz:adequate:Cav:weak:Exon 4:ExonStart false|EEMQ

CAG GAG ATG CTG :Koz:adequate:Cav:weak:Exon 4:ExonStart false|QEML

AAC GTG ATG GTG :Koz:strong:Cav:adequate:Exon 5:ExonStart false|NVMV

AAC CAC ATG TCG :Koz:weak:Cav:adequate:Exon 5:ExonStart false|NHMS

CCT TCG ATG GCC :Koz:adequate:Cav:weak:Exon 6:ExonStart false|PSMA

GCC ATC ATG CAT :Koz:adequate:Cav:strong:Exon 7:ExonStart false|AIMH

GCA GAT ATG CTG :Koz:adequate:Cav:weak:Exon 7:ExonStart false|ADML

ACC CAC ATG TGG :Koz:weak:Cav:adequate:Exon 8:ExonStart false|THMW

CG32281-PA

GTT TGG ATG TTT :Koz:weak:Cav:weak:Exon 1:ExonStart false|VWMF

CGA AAC ATG GAC :Koz:strong:Cav:adequate:Exon 1:ExonStart false|RNMD***AcetSite**

CGC GGA ATG CAG :Koz:adequate:Cav:adequate:Exon 1:ExonStart false|RGMQ

CGT GTT ATG CCG :Koz:adequate:Cav:weak:Exon 1:ExonStart false|RVMP

CTG AAA ATG GAG :Koz:strong:Cav:adequate:Exon 1:ExonStart false|LKME

GTC AAA ATG CTG :Koz:adequate:Cav:strong:Exon 1:ExonStart false|VKML

ATA AAG ATG AGC :Koz:adequate:Cav:adequate:Exon 1:ExonStart false|IKMS

ATT ACA ATG AAT :Koz:adequate:Cav:adequate:Exon 1:ExonStart false|ITMN

CCA GCA ATG GCT :Koz:strong:Cav:weak:Exon 1:ExonStart false|PAMA

AAG GAC ATG TAT :Koz:adequate:Cav:weak:Exon 1:ExonStart false|KDMT

CTA AAC ATG CTA :Koz:adequate:Cav:adequate:Exon 1:ExonStart false|LNML

CG3265-PA

ACC AAC ATG GCT :Koz:strong:Cav:strong:Exon 2:ExonStart false|TNMA*****

CAC GAT ATG CTA :Koz:adequate:Cav:adequate:Exon 2:ExonStart false|HDML

CAG TTC ATG GAC :Koz:adequate:Cav:weak:Exon 3:ExonStart false|QFMD

ATG GAC ATG CTG :Koz:adequate:Cav:weak:Exon 3:ExonStart false|MDML

AAG AAG ATG TCT :Koz:adequate:Cav:adequate:Exon 3:ExonStart false|KKMS***NonAcetSite**

GCC CCA ATG GGC :Koz:adequate:Cav:adequate:Exon 4:ExonStart false|APMG

CCA GCA ATG ACG :Koz:adequate:Cav:weak:Exon 4:ExonStart false|PAMT

GAA GAG ATG TCA :Koz:adequate:Cav:weak:Exon 5:ExonStart false|EEMS

CAG GTG ATG GAT :Koz:strong:Cav:weak:Exon 6:ExonStart false|QVMD

ATG GAT ATG CGC :Koz:adequate:Cav:weak:Exon 6:ExonStart false|MDMR

CG3265-PB

ACC AAC ATG GCT :Koz:strong:Cav:strong:Exon 2:ExonStart false|TNMA

CAC GAT ATG CTA :Koz:adequate:Cav:adequate:Exon 2:ExonStart false|HDML

CAG TTC ATG GAC :Koz:adequate:Cav:weak:Exon 3:ExonStart false|QFMD

ATG GAC ATG CTG :Koz:adequate:Cav:weak:Exon 3:ExonStart false|MDML

AAG AAG ATG TCT :Koz:adequate:Cav:adequate:Exon 3:ExonStart false|KKMS***NonAcetSite**

GCC CCA ATG GGC :Koz:adequate:Cav:adequate:Exon 4:ExonStart false|APMG

CCA GCA ATG ACG :Koz:adequate:Cav:weak:Exon 4:ExonStart false|PAMT

GAA GAG ATG TCA :Koz:adequate:Cav:weak:Exon 5:ExonStart false|EEMS

CAG GTG ATG GAT :Koz:strong:Cav:weak:Exon 6:ExonStart false|QVMD

ATG GAT ATG CGC :Koz:adequate:Cav:weak:Exon 6:ExonStart false|MDMR

CG3265-PD

ACC AAC ATG GCT :Koz:strong:Cav:strong:Exon 2:ExonStart false|TNMA

CAC GAT ATG CTA :Koz:adequate:Cav:adequate:Exon 2:ExonStart false|HDML

CAG TTC ATG GAC :Koz:adequate:Cav:weak:Exon 3:ExonStart false|QFMD

ATG GAC ATG CTG :Koz:adequate:Cav:weak:Exon 3:ExonStart false|MDML

AAG AAG ATG TCT :Koz:adequate:Cav:adequate:Exon 3:ExonStart false|KKMS***NonAcetSite**

GCC CCA ATG GGC :Koz:adequate:Cav:adequate:Exon 4:ExonStart false|APMG

CCA GCA ATG ACG :Koz:adequate:Cav:weak:Exon 4:ExonStart false|PAMT

GAA GAG ATG TCA :Koz:adequate:Cav:weak:Exon 5:ExonStart false|EEMS

CAG GTG ATG GAT :Koz:strong:Cav:weak:Exon 6:ExonStart false|QVMD

ATG GAT ATG CGC :Koz:adequate:Cav:weak:Exon 6:ExonStart false|MDMR

CG3265-PE

ACC AAC ATG GCT :Koz:strong:Cav:strong:Exon 2:ExonStart false|TNMA

CAC GAT ATG CTA :Koz:adequate:Cav:adequate:Exon 2:ExonStart false|HDML

CAG TTC ATG GAC :Koz:adequate:Cav:weak:Exon 3:ExonStart false|QFMD

ATG GAC ATG CTG :Koz:adequate:Cav:weak:Exon 3:ExonStart false|MDML

AAG AAG ATG TCT :Koz:adequate:Cav:adequate:Exon 3:ExonStart false|KKMS***NonAcetSite**

GCC CCA ATG GGC :Koz:adequate:Cav:adequate:Exon 4:ExonStart false|APMG

CCA GCA ATG ACG :Koz:adequate:Cav:weak:Exon 4:ExonStart false|PAMT

GAA GAG ATG TCA :Koz:adequate:Cav:weak:Exon 5:ExonStart false|EEMS

CAG GTG ATG GAT :Koz:strong:Cav:weak:Exon 6:ExonStart false|QVMD

ATG GAT ATG CGC :Koz:adequate:Cav:weak:Exon 6:ExonStart false|MDMR

CG3273-PA

CCC AAG ATG GAC :Koz:strong:Cav:strong:Exon 1:ExonStart false|PKMD

ATC TCC ATG ACT :Koz:weak:Cav:adequate:Exon 1:ExonStart false|ISMT

ATG ACT ATG GAC :Koz:strong:Cav:adequate:Exon 1:ExonStart false|MTMD

CAG GCC ATG GAG :Koz:strong:Cav:weak:Exon 1:ExonStart false|QAME

CGT CAC ATG AAA :Koz:weak:Cav:weak:Exon 1:ExonStart false|RHMK

AAA TGG ATG CGG :Koz:weak:Cav:weak:Exon 1:ExonStart false|KWMR

GAA GAG ATG AAG :Koz:adequate:Cav:weak:Exon 1:ExonStart false|EEMK

TCA GTC ATG GAG :Koz:strong:Cav:weak:Exon 1:ExonStart false|SVME

AAG AAC ATG CTG :Koz:adequate:Cav:adequate:Exon 1:ExonStart false|KNML

TCG AGC ATG GAG :Koz:strong:Cav:adequate:Exon 1:ExonStart false|SSME

TGT GTA ATG GCA :Koz:strong:Cav:weak:Exon 1:ExonStart false|CVMA

GAA GTC ATG CAT :Koz:adequate:Cav:weak:Exon 1:ExonStart false|EVMH

GAA CCC ATG ATT :Koz:weak:Cav:weak:Exon 1:ExonStart false|EPMI

GGC ACC ATG AAA :Koz:adequate:Cav:strong:Exon 1:ExonStart false|GTMK

CTG AAG ATG AGA :Koz:adequate:Cav:adequate:Exon 1:ExonStart false|LKMR***NonAcetSite**

CG3298-PB

ATA CGA ATG TAT :Koz:weak:Cav:weak:Exon 1:ExonStart false|IRMT

AAT TTG ATG GCG :Koz:adequate:Cav:weak:Exon 1:ExonStart false|NLMA***AcetSite**

GGC TCT ATG CTG :Koz:weak:Cav:adequate:Exon 1:ExonStart false|GSML

CAG TCA ATG CGA :Koz:weak:Cav:weak:Exon 1:ExonStart false|QSMR

GTC GCC ATG ACA :Koz:adequate:Cav:adequate:Exon 1:ExonStart false|VAMT

GAC ACT ATG CCA :Koz:adequate:Cav:strong:Exon 2:ExonStart false|DTMP

GCG ACA ATG GAG :Koz:strong:Cav:adequate:Exon 2:ExonStart false|ATME

CGG AAT ATG AAC :Koz:adequate:Cav:adequate:Exon 2:ExonStart false|RNMN

GAG GAT ATG CAA :Koz:adequate:Cav:weak:Exon 2:ExonStart false|EDMQ

GAT AAT ATG GAA :Koz:strong:Cav:adequate:Exon 2:ExonStart false|DNME

TTC GCG ATG TAC :Koz:adequate:Cav:adequate:Exon 2:ExonStart false|FAMT

CG33138-PA

TCC GCT ATG GCC :Koz:strong:Cav:adequate:Exon 1:ExonStart false|SAMA

CAG GTT ATG GCC :Koz:strong:Cav:weak:Exon 3:ExonStart false|QVMA

GCC ATC ATG GAG :Koz:strong:Cav:strong:Exon 3:ExonStart false|AIME

AAG CGC ATG ATC :Koz:weak:Cav:weak:Exon 3:ExonStart false|KRMI

ACC TCT ATG CTA :Koz:weak:Cav:adequate:Exon 3:ExonStart false|TSML

TCT GGA ATG CCT :Koz:adequate:Cav:weak:Exon 4:ExonStart false|SGMP

TTG GGT ATG GCC :Koz:strong:Cav:weak:Exon 4:ExonStart false|LGMA

TGG GAC ATG GGC :Koz:strong:Cav:weak:Exon 4:ExonStart false|WDMG

CGC TGG ATG GAG :Koz:adequate:Cav:adequate:Exon 4:ExonStart false|RWME

TGG CTG ATG GAC :Koz:adequate:Cav:weak:Exon 4:ExonStart false|WLMD

AAG GAG ATG TAT :Koz:adequate:Cav:weak:Exon 4:ExonStart false|KEMT

ACG CAC ATG TCG :Koz:weak:Cav:weak:Exon 4:ExonStart false|THMS***NonAcetSite**

CAC AAG ATG ATT :Koz:adequate:Cav:strong:Exon 4:ExonStart false|HKMI

AAC TTT ATG GGC :Koz:adequate:Cav:adequate:Exon 4:ExonStart false|NFMG

CGA GCC ATG AAT :Koz:adequate:Cav:weak:Exon 4:ExonStart false|RAMN

CG33139-PA

CGT GTG ATG GAA :Koz:strong:Cav:weak:Exon 1:ExonStart false|RVME

ATG GAA ATG GCA :Koz:strong:Cav:weak:Exon 1:ExonStart false|MEMA

CTG AGC ATG CGA :Koz:adequate:Cav:adequate:Exon 1:ExonStart false|LSMR

CGC TGG ATG GCG :Koz:adequate:Cav:adequate:Exon 1:ExonStart false|RWMA

ACA CTC ATG AAG :Koz:weak:Cav:weak:Exon 1:ExonStart false|TLMK

CGT CTG ATG GCC :Koz:adequate:Cav:weak:Exon 1:ExonStart false|RLMA

GAG GCC ATG GCC :Koz:strong:Cav:weak:Exon 1:ExonStart false|EAMA

GTG GTG ATG AGA :Koz:adequate:Cav:weak:Exon 1:ExonStart false|VVMR

GAT CAC ATG AAC :Koz:weak:Cav:weak:Exon 1:ExonStart false|DHMN***AcetSite**

ATT AAG ATG ATG :Koz:adequate:Cav:adequate:Exon 1:ExonStart false|IKMM

AAG ATG ATG AAG :Koz:adequate:Cav:adequate:Exon 1:ExonStart false|KMMK

GAA CTG ATG GAG :Koz:adequate:Cav:weak:Exon 1:ExonStart false|ELME

CTG AAC ATG CTG :Koz:adequate:Cav:adequate:Exon 1:ExonStart false|LNML

GGT ATC ATG CTG :Koz:adequate:Cav:adequate:Exon 1:ExonStart false|GIML

CAA GTG ATG ATT :Koz:adequate:Cav:weak:Exon 1:ExonStart false|QVMI

GAG GAC ATG CCC :Koz:adequate:Cav:weak:Exon 1:ExonStart false|EDMP

GAG TTT ATG CCG :Koz:weak:Cav:weak:Exon 1:ExonStart false|EFMP

GGC ACG ATG ACG :Koz:adequate:Cav:strong:Exon 1:ExonStart false|GTMT

GAG AAG ATG AGC :Koz:adequate:Cav:adequate:Exon 1:ExonStart false|EKMS

GAG CCC ATG AAG :Koz:weak:Cav:weak:Exon 1:ExonStart false|EPMK

GGT ATC ATG CTC :Koz:adequate:Cav:adequate:Exon 2:ExonStart false|GIML

ATC GAG ATG TCA :Koz:adequate:Cav:adequate:Exon 2:ExonStart false|IEMS

ATT GCC ATG CTG :Koz:adequate:Cav:weak:Exon 2:ExonStart false|IAML

TAT CCC ATG ACA :Koz:weak:Cav:weak:Exon 2:ExonStart false|TPMT

ATG ACA ATG GGA :Koz:strong:Cav:adequate:Exon 2:ExonStart false|MTMG

GTT TTC ATG TCG :Koz:weak:Cav:weak:Exon 2:ExonStart false|VFMS

ATC GAG ATG TTC :Koz:adequate:Cav:adequate:Exon 2:ExonStart false|IEMF

TTC CCA ATG GTT :Koz:adequate:Cav:adequate:Exon 2:ExonStart false|FPMV

GAA GTG ATG GAC :Koz:strong:Cav:weak:Exon 2:ExonStart false|EVMD

CAG GTA ATG TGC :Koz:adequate:Cav:weak:Exon 2:ExonStart false|QVMC

ATT ACC ATG TAC :Koz:adequate:Cav:adequate:Exon 2:ExonStart false|ITMT

CG3396-PA

... ... ATG TTC :Koz:weak:Cav:weak:Exon 1:ExonStart false|nullnullMF

CAT CTA ATG TCG :Koz:weak:Cav:weak:Exon 3:ExonStart false|HLMS

AAC ACC ATG TAC :Koz:adequate:Cav:strong:Exon 3:ExonStart false|NTMT

TTC AAC ATG AAG :Koz:adequate:Cav:strong:Exon 3:ExonStart false|FNMK***NonAcetSite**

AAC GAT ATG GAC :Koz:strong:Cav:adequate:Exon 3:ExonStart false|NDMD

GAC AAC ATG GCC :Koz:strong:Cav:strong:Exon 3:ExonStart false|DNMA

CG3399-PA

GAA CTC ATG GCC :Koz:adequate:Cav:weak:Exon 2:ExonStart false|ELMA

GGC ACA ATG GAG :Koz:strong:Cav:strong:Exon 2:ExonStart false|GTME

GGT CAA ATG CTA :Koz:weak:Cav:weak:Exon 4:ExonStart false|GQML

TCA ACG ATG CAA :Koz:adequate:Cav:adequate:Exon 4:ExonStart false|STMQ

TAT GAA ATG TAC :Koz:adequate:Cav:weak:Exon 5:ExonStart false|TEMT

CCG CCC ATG AGT :Koz:weak:Cav:weak:Exon 5:ExonStart false|PPMS

AAT ACC ATG CGC :Koz:adequate:Cav:adequate:Exon 6:ExonStart false|NTMR

AAG CCA ATG CGT :Koz:weak:Cav:weak:Exon 6:ExonStart false|KPMR

CAG CAC ATG AGC :Koz:weak:Cav:weak:Exon 6:ExonStart false|QHMS***NonAcetSite**

ATT TCC ATG GCC :Koz:adequate:Cav:weak:Exon 6:ExonStart false|ISMA

AAC TAT ATG AAC :Koz:weak:Cav:adequate:Exon 6:ExonStart false|NTMN

GCA CAA ATG GAC :Koz:adequate:Cav:weak:Exon 6:ExonStart false|AQMD

GAG ATC ATG GAG :Koz:strong:Cav:adequate:Exon 7:ExonStart false|EIME

TCC AAA ATG GAG :Koz:strong:Cav:strong:Exon 7:ExonStart false|SKME

AAA TCG ATG GCC :Koz:adequate:Cav:weak:Exon 7:ExonStart false|KSMA

GAG ACC ATG CGC :Koz:adequate:Cav:adequate:Exon 7:ExonStart false|ETMR

GAA TTA ATG AAG :Koz:weak:Cav:weak:Exon 8:ExonStart true|ELMK

GAG CGC ATG CTG :Koz:weak:Cav:weak:Exon 8:ExonStart false|ERML

ATG CTG ATG AGG :Koz:weak:Cav:weak:Exon 8:ExonStart false|MLMR

CG3399-PB

ATT TTA ATG CGA :Koz:weak:Cav:weak:Exon 2:ExonStart false|ILMR

GGT CAA ATG CTA :Koz:weak:Cav:weak:Exon 4:ExonStart false|GQML

TCA ACG ATG CAA :Koz:adequate:Cav:adequate:Exon 4:ExonStart false|STMQ

TAT GAA ATG TAC :Koz:adequate:Cav:weak:Exon 5:ExonStart false|TEMT

CCG CCC ATG AGT :Koz:weak:Cav:weak:Exon 5:ExonStart false|PPMS

AAT ACC ATG CGC :Koz:adequate:Cav:adequate:Exon 6:ExonStart false|NTMR

AAG CCA ATG CGT :Koz:weak:Cav:weak:Exon 6:ExonStart false|KPMR

CAG CAC ATG AGC :Koz:weak:Cav:weak:Exon 6:ExonStart false|QHMS***NonAcetSite**

ATT TCC ATG GCC :Koz:adequate:Cav:weak:Exon 6:ExonStart false|ISMA

AAC TAT ATG AAC :Koz:weak:Cav:adequate:Exon 6:ExonStart false|NTMN

GCA CAA ATG GAC :Koz:adequate:Cav:weak:Exon 6:ExonStart false|AQMD

GAG ATC ATG GAG :Koz:strong:Cav:adequate:Exon 7:ExonStart false|EIME

TCC AAA ATG GAG :Koz:strong:Cav:strong:Exon 7:ExonStart false|SKME

AAA TCG ATG GCC :Koz:adequate:Cav:weak:Exon 7:ExonStart false|KSMA

GAG ACC ATG CGC :Koz:adequate:Cav:adequate:Exon 7:ExonStart false|ETMR

GAA TTA ATG AAG :Koz:weak:Cav:weak:Exon 8:ExonStart true|ELMK

GAG CGC ATG CTG :Koz:weak:Cav:weak:Exon 8:ExonStart false|ERML

ATG CTG ATG AGG :Koz:weak:Cav:weak:Exon 8:ExonStart false|MLMR

CG3399-PC

GGT AAG ATG ATT :Koz:adequate:Cav:adequate:Exon 1:ExonStart false|GKMI

TAT CCA ATG GAA :Koz:adequate:Cav:weak:Exon 1:ExonStart false|TPME

CCG GCT ATG ATG :Koz:adequate:Cav:weak:Exon 1:ExonStart false|PAMM

GCT ATG ATG GTC :Koz:strong:Cav:adequate:Exon 1:ExonStart false|AMMV

TCC GCC ATG CAG :Koz:adequate:Cav:adequate:Exon 1:ExonStart false|SAMQ

ATC AAC ATG GGG :Koz:strong:Cav:strong:Exon 2:ExonStart false|INMG

GTG CGA ATG GGA :Koz:adequate:Cav:weak:Exon 2:ExonStart false|VRMG

CGT TTG ATG GGT :Koz:adequate:Cav:weak:Exon 2:ExonStart false|RLMG

GGT CAA ATG CTA :Koz:weak:Cav:weak:Exon 4:ExonStart false|GQML

TCA ACG ATG CAA :Koz:adequate:Cav:adequate:Exon 4:ExonStart false|STMQ

TAT GAA ATG TAC :Koz:adequate:Cav:weak:Exon 5:ExonStart false|TEMT

CCG CCC ATG AGT :Koz:weak:Cav:weak:Exon 5:ExonStart false|PPMS

AAT ACC ATG CGC :Koz:adequate:Cav:adequate:Exon 6:ExonStart false|NTMR

AAG CCA ATG CGT :Koz:weak:Cav:weak:Exon 6:ExonStart false|KPMR

CAG CAC ATG AGC :Koz:weak:Cav:weak:Exon 6:ExonStart false|QHMS***NonAcetSite**

ATT TCC ATG GCC :Koz:adequate:Cav:weak:Exon 6:ExonStart false|ISMA

AAC TAT ATG AAC :Koz:weak:Cav:adequate:Exon 6:ExonStart false|NTMN

GCA CAA ATG GAC :Koz:adequate:Cav:weak:Exon 6:ExonStart false|AQMD

GAG ATC ATG GAG :Koz:strong:Cav:adequate:Exon 7:ExonStart false|EIME

TCC AAA ATG GAG :Koz:strong:Cav:strong:Exon 7:ExonStart false|SKME

AAA TCG ATG GCC :Koz:adequate:Cav:weak:Exon 7:ExonStart false|KSMA

GAG ACC ATG CGC :Koz:adequate:Cav:adequate:Exon 7:ExonStart false|ETMR

GAA TTA ATG AAG :Koz:weak:Cav:weak:Exon 8:ExonStart true|ELMK

GAG CGC ATG CTG :Koz:weak:Cav:weak:Exon 8:ExonStart false|ERML

ATG CTG ATG AGG :Koz:weak:Cav:weak:Exon 8:ExonStart false|MLMR

CG3399-PD

ATC AAC ATG GGG :Koz:strong:Cav:strong:Exon 2:ExonStart false|INMG

GTG CGA ATG GGA :Koz:adequate:Cav:weak:Exon 2:ExonStart false|VRMG

CGT TTG ATG GGT :Koz:adequate:Cav:weak:Exon 2:ExonStart false|RLMG

ATT TTA ATG CGA :Koz:weak:Cav:weak:Exon 3:ExonStart false|ILMR

GGT CAA ATG CTA :Koz:weak:Cav:weak:Exon 5:ExonStart false|GQML

TCA ACG ATG CAA :Koz:adequate:Cav:adequate:Exon 5:ExonStart false|STMQ

TAT GAA ATG TAC :Koz:adequate:Cav:weak:Exon 6:ExonStart false|TEMT

CCG CCC ATG AGT :Koz:weak:Cav:weak:Exon 6:ExonStart false|PPMS

AAT ACC ATG CGC :Koz:adequate:Cav:adequate:Exon 7:ExonStart false|NTMR

AAG CCA ATG CGT :Koz:weak:Cav:weak:Exon 7:ExonStart false|KPMR

CAG CAC ATG AGC :Koz:weak:Cav:weak:Exon 7:ExonStart false|QHMS***NonAcetSite**

ATT TCC ATG GCC :Koz:adequate:Cav:weak:Exon 7:ExonStart false|ISMA

AAC TAT ATG AAC :Koz:weak:Cav:adequate:Exon 7:ExonStart false|NTMN

GCA CAA ATG GAC :Koz:adequate:Cav:weak:Exon 7:ExonStart false|AQMD

GAG ATC ATG GAG :Koz:strong:Cav:adequate:Exon 8:ExonStart false|EIME

TCC AAA ATG GAG :Koz:strong:Cav:strong:Exon 8:ExonStart false|SKME

AAA TCG ATG GCC :Koz:adequate:Cav:weak:Exon 8:ExonStart false|KSMA

GAG ACC ATG CGC :Koz:adequate:Cav:adequate:Exon 8:ExonStart false|ETMR

GAA TTA ATG AAG :Koz:weak:Cav:weak:Exon 9:ExonStart true|ELMK

GAG CGC ATG CTG :Koz:weak:Cav:weak:Exon 9:ExonStart false|ERML

ATG CTG ATG AGG :Koz:weak:Cav:weak:Exon 9:ExonStart false|MLMR

CG3401-PA

TTC AAG ATG AGA :Koz:adequate:Cav:strong:Exon 1:ExonStart false|FKMR

GGA ACC ATG GAG :Koz:strong:Cav:adequate:Exon 3:ExonStart false|GTME

TCC GGA ATG GGC :Koz:strong:Cav:adequate:Exon 4:ExonStart false|SGMG***NonAcetSite**

CGC ATC ATG AAC :Koz:adequate:Cav:strong:Exon 4:ExonStart false|RIMN

CTG ACC ATG TCC :Koz:adequate:Cav:adequate:Exon 4:ExonStart false|LTMS

GTC AAC ATG GTT :Koz:strong:Cav:strong:Exon 4:ExonStart false|VNMV

TTC TTC ATG CCC :Koz:weak:Cav:adequate:Exon 4:ExonStart false|FFMP

CAG CAG ATG TTC :Koz:weak:Cav:weak:Exon 4:ExonStart false|QQMF

AAG AAC ATG ATG :Koz:adequate:Cav:adequate:Exon 4:ExonStart false|KNMM

AAC ATG ATG GCC :Koz:strong:Cav:strong:Exon 4:ExonStart false|NMMA

GGC CGC ATG TCC :Koz:weak:Cav:adequate:Exon 4:ExonStart false|GRMS

ATG TCC ATG AAG :Koz:weak:Cav:weak:Exon 4:ExonStart false|MSMK

GAG CAG ATG CTG :Koz:weak:Cav:weak:Exon 4:ExonStart false|EQML

CTG AAG ATG TCC :Koz:adequate:Cav:adequate:Exon 4:ExonStart false|LKMS

TCG GCC ATG TTC :Koz:adequate:Cav:weak:Exon 4:ExonStart false|SAMF

GAG GGC ATG GAC :Koz:strong:Cav:weak:Exon 4:ExonStart false|EGMD

GAC GAG ATG GAG :Koz:strong:Cav:adequate:Exon 4:ExonStart false|DEME

AGC AAC ATG AAC :Koz:adequate:Cav:strong:Exon 4:ExonStart false|SNMN

CG3403-PA

TGA AAC ATG AAG :Koz:adequate:Cav:adequate:Exon 1:ExonStart false|stopNMK*****

ATG AAG ATG GCT :Koz:strong:Cav:adequate:Exon 1:ExonStart false|MKMA***AcetSite**

GAG GAG ATG GAC :Koz:strong:Cav:weak:Exon 2:ExonStart false|EEMD

CTG ACC ATG CCC :Koz:adequate:Cav:adequate:Exon 2:ExonStart false|LTMP

TTC TGC ATG GAG :Koz:adequate:Cav:adequate:Exon 2:ExonStart false|FCME

ACC CAG ATG ACA :Koz:weak:Cav:adequate:Exon 2:ExonStart false|TQMT

AAT CTG ATG TCG :Koz:weak:Cav:weak:Exon 3:ExonStart false|NLMS

CG3430-PA

TAG ATA ATG CCC :Koz:adequate:Cav:adequate:Exon 1:ExonStart false|stopIMP

TGT GCG ATG GAG :Koz:strong:Cav:weak:Exon 1:ExonStart false|CAME***AcetSite**

CGT GGA ATG ATC :Koz:adequate:Cav:weak:Exon 1:ExonStart false|RGMI

CAG GAC ATG ATG :Koz:adequate:Cav:weak:Exon 1:ExonStart false|QDMM

GAC ATG ATG GAC :Koz:strong:Cav:strong:Exon 1:ExonStart false|DMMD

CGT ACC ATG TTC :Koz:adequate:Cav:adequate:Exon 1:ExonStart false|RTMF

GAA GCC ATG GAT :Koz:strong:Cav:weak:Exon 1:ExonStart false|EAMD

CCC AGC ATG GCA :Koz:strong:Cav:strong:Exon 1:ExonStart false|PSMA

GCA TGC ATG GTA :Koz:adequate:Cav:weak:Exon 1:ExonStart false|ACMV

CTG CGC ATG CTT :Koz:weak:Cav:weak:Exon 1:ExonStart false|LRML

TCT GAG ATG CAG :Koz:adequate:Cav:weak:Exon 1:ExonStart false|SEMQ

CAT TAC ATG CCC :Koz:weak:Cav:weak:Exon 1:ExonStart false|HTMP

ATG CCC ATG ACT :Koz:weak:Cav:weak:Exon 1:ExonStart false|MPMT

ACG TGC ATG CAA :Koz:weak:Cav:weak:Exon 2:ExonStart false|TCMQ

CGA AGT ATG TTG :Koz:adequate:Cav:adequate:Exon 2:ExonStart false|RSML

ACT GAA ATG ATC :Koz:adequate:Cav:weak:Exon 3:ExonStart false|TEMI

GTG GAC ATG CGT :Koz:adequate:Cav:weak:Exon 3:ExonStart false|VDMR

CG3579-PA

... ... ATG AGC :Koz:weak:Cav:weak:Exon 1:ExonStart false|nullnullMS

TCT GCA ATG GCC :Koz:strong:Cav:weak:Exon 1:ExonStart false|SAMA***AcetSite**

CCA AGT ATG GAG :Koz:strong:Cav:adequate:Exon 2:ExonStart false|PSME

GAG GAG ATG ATG :Koz:adequate:Cav:weak:Exon 2:ExonStart false|EEMM

GAG ATG ATG CGG :Koz:adequate:Cav:adequate:Exon 2:ExonStart false|EMMR

AAG GCC ATG CGC :Koz:adequate:Cav:weak:Exon 4:ExonStart false|KAMR

CTG CTG ATG ATT :Koz:weak:Cav:weak:Exon 4:ExonStart false|LLMI

CG3633-PA

TTC TCT ATG AAA :Koz:weak:Cav:adequate:Exon 1:ExonStart false|FSMK

ATG AAA ATG ATC :Koz:adequate:Cav:adequate:Exon 1:ExonStart false|MKMI

CAG CAA ATG TCC :Koz:weak:Cav:weak:Exon 1:ExonStart false|QQMS***NonAcetSite**

TCG GCG ATG CAG :Koz:adequate:Cav:weak:Exon 1:ExonStart false|SAMQ

TGT CTG ATG GTC :Koz:adequate:Cav:weak:Exon 1:ExonStart false|CLMV

CTG ACC ATG GCT :Koz:strong:Cav:adequate:Exon 1:ExonStart false|LTMA

AAC TGG ATG AAG :Koz:weak:Cav:adequate:Exon 1:ExonStart false|NWMK

GAG GGC ATG ATT :Koz:adequate:Cav:weak:Exon 1:ExonStart false|EGMI

AAA GTA ATG GTG :Koz:strong:Cav:weak:Exon 1:ExonStart false|KVMV

ATC GCA ATG ACT :Koz:adequate:Cav:adequate:Exon 1:ExonStart false|IAMT

GGC CAC ATG GAC :Koz:adequate:Cav:adequate:Exon 1:ExonStart false|GHMD

TCC TAT ATG CCG :Koz:weak:Cav:adequate:Exon 1:ExonStart false|STMP

AAG TTC ATG AGC :Koz:weak:Cav:weak:Exon 1:ExonStart false|KFMS

TCG CTG ATG CAG :Koz:weak:Cav:weak:Exon 1:ExonStart false|SLMQ

CG3752-PA

TTC AAA ATG CTG :Koz:adequate:Cav:strong:Exon 1:ExonStart false|FKML

CGT CGC ATG GAT :Koz:adequate:Cav:weak:Exon 3:ExonStart false|RRMD

GAT CTC ATG GAA :Koz:adequate:Cav:weak:Exon 3:ExonStart false|DLME

TAC AGC ATG TCC :Koz:adequate:Cav:strong:Exon 4:ExonStart false|TSMS***NonAcetSite**

ATT CCC ATG GAC :Koz:adequate:Cav:weak:Exon 4:ExonStart false|IPMD

ATC CTG ATG ATG :Koz:weak:Cav:adequate:Exon 4:ExonStart false|ILMM

CTG ATG ATG GCC :Koz:strong:Cav:adequate:Exon 4:ExonStart false|LMMA

ACC GAT ATG GAC :Koz:strong:Cav:adequate:Exon 4:ExonStart false|TDMD

TTT AAC ATG GGC :Koz:strong:Cav:adequate:Exon 4:ExonStart false|FNMG

GAG CAG ATG GAA :Koz:adequate:Cav:weak:Exon 4:ExonStart false|EQME

CTT GGA ATG ATC :Koz:adequate:Cav:weak:Exon 4:ExonStart false|LGMI

GAT GAT ATG ACC :Koz:adequate:Cav:weak:Exon 4:ExonStart false|DDMT

TAC AAG ATG TCC :Koz:adequate:Cav:strong:Exon 4:ExonStart false|TKMS

CG3835-PA

TCC ACG ATG AGA :Koz:adequate:Cav:strong:Exon 2:ExonStart false|STMR

ACC TTG ATG AGG :Koz:weak:Cav:adequate:Exon 2:ExonStart false|TLMR

CGC ACA ATG GCG :Koz:strong:Cav:strong:Exon 2:ExonStart false|RTMA***NonAcetSite**

GAC CTT ATG TCC :Koz:weak:Cav:adequate:Exon 4:ExonStart false|DLMS

TAC CAC ATG AAG :Koz:weak:Cav:adequate:Exon 4:ExonStart false|THMK

CTT TCG ATG CTC :Koz:weak:Cav:weak:Exon 4:ExonStart false|LSML

TTC TAC ATG CTC :Koz:weak:Cav:adequate:Exon 5:ExonStart false|FTML

GAC GGT ATG GAG :Koz:strong:Cav:adequate:Exon 5:ExonStart false|DGME

CGC GAA ATG GTG :Koz:strong:Cav:adequate:Exon 5:ExonStart false|REMV

GAC GTG ATG CGA :Koz:adequate:Cav:adequate:Exon 5:ExonStart false|DVMR

GGC TAC ATG CGC :Koz:weak:Cav:adequate:Exon 5:ExonStart false|GTMR

CGC GAG ATG AAG :Koz:adequate:Cav:adequate:Exon 5:ExonStart false|REMK

CG3835-PB

TCC ACG ATG AGA :Koz:adequate:Cav:strong:Exon 2:ExonStart false|STMR

ACC TTG ATG AGG :Koz:weak:Cav:adequate:Exon 2:ExonStart false|TLMR

CGC ACA ATG GCG :Koz:strong:Cav:strong:Exon 2:ExonStart false|RTMA***NonAcetSite**

GAC CTT ATG TCC :Koz:weak:Cav:adequate:Exon 4:ExonStart false|DLMS

TAC CAC ATG AAG :Koz:weak:Cav:adequate:Exon 4:ExonStart false|THMK

CTT TCG ATG CTC :Koz:weak:Cav:weak:Exon 4:ExonStart false|LSML

TTC TAC ATG CTC :Koz:weak:Cav:adequate:Exon 5:ExonStart false|FTML

GAC GGT ATG GAG :Koz:strong:Cav:adequate:Exon 5:ExonStart false|DGME

CGC GAA ATG GTG :Koz:strong:Cav:adequate:Exon 5:ExonStart false|REMV

GAC GTG ATG CGA :Koz:adequate:Cav:adequate:Exon 5:ExonStart false|DVMR

GGC TAC ATG CGC :Koz:weak:Cav:adequate:Exon 5:ExonStart false|GTMR

CGC GAG ATG AAG :Koz:adequate:Cav:adequate:Exon 5:ExonStart false|REMK

CG3835-PC

TCC ACG ATG AGA :Koz:adequate:Cav:strong:Exon 2:ExonStart false|STMR

ACC TTG ATG AGG :Koz:weak:Cav:adequate:Exon 2:ExonStart false|TLMR

CGC ACA ATG GCG :Koz:strong:Cav:strong:Exon 2:ExonStart false|RTMA***NonAcetSite**

GAC CTT ATG TCC :Koz:weak:Cav:adequate:Exon 4:ExonStart false|DLMS

TAC CAC ATG AAG :Koz:weak:Cav:adequate:Exon 4:ExonStart false|THMK

CTT TCG ATG CTC :Koz:weak:Cav:weak:Exon 4:ExonStart false|LSML

TTC TAC ATG CTC :Koz:weak:Cav:adequate:Exon 5:ExonStart false|FTML

GAC GGT ATG GAG :Koz:strong:Cav:adequate:Exon 5:ExonStart false|DGME

CGC GAA ATG GTG :Koz:strong:Cav:adequate:Exon 5:ExonStart false|REMV

GAC GTG ATG CGA :Koz:adequate:Cav:adequate:Exon 5:ExonStart false|DVMR

GGC TAC ATG CGC :Koz:weak:Cav:adequate:Exon 5:ExonStart false|GTMR

CGC GAG ATG AAG :Koz:adequate:Cav:adequate:Exon 5:ExonStart false|REMK

CG4027-PA

TAC AAA ATG TGT :Koz:adequate:Cav:strong:Exon 2:ExonStart false|TKMC

TCT GGC ATG TGC :Koz:adequate:Cav:weak:Exon 2:ExonStart false|SGMC***NonAcetSite**

GGT GTG ATG GTC :Koz:strong:Cav:weak:Exon 2:ExonStart false|GVMV

GTC GGC ATG GGC :Koz:strong:Cav:adequate:Exon 2:ExonStart false|VGMG***NonAcetSite**

GAC GAT ATG GAG :Koz:strong:Cav:adequate:Exon 2:ExonStart false|DDME

GAG AAG ATG ACC :Koz:adequate:Cav:adequate:Exon 2:ExonStart false|EKMT

CAG ATC ATG TTC :Koz:adequate:Cav:adequate:Exon 2:ExonStart false|QIMF

CCC GCC ATG TAT :Koz:adequate:Cav:adequate:Exon 2:ExonStart false|PAMT

TAC CTG ATG AAG :Koz:weak:Cav:adequate:Exon 2:ExonStart false|TLMK

CAG GAG ATG GCC :Koz:strong:Cav:weak:Exon 2:ExonStart false|QEMA

TTG GGA ATG GAG :Koz:strong:Cav:weak:Exon 2:ExonStart false|LGME

TCC ATC ATG AAG :Koz:adequate:Cav:strong:Exon 2:ExonStart false|SIMK

ACC ACC ATG TAC :Koz:adequate:Cav:strong:Exon 2:ExonStart false|TTMT

GAC CGT ATG CAG :Koz:weak:Cav:adequate:Exon 2:ExonStart false|DRMQ

TCG ACC ATG AAG :Koz:adequate:Cav:adequate:Exon 2:ExonStart false|STMK

CAG CAG ATG TGG :Koz:weak:Cav:weak:Exon 2:ExonStart false|QQMW

CG4027-PB

TAC AAA ATG TGT :Koz:adequate:Cav:strong:Exon 2:ExonStart false|TKMC

TCT GGC ATG TGC :Koz:adequate:Cav:weak:Exon 2:ExonStart false|SGMC***NonAcetSite**

GGT GTG ATG GTC :Koz:strong:Cav:weak:Exon 2:ExonStart false|GVMV

GTC GGC ATG GGC :Koz:strong:Cav:adequate:Exon 2:ExonStart false|VGMG***NonAcetSite**

GAC GAT ATG GAG :Koz:strong:Cav:adequate:Exon 2:ExonStart false|DDME

GAG AAG ATG ACC :Koz:adequate:Cav:adequate:Exon 2:ExonStart false|EKMT

CAG ATC ATG TTC :Koz:adequate:Cav:adequate:Exon 2:ExonStart false|QIMF

CCC GCC ATG TAT :Koz:adequate:Cav:adequate:Exon 2:ExonStart false|PAMT

TAC CTG ATG AAG :Koz:weak:Cav:adequate:Exon 2:ExonStart false|TLMK

CAG GAG ATG GCC :Koz:strong:Cav:weak:Exon 2:ExonStart false|QEMA

TTG GGA ATG GAG :Koz:strong:Cav:weak:Exon 2:ExonStart false|LGME

TCC ATC ATG AAG :Koz:adequate:Cav:strong:Exon 2:ExonStart false|SIMK

ACC ACC ATG TAC :Koz:adequate:Cav:strong:Exon 2:ExonStart false|TTMT

GAC CGT ATG CAG :Koz:weak:Cav:adequate:Exon 2:ExonStart false|DRMQ

TCG ACC ATG AAG :Koz:adequate:Cav:adequate:Exon 2:ExonStart false|STMK

CAG CAG ATG TGG :Koz:weak:Cav:weak:Exon 2:ExonStart false|QQMW

CG4043-PA

TAT AAT ATG AAT :Koz:adequate:Cav:adequate:Exon 1:ExonStart false|TNMN

GCG AAA ATG GAC :Koz:strong:Cav:adequate:Exon 1:ExonStart false|AKMD***AcetSite**

CGC CAG ATG CAC :Koz:weak:Cav:adequate:Exon 1:ExonStart false|RQMH

TCC GTG ATG TAC :Koz:adequate:Cav:adequate:Exon 1:ExonStart false|SVMT

TCC AAG ATG TCC :Koz:adequate:Cav:strong:Exon 2:ExonStart false|SKMS

CTA GCC ATG CTC :Koz:adequate:Cav:weak:Exon 3:ExonStart false|LAML

CG4098-PA

AGT CCA ATG ACG :Koz:weak:Cav:weak:Exon 1:ExonStart false|SPMT

GCC CAA ATG ATG :Koz:weak:Cav:adequate:Exon 1:ExonStart false|AQMM***AcetSite**

CAA ATG ATG TCA :Koz:adequate:Cav:adequate:Exon 1:ExonStart false|QMMS

CAC CTA ATG TGC :Koz:weak:Cav:adequate:Exon 1:ExonStart false|HLMC

AAC AAC ATG TAT :Koz:adequate:Cav:strong:Exon 1:ExonStart false|NNMT

ATA CAG ATG GTT :Koz:adequate:Cav:weak:Exon 2:ExonStart false|IQMV

GGT GGA ATG GTC :Koz:strong:Cav:weak:Exon 2:ExonStart false|GGMV

GCC AAC ATG GTT :Koz:strong:Cav:strong:Exon 2:ExonStart false|ANMV

GCT TGG ATG GAA :Koz:adequate:Cav:weak:Exon 2:ExonStart false|AWME

GAG CTA ATG GCC :Koz:adequate:Cav:weak:Exon 2:ExonStart false|ELMA

CG4241-PA

TCT GCG ATG AGC :Koz:adequate:Cav:weak:Exon 2:ExonStart false|SAMS

ATC GCC ATG TCC :Koz:adequate:Cav:adequate:Exon 2:ExonStart false|IAMS

AGC ACG ATG ACG :Koz:adequate:Cav:strong:Exon 2:ExonStart false|STMT***NonAcetSite**

ACA CCC ATG CGC :Koz:weak:Cav:weak:Exon 2:ExonStart false|TPMR

GCC ACG ATG GCC :Koz:strong:Cav:strong:Exon 3:ExonStart false|ATMA

GCC CGC ATG GCC :Koz:adequate:Cav:adequate:Exon 4:ExonStart false|ARMA

CGA AGA ATG CAG :Koz:adequate:Cav:adequate:Exon 5:ExonStart false|RRMQ

CAG ACA ATG CGG :Koz:adequate:Cav:adequate:Exon 5:ExonStart false|QTMR

CTT AGC ATG AAC :Koz:adequate:Cav:adequate:Exon 6:ExonStart false|LSMN

CG4241-PC

TCT GCG ATG AGC :Koz:adequate:Cav:weak:Exon 2:ExonStart false|SAMS

ATC GCC ATG TCC :Koz:adequate:Cav:adequate:Exon 2:ExonStart false|IAMS

AGC ACG ATG ACG :Koz:adequate:Cav:strong:Exon 2:ExonStart false|STMT***NonAcetSite**

ACA CCC ATG CGC :Koz:weak:Cav:weak:Exon 2:ExonStart false|TPMR

GCC ACG ATG GCC :Koz:strong:Cav:strong:Exon 3:ExonStart false|ATMA

GCC CGC ATG GCC :Koz:adequate:Cav:adequate:Exon 4:ExonStart false|ARMA

CGA AGA ATG CAG :Koz:adequate:Cav:adequate:Exon 5:ExonStart false|RRMQ

CAG ACA ATG CGG :Koz:adequate:Cav:adequate:Exon 5:ExonStart false|QTMR

CTT AGC ATG AAC :Koz:adequate:Cav:adequate:Exon 6:ExonStart false|LSMN

CG4289-PA

ATA CAG ATG TCC :Koz:weak:Cav:weak:Exon 1:ExonStart false|IQMS

ACC GTC ATG GCG :Koz:strong:Cav:adequate:Exon 1:ExonStart false|TVMA***AcetSite**

GAG ATC ATG TAA :Koz:adequate:Cav:adequate:Exon 3:ExonStart false|EIMstop

CG4428-PA

CAG TAG ATG CTG :Koz:weak:Cav:weak:Exon 1:ExonStart false|QstopML

CGG ATC ATG GAG :Koz:strong:Cav:adequate:Exon 1:ExonStart false|RIME***AcetSite**

GGC TGC ATG CTG :Koz:weak:Cav:adequate:Exon 2:ExonStart false|GCML

GGT CAG ATG GTT :Koz:adequate:Cav:weak:Exon 2:ExonStart false|GQMV

GCC CAG ATG GGA :Koz:adequate:Cav:adequate:Exon 2:ExonStart false|AQMG

GTG GCC ATG GAT :Koz:strong:Cav:weak:Exon 3:ExonStart false|VAMD

TGT GGC ATG ATC :Koz:adequate:Cav:weak:Exon 4:ExonStart false|CGMI

TCC GCC ATG GAC :Koz:strong:Cav:adequate:Exon 4:ExonStart false|SAMD

CCC ACC ATG CCG :Koz:adequate:Cav:strong:Exon 4:ExonStart false|PTMP

CG4645-PA

ATC TAA ATG GAG :Koz:adequate:Cav:adequate:Exon 1:ExonStart false|IstopME*****

ACA CCC ATG GCC :Koz:adequate:Cav:weak:Exon 1:ExonStart false|TPMA***AcetSite**

TAT GAC ATG ACC :Koz:adequate:Cav:weak:Exon 1:ExonStart false|TDMT

ACC TAT ATG GTG :Koz:adequate:Cav:adequate:Exon 1:ExonStart false|TTMV

AAC TCC ATG ATA :Koz:weak:Cav:adequate:Exon 1:ExonStart false|NSMI

CTG CGC ATG AAC :Koz:weak:Cav:weak:Exon 1:ExonStart false|LRMN

AGC TAC ATG CCG :Koz:weak:Cav:adequate:Exon 3:ExonStart false|STMP

TCG CTG ATG TGC :Koz:weak:Cav:weak:Exon 3:ExonStart false|SLMC

CG4930-PA

AGG GGA ATG TCC :Koz:adequate:Cav:weak:Exon 1:ExonStart false|RGMS

GAC AAA ATG GCA :Koz:strong:Cav:strong:Exon 1:ExonStart false|DKMA***AcetSite**

ACT CTA ATG AAG :Koz:weak:Cav:weak:Exon 2:ExonStart false|TLMK

GAC AAG ATG GTG :Koz:strong:Cav:strong:Exon 2:ExonStart false|DKMV

GTG AAA ATG GAA :Koz:strong:Cav:adequate:Exon 2:ExonStart false|VKME

CG5073-PA

GTA GCA ATG ACA :Koz:adequate:Cav:weak:Exon 1:ExonStart false|VAMT

ATG ACA ATG GGC :Koz:strong:Cav:adequate:Exon 1:ExonStart false|MTMG***AcetSite**

CAA ATA ATG CTG :Koz:adequate:Cav:adequate:Exon 5:ExonStart false|QIML

CG5178-PA

GCC AAG ATG TGT :Koz:adequate:Cav:strong:Exon 2:ExonStart false|AKMC

TCG GGC ATG TGC :Koz:adequate:Cav:weak:Exon 2:ExonStart false|SGMC***NonAcetSite**

GGT GTG ATG GTG :Koz:strong:Cav:weak:Exon 2:ExonStart false|GVMV

GTG GGT ATG GGT :Koz:strong:Cav:weak:Exon 2:ExonStart false|VGMG***NonAcetSite**

GAC GAC ATG GAG :Koz:strong:Cav:adequate:Exon 2:ExonStart false|DDME

GAG AAG ATG ACC :Koz:adequate:Cav:adequate:Exon 2:ExonStart false|EKMT

CAG ATC ATG TTC :Koz:adequate:Cav:adequate:Exon 2:ExonStart false|QIMF

CCG GCC ATG TAC :Koz:adequate:Cav:weak:Exon 2:ExonStart false|PAMT

TAC CTG ATG AAG :Koz:weak:Cav:adequate:Exon 2:ExonStart false|TLMK

CAG GAG ATG GCC :Koz:strong:Cav:weak:Exon 2:ExonStart false|QEMA

CTG GGC ATG GAG :Koz:strong:Cav:weak:Exon 2:ExonStart false|LGME

TCG ATC ATG AAG :Koz:adequate:Cav:adequate:Exon 2:ExonStart false|SIMK

ACC ACC ATG TAC :Koz:adequate:Cav:strong:Exon 2:ExonStart false|TTMT

GAT CGT ATG CAG :Koz:weak:Cav:weak:Exon 3:ExonStart false|DRMQ

CAG CAG ATG TGG :Koz:weak:Cav:weak:Exon 3:ExonStart false|QQMW

CG5355-PA

TTG CAA ATG CAT :Koz:weak:Cav:weak:Exon 1:ExonStart false|LQMH

AGA GCC ATG CCA :Koz:adequate:Cav:weak:Exon 2:ExonStart false|RAMP***NonAcetSite**

TGT CCC ATG CGT :Koz:weak:Cav:weak:Exon 2:ExonStart false|CPMR

TAC TTT ATG AAC :Koz:weak:Cav:adequate:Exon 2:ExonStart false|TFMN

AGC GTC ATG TAC :Koz:adequate:Cav:adequate:Exon 2:ExonStart false|SVMT

AAG TAC ATG GCC :Koz:adequate:Cav:weak:Exon 2:ExonStart false|KTMA

GTT CCC ATG TTC :Koz:weak:Cav:weak:Exon 7:ExonStart false|VPMF

ATC AGC ATG CTG :Koz:adequate:Cav:strong:Exon 7:ExonStart false|ISML

GGG CTT ATG TTC :Koz:weak:Cav:weak:Exon 8:ExonStart false|GLMF

GGT GTC ATG GAT :Koz:strong:Cav:weak:Exon 9:ExonStart false|GVMD

ATG GAT ATG TTG :Koz:adequate:Cav:weak:Exon 9:ExonStart false|MDML

CG5374-PA

AGG AAA ATG TCG :Koz:adequate:Cav:adequate:Exon 1:ExonStart false|RKMS*****

AAC GTT ATG GCC :Koz:strong:Cav:adequate:Exon 2:ExonStart false|NVMA

GAC AAG ATG CTG :Koz:adequate:Cav:strong:Exon 2:ExonStart false|DKML

ACC TCG ATG AGC :Koz:weak:Cav:adequate:Exon 3:ExonStart false|TSMS

TCA GCC ATG GTT :Koz:strong:Cav:weak:Exon 3:ExonStart false|SAMV

CAG CAG ATG CCC :Koz:weak:Cav:weak:Exon 3:ExonStart false|QQMP

ACC AAG ATG AAG :Koz:adequate:Cav:strong:Exon 3:ExonStart false|TKMK

ATG AAG ATG GGT :Koz:strong:Cav:adequate:Exon 3:ExonStart false|MKMG***NonAcetSite**

ATC AAC ATG ATC :Koz:adequate:Cav:strong:Exon 3:ExonStart false|INMI

TTG TGC ATG AAG :Koz:weak:Cav:weak:Exon 3:ExonStart false|LCMK

GGA GCC ATG GCT :Koz:strong:Cav:weak:Exon 3:ExonStart false|GAMA

ACC AAC ATG GAC :Koz:strong:Cav:strong:Exon 3:ExonStart false|TNMD

GCC TCA ATG GTG :Koz:adequate:Cav:adequate:Exon 3:ExonStart false|ASMV

GAT GAG ATG GAG :Koz:strong:Cav:weak:Exon 4:ExonStart false|DEME

CCC GCC ATG TCC :Koz:adequate:Cav:adequate:Exon 4:ExonStart false|PAMS

GAC GAC ATG ATC :Koz:adequate:Cav:adequate:Exon 4:ExonStart false|DDMI

CG5374-PB

AGG AAA ATG TCG :Koz:adequate:Cav:adequate:Exon 2:ExonStart false|RKMS*****

AAC GTT ATG GCC :Koz:strong:Cav:adequate:Exon 3:ExonStart false|NVMA

GAC AAG ATG CTG :Koz:adequate:Cav:strong:Exon 3:ExonStart false|DKML

ACC TCG ATG AGC :Koz:weak:Cav:adequate:Exon 4:ExonStart false|TSMS

TCA GCC ATG GTT :Koz:strong:Cav:weak:Exon 4:ExonStart false|SAMV

CAG CAG ATG CCC :Koz:weak:Cav:weak:Exon 4:ExonStart false|QQMP

ACC AAG ATG AAG :Koz:adequate:Cav:strong:Exon 4:ExonStart false|TKMK

ATG AAG ATG GGT :Koz:strong:Cav:adequate:Exon 4:ExonStart false|MKMG***NonAcetSite**

ATC AAC ATG ATC :Koz:adequate:Cav:strong:Exon 4:ExonStart false|INMI

TTG TGC ATG AAG :Koz:weak:Cav:weak:Exon 4:ExonStart false|LCMK

GGA GCC ATG GCT :Koz:strong:Cav:weak:Exon 4:ExonStart false|GAMA

ACC AAC ATG GAC :Koz:strong:Cav:strong:Exon 4:ExonStart false|TNMD

GCC TCA ATG GTG :Koz:adequate:Cav:adequate:Exon 4:ExonStart false|ASMV

GAT GAG ATG GAG :Koz:strong:Cav:weak:Exon 5:ExonStart false|DEME

CCC GCC ATG TCC :Koz:adequate:Cav:adequate:Exon 5:ExonStart false|PAMS

GAC GAC ATG ATC :Koz:adequate:Cav:adequate:Exon 5:ExonStart false|DDMI

CG5417-PA

... ... ATG CCA :Koz:weak:Cav:weak:Exon 1:ExonStart false|nullnullMP

CAC GAA ATG GTT :Koz:strong:Cav:adequate:Exon 2:ExonStart false|HEMV***NonAcetSite**

ACC TAC ATG TGC :Koz:weak:Cav:adequate:Exon 4:ExonStart false|TTMC

TGC CTG ATG CGC :Koz:weak:Cav:adequate:Exon 4:ExonStart false|CLMR

CCC GCC ATG ATG :Koz:adequate:Cav:adequate:Exon 5:ExonStart false|PAMM

GCC ATG ATG AGC :Koz:adequate:Cav:strong:Exon 5:ExonStart false|AMMS

ATG AGC ATG TAC :Koz:adequate:Cav:adequate:Exon 5:ExonStart false|MSMT

CAG TTC ATG AAG :Koz:weak:Cav:weak:Exon 5:ExonStart false|QFMK

AGC AAG ATG GAC :Koz:strong:Cav:strong:Exon 5:ExonStart false|SKMD

CG5433-PA

GGC AAA ATG ACG :Koz:adequate:Cav:strong:Exon 2:ExonStart false|GKMT*****

ACG CAA ATG TCG :Koz:weak:Cav:weak:Exon 2:ExonStart false|TQMS***AcetSite**

TCG ATT ATG AAC :Koz:adequate:Cav:adequate:Exon 2:ExonStart false|SIMN

TCG GAC ATG CTG :Koz:adequate:Cav:weak:Exon 2:ExonStart false|SDML

CAG GTG ATG ATG :Koz:adequate:Cav:weak:Exon 2:ExonStart false|QVMM

GTG ATG ATG GCC :Koz:strong:Cav:adequate:Exon 2:ExonStart false|VMMA

GAG TTC ATG GCG :Koz:adequate:Cav:weak:Exon 2:ExonStart false|EFMA

CAC AAC ATG TCG :Koz:adequate:Cav:strong:Exon 2:ExonStart false|HNMS

GCA ACC ATG CTG :Koz:adequate:Cav:adequate:Exon 3:ExonStart false|ATML

CAA GGC ATG TTT :Koz:adequate:Cav:weak:Exon 5:ExonStart false|QGMF

TGT GCA ATG CGG :Koz:adequate:Cav:weak:Exon 5:ExonStart false|CAMR

CG5436-PA

GCC AAG ATG CCA :Koz:adequate:Cav:strong:Exon 1:ExonStart false|AKMP

GTG GCC ATG AAC :Koz:adequate:Cav:weak:Exon 1:ExonStart false|VAMN

AGC TCG ATG GTA :Koz:adequate:Cav:adequate:Exon 1:ExonStart false|SSMV

ACC AAA ATG AAG :Koz:adequate:Cav:strong:Exon 1:ExonStart false|TKMK

GCT AAA ATG GAC :Koz:strong:Cav:adequate:Exon 1:ExonStart false|AKMD

GGG GTG ATG ACC :Koz:adequate:Cav:weak:Exon 1:ExonStart false|GVMT

GAC CGC ATG CTC :Koz:weak:Cav:adequate:Exon 1:ExonStart false|DRML

GAG GCG ATG AAG :Koz:adequate:Cav:weak:Exon 1:ExonStart false|EAMK

CCC ATC ATG ACC :Koz:adequate:Cav:strong:Exon 1:ExonStart false|PIMT

ACC AAG ATG CAC :Koz:adequate:Cav:strong:Exon 1:ExonStart false|TKMH***NonAcetSite**

CG5517-PA

AAC TCA ATG TAT :Koz:weak:Cav:adequate:Exon 1:ExonStart false|NSMT

CCC AAG ATG ACA :Koz:adequate:Cav:strong:Exon 2:ExonStart false|PKMT***NonAcetSite**

GAC AGC ATG GAG :Koz:strong:Cav:strong:Exon 2:ExonStart false|DSME

GGC CAC ATG TCT :Koz:weak:Cav:adequate:Exon 4:ExonStart false|GHMS

GAG CAC ATG CTG :Koz:weak:Cav:weak:Exon 4:ExonStart false|EHML

CCC CTG ATG ACC :Koz:weak:Cav:adequate:Exon 4:ExonStart false|PLMT

AAC ATA ATG TGC :Koz:adequate:Cav:strong:Exon 5:ExonStart false|NIMC

GAG GGC ATG GTA :Koz:strong:Cav:weak:Exon 6:ExonStart false|EGMV

GAT CTT ATG GCC :Koz:adequate:Cav:weak:Exon 8:ExonStart false|DLMA

TTG GAA ATG CTC :Koz:adequate:Cav:weak:Exon 8:ExonStart false|LEML

AAC GAA ATG AGA :Koz:adequate:Cav:adequate:Exon 8:ExonStart false|NEMR

TCG TCC ATG CAA :Koz:weak:Cav:weak:Exon 8:ExonStart false|SSMQ

GCA TGC ATG ACT :Koz:weak:Cav:weak:Exon 9:ExonStart false|ACMT

TTC GAC ATG TCC :Koz:adequate:Cav:adequate:Exon 9:ExonStart false|FDMS

AAC CAT ATG ATG :Koz:weak:Cav:adequate:Exon 9:ExonStart false|NHMM

CAT ATG ATG GTG :Koz:strong:Cav:adequate:Exon 9:ExonStart false|HMMV

ATG GTG ATG CTG :Koz:adequate:Cav:weak:Exon 9:ExonStart false|MVML

AGT GTG ATG GGA :Koz:strong:Cav:weak:Exon 9:ExonStart false|SVMG

GCC AAT ATG GAG :Koz:strong:Cav:strong:Exon 10:ExonStart false|ANME

GAC GCT ATG GAA :Koz:strong:Cav:adequate:Exon 10:ExonStart false|DAME

CGG CAA ATG CTC :Koz:weak:Cav:weak:Exon 11:ExonStart false|RQML

AAC ATA ATG GTA :Koz:strong:Cav:strong:Exon 12:ExonStart false|NIMV

GAG GAC ATG CCA :Koz:adequate:Cav:weak:Exon 13:ExonStart false|EDMP

ATA GCC ATG CAG :Koz:adequate:Cav:weak:Exon 13:ExonStart false|IAMQ

ACA AAC ATG GAG :Koz:strong:Cav:adequate:Exon 14:ExonStart false|TNME

CG5625-PA

GCA GTA ATG ACC :Koz:adequate:Cav:weak:Exon 1:ExonStart false|AVMT*****

ATG ACC ATG CCG :Koz:adequate:Cav:adequate:Exon 2:ExonStart false|MTMP***NonAcetSite**

TTT CAA ATG AAC :Koz:weak:Cav:weak:Exon 2:ExonStart false|FQMN

GAG CGT ATG CTG :Koz:weak:Cav:weak:Exon 2:ExonStart false|ERML

AGC ACC ATG CTG :Koz:adequate:Cav:strong:Exon 2:ExonStart false|STML

CTG TAC ATG GCC :Koz:adequate:Cav:weak:Exon 3:ExonStart false|LTMA

GTC GAG ATG TGC :Koz:adequate:Cav:adequate:Exon 3:ExonStart false|VEMC

GAT GTG ATG GTT :Koz:strong:Cav:weak:Exon 3:ExonStart false|DVMV

GCC GAG ATG AAC :Koz:adequate:Cav:adequate:Exon 3:ExonStart false|AEMN

GTC CGC ATG CAG :Koz:weak:Cav:adequate:Exon 3:ExonStart false|VRMQ

TAC CTA ATG GAG :Koz:adequate:Cav:adequate:Exon 3:ExonStart false|TLME

ACC CGA ATG GAC :Koz:adequate:Cav:adequate:Exon 4:ExonStart false|TRMD

ATG GAC ATG CCG :Koz:adequate:Cav:weak:Exon 4:ExonStart false|MDMP

CAG CGC ATG AAC :Koz:weak:Cav:weak:Exon 4:ExonStart false|QRMN

ATG AAC ATG AAC :Koz:adequate:Cav:adequate:Exon 4:ExonStart false|MNMN

CTA GTT ATG AAC :Koz:adequate:Cav:weak:Exon 5:ExonStart false|LVMN

CAC CTC ATG CGC :Koz:weak:Cav:adequate:Exon 6:ExonStart false|HLMR

CCG GAC ATG CAG :Koz:adequate:Cav:weak:Exon 6:ExonStart false|PDMQ

TAT AAA ATG CTG :Koz:adequate:Cav:adequate:Exon 6:ExonStart false|TKML

CG5625-PB

ACG TCC ATG TAT :Koz:weak:Cav:weak:Exon 1:ExonStart false|TSMT

CGG ACC ATG CCG :Koz:adequate:Cav:adequate:Exon 2:ExonStart false|RTMP***NonAcetSite**

TTT CAA ATG AAC :Koz:weak:Cav:weak:Exon 2:ExonStart false|FQMN

GAG CGT ATG CTG :Koz:weak:Cav:weak:Exon 2:ExonStart false|ERML

AGC ACC ATG CTG :Koz:adequate:Cav:strong:Exon 2:ExonStart false|STML

CTG TAC ATG GCC :Koz:adequate:Cav:weak:Exon 3:ExonStart false|LTMA

GTC GAG ATG TGC :Koz:adequate:Cav:adequate:Exon 3:ExonStart false|VEMC

GAT GTG ATG GTT :Koz:strong:Cav:weak:Exon 3:ExonStart false|DVMV

GCC GAG ATG AAC :Koz:adequate:Cav:adequate:Exon 3:ExonStart false|AEMN

GTC CGC ATG CAG :Koz:weak:Cav:adequate:Exon 3:ExonStart false|VRMQ

TAC CTA ATG GAG :Koz:adequate:Cav:adequate:Exon 3:ExonStart false|TLME

ACC CGA ATG GAC :Koz:adequate:Cav:adequate:Exon 4:ExonStart false|TRMD

ATG GAC ATG CCG :Koz:adequate:Cav:weak:Exon 4:ExonStart false|MDMP

CAG CGC ATG AAC :Koz:weak:Cav:weak:Exon 4:ExonStart false|QRMN

ATG AAC ATG AAC :Koz:adequate:Cav:adequate:Exon 4:ExonStart false|MNMN

CTA GTT ATG AAC :Koz:adequate:Cav:weak:Exon 5:ExonStart false|LVMN

CAC CTC ATG CGC :Koz:weak:Cav:adequate:Exon 6:ExonStart false|HLMR

CCG GAC ATG CAG :Koz:adequate:Cav:weak:Exon 6:ExonStart false|PDMQ

TAT AAA ATG CTG :Koz:adequate:Cav:adequate:Exon 6:ExonStart false|TKML

CG5800-PA

GAG GGT ATG CAG :Koz:adequate:Cav:weak:Exon 1:ExonStart false|EGMQ

CTG TTC ATG AAC :Koz:weak:Cav:weak:Exon 2:ExonStart false|LFMN

ACT CGC ATG GAC :Koz:adequate:Cav:weak:Exon 2:ExonStart false|TRMD

CAG CAC ATG GAC :Koz:adequate:Cav:weak:Exon 2:ExonStart false|QHMD

AGC ACC ATG GAG :Koz:strong:Cav:strong:Exon 2:ExonStart false|STME***NonAcetSite**

ATG GAG ATG TTG :Koz:adequate:Cav:weak:Exon 2:ExonStart false|MEML

TTG GAT ATG GGT :Koz:strong:Cav:weak:Exon 2:ExonStart false|LDMG

ATC ACC ATG CTG :Koz:adequate:Cav:strong:Exon 2:ExonStart false|ITML

GTC GTT ATG TTC :Koz:adequate:Cav:adequate:Exon 2:ExonStart false|VVMF

GAG TAC ATG ATT :Koz:weak:Cav:weak:Exon 2:ExonStart false|ETMI

TTC CTG ATG CGC :Koz:weak:Cav:adequate:Exon 2:ExonStart false|FLMR

GAA ACT ATG GCA :Koz:strong:Cav:adequate:Exon 2:ExonStart false|ETMA

AGC CAA ATG AAG :Koz:weak:Cav:adequate:Exon 2:ExonStart false|SQMK

AAC AAG ATG ACT :Koz:adequate:Cav:strong:Exon 2:ExonStart false|NKMT

ACT CTA ATG GAC :Koz:adequate:Cav:weak:Exon 2:ExonStart false|TLMD

CG5972-PA

GAC ACA ATG GCA :Koz:strong:Cav:strong:Exon 1:ExonStart false|DTMA*****

GCG GCG ATG TGC :Koz:adequate:Cav:weak:Exon 1:ExonStart false|AAMC***NonAcetSite**

AGG TTC ATG ATG :Koz:weak:Cav:weak:Exon 1:ExonStart false|RFMM

TTC ATG ATG CGA :Koz:adequate:Cav:strong:Exon 1:ExonStart false|FMMR

GAG CAG ATG TAT :Koz:weak:Cav:weak:Exon 1:ExonStart false|EQMT

AGC TTC ATG GAG :Koz:adequate:Cav:adequate:Exon 1:ExonStart false|SFME

AGC GAA ATG AAA :Koz:adequate:Cav:adequate:Exon 1:ExonStart false|SEMK

CG6255-PA

CAC AAA ATG TCA :Koz:adequate:Cav:strong:Exon 1:ExonStart false|HKMS

AGC AAG ATG CTC :Koz:adequate:Cav:strong:Exon 1:ExonStart false|SKML

GCG GAT ATG GTG :Koz:strong:Cav:weak:Exon 1:ExonStart false|ADMV

TCG CAA ATG CTG :Koz:weak:Cav:weak:Exon 1:ExonStart false|SQML

GGA ATA ATG CCG :Koz:adequate:Cav:adequate:Exon 1:ExonStart false|GIMP

ATT GTC ATG ATT :Koz:adequate:Cav:weak:Exon 1:ExonStart false|IVMI

CGT CGA ATG GGT :Koz:adequate:Cav:weak:Exon 1:ExonStart false|RRMG***AcetSite**

GTC CGT ATG ACC :Koz:weak:Cav:adequate:Exon 1:ExonStart false|VRMT

GAG GAA ATG ATC :Koz:adequate:Cav:weak:Exon 1:ExonStart false|EEMI

CG6359-PA

ACC ACT ATG ATG :Koz:adequate:Cav:strong:Exon 1:ExonStart false|TTMM*****

ACT ATG ATG GTT :Koz:strong:Cav:adequate:Exon 1:ExonStart false|TMMV***NonAcetSite**

ACC ACC ATG GCG :Koz:strong:Cav:strong:Exon 1:ExonStart false|TTMA

GTC CGG ATG CGG :Koz:weak:Cav:adequate:Exon 1:ExonStart false|VRMR

CGG CAG ATG CCC :Koz:weak:Cav:weak:Exon 3:ExonStart false|RQMP

CTG CAT ATG TTC :Koz:weak:Cav:weak:Exon 3:ExonStart false|LHMF

CG6767-PA

TGT GTG ATG CCA :Koz:adequate:Cav:weak:Exon 2:ExonStart false|CVMP

TCC AGA ATG CCG :Koz:adequate:Cav:strong:Exon 2:ExonStart false|SRMP***NonAcetSite**

AAC TTG ATG GAG :Koz:adequate:Cav:adequate:Exon 3:ExonStart false|NLME

CTG ATC ATG ATC :Koz:adequate:Cav:adequate:Exon 3:ExonStart false|LIMI

GCC AAC ATG CTG :Koz:adequate:Cav:strong:Exon 4:ExonStart false|ANML

ATC ACC ATG GAT :Koz:strong:Cav:strong:Exon 4:ExonStart false|ITMD

GCC TCT ATG GTA :Koz:adequate:Cav:adequate:Exon 6:ExonStart false|ASMV

GAT GAC ATG GCC :Koz:strong:Cav:weak:Exon 6:ExonStart false|DDMA

GGA CAC ATG CGG :Koz:weak:Cav:weak:Exon 6:ExonStart false|GHMR

GTC TCG ATG ATG :Koz:weak:Cav:adequate:Exon 7:ExonStart false|VSMM

TCG ATG ATG TTC :Koz:adequate:Cav:adequate:Exon 7:ExonStart false|SMMF

CG6767-PB

TGA ATA ATG CCC :Koz:adequate:Cav:adequate:Exon 1:ExonStart false|stopIMP

TGT GTG ATG CCA :Koz:adequate:Cav:weak:Exon 2:ExonStart false|CVMP

TCC AGA ATG CCG :Koz:adequate:Cav:strong:Exon 2:ExonStart false|SRMP***NonAcetSite**

AAC TTG ATG GAG :Koz:adequate:Cav:adequate:Exon 3:ExonStart false|NLME

CTG ATC ATG ATC :Koz:adequate:Cav:adequate:Exon 3:ExonStart false|LIMI

GCC AAC ATG CTG :Koz:adequate:Cav:strong:Exon 5:ExonStart false|ANML

ATC ACC ATG GAT :Koz:strong:Cav:strong:Exon 5:ExonStart false|ITMD

GCC TCT ATG GTA :Koz:adequate:Cav:adequate:Exon 7:ExonStart false|ASMV

GAT GAC ATG GCC :Koz:strong:Cav:weak:Exon 7:ExonStart false|DDMA

GGA CAC ATG CGG :Koz:weak:Cav:weak:Exon 7:ExonStart false|GHMR

GTC TCG ATG ATG :Koz:weak:Cav:adequate:Exon 8:ExonStart false|VSMM

TCG ATG ATG TTC :Koz:adequate:Cav:adequate:Exon 8:ExonStart false|SMMF

CG6846-PA

AGC AAG ATG AAA :Koz:adequate:Cav:strong:Exon 2:ExonStart false|SKMK*****

CGC CTC ATG TCC :Koz:weak:Cav:adequate:Exon 2:ExonStart false|RLMS***NonAcetSite**

CGT TCC ATG CCC :Koz:weak:Cav:weak:Exon 2:ExonStart false|RSMP

CAG CCC ATG GAG :Koz:adequate:Cav:weak:Exon 2:ExonStart false|QPME

CG6950-PA

GGC AAA ATG CTA :Koz:adequate:Cav:strong:Exon 2:ExonStart false|GKML

ATT AAA ATG GAG :Koz:strong:Cav:adequate:Exon 3:ExonStart false|IKME***AcetSite**

CTG GCC ATG CAA :Koz:adequate:Cav:weak:Exon 3:ExonStart false|LAMQ

ACC ATC ATG GGC :Koz:strong:Cav:strong:Exon 3:ExonStart false|TIMG

GAG CCT ATG GTC :Koz:adequate:Cav:weak:Exon 3:ExonStart false|EPMV

GTC AAG ATG GCC :Koz:strong:Cav:strong:Exon 3:ExonStart false|VKMA

ACA AAG ATG ATT :Koz:adequate:Cav:adequate:Exon 4:ExonStart false|TKMI

CCG GGC ATG TGG :Koz:adequate:Cav:weak:Exon 4:ExonStart false|PGMW

CTG CAG ATG GTG :Koz:adequate:Cav:weak:Exon 4:ExonStart false|LQMV

GAC TTC ATG GCC :Koz:adequate:Cav:adequate:Exon 4:ExonStart false|DFMA

TCC GGT ATG CGT :Koz:adequate:Cav:adequate:Exon 4:ExonStart false|SGMR

TAC TTT ATG CTG :Koz:weak:Cav:adequate:Exon 4:ExonStart false|TFML

AAG TGG ATG ACC :Koz:weak:Cav:weak:Exon 4:ExonStart false|KWMT

AAG AAC ATG GGC :Koz:strong:Cav:adequate:Exon 4:ExonStart false|KNMG

CG6950-PB

TTA CAG ATG CTA :Koz:weak:Cav:weak:Exon 1:ExonStart false|LQML

ATT AAA ATG GAG :Koz:strong:Cav:adequate:Exon 1:ExonStart false|IKME***AcetSite**

CTG GCC ATG CAA :Koz:adequate:Cav:weak:Exon 1:ExonStart false|LAMQ

ACC ATC ATG GGC :Koz:strong:Cav:strong:Exon 1:ExonStart false|TIMG

GAG CCT ATG GTC :Koz:adequate:Cav:weak:Exon 1:ExonStart false|EPMV

GTC AAG ATG GCC :Koz:strong:Cav:strong:Exon 1:ExonStart false|VKMA

ACA AAG ATG ATT :Koz:adequate:Cav:adequate:Exon 2:ExonStart false|TKMI

CCG GGC ATG TGG :Koz:adequate:Cav:weak:Exon 2:ExonStart false|PGMW

CTG CAG ATG GTG :Koz:adequate:Cav:weak:Exon 2:ExonStart false|LQMV

GAC TTC ATG GCC :Koz:adequate:Cav:adequate:Exon 2:ExonStart false|DFMA

TCC GGT ATG CGT :Koz:adequate:Cav:adequate:Exon 2:ExonStart false|SGMR

TAC TTT ATG CTG :Koz:weak:Cav:adequate:Exon 2:ExonStart false|TFML

AAG TGG ATG ACC :Koz:weak:Cav:weak:Exon 2:ExonStart false|KWMT

AAG AAC ATG GGC :Koz:strong:Cav:adequate:Exon 2:ExonStart false|KNMG

CG6950-PC

TAA TTT ATG CTA :Koz:weak:Cav:weak:Exon 1:ExonStart false|stopFML

ATT AAA ATG GAG :Koz:strong:Cav:adequate:Exon 2:ExonStart false|IKME***AcetSite**

CTG GCC ATG CAA :Koz:adequate:Cav:weak:Exon 2:ExonStart false|LAMQ

ACC ATC ATG GGC :Koz:strong:Cav:strong:Exon 2:ExonStart false|TIMG

GAG CCT ATG GTC :Koz:adequate:Cav:weak:Exon 2:ExonStart false|EPMV

GTC AAG ATG GCC :Koz:strong:Cav:strong:Exon 2:ExonStart false|VKMA

ACA AAG ATG ATT :Koz:adequate:Cav:adequate:Exon 3:ExonStart false|TKMI

CCG GGC ATG TGG :Koz:adequate:Cav:weak:Exon 3:ExonStart false|PGMW

CTG CAG ATG GTG :Koz:adequate:Cav:weak:Exon 3:ExonStart false|LQMV

GAC TTC ATG GCC :Koz:adequate:Cav:adequate:Exon 3:ExonStart false|DFMA

TCC GGT ATG CGT :Koz:adequate:Cav:adequate:Exon 3:ExonStart false|SGMR

TAC TTT ATG CTG :Koz:weak:Cav:adequate:Exon 3:ExonStart false|TFML

AAG TGG ATG ACC :Koz:weak:Cav:weak:Exon 3:ExonStart false|KWMT

AAG AAC ATG GGC :Koz:strong:Cav:adequate:Exon 3:ExonStart false|KNMG

CG6950-PD

TTT TGG ATG CTA :Koz:weak:Cav:weak:Exon 1:ExonStart false|FWML

ATT AAA ATG GAG :Koz:strong:Cav:adequate:Exon 2:ExonStart false|IKME***AcetSite**

CTG GCC ATG CAA :Koz:adequate:Cav:weak:Exon 2:ExonStart false|LAMQ

ACC ATC ATG GGC :Koz:strong:Cav:strong:Exon 2:ExonStart false|TIMG

GAG CCT ATG GTC :Koz:adequate:Cav:weak:Exon 2:ExonStart false|EPMV

GTC AAG ATG GCC :Koz:strong:Cav:strong:Exon 2:ExonStart false|VKMA

ACA AAG ATG ATT :Koz:adequate:Cav:adequate:Exon 3:ExonStart false|TKMI

CCG GGC ATG TGG :Koz:adequate:Cav:weak:Exon 3:ExonStart false|PGMW

CTG CAG ATG GTG :Koz:adequate:Cav:weak:Exon 3:ExonStart false|LQMV

GAC TTC ATG GCC :Koz:adequate:Cav:adequate:Exon 3:ExonStart false|DFMA

TCC GGT ATG CGT :Koz:adequate:Cav:adequate:Exon 3:ExonStart false|SGMR

TAC TTT ATG CTG :Koz:weak:Cav:adequate:Exon 3:ExonStart false|TFML

AAG TGG ATG ACC :Koz:weak:Cav:weak:Exon 3:ExonStart false|KWMT

AAG AAC ATG GGC :Koz:strong:Cav:adequate:Exon 3:ExonStart false|KNMG

CG7224-PA

AAC AAA ATG CAA :Koz:adequate:Cav:strong:Exon 1:ExonStart false|NKMQ

CCC CAA ATG GGC :Koz:adequate:Cav:adequate:Exon 1:ExonStart false|PQMG

GGC GAC ATG GTG :Koz:strong:Cav:adequate:Exon 2:ExonStart false|GDMV***NonAcetSite**

AAG CTA ATG GCC :Koz:adequate:Cav:weak:Exon 2:ExonStart false|KLMA

CG7319-PC

... ... ATG GCT :Koz:adequate:Cav:weak:Exon 1:ExonStart false|nullnullMA

TTT ATA ATG TCG :Koz:adequate:Cav:adequate:Exon 2:ExonStart false|FIMS

CAG TTC ATG AAG :Koz:weak:Cav:weak:Exon 2:ExonStart false|QFMK***NonAcetSite**

AAG AAG ATG GCC :Koz:strong:Cav:adequate:Exon 3:ExonStart false|KKMA

AGC GGC ATG CGC :Koz:adequate:Cav:adequate:Exon 3:ExonStart false|SGMR

CCT CCA ATG CCC :Koz:weak:Cav:weak:Exon 3:ExonStart false|PPMP

TTC CTC ATG GAC :Koz:adequate:Cav:adequate:Exon 3:ExonStart false|FLMD

ACC TGT ATG ACT :Koz:weak:Cav:adequate:Exon 3:ExonStart false|TCMT

TCG GTG ATG TCG :Koz:adequate:Cav:weak:Exon 3:ExonStart false|SVMS

CCC GTC ATG AAG :Koz:adequate:Cav:adequate:Exon 3:ExonStart false|PVMK

TTT AGC ATG CGC :Koz:adequate:Cav:adequate:Exon 3:ExonStart false|FSMR

CG7393-PA

AAC GAA ATG TCG :Koz:adequate:Cav:adequate:Exon 2:ExonStart false|NEMS

CGC AAA ATG GCC :Koz:strong:Cav:strong:Exon 2:ExonStart false|RKMA***AcetSite**

AAG CAC ATG GAT :Koz:adequate:Cav:weak:Exon 2:ExonStart false|KHMD

GTG TAC ATG GTG :Koz:adequate:Cav:weak:Exon 2:ExonStart false|VTMV

CAC TTG ATG GAC :Koz:adequate:Cav:adequate:Exon 2:ExonStart false|HLMD

AGC GAG ATG ACC :Koz:adequate:Cav:adequate:Exon 2:ExonStart false|SEMT

GAG ATC ATG CTC :Koz:adequate:Cav:adequate:Exon 2:ExonStart false|EIML

AAC TGG ATG CAC :Koz:weak:Cav:adequate:Exon 2:ExonStart false|NWMH

TGC ATC ATG GCT :Koz:strong:Cav:strong:Exon 2:ExonStart false|CIMA

CTC ATC ATG GAA :Koz:strong:Cav:strong:Exon 2:ExonStart false|LIME

GAG TTC ATG AGC :Koz:weak:Cav:weak:Exon 2:ExonStart false|EFMS

CCG GTG ATG CCG :Koz:adequate:Cav:weak:Exon 2:ExonStart false|PVMP

GAG AAG ATG CTG :Koz:adequate:Cav:adequate:Exon 2:ExonStart false|EKML

CCG TAC ATG GAG :Koz:adequate:Cav:weak:Exon 2:ExonStart false|PTME

CGG GAG ATG GTC :Koz:strong:Cav:weak:Exon 2:ExonStart false|REMV

CG7478-PA

CCA AAC ATG TGT :Koz:adequate:Cav:adequate:Exon 2:ExonStart false|PNMC

TCC GGC ATG TGC :Koz:adequate:Cav:adequate:Exon 2:ExonStart false|SGMC***NonAcetSite**

GGC GTG ATG GTG :Koz:strong:Cav:adequate:Exon 2:ExonStart false|GVMV

GTG GGT ATG GGT :Koz:strong:Cav:weak:Exon 2:ExonStart false|VGMG

GAT GAC ATG GAG :Koz:strong:Cav:weak:Exon 2:ExonStart false|DDME

GAG AAG ATG ACC :Koz:adequate:Cav:adequate:Exon 2:ExonStart false|EKMT

CAG ATC ATG TTC :Koz:adequate:Cav:adequate:Exon 2:ExonStart false|QIMF

CCG GCC ATG TAC :Koz:adequate:Cav:weak:Exon 2:ExonStart false|PAMT

TAC CTG ATG AAG :Koz:weak:Cav:adequate:Exon 2:ExonStart false|TLMK

CAG GAG ATG GCC :Koz:strong:Cav:weak:Exon 2:ExonStart false|QEMA

CTG GGC ATG GAG :Koz:strong:Cav:weak:Exon 2:ExonStart false|LGME

TCC ATC ATG AAG :Koz:adequate:Cav:strong:Exon 2:ExonStart false|SIMK

ACT ACC ATG TAT :Koz:adequate:Cav:adequate:Exon 2:ExonStart false|TTMT

GAC CGT ATG CAA :Koz:weak:Cav:adequate:Exon 3:ExonStart false|DRMQ

CAG CAG ATG TGG :Koz:weak:Cav:weak:Exon 3:ExonStart false|QQMW

CG7610-PA

GCA AGG ATG ATG :Koz:adequate:Cav:adequate:Exon 2:ExonStart true|ARMM

AGG ATG ATG ATG :Koz:adequate:Cav:adequate:Exon 2:ExonStart false|RMMM

ATG ATG ATG CAA :Koz:adequate:Cav:adequate:Exon 2:ExonStart false|MMMQ

CTG GCC ATG GAG :Koz:strong:Cav:weak:Exon 2:ExonStart false|LAME

GCC ACC ATG CTG :Koz:adequate:Cav:strong:Exon 2:ExonStart false|ATML

CGT GGC ATG GCC :Koz:strong:Cav:weak:Exon 2:ExonStart false|RGMA***NonAcetSite**

CTG AAG ATG ATT :Koz:adequate:Cav:adequate:Exon 2:ExonStart false|LKMI

CAA TCG ATG AAG :Koz:weak:Cav:weak:Exon 2:ExonStart false|QSMK

ATG AAG ATG GTG :Koz:strong:Cav:adequate:Exon 2:ExonStart false|MKMV

ATC CTG ATG GTG :Koz:adequate:Cav:adequate:Exon 2:ExonStart false|ILMV

TAC ACC ATG AAG :Koz:adequate:Cav:strong:Exon 2:ExonStart false|TTMK

TCC CGT ATG ACT :Koz:weak:Cav:adequate:Exon 2:ExonStart false|SRMT

ACT GCC ATG GAC :Koz:strong:Cav:weak:Exon 2:ExonStart false|TAMD

GGT GAG ATG ATC :Koz:adequate:Cav:weak:Exon 2:ExonStart false|GEMI

CG7610-PB

CAC AGG ATG ATG :Koz:adequate:Cav:strong:Exon 2:ExonStart false|HRMM

AGG ATG ATG ATG :Koz:adequate:Cav:adequate:Exon 2:ExonStart false|RMMM

ATG ATG ATG CAA :Koz:adequate:Cav:adequate:Exon 2:ExonStart false|MMMQ

CTG GCC ATG GAG :Koz:strong:Cav:weak:Exon 2:ExonStart false|LAME

GCC ACC ATG CTG :Koz:adequate:Cav:strong:Exon 2:ExonStart false|ATML

CGT GGC ATG GCC :Koz:strong:Cav:weak:Exon 2:ExonStart false|RGMA***NonAcetSite**

CTG AAG ATG ATT :Koz:adequate:Cav:adequate:Exon 2:ExonStart false|LKMI

CAA TCG ATG AAG :Koz:weak:Cav:weak:Exon 2:ExonStart false|QSMK

ATG AAG ATG GTG :Koz:strong:Cav:adequate:Exon 2:ExonStart false|MKMV

ATC CTG ATG GTG :Koz:adequate:Cav:adequate:Exon 2:ExonStart false|ILMV

TAC ACC ATG AAG :Koz:adequate:Cav:strong:Exon 2:ExonStart false|TTMK

TCC CGT ATG ACT :Koz:weak:Cav:adequate:Exon 2:ExonStart false|SRMT

ACT GCC ATG GAC :Koz:strong:Cav:weak:Exon 2:ExonStart false|TAMD

GGT GAG ATG ATC :Koz:adequate:Cav:weak:Exon 2:ExonStart false|GEMI

CG7610-PC

CAC AGG ATG ATG :Koz:adequate:Cav:strong:Exon 1:ExonStart false|HRMM

AGG ATG ATG ATG :Koz:adequate:Cav:adequate:Exon 1:ExonStart false|RMMM

ATG ATG ATG CAA :Koz:adequate:Cav:adequate:Exon 1:ExonStart false|MMMQ

CTG GCC ATG GAG :Koz:strong:Cav:weak:Exon 1:ExonStart false|LAME

GCC ACC ATG CTG :Koz:adequate:Cav:strong:Exon 1:ExonStart false|ATML

CGT GGC ATG GCC :Koz:strong:Cav:weak:Exon 1:ExonStart false|RGMA***NonAcetSite**

CTG AAG ATG ATT :Koz:adequate:Cav:adequate:Exon 1:ExonStart false|LKMI

CAA TCG ATG AAG :Koz:weak:Cav:weak:Exon 1:ExonStart false|QSMK

ATG AAG ATG GTG :Koz:strong:Cav:adequate:Exon 1:ExonStart false|MKMV

ATC CTG ATG GTG :Koz:adequate:Cav:adequate:Exon 1:ExonStart false|ILMV

TAC ACC ATG AAG :Koz:adequate:Cav:strong:Exon 1:ExonStart false|TTMK

TCC CGT ATG ACT :Koz:weak:Cav:adequate:Exon 1:ExonStart false|SRMT

ACT GCC ATG GAC :Koz:strong:Cav:weak:Exon 1:ExonStart false|TAMD

GGT GAG ATG ATC :Koz:adequate:Cav:weak:Exon 1:ExonStart false|GEMI

CG7635-PA

ACA GCA ATG GAG :Koz:strong:Cav:weak:Exon 1:ExonStart false|TAME*****

GAA GAT ATG CGC :Koz:adequate:Cav:weak:Exon 1:ExonStart false|EDMR

GCG TAC ATG GTC :Koz:adequate:Cav:weak:Exon 1:ExonStart false|ATMV***NonAcetSite**

GTC AAC ATG GGC :Koz:strong:Cav:strong:Exon 1:ExonStart false|VNMG***NonAcetSite**

GCC GGC ATG GCA :Koz:strong:Cav:adequate:Exon 1:ExonStart false|AGMA

GGC TGC ATG GAA :Koz:adequate:Cav:adequate:Exon 2:ExonStart false|GCME

CCC GGC ATG TTC :Koz:adequate:Cav:adequate:Exon 2:ExonStart false|PGMF

CAG GAG ATG CTG :Koz:adequate:Cav:weak:Exon 2:ExonStart false|QEML

TAC AGC ATG TCC :Koz:adequate:Cav:strong:Exon 2:ExonStart false|TSMS

CAC AAT ATG CAG :Koz:adequate:Cav:strong:Exon 2:ExonStart false|HNMQ

GGC GTC ATG GTG :Koz:strong:Cav:adequate:Exon 2:ExonStart false|GVMV

GTT TCC ATG CAG :Koz:weak:Cav:weak:Exon 3:ExonStart false|VSMQ

CGT GCC ATG GCC :Koz:strong:Cav:weak:Exon 3:ExonStart false|RAMA

CTG CCC ATG GAG :Koz:adequate:Cav:weak:Exon 4:ExonStart false|LPME

CAC TTG ATG GGC :Koz:adequate:Cav:adequate:Exon 4:ExonStart false|HLMG

CG7741-PA

CAA TTA ATG CAC :Koz:weak:Cav:weak:Exon 3:ExonStart false|QLMH

ACC AAC ATG ATG :Koz:adequate:Cav:strong:Exon 3:ExonStart false|TNMM

AAC ATG ATG AAG :Koz:adequate:Cav:strong:Exon 3:ExonStart false|NMMK

ATA ACC ATG GAC :Koz:strong:Cav:adequate:Exon 3:ExonStart false|ITMD***AcetSite**

TTT GGT ATG CAG :Koz:adequate:Cav:weak:Exon 5:ExonStart false|FGMQ

TTT CGG ATG CCC :Koz:weak:Cav:weak:Exon 6:ExonStart false|FRMP

TAC GAC ATG GAT :Koz:strong:Cav:adequate:Exon 7:ExonStart false|TDMD

CAT GTC ATG ATA :Koz:adequate:Cav:weak:Exon 8:ExonStart false|HVMI

GAG AAA ATG CTG :Koz:adequate:Cav:adequate:Exon 9:ExonStart false|EKML

CCT GAA ATG GGA :Koz:strong:Cav:weak:Exon 9:ExonStart false|PEMG

CGG CAA ATG AAA :Koz:weak:Cav:weak:Exon 9:ExonStart false|RQMK

CG7867-PD

AGG CAA ATG TAT :Koz:weak:Cav:weak:Exon 2:ExonStart false|RQMT

TAT CCC ATG GCT :Koz:adequate:Cav:weak:Exon 2:ExonStart false|TPMA***AcetSite**

CGG CAC ATG AAA :Koz:weak:Cav:weak:Exon 2:ExonStart false|RHMK

TAT CAC ATG CTC :Koz:weak:Cav:weak:Exon 3:ExonStart false|THML

TGC CAG ATG AAG :Koz:weak:Cav:adequate:Exon 4:ExonStart false|CQMK

GAG CTG ATG CTG :Koz:weak:Cav:weak:Exon 4:ExonStart false|ELML

CGG GCA ATG CCG :Koz:adequate:Cav:weak:Exon 4:ExonStart false|RAMP

GAG GAG ATG CGC :Koz:adequate:Cav:weak:Exon 4:ExonStart false|EEMR

GCC ACG ATG CTG :Koz:adequate:Cav:strong:Exon 5:ExonStart false|ATML

GCC ACC ATG CTG :Koz:adequate:Cav:strong:Exon 5:ExonStart false|ATML

GAG GAG ATG TCA :Koz:adequate:Cav:weak:Exon 5:ExonStart false|EEMS

AAG CCC ATG GAG :Koz:adequate:Cav:weak:Exon 6:ExonStart false|KPME

CG8127-PB

AAA TGC ATG GAG :Koz:adequate:Cav:weak:Exon 1:ExonStart false|KCME

TTT GAG ATG CTG :Koz:adequate:Cav:weak:Exon 1:ExonStart false|FEML***AcetSite**

GGT GGG ATG AAG :Koz:adequate:Cav:weak:Exon 1:ExonStart false|GGMK

GCA TAT ATG GAT :Koz:adequate:Cav:weak:Exon 1:ExonStart false|ATMD

AGC AAT ATG CAC :Koz:adequate:Cav:strong:Exon 1:ExonStart false|SNMH

TCG GTG ATG CGG :Koz:adequate:Cav:weak:Exon 1:ExonStart false|SVMR

GTG GGC ATG AGT :Koz:adequate:Cav:weak:Exon 3:ExonStart false|VGMS

GCG GCC ATG CAA :Koz:adequate:Cav:weak:Exon 4:ExonStart false|AAMQ

TCG GCG ATG CGG :Koz:adequate:Cav:weak:Exon 4:ExonStart false|SAMR

TAC TCC ATG CCC :Koz:weak:Cav:adequate:Exon 4:ExonStart false|TSMP

GCC GGC ATG ATT :Koz:adequate:Cav:adequate:Exon 5:ExonStart false|AGMI

ATC TGC ATG TTT :Koz:weak:Cav:adequate:Exon 5:ExonStart false|ICMF

CAG GTG ATG CGA :Koz:adequate:Cav:weak:Exon 5:ExonStart false|QVMR

GAG CGC ATG AAC :Koz:weak:Cav:weak:Exon 5:ExonStart false|ERMN

AAC TCG ATG AAC :Koz:weak:Cav:adequate:Exon 5:ExonStart false|NSMN

GAG AAG ATG TAC :Koz:adequate:Cav:adequate:Exon 5:ExonStart false|EKMT

GAG ACG ATG CCC :Koz:adequate:Cav:adequate:Exon 5:ExonStart false|ETMP

CAG CAG ATG TGG :Koz:weak:Cav:weak:Exon 5:ExonStart false|QQMW

TGG TCC ATG GAG :Koz:adequate:Cav:weak:Exon 5:ExonStart false|WSME

GAT GCC ATG GAC :Koz:strong:Cav:weak:Exon 5:ExonStart false|DAMD

GCT GAG ATG GAT :Koz:strong:Cav:weak:Exon 5:ExonStart false|AEMD

CAC GCG ATG TGC :Koz:adequate:Cav:adequate:Exon 6:ExonStart false|HAMC

GAG GAT ATG CCC :Koz:adequate:Cav:weak:Exon 6:ExonStart false|EDMP

TCG CTG ATG GAC :Koz:adequate:Cav:weak:Exon 6:ExonStart false|SLMD

CTG CAC ATG CAC :Koz:weak:Cav:weak:Exon 6:ExonStart false|LHMH

GCC TCG ATG GCC :Koz:adequate:Cav:adequate:Exon 6:ExonStart false|ASMA

TCT CTC ATG GCC :Koz:adequate:Cav:weak:Exon 6:ExonStart false|SLMA

CCG CGC ATG ACG :Koz:weak:Cav:weak:Exon 6:ExonStart false|PRMT

GAG CAG ATG AAG :Koz:weak:Cav:weak:Exon 6:ExonStart false|EQMK

AAG GTA ATG CTG :Koz:adequate:Cav:weak:Exon 6:ExonStart false|KVML

CG8235-PA

... ..A ATG CTG :Koz:weak:Cav:weak:Exon 1:ExonStart false|nullnullML

AGA ACA ATG GCC :Koz:strong:Cav:adequate:Exon 1:ExonStart false|RTMA***AcetSite**

GTA GTT ATG CCG :Koz:adequate:Cav:weak:Exon 2:ExonStart false|VVMP

GAA GAG ATG CAG :Koz:adequate:Cav:weak:Exon 2:ExonStart false|EEMQ

GTG GTC ATG TGT :Koz:adequate:Cav:weak:Exon 2:ExonStart false|VVMC

GCC AAA ATG CGC :Koz:adequate:Cav:strong:Exon 2:ExonStart false|AKMR

GAG GCT ATG GTC :Koz:strong:Cav:weak:Exon 2:ExonStart false|EAMV

ATG GTC ATG TGC :Koz:adequate:Cav:weak:Exon 2:ExonStart false|MVMC

CG8266-PA

TTG TGT ATG AAG :Koz:weak:Cav:weak:Exon 1:ExonStart false|LCMK

ACA AGC ATG AGT :Koz:adequate:Cav:adequate:Exon 3:ExonStart false|TSMS

CTG GGA ATG TCG :Koz:adequate:Cav:weak:Exon 3:ExonStart false|LGMS

GAT CTA ATG GTT :Koz:adequate:Cav:weak:Exon 3:ExonStart false|DLMV

CCG GGC ATG GAT :Koz:strong:Cav:weak:Exon 3:ExonStart false|PGMD

AAA TGG ATG AAG :Koz:weak:Cav:weak:Exon 3:ExonStart false|KWMK

GAG GAA ATG CTG :Koz:adequate:Cav:weak:Exon 4:ExonStart false|EEML

AAC GCC ATG CTC :Koz:adequate:Cav:adequate:Exon 7:ExonStart false|NAML

GCT GAG ATG GTG :Koz:strong:Cav:weak:Exon 7:ExonStart false|AEMV

ATG GTG ATG CTG :Koz:adequate:Cav:weak:Exon 7:ExonStart false|MVML

ATG CTG ATG TGC :Koz:weak:Cav:weak:Exon 7:ExonStart false|MLMC***NonAcetSite**

GCT CCC ATG CCC :Koz:weak:Cav:weak:Exon 7:ExonStart false|APMP

TAC GGA ATG CCA :Koz:adequate:Cav:adequate:Exon 7:ExonStart false|TGMP

CCA CTC ATG CCA :Koz:weak:Cav:weak:Exon 7:ExonStart false|PLMP

GAG ACA ATG CCA :Koz:adequate:Cav:adequate:Exon 7:ExonStart false|ETMP

AAT GCT ATG GAA :Koz:strong:Cav:weak:Exon 11:ExonStart false|NAME

GGC TTT ATG CAG :Koz:weak:Cav:adequate:Exon 11:ExonStart false|GFMQ

CG8266-PB

TTG TGT ATG AAG :Koz:weak:Cav:weak:Exon 1:ExonStart false|LCMK

ACA AGC ATG AGT :Koz:adequate:Cav:adequate:Exon 3:ExonStart false|TSMS

CTG GGA ATG TCG :Koz:adequate:Cav:weak:Exon 3:ExonStart false|LGMS

GAT CTA ATG GTT :Koz:adequate:Cav:weak:Exon 3:ExonStart false|DLMV

CCG GGC ATG GAT :Koz:strong:Cav:weak:Exon 3:ExonStart false|PGMD

AAA TGG ATG AAG :Koz:weak:Cav:weak:Exon 3:ExonStart false|KWMK

GAG GAA ATG CTG :Koz:adequate:Cav:weak:Exon 4:ExonStart false|EEML

AAC GCC ATG CTC :Koz:adequate:Cav:adequate:Exon 7:ExonStart false|NAML

GCT GAG ATG GTG :Koz:strong:Cav:weak:Exon 7:ExonStart false|AEMV

ATG GTG ATG CTG :Koz:adequate:Cav:weak:Exon 7:ExonStart false|MVML

ATG CTG ATG TGC :Koz:weak:Cav:weak:Exon 7:ExonStart false|MLMC***NonAcetSite**

GCT CCC ATG CCC :Koz:weak:Cav:weak:Exon 7:ExonStart false|APMP

TAC GGA ATG CCA :Koz:adequate:Cav:adequate:Exon 7:ExonStart false|TGMP

CCA CTC ATG CCA :Koz:weak:Cav:weak:Exon 7:ExonStart false|PLMP

GAG ACA ATG CCA :Koz:adequate:Cav:adequate:Exon 7:ExonStart false|ETMP

AAT GCT ATG GAA :Koz:strong:Cav:weak:Exon 11:ExonStart false|NAME

GGC TTT ATG CAG :Koz:weak:Cav:adequate:Exon 11:ExonStart false|GFMQ

CG8472-PA

ACA AAA ATG GCC :Koz:strong:Cav:adequate:Exon 2:ExonStart false|TKMA*****

ACA GTT ATG CGC :Koz:adequate:Cav:weak:Exon 3:ExonStart false|TVMR

CAG GAC ATG ATC :Koz:adequate:Cav:weak:Exon 3:ExonStart false|QDMI

CTT ACC ATG ATG :Koz:adequate:Cav:adequate:Exon 4:ExonStart false|LTMM

ACC ATG ATG GCA :Koz:strong:Cav:strong:Exon 4:ExonStart false|TMMA

CGC AAA ATG AAG :Koz:adequate:Cav:strong:Exon 4:ExonStart false|RKMK***NonAcetSite**

CAC GTG ATG ACA :Koz:adequate:Cav:adequate:Exon 4:ExonStart false|HVMT

GAT GAG ATG ATC :Koz:adequate:Cav:weak:Exon 4:ExonStart false|DEMI

GTG ACT ATG ATG :Koz:adequate:Cav:adequate:Exon 5:ExonStart false|VTMM

ACT ATG ATG ACA :Koz:adequate:Cav:adequate:Exon 5:ExonStart false|TMMT

CG8472-PB

ATC ATA ATG GCC :Koz:strong:Cav:strong:Exon 1:ExonStart false|IIMA*****

ACA GTT ATG CGC :Koz:adequate:Cav:weak:Exon 2:ExonStart false|TVMR

CAG GAC ATG ATC :Koz:adequate:Cav:weak:Exon 2:ExonStart false|QDMI

CTT ACC ATG ATG :Koz:adequate:Cav:adequate:Exon 3:ExonStart false|LTMM

ACC ATG ATG GCA :Koz:strong:Cav:strong:Exon 3:ExonStart false|TMMA

CGC AAA ATG AAG :Koz:adequate:Cav:strong:Exon 3:ExonStart false|RKMK***NonAcetSite**

CAC GTG ATG ACA :Koz:adequate:Cav:adequate:Exon 3:ExonStart false|HVMT

GAT GAG ATG ATC :Koz:adequate:Cav:weak:Exon 3:ExonStart false|DEMI

GTG ACT ATG ATG :Koz:adequate:Cav:adequate:Exon 4:ExonStart false|VTMM

ACT ATG ATG ACA :Koz:adequate:Cav:adequate:Exon 4:ExonStart false|TMMT

CG8520-PA

TCT GCT ATG TTC :Koz:adequate:Cav:weak:Exon 2:ExonStart false|SAMF

TGC CAC ATG AGC :Koz:weak:Cav:adequate:Exon 2:ExonStart false|CHMS***NonAcetSite**

CAA CTG ATG ACC :Koz:weak:Cav:weak:Exon 2:ExonStart false|QLMT

CAG ACG ATG AAG :Koz:adequate:Cav:adequate:Exon 2:ExonStart false|QTMK

ACT CTG ATG GAC :Koz:adequate:Cav:weak:Exon 2:ExonStart false|TLMD

TCG TTC ATG AAC :Koz:weak:Cav:weak:Exon 3:ExonStart false|SFMN

GAC GCC ATG GTG :Koz:strong:Cav:adequate:Exon 3:ExonStart false|DAMV

GGC AGT ATG AAT :Koz:adequate:Cav:strong:Exon 3:ExonStart false|GSMN

AAT CGC ATG TTC :Koz:weak:Cav:weak:Exon 3:ExonStart false|NRMF

GCA CAG ATG CGA :Koz:weak:Cav:weak:Exon 4:ExonStart false|AQMR

ACC CTA ATG GAC :Koz:adequate:Cav:adequate:Exon 4:ExonStart false|TLMD

TAT GAA ATG CAG :Koz:adequate:Cav:weak:Exon 5:ExonStart false|TEMQ

CG8604-PA

CGC TGA ATG ACC :Koz:weak:Cav:adequate:Exon 1:ExonStart false|RstopMT

GGC ATA ATG CTG :Koz:adequate:Cav:strong:Exon 1:ExonStart false|GIML***AcetSite**

ACT CTG ATG GAT :Koz:adequate:Cav:weak:Exon 3:ExonStart false|TLMD

CCC GAA ATG AAG :Koz:adequate:Cav:adequate:Exon 3:ExonStart false|PEMK

TGG CTG ATG GGT :Koz:adequate:Cav:weak:Exon 10:ExonStart false|WLMG

CG8728-PA

CAA CAC ATG ATG :Koz:weak:Cav:weak:Exon 1:ExonStart false|QHMM

CAC ATG ATG AAC :Koz:adequate:Cav:strong:Exon 1:ExonStart false|HMMN

ATC GGC ATG CTG :Koz:adequate:Cav:adequate:Exon 1:ExonStart false|IGML

CGC CTT ATG GCC :Koz:adequate:Cav:adequate:Exon 2:ExonStart false|RLMA***NonAcetSite**

CTG GGC ATG CGG :Koz:adequate:Cav:weak:Exon 3:ExonStart false|LGMR

ATA CTG ATG GAC :Koz:adequate:Cav:weak:Exon 3:ExonStart false|ILMD

ATG GAC ATG ATC :Koz:adequate:Cav:weak:Exon 3:ExonStart false|MDMI

GTG CTA ATG AAC :Koz:weak:Cav:weak:Exon 3:ExonStart false|VLMN

AAG CGC ATG GTC :Koz:adequate:Cav:weak:Exon 3:ExonStart false|KRMV

AAT ATC ATG ATG :Koz:adequate:Cav:adequate:Exon 5:ExonStart false|NIMM

ATC ATG ATG GGC :Koz:strong:Cav:strong:Exon 5:ExonStart false|IMMG

AAG GGC ATG TAC :Koz:adequate:Cav:weak:Exon 5:ExonStart false|KGMT

CAC TGG ATG TAC :Koz:weak:Cav:adequate:Exon 5:ExonStart false|HWMT

CAG CAC ATG AAC :Koz:weak:Cav:weak:Exon 5:ExonStart false|QHMN

AAC GAC ATG GTC :Koz:strong:Cav:adequate:Exon 5:ExonStart false|NDMV

CGC GAG ATG ATG :Koz:adequate:Cav:adequate:Exon 5:ExonStart false|REMM

GAG ATG ATG GGC :Koz:strong:Cav:adequate:Exon 5:ExonStart false|EMMG

ATG GGC ATG GCC :Koz:strong:Cav:weak:Exon 5:ExonStart false|MGMA

GAG CTA ATG CGC :Koz:weak:Cav:weak:Exon 5:ExonStart false|ELMR

CAG TCC ATG TTA :Koz:weak:Cav:weak:Exon 5:ExonStart false|QSML

TTA CTA ATG AAC :Koz:weak:Cav:weak:Exon 5:ExonStart false|LLMN

CCC GAG ATG AGC :Koz:adequate:Cav:adequate:Exon 6:ExonStart false|PEMS

CG8863-PA

AAC AAA ATG GTT :Koz:strong:Cav:strong:Exon 2:ExonStart false|NKMV*****

AAT CCT ATG GAC :Koz:adequate:Cav:weak:Exon 2:ExonStart false|NPMD

CAC CAG ATG TCC :Koz:weak:Cav:adequate:Exon 2:ExonStart false|HQMS***NonAcetSite**

GGC ATC ATG CAG :Koz:adequate:Cav:strong:Exon 2:ExonStart false|GIMQ

AAG GGA ATG CGG :Koz:adequate:Cav:weak:Exon 2:ExonStart false|KGMR

GAT TTA ATG ATG :Koz:weak:Cav:weak:Exon 2:ExonStart false|DLMM

TTA ATG ATG AAG :Koz:adequate:Cav:adequate:Exon 2:ExonStart false|LMMK

ATG AAG ATG CCG :Koz:adequate:Cav:adequate:Exon 2:ExonStart false|MKMP

CAT GAG ATG ACC :Koz:adequate:Cav:weak:Exon 2:ExonStart false|HEMT

GAG GGT ATG CCC :Koz:adequate:Cav:weak:Exon 2:ExonStart false|EGMP

AAC CCC ATG GAG :Koz:adequate:Cav:adequate:Exon 2:ExonStart false|NPME

CAG CGC ATG GCC :Koz:adequate:Cav:weak:Exon 3:ExonStart false|QRMA

CG8863-PB

AAC AAA ATG GTT :Koz:strong:Cav:strong:Exon 2:ExonStart false|NKMV*****

AAT CCT ATG GAC :Koz:adequate:Cav:weak:Exon 2:ExonStart false|NPMD

CAC CAG ATG TCC :Koz:weak:Cav:adequate:Exon 2:ExonStart false|HQMS***NonAcetSite**

GGC ATC ATG CAG :Koz:adequate:Cav:strong:Exon 2:ExonStart false|GIMQ

AAG GGA ATG CGG :Koz:adequate:Cav:weak:Exon 2:ExonStart false|KGMR

GAT TTA ATG ATG :Koz:weak:Cav:weak:Exon 2:ExonStart false|DLMM

TTA ATG ATG AAG :Koz:adequate:Cav:adequate:Exon 2:ExonStart false|LMMK

ATG AAG ATG CCG :Koz:adequate:Cav:adequate:Exon 2:ExonStart false|MKMP

CAT GAG ATG ACC :Koz:adequate:Cav:weak:Exon 2:ExonStart false|HEMT

GAG GGT ATG CCC :Koz:adequate:Cav:weak:Exon 2:ExonStart false|EGMP

AAC CCC ATG GAG :Koz:adequate:Cav:adequate:Exon 2:ExonStart false|NPME

CAG CGC ATG GCC :Koz:adequate:Cav:weak:Exon 3:ExonStart false|QRMA

CG8863-PC

AAC AAA ATG GTT :Koz:strong:Cav:strong:Exon 2:ExonStart false|NKMV*****

AAT CCT ATG GAC :Koz:adequate:Cav:weak:Exon 2:ExonStart false|NPMD

CAC CAG ATG TCC :Koz:weak:Cav:adequate:Exon 2:ExonStart false|HQMS***NonAcetSite**

GGC ATC ATG CAG :Koz:adequate:Cav:strong:Exon 2:ExonStart false|GIMQ

AAG GGA ATG CGG :Koz:adequate:Cav:weak:Exon 2:ExonStart false|KGMR

GAT TTA ATG ATG :Koz:weak:Cav:weak:Exon 2:ExonStart false|DLMM

TTA ATG ATG AAG :Koz:adequate:Cav:adequate:Exon 2:ExonStart false|LMMK

ATG AAG ATG CCG :Koz:adequate:Cav:adequate:Exon 2:ExonStart false|MKMP

CAT GAG ATG ACC :Koz:adequate:Cav:weak:Exon 2:ExonStart false|HEMT

GAG GGT ATG CCC :Koz:adequate:Cav:weak:Exon 2:ExonStart false|EGMP

AAC CCC ATG GAG :Koz:adequate:Cav:adequate:Exon 2:ExonStart false|NPME

CAG CGC ATG GCC :Koz:adequate:Cav:weak:Exon 3:ExonStart false|QRMA

CG8863-PD

AAC AAA ATG GTT :Koz:strong:Cav:strong:Exon 1:ExonStart false|NKMV*****

AAT CCT ATG GAC :Koz:adequate:Cav:weak:Exon 1:ExonStart false|NPMD

CAC CAG ATG TCC :Koz:weak:Cav:adequate:Exon 1:ExonStart false|HQMS***NonAcetSite**

GGC ATC ATG CAG :Koz:adequate:Cav:strong:Exon 1:ExonStart false|GIMQ

AAG GGA ATG CGG :Koz:adequate:Cav:weak:Exon 1:ExonStart false|KGMR

GAT TTA ATG ATG :Koz:weak:Cav:weak:Exon 1:ExonStart false|DLMM

TTA ATG ATG AAG :Koz:adequate:Cav:adequate:Exon 1:ExonStart false|LMMK

ATG AAG ATG CCG :Koz:adequate:Cav:adequate:Exon 1:ExonStart false|MKMP

CAT GAG ATG ACC :Koz:adequate:Cav:weak:Exon 1:ExonStart false|HEMT

GAG GGT ATG CCC :Koz:adequate:Cav:weak:Exon 1:ExonStart false|EGMP

AAC CCC ATG GAG :Koz:adequate:Cav:adequate:Exon 1:ExonStart false|NPME

CAG CGC ATG GCC :Koz:adequate:Cav:weak:Exon 2:ExonStart false|QRMA

CG8863-PE

AAC AAA ATG GTT :Koz:strong:Cav:strong:Exon 3:ExonStart false|NKMV*****

AAT CCT ATG GAC :Koz:adequate:Cav:weak:Exon 3:ExonStart false|NPMD

CAC CAG ATG TCC :Koz:weak:Cav:adequate:Exon 3:ExonStart false|HQMS***NonAcetSite**

GGC ATC ATG CAG :Koz:adequate:Cav:strong:Exon 3:ExonStart false|GIMQ

AAG GGA ATG CGG :Koz:adequate:Cav:weak:Exon 3:ExonStart false|KGMR

GAT TTA ATG ATG :Koz:weak:Cav:weak:Exon 3:ExonStart false|DLMM

TTA ATG ATG AAG :Koz:adequate:Cav:adequate:Exon 3:ExonStart false|LMMK

ATG AAG ATG CCG :Koz:adequate:Cav:adequate:Exon 3:ExonStart false|MKMP

CAT GAG ATG ACC :Koz:adequate:Cav:weak:Exon 3:ExonStart false|HEMT

GAG GGT ATG CCC :Koz:adequate:Cav:weak:Exon 3:ExonStart false|EGMP

AAC CCC ATG GAG :Koz:adequate:Cav:adequate:Exon 3:ExonStart false|NPME

CAG CGC ATG GCC :Koz:adequate:Cav:weak:Exon 4:ExonStart false|QRMA

CG8975-PA

TCT CAA ATG GCG :Koz:adequate:Cav:weak:Exon 1:ExonStart false|SQMA*****

GAC AAC ATG GAG :Koz:strong:Cav:strong:Exon 1:ExonStart false|DNME

CGC AAG ATG TCC :Koz:adequate:Cav:strong:Exon 2:ExonStart false|RKMS

TCC CTC ATG GAG :Koz:adequate:Cav:adequate:Exon 2:ExonStart false|SLME

TGG CAG ATG TAC :Koz:weak:Cav:weak:Exon 2:ExonStart false|WQMT

ATC GCC ATG GAG :Koz:strong:Cav:adequate:Exon 3:ExonStart false|IAME

TCG GAG ATG TAC :Koz:adequate:Cav:weak:Exon 3:ExonStart false|SEMT

GAA ACG ATG CCG :Koz:adequate:Cav:adequate:Exon 3:ExonStart false|ETMP

GGT CTG ATG CCT :Koz:weak:Cav:weak:Exon 3:ExonStart false|GLMP***NonAcetSite**

GTG CTG ATG TTC :Koz:weak:Cav:weak:Exon 3:ExonStart false|VLMF

ATT GGC ATG AAT :Koz:adequate:Cav:weak:Exon 3:ExonStart false|IGMN

GAC CTG ATG TCC :Koz:weak:Cav:adequate:Exon 3:ExonStart false|DLMS

AAC TTC ATG GAG :Koz:adequate:Cav:adequate:Exon 3:ExonStart false|NFME

ATG GAG ATG ATA :Koz:adequate:Cav:weak:Exon 3:ExonStart false|MEMI

CAG CGC ATG GGA :Koz:adequate:Cav:weak:Exon 3:ExonStart false|QRMG***NonAcetSite**

CG9031-PA

ACG AAA ATG AGT :Koz:adequate:Cav:adequate:Exon 1:ExonStart false|TKMS

GCA AAG ATG TCG :Koz:adequate:Cav:adequate:Exon 1:ExonStart false|AKMS***AcetSite**

TTC AAC ATG TCC :Koz:adequate:Cav:strong:Exon 2:ExonStart false|FNMS

TCG TCC ATG CCA :Koz:weak:Cav:weak:Exon 2:ExonStart false|SSMP

TTT GGT ATG GAG :Koz:strong:Cav:weak:Exon 2:ExonStart false|FGME

TCG GTC ATG AAG :Koz:adequate:Cav:weak:Exon 2:ExonStart false|SVMK

ATG AAG ATG GAG :Koz:strong:Cav:adequate:Exon 2:ExonStart false|MKME

CG9277-PA

TAA GTT ATG TTA :Koz:adequate:Cav:weak:Exon 1:ExonStart false|stopVML

GGC ACC ATG GAC :Koz:strong:Cav:strong:Exon 2:ExonStart false|GTMD

TCC GGT ATG GGA :Koz:strong:Cav:adequate:Exon 2:ExonStart false|SGMG***NonAcetSite**

AGG ATC ATG AAC :Koz:adequate:Cav:adequate:Exon 2:ExonStart false|RIMN

CTG ACC ATG TCC :Koz:adequate:Cav:adequate:Exon 2:ExonStart false|LTMS

GTC AAC ATG GTG :Koz:strong:Cav:strong:Exon 2:ExonStart false|VNMV

TTC TTC ATG CCC :Koz:weak:Cav:adequate:Exon 2:ExonStart false|FFMP

CAG CAG ATG TTC :Koz:weak:Cav:weak:Exon 2:ExonStart false|QQMF

AAG AAC ATG ATG :Koz:adequate:Cav:adequate:Exon 2:ExonStart false|KNMM

AAC ATG ATG GCC :Koz:strong:Cav:strong:Exon 2:ExonStart false|NMMA

GGA CGC ATG TCC :Koz:weak:Cav:weak:Exon 2:ExonStart false|GRMS

ATG TCC ATG AAG :Koz:weak:Cav:weak:Exon 2:ExonStart false|MSMK

GAG CAG ATG CTG :Koz:weak:Cav:weak:Exon 2:ExonStart false|EQML

CTG AAG ATG TCC :Koz:adequate:Cav:adequate:Exon 2:ExonStart false|LKMS

ACC GCT ATG TTC :Koz:adequate:Cav:adequate:Exon 2:ExonStart false|TAMF

GAG GGC ATG GAC :Koz:strong:Cav:weak:Exon 2:ExonStart false|EGMD

GAC GAG ATG GAG :Koz:strong:Cav:adequate:Exon 2:ExonStart false|DEME

AGC AAC ATG AAC :Koz:adequate:Cav:strong:Exon 2:ExonStart false|SNMN

CG9277-PB

TAC AAA ATG AGG :Koz:adequate:Cav:strong:Exon 1:ExonStart false|TKMR

GGC ACC ATG GAC :Koz:strong:Cav:strong:Exon 2:ExonStart false|GTMD

TCC GGT ATG GGA :Koz:strong:Cav:adequate:Exon 2:ExonStart false|SGMG***NonAcetSite**

AGG ATC ATG AAC :Koz:adequate:Cav:adequate:Exon 2:ExonStart false|RIMN

CTG ACC ATG TCC :Koz:adequate:Cav:adequate:Exon 2:ExonStart false|LTMS

GTC AAC ATG GTG :Koz:strong:Cav:strong:Exon 2:ExonStart false|VNMV

TTC TTC ATG CCC :Koz:weak:Cav:adequate:Exon 2:ExonStart false|FFMP

CAG CAG ATG TTC :Koz:weak:Cav:weak:Exon 2:ExonStart false|QQMF

AAG AAC ATG ATG :Koz:adequate:Cav:adequate:Exon 2:ExonStart false|KNMM

AAC ATG ATG GCC :Koz:strong:Cav:strong:Exon 2:ExonStart false|NMMA

GGA CGC ATG TCC :Koz:weak:Cav:weak:Exon 2:ExonStart false|GRMS

ATG TCC ATG AAG :Koz:weak:Cav:weak:Exon 2:ExonStart false|MSMK

GAG CAG ATG CTG :Koz:weak:Cav:weak:Exon 2:ExonStart false|EQML

CTG AAG ATG TCC :Koz:adequate:Cav:adequate:Exon 2:ExonStart false|LKMS

ACC GCT ATG TTC :Koz:adequate:Cav:adequate:Exon 2:ExonStart false|TAMF

GAG GGC ATG GAC :Koz:strong:Cav:weak:Exon 2:ExonStart false|EGMD

GAC GAG ATG GAG :Koz:strong:Cav:adequate:Exon 2:ExonStart false|DEME

AGC AAC ATG AAC :Koz:adequate:Cav:strong:Exon 2:ExonStart false|SNMN

CG9277-PC

GGC ACC ATG GAC :Koz:strong:Cav:strong:Exon 2:ExonStart false|GTMD

TCC GGT ATG GGA :Koz:strong:Cav:adequate:Exon 2:ExonStart false|SGMG***NonAcetSite**

AGG ATC ATG AAC :Koz:adequate:Cav:adequate:Exon 2:ExonStart false|RIMN

CTG ACC ATG TCC :Koz:adequate:Cav:adequate:Exon 2:ExonStart false|LTMS

GTC AAC ATG GTG :Koz:strong:Cav:strong:Exon 2:ExonStart false|VNMV

TTC TTC ATG CCC :Koz:weak:Cav:adequate:Exon 2:ExonStart false|FFMP

CAG CAG ATG TTC :Koz:weak:Cav:weak:Exon 2:ExonStart false|QQMF

AAG AAC ATG ATG :Koz:adequate:Cav:adequate:Exon 2:ExonStart false|KNMM

AAC ATG ATG GCC :Koz:strong:Cav:strong:Exon 2:ExonStart false|NMMA

GGA CGC ATG TCC :Koz:weak:Cav:weak:Exon 2:ExonStart false|GRMS

ATG TCC ATG AAG :Koz:weak:Cav:weak:Exon 2:ExonStart false|MSMK

GAG CAG ATG CTG :Koz:weak:Cav:weak:Exon 2:ExonStart false|EQML

CTG AAG ATG TCC :Koz:adequate:Cav:adequate:Exon 2:ExonStart false|LKMS

ACC GCT ATG TTC :Koz:adequate:Cav:adequate:Exon 2:ExonStart false|TAMF

GAG GGC ATG GAC :Koz:strong:Cav:weak:Exon 2:ExonStart false|EGMD

GAC GAG ATG GAG :Koz:strong:Cav:adequate:Exon 2:ExonStart false|DEME

AGC AAC ATG AAC :Koz:adequate:Cav:strong:Exon 2:ExonStart false|SNMN

CG9277-PD

GGC ACC ATG GAC :Koz:strong:Cav:strong:Exon 2:ExonStart false|GTMD

TCC GGT ATG GGA :Koz:strong:Cav:adequate:Exon 2:ExonStart false|SGMG***NonAcetSite**

AGG ATC ATG AAC :Koz:adequate:Cav:adequate:Exon 2:ExonStart false|RIMN

CTG ACC ATG TCC :Koz:adequate:Cav:adequate:Exon 2:ExonStart false|LTMS

GTC AAC ATG GTG :Koz:strong:Cav:strong:Exon 2:ExonStart false|VNMV

TTC TTC ATG CCC :Koz:weak:Cav:adequate:Exon 2:ExonStart false|FFMP

CAG CAG ATG TTC :Koz:weak:Cav:weak:Exon 2:ExonStart false|QQMF

AAG AAC ATG ATG :Koz:adequate:Cav:adequate:Exon 2:ExonStart false|KNMM

AAC ATG ATG GCC :Koz:strong:Cav:strong:Exon 2:ExonStart false|NMMA

GGA CGC ATG TCC :Koz:weak:Cav:weak:Exon 2:ExonStart false|GRMS

ATG TCC ATG AAG :Koz:weak:Cav:weak:Exon 2:ExonStart false|MSMK

GAG CAG ATG CTG :Koz:weak:Cav:weak:Exon 2:ExonStart false|EQML

CTG AAG ATG TCC :Koz:adequate:Cav:adequate:Exon 2:ExonStart false|LKMS

ACC GCT ATG TTC :Koz:adequate:Cav:adequate:Exon 2:ExonStart false|TAMF

GAG GGC ATG GAC :Koz:strong:Cav:weak:Exon 2:ExonStart false|EGMD

GAC GAG ATG GAG :Koz:strong:Cav:adequate:Exon 2:ExonStart false|DEME

AGC AAC ATG AAC :Koz:adequate:Cav:strong:Exon 2:ExonStart false|SNMN

CG9359-PA

ATC AAA ATG CGT :Koz:adequate:Cav:strong:Exon 1:ExonStart false|IKMR

GGC ACC ATG GAT :Koz:strong:Cav:strong:Exon 1:ExonStart false|GTMD

TCC GGC ATG GGA :Koz:strong:Cav:adequate:Exon 2:ExonStart false|SGMG***NonAcetSite**

CGC ATC ATG AAC :Koz:adequate:Cav:strong:Exon 2:ExonStart false|RIMN

GCC ACC ATG TCT :Koz:adequate:Cav:strong:Exon 2:ExonStart false|ATMS

GTG AAC ATG GTA :Koz:strong:Cav:adequate:Exon 2:ExonStart false|VNMV

TTC TTC ATG CCC :Koz:weak:Cav:adequate:Exon 2:ExonStart false|FFMP

CAG CAG ATG TTC :Koz:weak:Cav:weak:Exon 2:ExonStart false|QQMF

AAG AAC ATG ATG :Koz:adequate:Cav:adequate:Exon 2:ExonStart false|KNMM

AAC ATG ATG GCT :Koz:strong:Cav:strong:Exon 2:ExonStart false|NMMA

GGC CGC ATG TCC :Koz:weak:Cav:adequate:Exon 2:ExonStart false|GRMS

ATG TCC ATG AAG :Koz:weak:Cav:weak:Exon 2:ExonStart false|MSMK

GAG CAG ATG CTC :Koz:weak:Cav:weak:Exon 2:ExonStart false|EQML

CTC AAG ATG TCG :Koz:adequate:Cav:strong:Exon 2:ExonStart false|LKMS

ACC GCC ATG TTC :Koz:adequate:Cav:adequate:Exon 2:ExonStart false|TAMF

GAG GGA ATG GAC :Koz:strong:Cav:weak:Exon 2:ExonStart false|EGMD

GAC GAA ATG GAA :Koz:strong:Cav:adequate:Exon 2:ExonStart false|DEME

AGC AAC ATG AAC :Koz:adequate:Cav:strong:Exon 2:ExonStart false|SNMN

CG9373-PA

ACA ACA ATG AGC :Koz:adequate:Cav:adequate:Exon 2:ExonStart false|TTMS*****

ATG AGC ATG GAC :Koz:strong:Cav:adequate:Exon 2:ExonStart false|MSMD***AcetSite**

GAG AAA ATG AAC :Koz:adequate:Cav:adequate:Exon 2:ExonStart false|EKMN

GAC CAC ATG GAT :Koz:adequate:Cav:adequate:Exon 2:ExonStart false|DHMD

TTT AAC ATG ATG :Koz:adequate:Cav:adequate:Exon 3:ExonStart false|FNMM

AAC ATG ATG TCA :Koz:adequate:Cav:strong:Exon 3:ExonStart false|NMMS

ATT TCC ATG CTG :Koz:weak:Cav:weak:Exon 4:ExonStart false|ISML

CGT CAG ATG CTA :Koz:weak:Cav:weak:Exon 4:ExonStart false|RQML

CGT CGT ATG ACC :Koz:weak:Cav:weak:Exon 4:ExonStart false|RRMT

GGC GTT ATG TTT :Koz:adequate:Cav:adequate:Exon 4:ExonStart false|GVMF

GGT GGA ATG GGC :Koz:strong:Cav:weak:Exon 4:ExonStart false|GGMG

GCG CTT ATG GAT :Koz:adequate:Cav:weak:Exon 5:ExonStart false|ALMD

CG9412-PA

CCA GCT ATG GTC :Koz:strong:Cav:weak:Exon 3:ExonStart false|PAMV*****

ATG GTC ATG GAT :Koz:strong:Cav:weak:Exon 3:ExonStart false|MVMD***AcetSite**

CAG CCG ATG CGG :Koz:weak:Cav:weak:Exon 3:ExonStart false|QPMR

CCA GGC ATG CGC :Koz:adequate:Cav:weak:Exon 4:ExonStart false|PGMR

AAC GAC ATG GTG :Koz:strong:Cav:adequate:Exon 5:ExonStart false|NDMV

AAC AAC ATG GGC :Koz:strong:Cav:strong:Exon 5:ExonStart false|NNMG

CGT CCG ATG GGC :Koz:adequate:Cav:weak:Exon 5:ExonStart false|RPMG

GGT GGC ATG ATG :Koz:adequate:Cav:weak:Exon 5:ExonStart false|GGMM

GGC ATG ATG CGC :Koz:adequate:Cav:strong:Exon 5:ExonStart false|GMMR

CG9412-PB

CCA GCT ATG GTC :Koz:strong:Cav:weak:Exon 2:ExonStart false|PAMV*****

ATG GTC ATG GAT :Koz:strong:Cav:weak:Exon 2:ExonStart false|MVMD***AcetSite**

CAG CCG ATG CGG :Koz:weak:Cav:weak:Exon 2:ExonStart false|QPMR

CCA GGC ATG CGC :Koz:adequate:Cav:weak:Exon 3:ExonStart false|PGMR

AAC GAC ATG GTG :Koz:strong:Cav:adequate:Exon 4:ExonStart false|NDMV

AAC AAC ATG GGC :Koz:strong:Cav:strong:Exon 4:ExonStart false|NNMG

CGT CCG ATG GGC :Koz:adequate:Cav:weak:Exon 4:ExonStart false|RPMG

GGT GGC ATG ATG :Koz:adequate:Cav:weak:Exon 4:ExonStart false|GGMM

GGC ATG ATG CGC :Koz:adequate:Cav:strong:Exon 4:ExonStart false|GMMR

CG9412-PC

CCA GCT ATG GTC :Koz:strong:Cav:weak:Exon 2:ExonStart false|PAMV*****

ATG GTC ATG GAT :Koz:strong:Cav:weak:Exon 2:ExonStart false|MVMD***AcetSite**

CAG CCG ATG CGG :Koz:weak:Cav:weak:Exon 2:ExonStart false|QPMR

CCA GGC ATG CGC :Koz:adequate:Cav:weak:Exon 3:ExonStart false|PGMR

AAC GAC ATG GTG :Koz:strong:Cav:adequate:Exon 4:ExonStart false|NDMV

AAC AAC ATG GGC :Koz:strong:Cav:strong:Exon 4:ExonStart false|NNMG

CGT CCG ATG GGC :Koz:adequate:Cav:weak:Exon 4:ExonStart false|RPMG

GGT GGC ATG ATG :Koz:adequate:Cav:weak:Exon 4:ExonStart false|GGMM

GGC ATG ATG CGC :Koz:adequate:Cav:strong:Exon 4:ExonStart false|GMMR

CG9412-PD

CCA GCT ATG GTC :Koz:strong:Cav:weak:Exon 1:ExonStart false|PAMV*****

ATG GTC ATG GAT :Koz:strong:Cav:weak:Exon 1:ExonStart false|MVMD***AcetSite**

CAG CCG ATG CGG :Koz:weak:Cav:weak:Exon 1:ExonStart false|QPMR

CCA GGC ATG CGC :Koz:adequate:Cav:weak:Exon 2:ExonStart false|PGMR

AAC GAC ATG GTG :Koz:strong:Cav:adequate:Exon 3:ExonStart false|NDMV

AAC AAC ATG GGC :Koz:strong:Cav:strong:Exon 3:ExonStart false|NNMG

CGT CCG ATG GGC :Koz:adequate:Cav:weak:Exon 3:ExonStart false|RPMG

GGT GGC ATG ATG :Koz:adequate:Cav:weak:Exon 3:ExonStart false|GGMM

GGC ATG ATG CGC :Koz:adequate:Cav:strong:Exon 3:ExonStart false|GMMR

CG9412-PE

CCA GCT ATG GTC :Koz:strong:Cav:weak:Exon 3:ExonStart false|PAMV*****

ATG GTC ATG GAT :Koz:strong:Cav:weak:Exon 3:ExonStart false|MVMD***AcetSite**

CAG CCG ATG CGG :Koz:weak:Cav:weak:Exon 3:ExonStart false|QPMR

CCA GGC ATG CGC :Koz:adequate:Cav:weak:Exon 4:ExonStart false|PGMR

AAC GAC ATG GTG :Koz:strong:Cav:adequate:Exon 5:ExonStart false|NDMV

AAC AAC ATG GGC :Koz:strong:Cav:strong:Exon 5:ExonStart false|NNMG

CGT CCG ATG GGC :Koz:adequate:Cav:weak:Exon 5:ExonStart false|RPMG

GGT GGC ATG ATG :Koz:adequate:Cav:weak:Exon 5:ExonStart false|GGMM

GGC ATG ATG CGC :Koz:adequate:Cav:strong:Exon 5:ExonStart false|GMMR

CG9579-PA

TGC ATA ATG GAA :Koz:strong:Cav:strong:Exon 1:ExonStart false|CIME*****

GCG GCG ATG AAG :Koz:adequate:Cav:weak:Exon 2:ExonStart false|AAMK

GGT CTA ATG ATG :Koz:weak:Cav:weak:Exon 2:ExonStart false|GLMM

CTA ATG ATG CCA :Koz:adequate:Cav:adequate:Exon 2:ExonStart false|LMMP

GCC GCC ATG GCG :Koz:strong:Cav:adequate:Exon 2:ExonStart false|AAMA

GAG GAG ATG GCC :Koz:strong:Cav:weak:Exon 2:ExonStart false|EEMA

GAG CAG ATG TGC :Koz:weak:Cav:weak:Exon 2:ExonStart false|EQMC***NonAcetSite**

CGG ATC ATG TCG :Koz:adequate:Cav:adequate:Exon 2:ExonStart false|RIMS

CAC GAG ATG TCC :Koz:adequate:Cav:adequate:Exon 2:ExonStart false|HEMS

GAG GCC ATG ATG :Koz:adequate:Cav:weak:Exon 2:ExonStart false|EAMM

GCC ATG ATG GCC :Koz:strong:Cav:strong:Exon 2:ExonStart false|AMMA

AAG GCC ATG AAT :Koz:adequate:Cav:weak:Exon 3:ExonStart false|KAMN

CG9749-PA

ACA CCC ATG TTG :Koz:weak:Cav:weak:Exon 2:ExonStart false|TPML*****

ACC CCC ATG GCC :Koz:adequate:Cav:adequate:Exon 2:ExonStart false|TPMA***AcetSite**

AAC ATC ATG GAC :Koz:strong:Cav:strong:Exon 2:ExonStart false|NIMD***AcetSite**

AGC TAC ATG CAG :Koz:weak:Cav:adequate:Exon 2:ExonStart false|STMQ

GGC GAG ATG GAG :Koz:strong:Cav:adequate:Exon 2:ExonStart false|GEME

TCC CAG ATG AAC :Koz:weak:Cav:adequate:Exon 2:ExonStart false|SQMN

TAC TCG ATG TTG :Koz:weak:Cav:adequate:Exon 3:ExonStart false|TSML

CCG CAG ATG TCG :Koz:weak:Cav:weak:Exon 3:ExonStart false|PQMS

AAG CGA ATG TCG :Koz:weak:Cav:weak:Exon 3:ExonStart false|KRMS

TCA ACG ATG ACT :Koz:adequate:Cav:adequate:Exon 3:ExonStart false|STMT

CTT CCG ATG CCA :Koz:weak:Cav:weak:Exon 3:ExonStart false|LPMP

GCG GGC ATG ATG :Koz:adequate:Cav:weak:Exon 3:ExonStart false|AGMM

GGC ATG ATG CAA :Koz:adequate:Cav:strong:Exon 3:ExonStart false|GMMQ

AGC AGC ATG CCA :Koz:adequate:Cav:strong:Exon 3:ExonStart false|SSMP

CAC GAG ATG ACC :Koz:adequate:Cav:adequate:Exon 4:ExonStart false|HEMT

ATT GGC ATG CAC :Koz:adequate:Cav:weak:Exon 4:ExonStart false|IGMH

GGC GTC ATG GAT :Koz:strong:Cav:adequate:Exon 6:ExonStart false|GVMD

CG9759-PA

GGA ACG ATG CGT :Koz:adequate:Cav:adequate:Exon 1:ExonStart false|GTMR

CAG GAG ATG CCG :Koz:adequate:Cav:weak:Exon 1:ExonStart false|QEMP***NonAcetSite**

CG9759-PB

GGA ACG ATG CGT :Koz:adequate:Cav:adequate:Exon 1:ExonStart false|GTMR

CAG GAG ATG CCG :Koz:adequate:Cav:weak:Exon 1:ExonStart false|QEMP***NonAcetSite**

CG9765-PA

TAT GCC ATG GAA :Koz:strong:Cav:weak:Exon 1:ExonStart false|TAME*****

CTA GGA ATG GGA :Koz:strong:Cav:weak:Exon 1:ExonStart false|LGMG

ACG AGC ATG GAT :Koz:strong:Cav:adequate:Exon 2:ExonStart false|TSMD

AAG GCA ATG GCG :Koz:strong:Cav:weak:Exon 2:ExonStart false|KAMA

ACC GAT ATG GAT :Koz:strong:Cav:adequate:Exon 2:ExonStart false|TDMD

CTG TCC ATG ACC :Koz:weak:Cav:weak:Exon 3:ExonStart false|LSMT

GAA GCC ATG GAT :Koz:strong:Cav:weak:Exon 3:ExonStart false|EAMD

GAA ACC ATG GAA :Koz:strong:Cav:adequate:Exon 3:ExonStart false|ETME

ATG GAA ATG TTA :Koz:adequate:Cav:weak:Exon 3:ExonStart false|MEML

CAT CTT ATG CAA :Koz:weak:Cav:weak:Exon 3:ExonStart false|HLMQ

GAG GAA ATG CAA :Koz:adequate:Cav:weak:Exon 3:ExonStart false|EEMQ

GAA TCA ATG GAG :Koz:adequate:Cav:weak:Exon 3:ExonStart false|ESME

CCA GCT ATG GAG :Koz:strong:Cav:weak:Exon 3:ExonStart false|PAME

GAT CAA ATG GAT :Koz:adequate:Cav:weak:Exon 3:ExonStart false|DQMD

GAA TCC ATG AAA :Koz:weak:Cav:weak:Exon 3:ExonStart false|ESMK

AAT AAA ATG GAT :Koz:strong:Cav:adequate:Exon 3:ExonStart false|NKMD

TTA AAG ATG ACT :Koz:adequate:Cav:adequate:Exon 3:ExonStart false|LKMT

AAG GAC ATG CCC :Koz:adequate:Cav:weak:Exon 3:ExonStart false|KDMP

TTG CCA ATG AGC :Koz:weak:Cav:weak:Exon 3:ExonStart false|LPMS

TCA AAT ATG ACC :Koz:adequate:Cav:adequate:Exon 3:ExonStart false|SNMT

TTC TGT ATG GAA :Koz:adequate:Cav:adequate:Exon 3:ExonStart false|FCME

TTC TGC ATG CAG :Koz:weak:Cav:adequate:Exon 3:ExonStart false|FCMQ

GAA GTT ATG GAT :Koz:strong:Cav:weak:Exon 3:ExonStart false|EVMD

ATC TCC ATG AGG :Koz:weak:Cav:adequate:Exon 3:ExonStart false|ISMR

GCC GTG ATG AAG :Koz:adequate:Cav:adequate:Exon 3:ExonStart false|AVMK

GAT GAC ATG TTT :Koz:adequate:Cav:weak:Exon 3:ExonStart false|DDMF

TCT GCG ATG ATC :Koz:adequate:Cav:weak:Exon 3:ExonStart false|SAMI

GTT AAA ATG AGT :Koz:adequate:Cav:adequate:Exon 5:ExonStart false|VKMS

AAC GAT ATG GAT :Koz:strong:Cav:adequate:Exon 6:ExonStart false|NDMD***AcetSite**

AAA GAG ATG ACC :Koz:adequate:Cav:weak:Exon 9:ExonStart false|KEMT

AAG CAG ATG ATG :Koz:weak:Cav:weak:Exon 9:ExonStart false|KQMM

CAG ATG ATG GAC :Koz:strong:Cav:adequate:Exon 9:ExonStart false|QMMD

GAC AAG ATG AAG :Koz:adequate:Cav:strong:Exon 9:ExonStart false|DKMK

CAT GCC ATG CAG :Koz:adequate:Cav:weak:Exon 9:ExonStart false|HAMQ

GTC TCG ATG ACG :Koz:weak:Cav:adequate:Exon 10:ExonStart false|VSMT

CG9765-PB

TAT GCC ATG GAA :Koz:strong:Cav:weak:Exon 1:ExonStart false|TAME*****

CTA GGA ATG GGA :Koz:strong:Cav:weak:Exon 1:ExonStart false|LGMG

ACG AGC ATG GAT :Koz:strong:Cav:adequate:Exon 2:ExonStart false|TSMD

AAG GCA ATG GCG :Koz:strong:Cav:weak:Exon 2:ExonStart false|KAMA

ACC GAT ATG GAT :Koz:strong:Cav:adequate:Exon 2:ExonStart false|TDMD

CTG TCC ATG ACC :Koz:weak:Cav:weak:Exon 3:ExonStart false|LSMT

GAA GCC ATG GAT :Koz:strong:Cav:weak:Exon 3:ExonStart false|EAMD

GAA ACC ATG GAA :Koz:strong:Cav:adequate:Exon 3:ExonStart false|ETME

ATG GAA ATG TTA :Koz:adequate:Cav:weak:Exon 3:ExonStart false|MEML

CAT CTT ATG CAA :Koz:weak:Cav:weak:Exon 3:ExonStart false|HLMQ

GAG GAA ATG CAA :Koz:adequate:Cav:weak:Exon 3:ExonStart false|EEMQ

GAA TCA ATG GAG :Koz:adequate:Cav:weak:Exon 3:ExonStart false|ESME

CCA GCT ATG GAG :Koz:strong:Cav:weak:Exon 3:ExonStart false|PAME

GAT CAA ATG GAT :Koz:adequate:Cav:weak:Exon 3:ExonStart false|DQMD

GAA TCC ATG AAA :Koz:weak:Cav:weak:Exon 3:ExonStart false|ESMK

AAT AAA ATG GAT :Koz:strong:Cav:adequate:Exon 3:ExonStart false|NKMD

TTA AAG ATG ACT :Koz:adequate:Cav:adequate:Exon 3:ExonStart false|LKMT

AAG GAC ATG CCC :Koz:adequate:Cav:weak:Exon 3:ExonStart false|KDMP

TTG CCA ATG AGC :Koz:weak:Cav:weak:Exon 3:ExonStart false|LPMS

TCA AAT ATG ACC :Koz:adequate:Cav:adequate:Exon 3:ExonStart false|SNMT

GAA GTT ATG GAT :Koz:strong:Cav:weak:Exon 4:ExonStart false|EVMD

ATC TCC ATG AGG :Koz:weak:Cav:adequate:Exon 4:ExonStart false|ISMR

GCC GTG ATG AAG :Koz:adequate:Cav:adequate:Exon 4:ExonStart false|AVMK

GAT GAC ATG TTT :Koz:adequate:Cav:weak:Exon 4:ExonStart false|DDMF

TCT GCG ATG ATC :Koz:adequate:Cav:weak:Exon 4:ExonStart false|SAMI

GTT AAA ATG AGT :Koz:adequate:Cav:adequate:Exon 6:ExonStart false|VKMS

AAC GAT ATG GAT :Koz:strong:Cav:adequate:Exon 7:ExonStart false|NDMD***AcetSite**

AAA GAG ATG ACC :Koz:adequate:Cav:weak:Exon 10:ExonStart false|KEMT

AAG CAG ATG ATG :Koz:weak:Cav:weak:Exon 10:ExonStart false|KQMM

CAG ATG ATG GAC :Koz:strong:Cav:adequate:Exon 10:ExonStart false|QMMD

GAC AAG ATG AAG :Koz:adequate:Cav:strong:Exon 10:ExonStart false|DKMK

CAT GCC ATG CAG :Koz:adequate:Cav:weak:Exon 10:ExonStart false|HAMQ

GTC TCG ATG ACG :Koz:weak:Cav:adequate:Exon 11:ExonStart false|VSMT

CG9765-PC

AGC GGC ATG GAT :Koz:strong:Cav:adequate:Exon 1:ExonStart false|SGMD

ACG ACA ATG GAA :Koz:strong:Cav:adequate:Exon 1:ExonStart false|TTME

CTA TTT ATG GAA :Koz:adequate:Cav:weak:Exon 1:ExonStart false|LFME

AAC GAA ATG GAG :Koz:strong:Cav:adequate:Exon 1:ExonStart false|NEME

CAA GCC ATG AGT :Koz:adequate:Cav:weak:Exon 1:ExonStart false|QAMS

ACG AGC ATG GAT :Koz:strong:Cav:adequate:Exon 2:ExonStart false|TSMD

AAG GCA ATG GCG :Koz:strong:Cav:weak:Exon 2:ExonStart false|KAMA

ACC GAT ATG GAT :Koz:strong:Cav:adequate:Exon 2:ExonStart false|TDMD

CTG TCC ATG ACC :Koz:weak:Cav:weak:Exon 3:ExonStart false|LSMT

GAA GCC ATG GAT :Koz:strong:Cav:weak:Exon 3:ExonStart false|EAMD

GAA ACC ATG GAA :Koz:strong:Cav:adequate:Exon 3:ExonStart false|ETME

ATG GAA ATG TTA :Koz:adequate:Cav:weak:Exon 3:ExonStart false|MEML

CAT CTT ATG CAA :Koz:weak:Cav:weak:Exon 3:ExonStart false|HLMQ

GAG GAA ATG CAA :Koz:adequate:Cav:weak:Exon 3:ExonStart false|EEMQ

GAA TCA ATG GAG :Koz:adequate:Cav:weak:Exon 3:ExonStart false|ESME

CCA GCT ATG GAG :Koz:strong:Cav:weak:Exon 3:ExonStart false|PAME

GAT CAA ATG GAT :Koz:adequate:Cav:weak:Exon 3:ExonStart false|DQMD

GAA TCC ATG AAA :Koz:weak:Cav:weak:Exon 3:ExonStart false|ESMK

AAT AAA ATG GAT :Koz:strong:Cav:adequate:Exon 3:ExonStart false|NKMD

TTA AAG ATG ACT :Koz:adequate:Cav:adequate:Exon 3:ExonStart false|LKMT

AAG GAC ATG CCC :Koz:adequate:Cav:weak:Exon 3:ExonStart false|KDMP

TTG CCA ATG AGC :Koz:weak:Cav:weak:Exon 3:ExonStart false|LPMS

TCA AAT ATG ACC :Koz:adequate:Cav:adequate:Exon 3:ExonStart false|SNMT

TTC TGT ATG GAA :Koz:adequate:Cav:adequate:Exon 3:ExonStart false|FCME

TTC TGC ATG CAG :Koz:weak:Cav:adequate:Exon 3:ExonStart false|FCMQ

GAA GTT ATG GAT :Koz:strong:Cav:weak:Exon 3:ExonStart false|EVMD

ATC TCC ATG AGG :Koz:weak:Cav:adequate:Exon 3:ExonStart false|ISMR

GCC GTG ATG AAG :Koz:adequate:Cav:adequate:Exon 3:ExonStart false|AVMK

GAT GAC ATG TTT :Koz:adequate:Cav:weak:Exon 3:ExonStart false|DDMF

TCT GCG ATG ATC :Koz:adequate:Cav:weak:Exon 3:ExonStart false|SAMI

GTT AAA ATG AGT :Koz:adequate:Cav:adequate:Exon 5:ExonStart false|VKMS

AAC GAT ATG GAT :Koz:strong:Cav:adequate:Exon 6:ExonStart false|NDMD***AcetSite**

AAA GAG ATG ACC :Koz:adequate:Cav:weak:Exon 9:ExonStart false|KEMT

AAG CAG ATG ATG :Koz:weak:Cav:weak:Exon 9:ExonStart false|KQMM

CAG ATG ATG GAC :Koz:strong:Cav:adequate:Exon 9:ExonStart false|QMMD

GAC AAG ATG AAG :Koz:adequate:Cav:strong:Exon 9:ExonStart false|DKMK

CAT GCC ATG CAG :Koz:adequate:Cav:weak:Exon 9:ExonStart false|HAMQ

GTC TCG ATG ACG :Koz:weak:Cav:adequate:Exon 10:ExonStart false|VSMT

CG9765-PD

AGC GGC ATG GAT :Koz:strong:Cav:adequate:Exon 1:ExonStart false|SGMD

ACG ACA ATG GAA :Koz:strong:Cav:adequate:Exon 1:ExonStart false|TTME

CTA TTT ATG GAA :Koz:adequate:Cav:weak:Exon 1:ExonStart false|LFME

ACG AGC ATG GAT :Koz:strong:Cav:adequate:Exon 2:ExonStart false|TSMD

AAG GCA ATG GCG :Koz:strong:Cav:weak:Exon 2:ExonStart false|KAMA

ACC GAT ATG GAT :Koz:strong:Cav:adequate:Exon 2:ExonStart false|TDMD

CTG TCC ATG ACC :Koz:weak:Cav:weak:Exon 3:ExonStart false|LSMT

GAA GCC ATG GAT :Koz:strong:Cav:weak:Exon 3:ExonStart false|EAMD

GAA ACC ATG GAA :Koz:strong:Cav:adequate:Exon 3:ExonStart false|ETME

ATG GAA ATG TTA :Koz:adequate:Cav:weak:Exon 3:ExonStart false|MEML

CAT CTT ATG CAA :Koz:weak:Cav:weak:Exon 3:ExonStart false|HLMQ

GAG GAA ATG CAA :Koz:adequate:Cav:weak:Exon 3:ExonStart false|EEMQ

GAA TCA ATG GAG :Koz:adequate:Cav:weak:Exon 3:ExonStart false|ESME

CCA GCT ATG GAG :Koz:strong:Cav:weak:Exon 3:ExonStart false|PAME

GAT CAA ATG GAT :Koz:adequate:Cav:weak:Exon 3:ExonStart false|DQMD

GAA TCC ATG AAA :Koz:weak:Cav:weak:Exon 3:ExonStart false|ESMK

AAT AAA ATG GAT :Koz:strong:Cav:adequate:Exon 3:ExonStart false|NKMD

TTA AAG ATG ACT :Koz:adequate:Cav:adequate:Exon 3:ExonStart false|LKMT

AAG GAC ATG CCC :Koz:adequate:Cav:weak:Exon 3:ExonStart false|KDMP

TTG CCA ATG AGC :Koz:weak:Cav:weak:Exon 3:ExonStart false|LPMS

TCA AAT ATG ACC :Koz:adequate:Cav:adequate:Exon 3:ExonStart false|SNMT

TTC TGT ATG GAA :Koz:adequate:Cav:adequate:Exon 3:ExonStart false|FCME

TTC TGC ATG CAG :Koz:weak:Cav:adequate:Exon 3:ExonStart false|FCMQ

GAA GTT ATG GAT :Koz:strong:Cav:weak:Exon 3:ExonStart false|EVMD

ATC TCC ATG AGG :Koz:weak:Cav:adequate:Exon 3:ExonStart false|ISMR

GCC GTG ATG AAG :Koz:adequate:Cav:adequate:Exon 3:ExonStart false|AVMK

GAT GAC ATG TTT :Koz:adequate:Cav:weak:Exon 3:ExonStart false|DDMF

TCT GCG ATG ATC :Koz:adequate:Cav:weak:Exon 3:ExonStart false|SAMI

GTT AAA ATG AGT :Koz:adequate:Cav:adequate:Exon 5:ExonStart false|VKMS

AAC GAT ATG GAT :Koz:strong:Cav:adequate:Exon 6:ExonStart false|NDMD***AcetSite**

AAA GAG ATG ACC :Koz:adequate:Cav:weak:Exon 9:ExonStart false|KEMT

AAG CAG ATG ATG :Koz:weak:Cav:weak:Exon 9:ExonStart false|KQMM

CAG ATG ATG GAC :Koz:strong:Cav:adequate:Exon 9:ExonStart false|QMMD

GAC AAG ATG AAG :Koz:adequate:Cav:strong:Exon 9:ExonStart false|DKMK

CAT GCC ATG CAG :Koz:adequate:Cav:weak:Exon 9:ExonStart false|HAMQ

GTC TCG ATG ACG :Koz:weak:Cav:adequate:Exon 10:ExonStart false|VSMT

CG9765-PE

GTT AAA ATG AGT :Koz:adequate:Cav:adequate:Exon 2:ExonStart false|VKMS

AAC GAT ATG GAT :Koz:strong:Cav:adequate:Exon 3:ExonStart false|NDMD***AcetSite**

AAA GAG ATG ACC :Koz:adequate:Cav:weak:Exon 6:ExonStart false|KEMT

AAG CAG ATG ATG :Koz:weak:Cav:weak:Exon 6:ExonStart false|KQMM

CAG ATG ATG GAC :Koz:strong:Cav:adequate:Exon 6:ExonStart false|QMMD

GAC AAG ATG AAG :Koz:adequate:Cav:strong:Exon 6:ExonStart false|DKMK

CAT GCC ATG CAG :Koz:adequate:Cav:weak:Exon 6:ExonStart false|HAMQ

GTC TCG ATG ACG :Koz:weak:Cav:adequate:Exon 7:ExonStart false|VSMT

CG9765-PF

GTT AAA ATG AGT :Koz:adequate:Cav:adequate:Exon 3:ExonStart false|VKMS

AAC GAT ATG GAT :Koz:strong:Cav:adequate:Exon 4:ExonStart false|NDMD***AcetSite**

AAA GAG ATG ACC :Koz:adequate:Cav:weak:Exon 7:ExonStart false|KEMT

AAG CAG ATG ATG :Koz:weak:Cav:weak:Exon 7:ExonStart false|KQMM

CAG ATG ATG GAC :Koz:strong:Cav:adequate:Exon 7:ExonStart false|QMMD

GAC AAG ATG AAG :Koz:adequate:Cav:strong:Exon 7:ExonStart false|DKMK

CAT GCC ATG CAG :Koz:adequate:Cav:weak:Exon 7:ExonStart false|HAMQ

GTC TCG ATG ACG :Koz:weak:Cav:adequate:Exon 8:ExonStart false|VSMT

CG9765-PG

GTT AAA ATG AGT :Koz:adequate:Cav:adequate:Exon 3:ExonStart false|VKMS

AAC GAT ATG GAT :Koz:strong:Cav:adequate:Exon 4:ExonStart false|NDMD***AcetSite**

AAA GAG ATG ACC :Koz:adequate:Cav:weak:Exon 7:ExonStart false|KEMT

AAG CAG ATG ATG :Koz:weak:Cav:weak:Exon 7:ExonStart false|KQMM

CAG ATG ATG GAC :Koz:strong:Cav:adequate:Exon 7:ExonStart false|QMMD

GAC AAG ATG AAG :Koz:adequate:Cav:strong:Exon 7:ExonStart false|DKMK

CAT GCC ATG CAG :Koz:adequate:Cav:weak:Exon 7:ExonStart false|HAMQ

GTC TCG ATG ACG :Koz:weak:Cav:adequate:Exon 8:ExonStart false|VSMT

CG9795-PC

CAC CGT ATG CAA :Koz:weak:Cav:adequate:Exon 1:ExonStart false|HRMQ

TTT TGG ATG GCC :Koz:adequate:Cav:weak:Exon 1:ExonStart false|FWMA***AcetSite**

AGC AAC ATG AGC :Koz:adequate:Cav:strong:Exon 2:ExonStart false|SNMS

AAG CAA ATG TTT :Koz:weak:Cav:weak:Exon 3:ExonStart false|KQMF

TAT CCG ATG CTC :Koz:weak:Cav:weak:Exon 4:ExonStart false|TPML

AAC AAC ATG ATG :Koz:adequate:Cav:strong:Exon 4:ExonStart false|NNMM

AAC ATG ATG GGA :Koz:strong:Cav:strong:Exon 4:ExonStart false|NMMG

GCT CTA ATG GAA :Koz:adequate:Cav:weak:Exon 5:ExonStart false|ALME

GGG GGC ATG CGG :Koz:adequate:Cav:weak:Exon 6:ExonStart false|GGMR

CCA GGA ATG ATG :Koz:adequate:Cav:weak:Exon 7:ExonStart false|PGMM

GGA ATG ATG ACG :Koz:adequate:Cav:adequate:Exon 7:ExonStart false|GMMT

AAA TTA ATG GTT :Koz:adequate:Cav:weak:Exon 8:ExonStart false|KLMV

CAA GTA ATG CAG :Koz:adequate:Cav:weak:Exon 9:ExonStart false|QVMQ

GGA ACA ATG CAT :Koz:adequate:Cav:adequate:Exon 9:ExonStart false|GTMH

GO Slim analysis

To assess whether particular protein functions or functional domains are associated with the N-terminal acetylation state, a Gene Ontology analysis on a reduced set of GO categories (referred to as GO Slim) on all three levels, namely *Cellular Component*, *Molecular Function* and *Biological Process* was performed [1]. For all three levels we see an overrepresentation both for the acetylated and for the non-acetylated gene models (p-value for both <0.05), such as the GO categories *cytoplasm* or *cytosol* (Supplemental Figure S2A) or *metabolic process* and *translation* (Supplemental Figure S2C). This indicates that proteins derived from these gene models are overrepresented in our dataset but have no preference for a specific acetylation state. Similarly, other categories like *transcription factor activity* and *receptor activity* (Supplemental Figure S2B) are underrepresented (p<0.05) in the dataset independent of the acetylation state of the identified proteins. For other categories we observe a statistically significant overrepresentation (e.g. *nucleus* (Supplemental Figure S2A), cell-cell signaling (Supplemental Figure S2C)) of acetylated N-termini (p<0.05) indicating that proteins that derive from genes of these GO-categories are preferentially acetylated. Inversely, we observe a statistical overrepresentation of the category *structural molecule activity correlated* with non-acetylated amino-termini (p<0.05, Supplemental Figure S2B). Likewise non-acetylated amino-termini were underrepresented for *transcription regulator activity* and *protein kinase activity* (p<0.05, Supplemental Figure S2B). The results of this analysis are shown in Table 5 as well as in Supplemental Figure S2A-C. However, despite the fact that some categories show a statistically significant (p<0.05) over- or underrepresentation of either acetylated or free N-termini, the overall spread of the distributions of acetylated versus non-acetylated gene models does not allow to make a clear correlation of protein function with a certain GO-category or a group of GO-categories.

1. Ashburner M, Ball CA, Blake JA, Botstein D, Butler H, et al. (2000) Gene ontology: tool for the unification of biology. The Gene Ontology Consortium. Nat Genet 25: 25-29.
